# A newly emerging alphasatellite affects banana bunchy top virus replication, transcription, siRNA production and transmission by aphids

**DOI:** 10.1371/journal.ppat.1010448

**Published:** 2022-04-12

**Authors:** Valentin Guyot, Rajendran Rajeswaran, Huong Cam Chu, Chockalingam Karthikeyan, Nathalie Laboureau, Serge Galzi, Lyna F. T. Mukwa, Mart Krupovic, P. Lava Kumar, Marie-Line Iskra-Caruana, Mikhail M. Pooggin

**Affiliations:** 1 PHIM Plant Health Institute, University of Montpellier, INRAE, CIRAD, IRD, Institute Agro, Montpellier, France; 2 Faculté des Sciences Agronomiques, Université Pédagogique Nationale, Kinshasa, Democratic Republic of the Congo; 3 Institut Pasteur, Université Paris Cité, CNRS UMR6047, Archaeal Virology Unit, Paris, France; 4 International Institute of Tropical Agriculture (IITA), Ibadan, Nigeria; 5 CIRAD, DGD-RS, Montpellier, France; The Ohio State University, UNITED STATES

## Abstract

Banana bunchy top virus (BBTV) is a six-component ssDNA virus (genus *Babuvirus*, family *Nanoviridae*) transmitted by aphids, infecting monocots (mainly species in the family Musaceae) and likely originating from South-East Asia where it is frequently associated with self-replicating alphasatellites. Illumina sequencing analysis of banana aphids and leaf samples from Africa revealed an alphasatellite that should be classified in a new genus, phylogenetically related to alphasatellites of nanoviruses infecting dicots. Alphasatellite DNA was encapsidated by BBTV coat protein and accumulated at high levels in plants and aphids, thereby reducing helper virus loads, altering relative abundance (formula) of viral genome components and interfering with virus transmission by aphids. BBTV and alphasatellite clones infected dicot *Nicotiana benthamiana*, followed by recovery and symptomless persistence of alphasatellite, and BBTV replication protein (Rep), but not alphasatellite Rep, induced leaf chlorosis. Transcriptome sequencing revealed 21, 22 and 24 nucleotide small interfering (si)RNAs covering both strands of the entire viral genome, monodirectional Pol II transcription units of viral mRNAs and pervasive transcription of each component and alphasatellite in both directions, likely generating double-stranded precursors of viral siRNAs. Consistent with the latter hypothesis, viral DNA formulas with and without alphasatellite resembled viral siRNA formulas but not mRNA formulas. Alphasatellite decreased transcription efficiency of DNA-N encoding a putative aphid transmission factor and increased relative siRNA production rates from Rep- and movement protein-encoding components. Alphasatellite itself spawned the most abundant siRNAs and had the lowest mRNA transcription rate. Collectively, following African invasion, BBTV got associated with an alphasatellite likely originating from a dicot plant and interfering with BBTV replication and transmission. Molecular analysis of virus-infected banana plants revealed new features of viral DNA transcription and siRNA biogenesis, both affected by alphasatellite. Costs and benefits of alphasatellite association with helper viruses are discussed.

## Author summary

Self-replicating alphasatellites are frequently associated with plant ssDNA viruses. Their origin and costs versus benefits for helper virus replication, antiviral defense evasion and transmission by insect vectors are poorly understood. Here we describe identification in Africa and in depth molecular and biological characterization of a newly emerging alphasatellite of BBTV, a multicomponent ssDNA babuvirus causing one of the most economically-important diseases of monocotyledonous bananas and plantains. Phylogenetically, this alphasatellite represents a novel genus and is more related to alphasatellites of nanoviruses infecting dicot hosts than to other BBTV alphasatellites previously identified only in Asia. Consistent with its hypothetical dicot origin, cloned alphasatellite and BBTV can establish systemic infection in a model dicot plant, followed by recovery and symptomless alphasatellite persistence. In banana plants, alphasatellite competes for the host replication and transcription machinery and accumulates at high levels, thereby reducing loads of the helper virus, modifying relative abundance of its components and interfering with its acquisition and transmission by aphids. On the other hand, plant antiviral defenses silence alphasatellite gene expression at both transcriptional and posttranscriptional levels, generating highly-abundant 21, 22 and 24 nucleotide small interfering RNAs, suggesting that alphasatellite may serve as a decoy protecting its helper virus from gene silencing.

## Introduction

Bananas and plantains (genus *Musa*, family Musaceae) are monocots susceptible to many diseases and pests. Banana bunchy top disease (BBTD) is one of the most economically important, causing up to 100% yield losses [1,2]. BBTD symptoms include discontinuous dark-green streaks on the leaf veins, midrib, petioles and pseudostem, chlorosis of leaf margins and strong reduction in size of leaves and petioles, resulting in a foliar bunch at the top of stunted plant [1,3]. BBTD is caused by banana bunchy top virus (BBTV) which is restricted to phloem tissues and transmitted by the banana aphid *Pentalonia nigronervosa* in a persistent circulative non-propagative manner [4–7]. BBTV host range was so far found to be restricted to monocots mainly from the families Musaceae and Zingiberaceae [8].

BBTV has a six-component circular single-stranded (ss)DNA genome and is classified within the genus *Babuvirus* of family *Nanoviridae* [9–12]. Each circular ssDNA component of the BBTV genome has a size of ca. 1.0 to 1.1 Kb and is individually encapsidated within isometric 18–20 nm virions [13]. BBTV replicates in the nucleus via a rolling circle mechanism mediated by viral replication protein (Rep) and the host replication machinery. Each BBTV component, namely DNAs C, M, N, R, S and U3, possesses a single ORF preceded by the host RNA polymerase II (Pol II) promoter region with a TATA or TATA-like box and followed by the Pol II terminator region with a polyadenylation signal [14–16]. DNA-R encodes a master Rep protein mediating replication of DNA-R itself and transreplication of other components [14,17]. DNA-C encodes a cell-cycle link protein (Clink) enhancing viral replication [18, 19]. DNA-M encodes a movement protein (MP) [20]. DNA-S encodes a coat protein (CP) encapsidating all viral ssDNA components [21]. DNA-N encodes a nuclear shuttle protein (NSP) [20], whose homologue is required for aphid transmission of eight-component ssDNA viruses from the genus *Nanovirus* of family *Nanoviridae* [22,23]. DNA-U3 encodes a small protein of unknown function [15]. All six components contain two regions of high sequence similarity: a common region stem-loop (CR-SL) and a common region major (CR-M) [10]. The CR-SL is an origin of replication containing a highly conserved nonanucleotide sequence TATTATTAC and three 5-bp-long repeats (iterons) that are likely involved in specific recognition and binding of the master Rep [10,24]. The CR-M is thought to be involved in regulation of transcription [10] and also contains primer-binding sites for complementary strand DNA synthesis [25].

BBTD was first recorded in 1889 in Fiji and then found throughout Asia, Oceania and Africa [1,26]. Sequenced BBTV isolates are classified into two phylogenetic groups with a distinct geographical delineation: the Pacific and Indian Oceans (PIO) group (Australia, Africa, India, Sri Lanka, Tonga, Hawaii) and the South-East Asia (SEA) group (Vietnam, Philippines, Indonesia, Taiwan, China, Japan) [26–28]. SEA is a diversity hotspot and most likely origin of BBTV [26] from where the virus was exported to other regions of the world, following banana domestication between 7,000 and 10,000 years ago [29,30], via long-distance movements of virus-infected propagules. The first of such movements was from SEA to India approximately 1,000 years ago and the Indian subcontinent was in turn an origin of two movement events to Sub-Saharan Africa between 1825 and 1934 [26]. The SEA isolates of BBTV are frequently associated with alphasatellites [31–39]. Alphasatellites (family *Alphasatellitidae*) have a circular ssDNA genome encoding a Rep protein that enables autonomous replication of alphasatellite DNA but cannot transreplicate BBTV components [17]. Similar to other plant ssDNA virus-associated satellites (betasatellites and deltasatellites; family *Tolecusatellitidae*), alphasatellites depend on their helper virus for movement, encapsidation and plant-to-plant transmission [40]. Alphasatellites are associated with helper viruses of the *Babuvirus* and *Nanovirus* genera of family *Nanoviridae*, the *Cofodevirus* genus of *Metaxiviridae*, and the *Begomovirus* and *Mastrevirus* genera of *Geminiviridae* and are classified into subfamilies *Nanoalphasatellitinae*, *Petromoalphasatellitinae* and *Geminialphasatellitinae* [41,42]. *Nanoalphasatellitinae* and *Petromoalphasatellitinae* alphasatellites have a size of ca. 1.1 Kb (in some genera, ca. 1.3 Kb), similar to a component size of their helper nano-, cofode- and babuviruses, whereas *Geminialphasatellitinae* ones have a size of ca. 1.3 Kb, approximately a half-size of their helper geminivirids. Notably, *Petromoalphasatellitinae* alphasatellites are associated with BBTV, cardamom bushy stunt virus (another member of the genus *Babuvirus*) and coconut foliar decay virus (genus *Cofodevirus*) which all infect monocots, while *Nanoalphasatellitinae* alphasatellites are associated with helper viruses of the genus *Nanovirus* which infect dicots.

Impacts of alphasatellites on BBTV infection and disease transmission by aphids have not been studied so far. In the case of related nanovirids, an alphasatellite associated with faba bean necrotic yellows virus (genus *Nanovirus*) had a negative impact on helper virus infectivity (reducing the number of infected plants following agroinoculation with viral infectious clones) and reduced disease severity [43]. Another study in the same pathosystem, however, revealed no effect of the alphasatellite on disease severity and demonstrated that the transmission rate of the helper virus by aphids was increased in the presence of alphasatellite, despite a substantial reduction of helper virus DNA loads in aphids [44]. Alphasatellites associated with geminivirids were also reported to affect helper virus accumulation and transmission as well as disease symptom development [45–50]. Thus, alphasatellites associated with bipartite begomoviruses (genus *Begomovirus*) increased symptom severity, affected helper virus accumulation and interfered with virus transmission by the whitefly *Bemisia tabaci* [49,50]. Interestingly, an alphasatellite associated with a monopartite geminivirus of the genus *Mastrevirus* enhanced disease severity in monocot wheat plants, which coincided with increased accumulation of helper virus DNA and decreased accumulation of helper virus-derived small interfering (si)RNAs [48]. However, an alphasatellite of a monopartite geminivirus of the genus *Begomovirus* did not affect siRNA production from its helper virus or betasatellite in *N*. *benthamiana* [51]. In the latter study, alphasatellite-derived siRNAs were also detected by blot hybridization, indicating that both helper virus and alphasatellite are targeted by the plant antiviral defense based on RNA interference (RNAi) [51].

The RNAi machinery has evolved in most eukaryotes to regulate endogenous gene expression and to defend against invasive nucleic acids such as viruses, satellites, viroids, transposons and transgenes [52]. The plant RNAi pathways are mediated by small (s)RNAs classified into siRNAs and micro (mi)RNAs, which are generated by Dicer-like (DCL) family proteins from double-stranded (ds)RNA precursors and are predominantly 21, 22 and 24 nucleotide (nt) long. All sRNA types and size classes bind Argonaute (AGO) family proteins and guide the resulting silencing complexes to repress target gene expression post-transcriptionally and/or transcriptionally in a sequence-specific manner. RNA-directed DNA methylation (RdDM) is one of the plant RNAi pathways operating in the nucleus to establish and maintain transcriptionally-silent chromatin via *de novo* cytosine methylation directed by 24 nt siRNAs [53,54]. DNA virus-infected plants accumulate 24 nt viral siRNAs that can potentially silence viral DNA transcription via RdDM as well as 21 and 22 nt viral siRNAs that can potentially silence viral mRNAs post-transcriptionally through their cleavage and degradation or translational repression [55–59]. Yet, DNA viruses of the families *Geminiviridae* and *Caulimoviridae* (reverse-transcribing dsDNA viruses) can evade and/or suppress RNAi (reviewed in [60] and [52]). Little is known about interactions of nanovirids with the plant RNAi machinery. For BBTV, MP and CP of a SEA isolate and Clink and MP of a PIO isolate were reported to suppress transgene silencing in *N*. *benthamiana* when expressed from an RNA virus vector [61,62]. Mechanisms of RNAi evasion by nanovirids can be similar to those proposed for geminivirids [60] due to resemblance in their replication mechanisms [11]. Interestingly, Rep proteins of begomoviral alphasatellites suppressed post-transcriptional transgene silencing in *N*. *benthamiana* [46] and, when expressed from an RNA virus vector, could restore expression of a transcriptionally silenced transgene in *N*. *benthamiana* [63].

Here we describe the identification and molecular and biological characterization of a first alphasatellite associated with BBTV isolates from the PIO phylogenetic group. It was identified in Democratic Republic of the Congo (DRC), one of the first countries in Sub-Saharan Africa where BBTD was introduced and first reported in Kisangani region of Tshopo province in 1958 [28,64]. This alphasatellite represents a new genus of the family *Alphasatellitidae*, related to several genera of the subfamily *Nanoalphasatellitinae*. We demonstrate that BBTV and alphasatellite clones can infect the dicot *N*. *benthamiana*, followed by recovery and symptomless persistence of the alphasatellite. We also show that the alphasatellite reduces BBTV accumulation in banana plants and aphids and interferes with virus transmission by aphids. By transcriptome and sRNA-ome sequencing we uncovered new features of Pol II transcription of viral mRNAs and biogenesis of viral siRNAs, both affected by the alphasatellite.

## Results and discussion

### Discovery and molecular characterization of the first alphasatellite associated with BBTV in Africa

Banana aphids (*P*. *nigronervosa*) collected in December 2016 on a bunchy top diseased banana plantain (genotype AAB) in the Boko village of Congo-Central province of DRC were placed on healthy seedlings of *Musa acuminata* Cavendish (genotype AAA) for disease transmission under laboratory conditions (see Materials and Methods). All the recipient plants eventually developed characteristic BBTD symptoms (**S1A Fig**), while no infection was obtained with aphids collected on a symptomless banana plant in the same field at the same time. PCR and immuno-capture (IC) PCR analyses with DNA-R specific primers confirmed the presence of BBTV in both the viruliferous aphids and the infected plants, respectively. One of the infected plants (numbered p4.3) which had been the first to exhibit disease symptoms was then used as a source plant for BBTD transmission tests. Both the viruliferous DRC aphids born on the source plant and the virus-free aphids from DRC and Gabon (GAB aphids) fed for 24 hrs on a detached leaf of the source plant transmitted the disease to recipient plants (**S1B** and **S1C Fig**) with a latency period of 3 to 9 weeks. A single DRC aphid placed on a recipient plant leaf was also able to transmit the virus (**S1D** and **S1E Fig**).

We then undertook *de novo* reconstruction of a complete viral genome from the infected plants and aphids using rolling circle amplification (RCA) of viral DNA followed by Illumina sequencing. RCA products were verified by digestion with AvaI (**S1G** and **S1H Fig**), the enzyme with a conserved restriction site in the BBTV CR-SL of previously characterized isolates from DRC [26,65]. Based on AvaI restriction analysis, the following 11 samples were selected for Illumina sequencing of undigested RCA products: (i) two leaf samples from the source plant p4.3 taken at two time points (one month apart), along with two pools of DRC aphids (8–10 adults) collected from this plant at the respective time points, (ii) two leaf samples taken from the recipient plants following disease transmission by DRC and GAB aphids, along with two pools of respective aphids (8–10 adults), and (iii) single DRC aphids from three recipient plants (**S2 Fig**). The Illumina reads were *de novo* assembled into contigs and their consensus sequences were verified as described in Material and Methods. BLASTn analysis of the resulting contigs revealed that they represent terminally-redundant sequences of the six circular components of the BBTV genome in all 11 samples. The reconstructed BBTV components from the source plant p4.3 shared high sequence similarity to respective components of other BBTV isolates from DRC, such as Mbk-24 collected in 2012 [65]: 99.0% pairwise identity in DNA-C (KU759879), 99.4% in DNA-M (KU759878); 99.5% in DNA-N (KU759880), 99.1% in DNA-R (KU687050), 99.5% in DNA-S (KU759877) and 98.9% in DNA-U3 (KU759869). Single nucleotide polymorphism (SNP) analysis revealed that at both time points the source plant p4.3 and all the aphid samples from this plant (including the single aphid) contained a single variant for DNAs C, N and R and two variants (v1 and v2) for DNAs M, S and U3, which differed by 3, 6 and 2 SNPs, respectively (**S1A Dataset**). In all cases the less abundant variant v2 was supported by substantial and comparable proportions of reads in all the plant and aphid samples (**S3 Fig**), indicating the stability of BBTV quasispecies population in the plant and its viruliferous aphids’ colony over one month period between the sampling time-points. However, BBTV quasispecies had slightly evolved upon transmission to new plants. Following DRC aphid transmission, the recipient plant and the aphids’ progeny contained only one of the two variants of DNAs M and S (v1 in both cases), while the variant v2 of DNA-U3 became more abundant than the variant v1 (**S3 Fig**). Following GAB aphid transmission, both variants of DNAs M and S were present in the recipient plant and the aphids’ progeny, albeit at altered ratios, while the two variants of DNA-U3 were replaced by a single variant (v3) derived from v2 (8 new SNPs) (**S3 Fig** and **S1A Dataset**).

Besides BBTV, 7 of the 11 sequenced samples (JGF-1, JGF-2, JGF-4, JGF-5, JGF-6, JGF-7, JGF-10) contained terminally-redundant contigs representing a 1.1 Kb genome of an alphasatellite, most closely related to four alphasatellites (AJ005966, AJ132187, MF510474, MF510475) associated with faba bean necrotic yellows virus (genus *Nanovirus*) (BLASTn: 67.4–68.7% pairwise identity within 69–75% query coverage; **S1D Dataset**). All the 7 samples including the leaf and aphid samples from the source plant p4.3 and two samples from recipient plants contained a single variant of this alphasatellite, indicating its genetic stability before and after transmission. Mapping of Illumina reads to the alphasatellite sequence revealed its presence in one of the remaining 4 samples (JGF-8), while 3 others contained very low numbers of alphasatellite reads (**S4 Fig**) that may represent cross-contamination during Illumina sequencing of multiplexed libraries in one flowcell.

To validate the Illumina sequencing results, we amplified by PCR and cloned the alphasatellite and six BBTV components from the aphid pool of the source plant p4.3 at the first sampling time-point (**S2 Fig**, sample JGF-5). Sanger sequencing of the resulting full-length clones confirmed the *de novo* assembled viral sequences and the presence of two variants (v1 and v2) for DNAs M, S and U3. We then designed specific diagnostic primers for each BBTV component and alphasatellite and performed single, duplex and multiplex PCR analysis of all the 11 samples, which confirmed the presence of respective components in each sample (**S5A** and **S5B Fig**). Consistent with Illumina read counts (**S4 Fig**), semi-quantitative multiplex PCR confirmed that alphasatellite is one of the most abundant virome components in the source plant p4.3 and its aphid colony and that relative abundance of BBTV components is altered when alphasatellite loads are reduced (**S5B Fig**).

To evaluate prevalence of the newly discovered alphasatellite in DRC and other countries of Sub-Saharan Africa, we performed PCR screening of BBTV-infected banana leaf samples collected in DRC in 2012 (n = 401) [64,65], Gabon in 2016 (n = 6), Nigeria in 2019 (n = 16), Togo in 2019 (n = 5) and Benin in 2018–2020 (n = 33). One of the samples from DRC (AAB plantain, Kimpoko village, Congo-Central province) was found to be PCR-positive for alphasatellite, whereas all other samples scored either PCR-negative, or yielded very low abundance or diffuse products (**S6 Fig**). Furthermore, BBTV-infected banana plants originating from Malawi and New Caledonia (maintained in CIRAD greenhouse, Montpellier) were also found to be alphasatellite-negative. IC-PCR analysis of the leaf tissues from selected DRC-2012 samples confirmed the presence of alphasatellite in the PCR-positive sample and demonstrated encapsidation of its DNA by BBTV coat protein, similar to DNA of DRC-2016 alphasatellite in BBTV-infected Cavendish (**S6A Fig**). Illumina sequencing of RCA-amplified viral DNA of selected samples from DRC (n = 5), Gabon (n = 3), Benin (n = 1), Malawi (n = 1) and New Caledonia (n = 1) confirmed the presence of alphasatellite in the PCR-positive DRC-2012 sample, while other samples did not contain any alphasatellite. A *de novo* assembled sequence of the DRC-2012 alphasatellite shared 98.1% identity with the sequence of DRC-2016 alphasatellite: the differences include 1-, 6- and 10-nt indels in the non-coding sequence and 18 SNPs, 3 of which resulting in 3 amino-acid substitutions in the Rep protein (**S1C Dataset**). The BBTV components of DRC-2012 and DRC-2016 isolates (**S1A** and **S1B Dataset**) were also found to differ: 98.8% identity in DNA-C, 99.3% in DNA-M, 99.3% in DNA-N, 99.5% in DNA-R, 99.5% in DNA-S, and 97.9% in DNA-U3. These findings indicate that the alphasatellite got associated with BBTV in DRC before its sampling in 2012 and then persisted in this country and evolved together with the helper virus in the following years, most likely via aphid transmission as we demonstrated under the laboratory conditions. The two villages in DRC where the alphasatellite was found are located ca. 100 km away from each other. So far, we obtained no evidence for this alphasatellite to spread in other countries, suggesting its recent emergence, which is also supported by its extremely low prevalence in the samples collected in DRC in 2012. Further surveys in DRC and elsewhere in the BBTV-affected areas are required to assess alphasatellite incidence and prevalence in BBTV-infected bananas in Sub-Saharan Africa.

### Sequence comparison and phylogenetic analysis of the DRC alphasatellite

**Fig 1:**
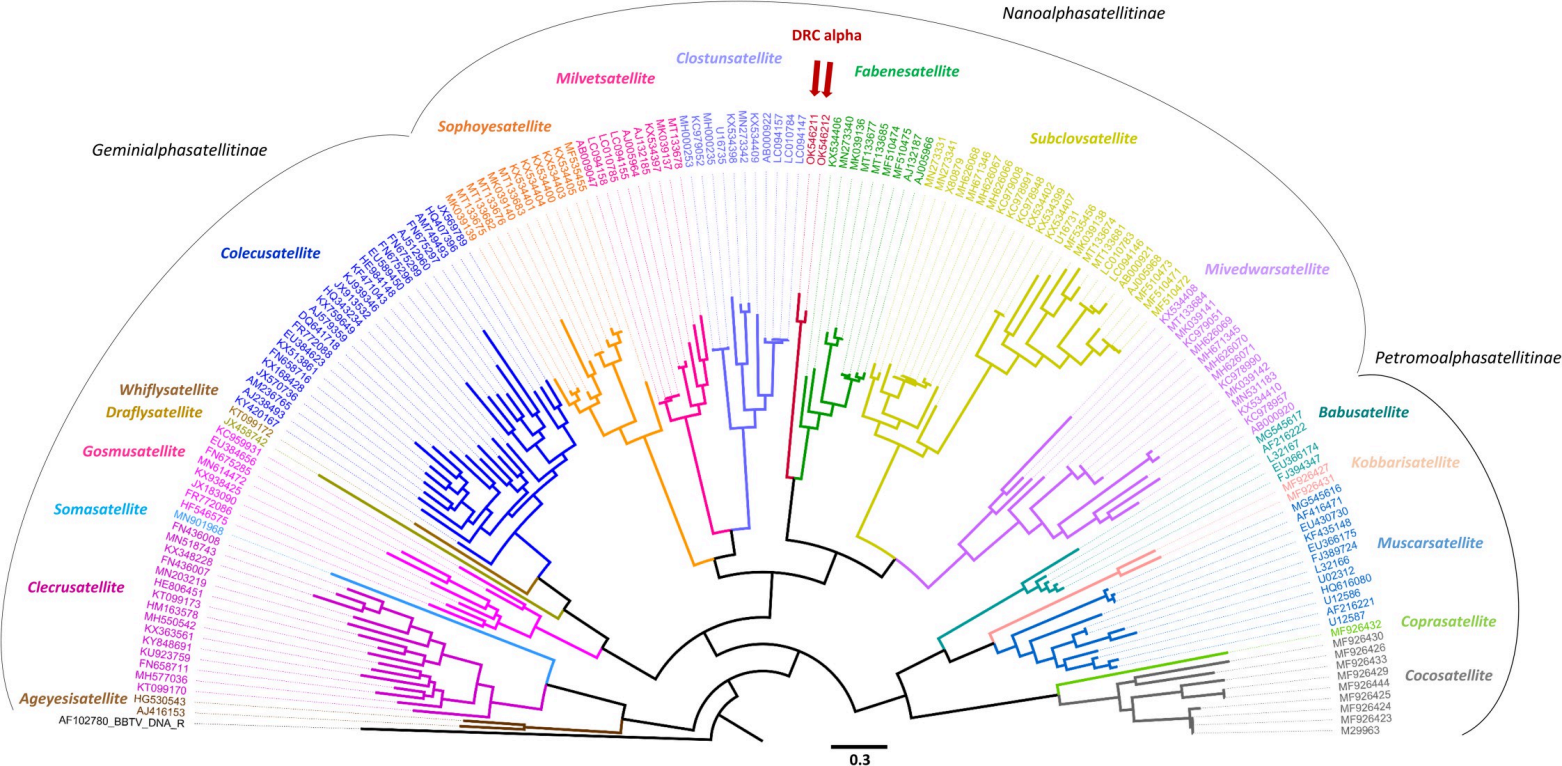
Phylogenetic analysis of DRC alphasatellite. A maximum likelihood phylogenetic tree of complete nucleotide sequences of the two isolates of DRC alphasatellite and all isolates of other alphasatellites associated with helper viruses of the families *Nanoviridae*, *Metaxiviridae* and *Geminiviridae* is rooted with BBTV DNA-R. The Genbank accession number is given for each alphasatellite. Alphasatellites’ genera are color-coded and their subfamilies delineated. Position of the DRC alphasatellite’s genus (*Banaphisatellite*) is indicated with red arrow. Note that the subfamily *Petromoalphasatellitinae* comprises all previously-identified BBTV alphasatellites representing 3 species, namely, BBTA1 (L32167, [31,32]; AF216222, [35]; FJ394347, [37]; MG545617, [39]; EU366174, Genbank only), BBTA2 (AF416471, [34]; EU430730, [36]; MG545616, [39]) and BBTA3 (L32166, [31,32]; U02312, [33]; AF216221, [35]; HQ616080, [38]; FJ389724, [37]; EU366175, U12586 and U12587, Genbank only), and alphasatellites of cardamom bushy dwarf virus (CBDA: KF435148; [72]) and coconut foliar decay virus (CFDA1: M29963, MF926423, MF926424, MF926425; CFDA2: MF926426; CFDA3: MF926427, MF926431; CFDA4: MF926429, MF926444, MF926433; CFDA5: MF926430; CFDA7: MF926432; [89]), which belong to the genera *Babusatellite* (BBTA1), *Muscarsatellite* (BBTA2, BBTA3, CBDA), *Cocosatellite* (CFDA1, CFDA2, CFDA4, CFDA5), *Coprasatellite* (CFDA7) and *Kobbarisatellite* (CFDA3). The genus *Fabenesatellite* of subfamily *Nanoalphasatellitinae*, the closest to BBTV DRC allphasatellite’s genus, comprises variants of faba been necrotic yellows alphasatellite 2 associated with faba bean necrotic yellows virus (AJ005966, AJ132187, MF510474, MF510475; [66,68]), sophora alopecuroides yellow stunt virus (KX534406; [67]), milk vetch chlorotic dwarf virus (MN273330 = MN273340; [70]) and parsley severe stunt-associated virus (MK039136, MT133677, MT133685; [69,71]).

Pairwise sequence comparisons (**S7 Fig**) and phylogenetic analysis (**Fig 1**) of all alphasatellite sequences available in GenBank in September 2021 plus the DRC-2012 and DRC-2016 isolates revealed that, based on current species and genus demarcation criteria (81% and 68% sequence identity, respectively) the DRC alphasatellites represent a novel species that belongs to a new genus falling deeply within the subfamily *Nanoalphasatellitinae* rather than *Petromoalphasatellitinae* (**Fig 1**). The DRC alphasatellite isolates share only 56.7 to 62.8% identity with BBTV SEA-associated alphasatellites and other alphasatellites of the subfamily *Petromoalphasatellitinae* (**S7A Fig**), and share higher identities (64.0 to 67.7%) with isolates of faba bean necrotic yellows alphasatellite 2 (FBNYA2) of the subfamily *Nanoalphasatellitinae* (**S7B Fig**). FBNYA2 is the only member of genus *Fabenesatellite* and is associated with four different viruses of the genus *Nanovirus* which infect dicots [66–71].

Sequence similarity-based clustering analysis of alphasatellite Rep proteins showed (**S8 Fig**) that the DRC alphasatellite is connected to the alphasatellites from *Fabenesatellite*, *Mivedwarsatellite*, *Subclovsatellite* and *Clostunsatellite* genera of the subfamily *Nanoalphasatellitinae*, all associated with the nanoviruses infecting dicot Fabaceae hosts. It is also connected to the alphasatellites from the genus *Gosmusatellite* of *Geminialphasatellitinae*, which are associated with the geminivirids infecting dicot Malvaceae and Asteraceae hosts. In contrast, the DRC alphasatellite is not directly connected to any previously identified alphasatellites of the *Babusatellite* and *Muscarsatellite* genera infecting monocot Musaceae and Zingiberaceae hosts in complexes with their helper babuviruses BBTV [banana bunchy top alphasatellite 1 (BBTA1), BBTA2 and BBTA3; [31–39] and cardamom bushy dwarf virus [72] (**S8 Fig**).

Taken together, two DRC alphasatellite isolates, which we name here banana bunchy top alphasatellite 4 (BBTA4) isolate DRC-2016 and BBTA4 isolate DRC-2012, belong to a novel species representing a new genus in the subfamily *Nanoalphasatellitinae* and likely originating from a dicot plant. We propose to name this new genus “*Banaphisatellite*” (banana aphid satellite), which would highlight discovery of BBTA4 in both banana and banana aphids and its ability to be transmitted by the aphids as well as to distinguish it from the genus *Babusatellite* of subfamily *Petromoalphasatellitinae* which includes the first alphasatellite species (BBTA1) found to be associated with BBTV in SEA [31,32]. To conform a binominal nomenclature recently adopted by the ICTV [73] we propose to name this new alphasatellite species *Banaphisatellite musae1*.

### BBTV and DRC alphasatellite can establish systemic infection in the dicot plant *Nicotiana benthamiana*

**Fig 2:**
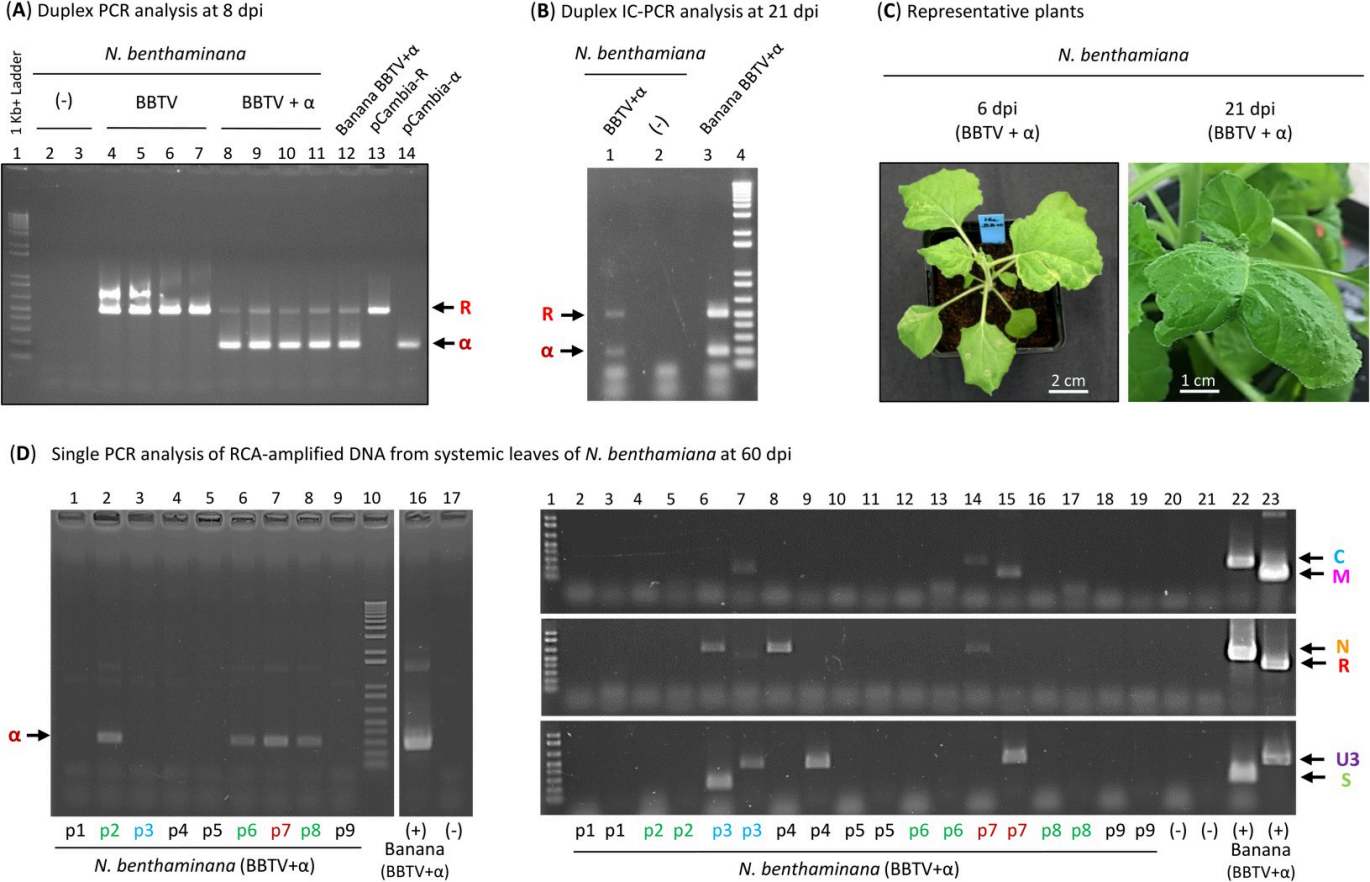
PCR and IC-PCR analysis of *Nicotiana benthamiana* plants agroinoculated with BBTV and alphasatellite clones. (**A**) Duplex PCR analysis with BBTV DNA-R and DRC alphasatellite (α) specific primers of total DNA from symptomatic leaves of *N*. *benthamiana* agroinoculated with six BBTV components (lanes 3–6), six BBTV components and alphasatellite (lanes 7–10) or empty vector (lanes 1–2) at 8 days post-inoculation (dpi). Total DNA from Cavendish banana co-infected with BBTV and DRC alphasatellite and plasmid DNA of BBTV DNA-R and alphasatellite constructs (pCambia-R and pCambia-α) were used as positive controls. Positions of DNA-R and alphasatellite PCR products are indicated by arrows. (**B**) Duplex IC-PCR analysis of systemic leaf tissues of *N*. *benthamiana* agroinoculated with six BBTV components and alphasatellite at 21 dpi (lane 1), mock inoculated *N*. *benthamiana* (lane 2) and Cavendish banana infected with BBTV and alphasatellite following aphid inoculation (lane 3). (**C**) Representative *N*. *benthamiana* plants agroinoculated with 6 BBTV components and alphasatellite at 6 and 21 dpi. Note that wild type and 16c lines of *N*. *benthamiana* exhibited similar and reproducible symptoms in two independent experiments. (**D**) Single PCR analysis with BBTV component (C, M, N, R, S, U3)- and alphasatellite-specific primers of RCA-amplified total DNA from systemic leaves of nine *N*. *benthamiana* plants (p1, p2, p3, p4, p5, p6, p7, p6, p9) agroinoculated with six BBTV components and alphasatellite at 60 dpi. Positions of PCR products for each BBTV component and alphasatellite are indicated by arrows. Note that the original gel image (available on request) was spliced to remove five lanes between lanes 10 and 16.

To test the hypothesis on the dicot plant origin of DRC alphasatellite we constructed infectious clones of the alphasatellite and its helper BBTV. To this end the monomeric clones of six BBTV components (DNA-C, OK546213; DNA-M_v1, OK546214; DNA-N, OK546215; DNA-R, OK546216; DNA-S_v1, OK546217; DNA-U3_v1, OK546218) and alphasatellite (OK546211) from the DRC-2016 aphids were used for construction of partial or, in the case of DNA-R, complete dimer clones (**S9A** and **S9B Fig**). The resulting constructs were mobilized to *Agrobacterium tumefaciens* for agro-inoculation (via leaf infiltration) of *N*. *benthamiana* seedlings. Six days post-inoculation (dpi), plants agroinoculated with the BBTV +/- alphasatellite constructs started to develop disease symptoms including chlorosis and downward curling of systemic leaves (**Fig 2C**). Semi-quantitative duplex PCR analysis of total DNA extracted from the symptomatic leaves at 8 dpi confirmed accumulation of BBTV DNA-R and also alphasatellite DNA in the case of its co-inoculation. Alphasatellite DNA accumulated at higher levels than DNA-R (**Fig 2A**), which is consistent with our findings in banana plants (**S5B Fig**), suggesting that alphasatellite is capable of more efficient self-replication than BBTV DNA-R. In line with this hypothesis, an alphasatellite Rep was ~10 times more active than a master Rep of its helper nanovirus in a replication origin cleavage and nucleotidyl-transfer reaction *in vitro* [66]. Comparative analysis of systemically-infected leaves of agroinoculated *N*. *benthamiana* (at 21 dpi) and aphid-inoculated *M*. *acuminata* Cavendish using IC-PCR (immuno-capture of viral particles, followed by DNase treatment and duplex PCR analysis of remaining encapsidated DNA) revealed that both DNA-R and alphasatellite DNA are encapsidated by BBTV coat protein in both plant species (**Fig 2B**).

Two months post-inoculation all the infected *N*. *benthamiana* plants appeared to recover from infection as newly growing leaves and eventually flowering organs did not exhibit any clear disease symptoms. These findings of systemic infection followed by recovery were reproduced in two independent agroinfiltration experiments with BBTV +/- alphasatellite constructs. PCR analysis of RCA-amplified total DNA extracted from upper leaves of 9 plants co-inoculated with BBTV and alphasatellite and recovered from disease symptoms, revealed that, in 6 plants, all BBTV components were below detection, while in the remaining 3 plants, five, four or two of the six components were readily detectable (**Fig 2D**; plants p3, p7 and p4, respectively). Thus, the recovery coincided with diminished accumulation of one or more components of BBTV genome. Remarkably, alphasatellite DNA was detected in four of the nine plants, three of which being PCR negative for any component of the helper virus (**Fig 2D**; plants p2, p6 and p8). This indicates that DRC alphasatellite can persist in asymptomatic leaf tissues even in the absence of helper virus, likely owing to its ability to self-replicate.

### Transient expression of BBTV Rep, but not alphasatellite Rep, induces chlorosis in *N*. *benthamiana*

To test whether any of the viral proteins can induce and/or suppress antiviral responses in *N*. *benthamiana*, we subcloned all the viral ORFs from the DRC-2016 alphasatellite and BBTV genome sequences under the control of CaMV 35S promoter and terminator for transient expression and silencing suppression assays in *N*. *benthamiana* 16c line transgenic for green fluorescence protein (GFP) (**S10A** and **S10B Fig**). The resulting constructs were co-expressed together with the GFP silencing inducer construct in *N*. *benthamiana* 16c leaves via agroinfiltration. Potyviral protein HC-Pro and bacterial β-glucuronidase (GUS) expression constructs were used as respectively positive and negative controls for silencing suppression. At 8 dpi, only HC-Pro strongly suppressed silencing, resulting in prominent GFP fluorescence under UV light, whereas none of the six BBTV protein or alphasatellite Rep expression constructs exhibited any strong silencing suppression activity (**S10C Fig**). Inspection of infiltrated leaves under day light revealed that the BBTV Rep construct, but not any other construct, induced strong chlorosis of the infiltrated tissues (**S10D Fig**). We hypothesize that, similar to other viruses, BBTV infection may induce both RNA silencing- and innate immunity-based defense responses (reviewed in [74]) resulting in plant recovery from viral infection, while DRC alphasatellite persistence following recovery can be explained by its ability to evade defense responses in *N*. *benthamiana*. The mechanism of defense evasion remains to be investigated.

### DRC alphasatellite interferes with BBTV transmission by banana aphids

To determine if DRC-2016 alphasatellite has any impact on BBTV transmission by aphids and/or development of BBTD symptoms, Cavendish banana plants infected with BBTV alone or co-infected with alphasatellite were taken for two consecutive transmission experiments designated C (4 BBTV vs 3 BBTV+alphasatellite plants) and D (4 BBTV vs 4 BBTV+alphasatellite plants). In each experiment, virus-free DRC aphids were placed on these source plants for three weeks to build colonies. Adult aphids were then taken from each source plant to inoculate recipient plants (5 aphids per recipient plant). After 4 days of inoculation, all aphids were collected from each recipient plant in pools for PCR diagnostics of DNA-R and alphasatellite and quantitative (q)PCR analysis of viral loads. All the aphid pools analyzed by PCR contained the virus and the virus with alphasatellite when it was present in the source plant (**S2 Dataset**). Symptom development was monitored during 80 days to record the dates of appearance of dark-green streaks (Morse code) on leaf midrib and veins (designated as first symptoms) and emergence of next leaf with systemic symptoms (a smaller leaf with shorter petiole, yellow margins and Morse code).

After 80 days, upper leaf samples from symptomatic recipient plants were taken for PCR diagnostics of DNA-R and alphasatellite and qPCR analysis of viral loads. In the case of source plants infected with BBTV alone, all the examined symptomatic recipient plants (n = 55) were found to be PCR-positive for BBTV. In the case of BBTV- and alphasatellite-coinfected source plants, all the examined symptomatic recipient plants (n = 28) were found to be PCR-positive for BBTV and most of them (n = 20) were also PCR positive for alphasatellite. Since all the 28 aphid pools contained alphasatellite DNA together with six BBTV components (**S2 Dataset**), alphasatellite could be lost during aphid inoculation or development of systemic infection in some of the recipient plants.

**Fig 3:**
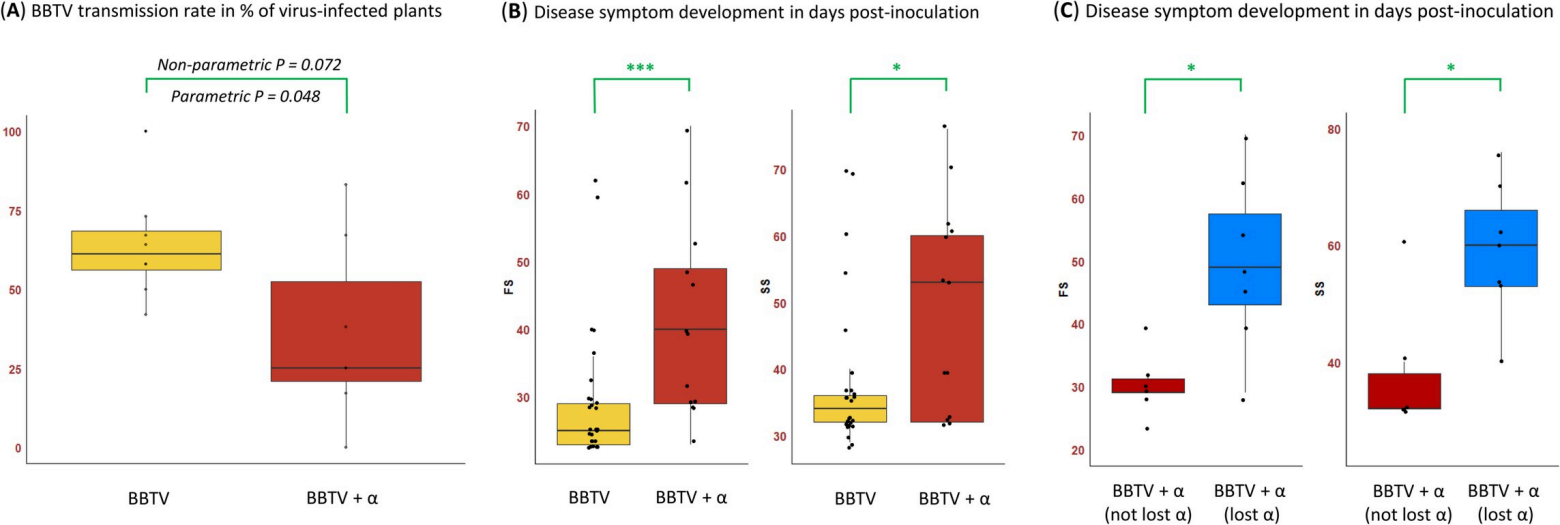
Impact of DRC alphasatellite on BBTV transmission by banana aphids and disease symptom development in recipient plants. (**A**) Transmission rates in percentage of virus-infected recipient plants per source plant infected with BBTV alone (n = 8) and co-infected with BBTV and alphasatellite (n = 7) are shown for two transmission experiments (C and D). Statistical significance of differences in the transmission rates with and without alphasatellite was calculated using in a parametric linear model test and a non-parametric Kruskal-Wallis and the resulting P-values are indicated. (**B**) Delay in development of first (FS) and systemic (SS) symptoms in recipient plants in days post-inoculation with BBTV alone (n = 26) and BBTV+alphasatellite (n = 13) in the transmission experiment D. (**C**) Delay in development of first (FS) and systemic (SS) symptoms in the recipient plants of experiment D in days post-inoculation with BBTV+alphasatellite plotted separately for the plants that contained alphasatellite (n = 6) and those that lost alphasatellite (n = 7). * Kruskal-Wallis P < 0.05. *** Kruskal-Wallis P < 0.005.

**Fig 4:**
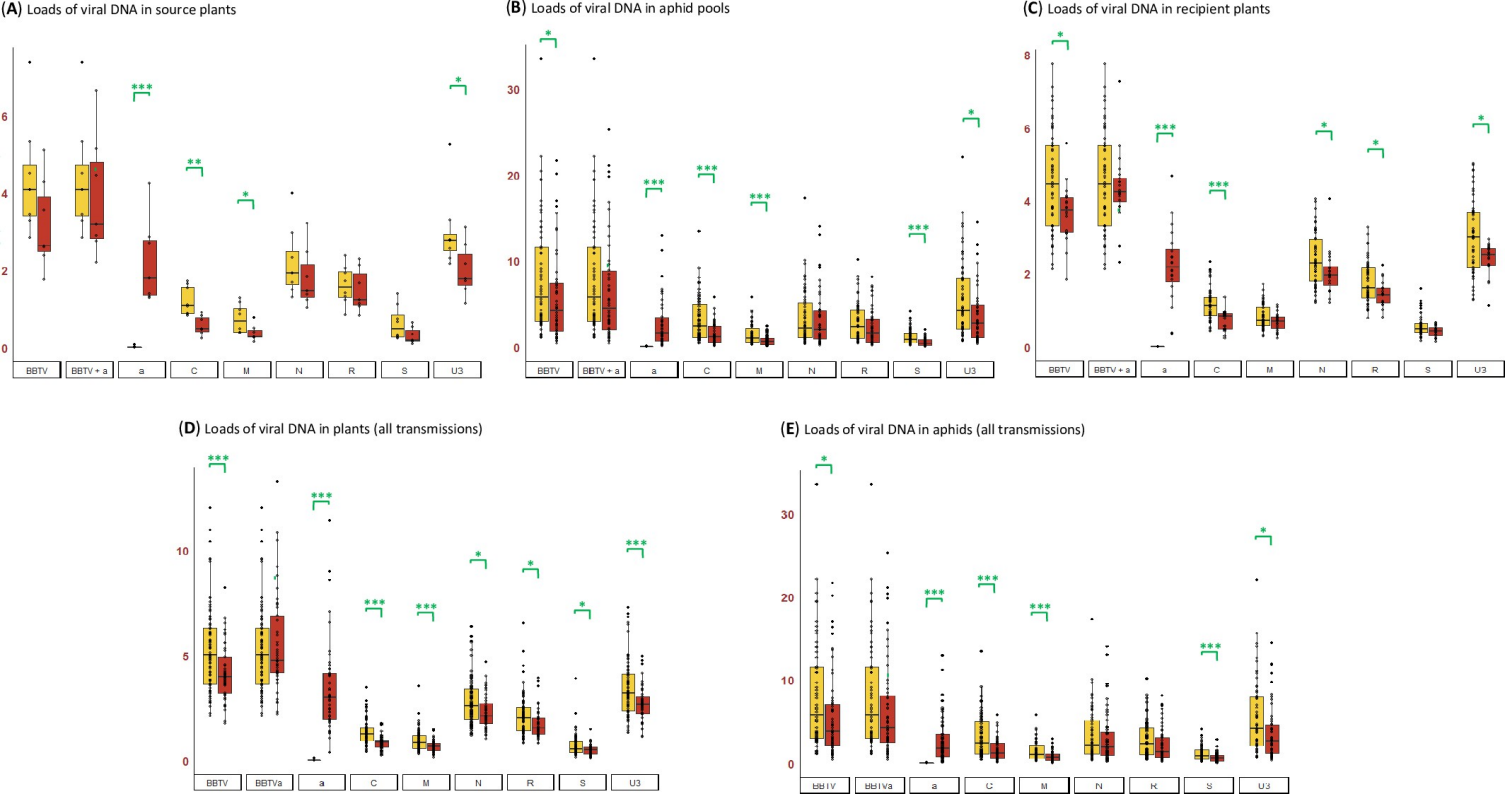
Impact of DRC alphasatellite on viral DNA loads in source plants, aphid pools and recipient plants. Viral DNA loads for each BBTV component (C, M, N, R, S, U3) and alphasatellite (a) as well as for total helper virus without (BBTV) or with (BBTVa) alphasatellite measured by quantitative PCR in (**A**) source plants without (n = 8) and with (n = 7) alphasatellite (transmission experiments C and D), (**B**) aphid pools without (n = 58) and with (n = 46) alphasatellite (experiments C and D), (**C**) recipient plants without (n = 52) and with (n = 20) alphasatellite (experiments C and D), (**D**) all plants without (n = 77) and with (n = 47) alphasatellite (all experiments) and (**E**) all aphid samples without (n = 58) and with (n = 56) alphasatellite (all experiments). * Kruskal-Wallis P < 0.05. *** Kruskal-Wallis P < 0.005.

The results of two transmission experiments showed a negative impact of DRC alphasatellite on the BBTV transmission rate (percentage of virus-infected plants among inoculated recipient plants) which was reduced from 63% to 37% (**Fig 3A**). Using parametric Student and linear model tests, the impact of the alphasatellite was found to be statistically significant (P = 0.041 and P = 0.048, respectively). However, a non-parametric Kruskal-Wallis test yielded a P-value (P = 0.072) exceeding an arbitrary threshold (P ≤ 0.05) of statistical significance. This is due to variation in BBTV transmission rates between the individual, alphasatellite-containing source plants, two of which giving bigger proportions of virus-infected per inoculated recipient plants (10/12 and 8/12) than the other five (2/12, 0/11, 3/12, 3/8, 3/12). In contrast, in the absence of alphasatellite the variation between the individual source plants was smaller (5/12, 7/12, 8/11, 8/12, 7/11, 7/12, 6/12 and 12/12) (**S2 Dataset** and **Fig 3A**). Thus, alphasatellite strongly interfered with BBTV transmission from 5 out of 7 source plants. When comparing individual recipient plants the success of viral infection did not depend on viral DNA loads in the aphid pools after 4-day inoculation (**S2 Dataset**). Nonetheless, the median load of helper virus DNA in the aphids was found to be lower in the presence of alphasatellite than in its absence (Kruskal-Wallis P = 0.045) (**Fig 4B**). Likewise, alphasatellite reduced median loads of helper virus DNA in both source and recipient plants, although only in the recipient plants the reduction was found to be statistically significant (Kruskal-Wallis P = 0.007 vs P = 0.086) (**Fig 4A** and **4C**).

It should be noted that all the examined pools of viruliferous aphids (independent of the transmission success) contained all the six DNA components of BBTV genome. Likewise, all the examined symptomatic recipient plants contained the complete BBTV genome, with an exception for two plants where DNA-N was below detection (**S2 Dataset**). Since these two plants developed disease symptoms, the nuclear shuttle protein (NSP) encoded by DNA-N is not required for systemic infection and symptom development. When the plant lacking DNA-N was taken in a follow-up aphid transmission experiment, none of 12 recipient plants developed any disease symptoms or contained any detectable amounts of viral DNA. This suggests that BBTV NSP may serve as an aphid transmission factor, similar to nanoviral NSP [22,23]. In further support of this hypothesis, a banana plant naturally infected with a mild strain of BBTV in Taiwan lacked DNA-N and banana aphids were not able to transmit this strain to new plants [37].

The effect of DRC alphasatellite on disease symptom development could be properly evaluated only in the experiment D (because the experiment C was disturbed by thrips infestation) and found to be negative: the first symptoms (FS) were delayed for ca. 15 days (Kruskal-Wallis P = 0.005), while the systemic symptoms (SS) for ca. 20 days (Kruskal-Wallis P = 0.032) (**Fig 3B** and **S2 Dataset**). Remarkably, both first and systemic symptoms were delayed much more pronouncedly in the recipient plants that had lost alphasatellite upon inoculation (n = 7) than in those containing the alphasatellite (n = 6) (Kruskal-Wallis P = 0.014 and 0.013, respectively) (**Fig 3C**). Note that in the experiment C, one of the 13 examined recipient plants lacked alphasatellite, but, in that plant, DNA-N was also below detection (**S2 Dataset**). We speculate that the loss of alphasatellite could be due to its competition with helper virus components for viral coat protein within a single co-infected cell and, additionally, a higher probability for six BBTV components to create the disease complex than for the six components to be associated with alphasatellite in single or adjacent cells. It is puzzling, however, why the loss of alphasatellite correlated with the pronounced delay in BBTV symptom development.

### DRC alphasatellite interferes with BBTV replication in plants and reduces BBTV loads both in plants and aphids

To evaluate the impact of DRC alphasatellite on BBTV replication in banana plants and on BBTV acquisition and persistence in aphids we performed qPCR analysis of viral DNA in BBTV-infected Cavendish plants (n = 122) and viruliferous DRC aphids (n = 114) sampled during all our transmission experiments from 2017 to 2021. The loads of each BBTV component (C, M, N, R, S, U3) and alphasatellite (alpha) as well as total viral DNA (C+M+N+R+S+U3+alpha) were measured using a host housekeeping gene (banana RPS2 and aphid EF1α) as an internal control for normalization (**Figs 4D and 4E** and **S11**; note that in these Figures “BBTV” indicates the helper virus DNA, while “BBTVa”—the total viral DNA).

In banana plants, median loads of all BBTV components were reduced in the presence of alphasatellite, thereby resulting in a ~25% decrease of the median load of total helper virus DNA (Kruskal-Wallis P = 0.004). The median loads of total viral DNA (BBTV+alpha) were found to be comparable in the presence and absence of alphasatellite (Kruskal-Wallis P = 0.70) (**Figs 4D** and **S11A**). This suggests that self-replicating alphasatellite competes with the helper virus for the host replication machinery, thereby reducing accumulation of helper virus DNA. Notably, the median load of alphasatellite is the highest among viral components, followed by DNAs U3, N and R, which also accumulate at relatively high levels, compared to DNAs C, M and S. In the absence of alphasatellite, DNA-U3 is the most abundant component followed by DNAs N, R, C, M and S (in that order) (**Fig 4D**). DNA-S encoding the coat protein is the least abundant component at both conditions, which might become a limiting factor for virus encapsidation in the presence of alphasatellite that reduces its accumulation.

In aphids, median loads of all BBTV components were also reduced in the presence of alphasatellite, thereby resulting in a ~40% decrease in the median load of total helper virus DNA (Kruskal-Wallis P = 0.025). The difference in median loads of total viral DNA in the presence and absence of alphasatellite was not statistically significant (Kruskal-Wallis P = 0.12) (**Figs 4E** and **S11B**). While alphasatellite is the most abundant viral component in virus-infected plants, in aphids it becomes the third most abundant after DNAs U3 and N and accumulates at levels comparable to those of DNA-R (**Fig 4**). Since the plant phloem sap ingested by aphids contains encapsidated viral DNA, a large proportion of alphasatellite may not be encapsidated *in planta* due to a limiting amount of the viral coat protein. In support of this hypothesis, our semi-quantitative duplex IC-PCR vs duplex PCR analyses revealed that high-abundance alphasatellite DNA is encapsidated less efficiently than low-abundance DNA-R in both banana and *N*. *benthamiana* plants (**Fig 2B** versus **Figs 2A** and **S5**).

**Fig 5:**
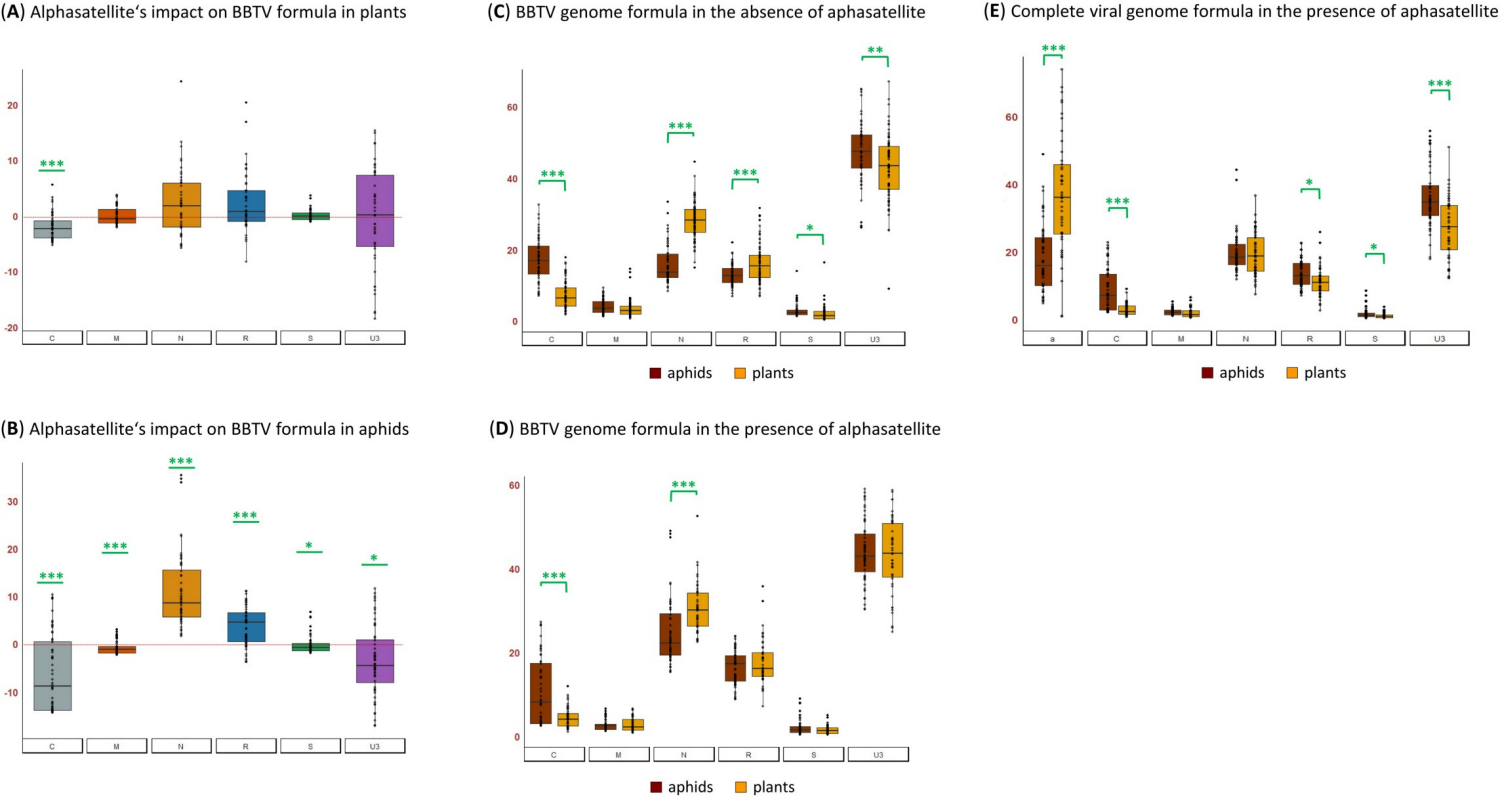
Impact of DRC alphasatellite on BBTV genome formulas in banana plants and aphids. Relative abundance (formula) of BBTV genome components in the presence and absence of alphasatellite was calculated in infected plants and aphids from all transmission experiments (see Fig 4D and 4E). (**A-B**) Alphasatellite-mediated alterations in BBTV genome formula in plants (**A**) and aphids (**B**) plotted for each component as difference in its percentage (above zero when increased and below zero when decreased). (**C-D**) Comparisons between BBTV genome formulas in plants and aphids in the absence (**C**) and presence (**D**) of alphasatellite plotted in percentage of total BBTV DNA. (**E**) Comparison between virome (BBTV + alphasatellite) formulas in the plants and aphids containing alphasatellite plotted in percentage of total viral DNA. * Kruskal-Wallis P < 0.05. *** Kruskal-Wallis P < 0.005.

To further evaluate the impact of DRC alphasatellite on helper virus replication, we analyzed its effect on relative abundance (formula) of BBTV genome components in banana plants. Being the most abundant virome component (on average 35% of total viral DNA; **Fig 5E**), alphasatellite leads to a substantial and statistically-significant decrease in relative abundance of DNA-C (Kruskal-Wallis P = 1.97E-05) but not other components, although median values are increased for DNAs N and R (**Fig 5A**) which together compensate for the more pronounced decrease in relative abundance of DNA-C. Therefore, alphasatellite might interfere more substantially with master Rep-mediated transreplication of DNA-C than other components, which in turn might lead to reduced efficiency of viral replication. Indeed, Clink encoded by DNA-C is a replication enhancer interacting with retinoblastoma-related protein and modulating cell cycle in favor of viral replication [18,19].

In aphids, alphasatellite becomes relatively less abundant (15% of total viral DNA; **Fig 5E**) and has a more pronounced impact on the helper virus formula, leading to a bigger decrease in relative abundance of DNA-C (Kruskal-Wallis P = 2.20E-16) and a more substantial (and statistically-significant) increase in relative abundances of DNAs N and R (Kruskal-Wallis P = 2.20E-16 and P = 0.0002, respectively) (**Fig 5B**). Thus, alphasatellite-mediated alterations in relative abundances of BBTV components *in planta* become more pronounced in aphids.

Further comparison of BBTV genome formulas between plants and aphids revealed that, in the absence of alphasatellite, DNA-N is relatively more abundant in plants, while DNA-C is relatively more abundant in aphids (**Fig 5C**). In the presence of alphasatellite, DNA-N is still relatively more abundant in plants, while DNA-C is still relatively more abundant in aphids, but these differences are less pronounced than in alphasatellite´s absence (**Fig 5D**).

In the presence of alphasatellite, the overall formula of viral DNA components differs between plants and aphids: in plants, relative abundance of alphasatellite DNA is comparable to that of DNA-U3 which is the most abundant component of helper virus, while alphasatellite becomes relatively less abundant than DNA-U3 in aphids and accumulates at levels comparable of those of DNA-N (which is the second most abundant component of the helper virus after DNA-U3 in plants and the third most abundant in aphids) (**Fig 5E**). This indicates that both alphasatellite and DNA-N are less efficiently acquired by aphids compared to other components, possibly due to differences in relative encapsidation of viral DNA. No difference in the relative abundance of DNAs N and M was found between plants and aphids, while relative abundances of DNAs C, R, S and U3 are slightly (and statistically significantly) increased in aphids compared to plants (**Fig 5E**).

Taken together our findings suggest that DRC alphasatellite being the most abundant viral component *in planta* interferes with encapsidation of helper virus components and their acquisition by aphids and/or with their persistence in aphids. In addition to apparent competition with helper virus for the plant replication machinery leading to reduced accumulation of all viral components, DRC alphasatellite has a more pronounced negative impact on accumulation of replication enhancer-encoding DNA-C, which might further reduce helper virus replication efficiency.

### DRC alphasatellite affects BBTV transcription

To further understand molecular mechanisms underlying alphasatellite-mediated alterations in helper virus accumulation, genome formula and transmission rate, we examined the impact of DRC alphasatellite on viral gene expression and antiviral RNAi responses by Illumina sequencing analysis of long and small RNAs from Cavendish plants infected with BBTV alone (n = 3) and co-infected with alphasatellite (n = 3) (**S3** and **S4 Datasets**). The plants were selected based on qPCR analysis of viral DNA loads and formulas to be representative of the analyzed infected plants with respect of both between-sample variation and differences in median values in the presence and absence of alphasatellite. In the selected plants, alphasatellite constituted on average ca. 40% of total viral DNA and the average load of total helper virus DNA was ca. 50% higher in the absence of alphasatellite than in its presence: the negative impact of alphasatellite on helper virus accumulation was distributed between all six DNA components (**S12 Fig**). Such selection enabled us to pool together RNA-seq reads for three plants per each condition (+/–alphasatellite) in order to account for between-plant variation and to quantify an average impact of alphasatellite on helper virus transcription and viral siRNA production.

**Fig 6:**
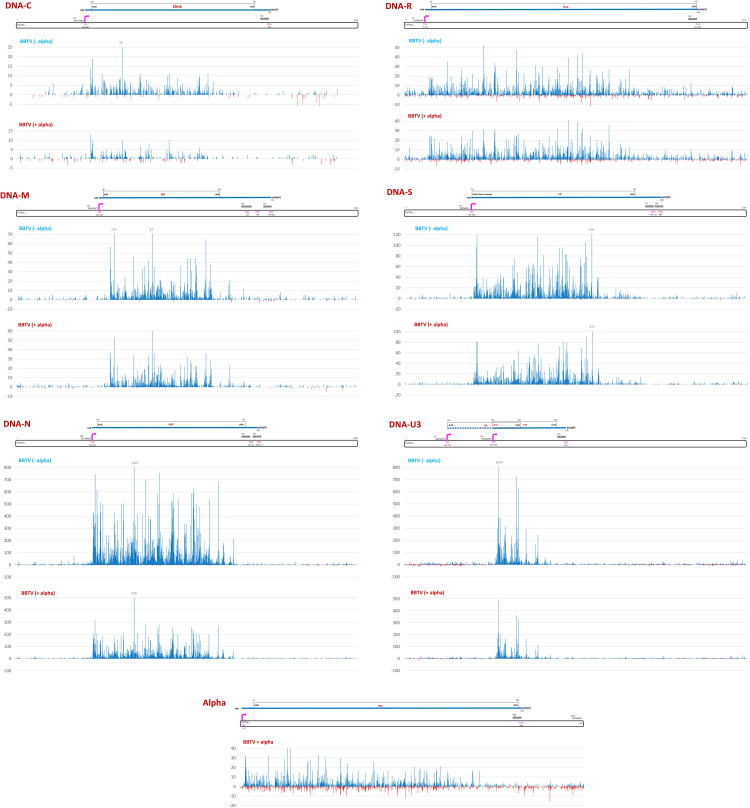
Single nucleotide resolution maps of Illumina mRNA-seq reads representing poly(A)-enriched viral transcripts from BBTV-infected Cavendish banana plants with or without DRC alphasatellite. For each of the two conditions, i.e. without (BBTV-alpha) and with (BBTV+alpha) alphasatellite, Illumina 75 nt paired-end reads of the three biological replicates (leaf tissues of three plants) were combined and mapped simultaneously onto the reference sequences of six BBTV components (-/+ alphasatellite). Histograms plot the numbers of viral 75 nt reads at each nucleotide position of the 1018-to-1111 nt BBTV genome components (DNAs C, M, N, R, S, U3) and 1105 nt alphasatellite (Alpha): blue bars above the axis represent sense reads starting at each respective position, while red bars below represent antisense reads ending at the respective position. The genome organizations of BBTV components and alphasatellite are shown schematically above the respective histograms, with the Pol II promoter (TATA-box and transcription start site, TSS) and terminator (polyA signal, PAS) elements indicated in pink, capped and polyadenylated mRNA shown as solid blue lines, viral protein-coding ORFs boxed and their nucleotide positions given.

Mapping of Illumina 75 nt paired-end mRNA-seq reads onto the viral reference sequences revealed monodirectional Pol II transcription units of viral mRNAs for each BBTV component and alphasatellite (**Figs 6** and **S13** and **S5 Dataset**). With exceptions for DNAs M and U3, these units corresponded to those predicted *in silico* based on positions of (i) TATA or TATA-like boxes at a sufficient distance (>31 nts) upstream of the AUG start codons of viral ORFs and (ii) poly(A) signals (AAUAAA or its variants with one nucleotide substitution) driving pre-mRNA cleavage and polyadenylation downstream of the stop codons. Abundance of the reads representing viral mRNAs was much lower at the predicted 5’-termini and 3’-regions, possibly due to biases in the Illumina mRNA-seq protocol that included poly(A) enrichment, RNA fragmentation, random primer cDNA synthesis and adapter ligation steps. In the case of DNA-S, the second in-frame AUG (A at nt 213) is likely used as an authentic translation initiation codon, as the first AUG (nt 198) is too close to the conserved TATA box (first T at nt 175) and too far from the region of high-abundance reads (starting from nt 216; **S13 Fig**). This finding is consistent with transcription start mapping by 5’-RACE at nt 208 of DNA-S of an Australian BBTV isolate [16] and N-terminal sequencing of its coat protein [21]. In the case of DNA-M, high-abundance reads begin at nt 287, downstream of the AUG start codon located at nt 267 and the 5’-end of mRNA-M previously mapped by 5’-RACE at nt 254 [16]. Thus, the conserved TATA-box at nt 247 appears to drive transcription of the shortened mRNA-M lacking the first AUG, while a TATA-like box at nt 223 may drive transcription of its longer version mapped previously. In the case of DNA-U3, a 5’-half of the previously mapped mRNA, whose transcription could be driven by a TATA-like box at nt 97 [16], was not represented with high-abundance reads (**Figs 6** and **S13**). Sequence inspection upstream of the high-abundance read region (from nt 283) revealed a conserved TATA box at nt 243. However, the only downstream ORF would begin with a non-AUG start codon UUG (nt 272) located too close to the TATA box and in a poor initiation context (UAA UUG CUC) lacking purines at -3 and +4 positions. Analysis of Illumina sRNA-seq reads revealed a highly-abundant 21 nt sRNA belonging to the conserved miR156 family [75] that maps with 2 mismatches to the DNA-U3 positions 272–292 in reverse orientation. This banana miRNA could potentially direct cleavage of mRNA-U3 between nts 281 and 282. For other BBTV mRNAs, high-abundance reads begin upstream of the predicted AUG start codons and at a short distance downstream of the 5´-ends mapped previously [16]: for DNA-C, at nt 220 (vs nt 209); for DNA-N, at nt 246 (vs nt 239); and for DNA-R at nt 83 (vs nt 69 and nt 72) (**Figs 6** and **S13**).

The poly(A) sites that we mapped for our African isolate (**Figs 6** and **S13**) correspond for most but not all viral mRNAs to those previously mapped for the Australian isolate [15]. In DNA-C, a single poly(A) site is located at the same position (nt 753) in both isolates. In DNA-M, poly(A) sites are at nt 710, 738 and 779, with the latter being 2 nts upstream of the previously mapped site. In DNA-N, a major poly(A) site is at nt 749, which corresponds to the previously mapped site, while minor sites are located just upstream (nt 746) and downstream (nt 768 and 772). In DNA-R, poly(A) sites are at nt 947 and 950, with the former corresponding to the previously mapped site. In DNA-S, poly(A) sites are at nt 778, 805 and 808, one of which (nt 778) corresponding to the previously mapped site. In DNA-U3, a single poly(A) site is at nt 502, which is too far upstream of the previously mapped site, making the 3´-UTR much shorter (35 vs 110 nts). It should be noted that, unlike other components, DNA-U3 sequences of the African and Australian isolates differ substantially and the predicted ORFs encode 77 vs 88 amino acid proteins, respectively.

In the case of the alphasatellite transcription unit, high-abundance mRNA-seq reads begin at nt 17, that is 45 nts downstream of a conserved TATA box (nt 1076) and upstream of the AUG start codon of Rep-encoding ORF (nt 41). The alphasatellite poly(A) site is at nt 903, that is at an optimal distance downstream of a conserved poly(A) signal (nt 880) and downstream of the Rep ORF stop codon (nt 892) (**Figs 6** and **S13**).

**Fig 7:**
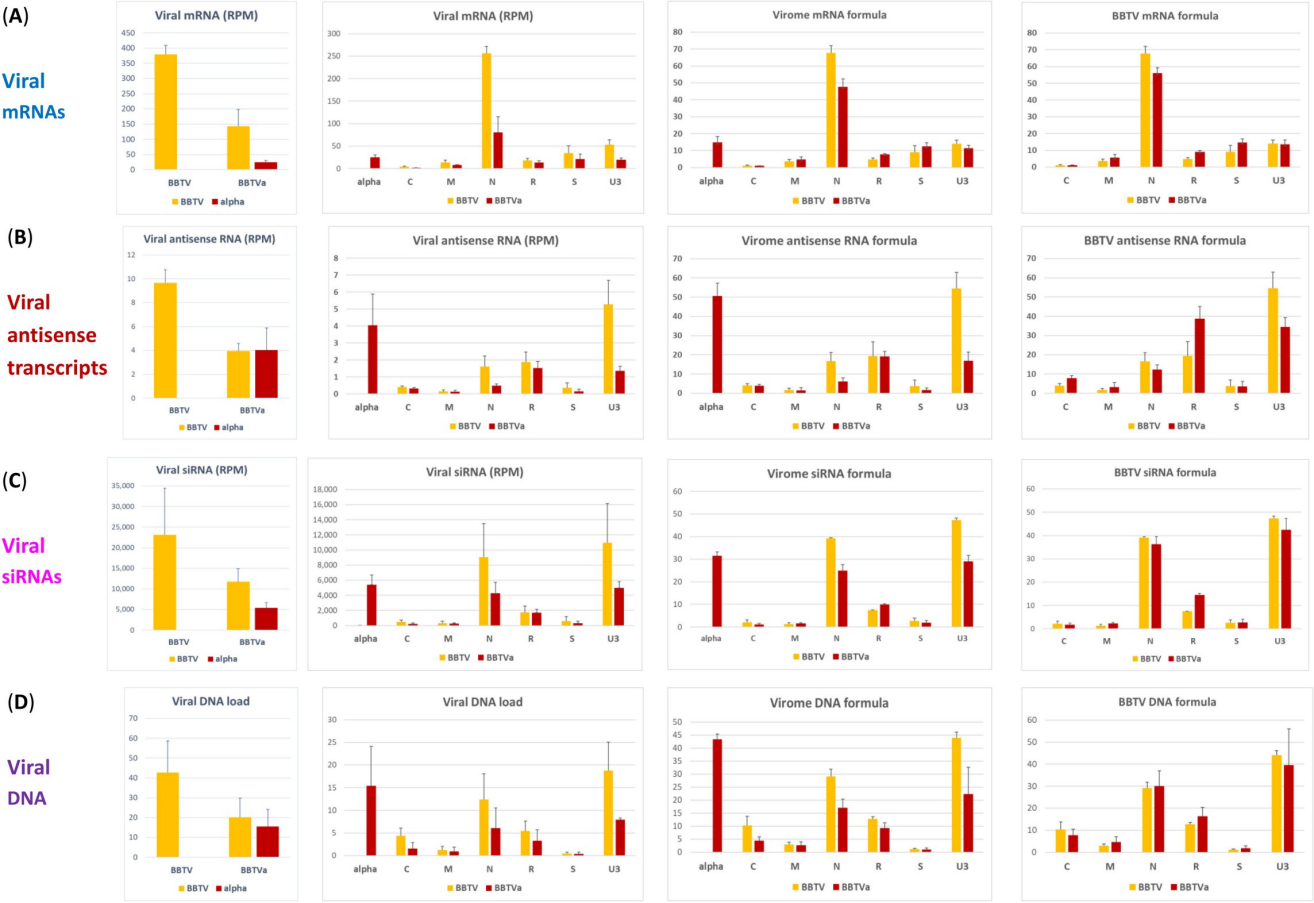
Impact of alphasatellite on accumulation and relative abundance of viral mRNAs, viral antisense transcripts and viral siRNAs. For each of the two conditions, i.e. without (BBTV) and with (BBTVa) alphasatellite, Illumina mRNA-seq 75 nt reads or sRNA-seq 19–25 nt reads of each of the three biological replicates (leaf tissues of three plants) were mapped with zero mismatches onto the reference sequences of all viral mRNA units and all complete viral genome components. Mapped mRNA unit sense reads, complete genome antisense reads, or complete genome sRNA sense+antisense reads were counted in reads per million (RPM) of total reads and their relative abundance (mRNA, antisense transcript, or sRNA formula) was calculated for helper virus (BBTV) and virome (BBTV + alphasatellite) in % of total. (**A-C**) Loads and formulas of viral mRNAs (**A**), viral antisense transcripts (**B**) and viral sRNAs (**C**). (**D**) Loads and formulas of viral DNA calculated by qPCR for each of the three plants (biological replicates) per condition (+/- alphasatellite). Error bars represent standard deviations.

**Fig 8:**
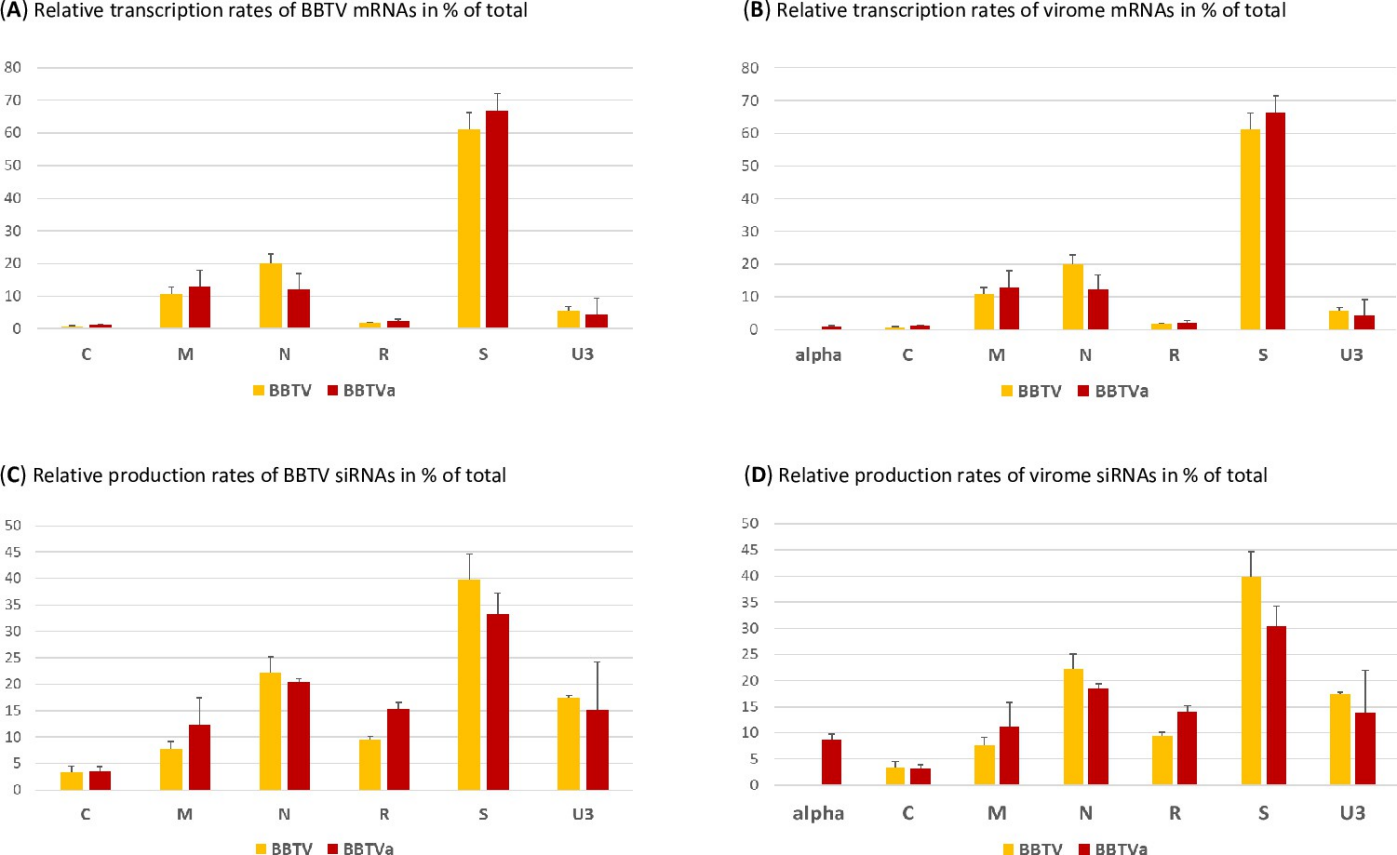
Impact of alphasatellite on relative transcription rates of viral mRNAs and relative production rates of viral siRNAs. Transcription rates of viral mRNAs were calculated by dividing mRNA counts in reads per million by viral DNA loads (measured by qPCR) and by mRNA unit sizes in nucleotides (alphasatellite = 902 nts; C = 549 nts; M = 526 nts; N = 511 nts; R = 883 nts; S = 603 nts; U3 = 231 nts) and the relative transcription rates of viral genome components in the presence (red) and absence (yellow) of alphasatellite (alpha) was plotted in percentage (%) of total BBTV (**A**) and virome (**B**) transcription rates. Production rates of viral 19–25 nt sRNAs were calculated by dividing sRNA counts in reads per million (RPM) by viral DNA loads (measured by qPCR) and the relative sRNA production rates from viral genome components in the presence (red) and absence (yellow) of alphasatellite (alpha) were plotted in % of total BBTV (**C**) and virome (**D**) production rates. Error bars represent standard deviations for three biological replicates.

The presence of alphasatellite did not result in any substantial alteration in positions and relative abundances of the reads representing viral mRNAs (**Figs 6** and **S13**) but had a negative impact on their average accumulation levels (calculated as mRNA unit forward reads normalized per million of total plant + viral reads), resulting in a 2.4-fold decrease in accumulation of total helper virus mRNA. This is more pronounced than a 2.1-fold decrease in accumulation of total helper virus DNA (**Fig 7A** versus **Fig 7D**). For each BBTV component, the average mRNA load was reduced, albeit at a different degree. For DNA-N, the reduction is most pronounced (about 3-fold) and notable, because mRNA-N is the most abundant in the absence of alphasatellite: it does remain the most abundant in the presence of alphasatellite, but its relative abundance becomes substantially lower in the BBTV mRNA formula (55% vs 67%), while mRNAs of other components become either relatively more abundant (M, R, S) or remain almost unchanged (C, U3) (**Fig 7A**). Remarkably, neither loads nor formulas of viral mRNAs correlated with those of viral DNAs (**Fig 7A** versus **Fig 7D**). This suggests differences in their transcription rates likely due to unequal strength of their Pol II promoters. Normalization of mRNA counts (in reads per million) by viral DNA loads and by transcription unit sizes to estimate mRNA transcription rates revealed that the least abundant DNA-S (1.1% of total viral DNA) is the most transcriptionally efficient component (61.1% of total), followed by DNAs N (20.0%), M (10.7%), U3 (5.7%), R (1.7%) and C (0.8%) (**Fig 8A**). In the presence of alphasatellite, the transcription rate was slightly increased for DNAs S (66.9%), M (13.0%), R (2.3%) and C (1.2%) and reduced for DNAs U3 (4.3%) and N (12.2%), with the latter being the most strongly affected, accounting for the most pronounced negative impact of alphasatellite on the load of mRNA-N. Notably, alphasatellite DNA being the most abundant (42.5% of total viral DNA) exhibits the lowest transcription rate (0.8% of total) (**Fig 8**). Previous analysis of viral DNA and RNA loads in two Cavendish plants sampled in China and found to be co-infected with BBTV and one or two alphasatellites, respectively, revealed substantial differences between viral DNA and RNA formulas [39]. The differences are generally comparable to those observed in our study: alphasatellite DNA was the most abundant, followed by DNAs U3 and N, while alphasatellite RNA was one of the least abundant. Furthermore, mRNA-N was the most abundant in both plants, followed by mRNAs S and U3 and the DNA-N promoter had the highest activity in driving GFP transgene expression in tobacco protoplasts, followed by the promoters of DNAs S and M [39]. In our case, however, the transcription rate of DNA-S was the highest, followed by DNAs N and M (**Fig 8A**).

Taking together, DRC alphasatellite competes with helper virus for both replication and transcription machinery of the host plant, thereby reducing accumulation of helper virus DNA and mRNA, and has the most pronounced negative impact on DNA-N transcription by Pol II. Together these effects of alphasatellite may synergistically reduce transmission efficiency of the helper virus, since mRNA-N encodes a putative aphid transmission factor (as described above).

Besides higher-abundance reads representing the monodirectional mRNA units, lower-abundance reads were found to cover complete (or near-complete) forward and reverse strands of each BBTV component and alphasatellite (**Figs 6** and **S14** and **S5 Dataset**). This is indicative of pervasive transcription of the entire viral DNA in both sense and antisense orientations by Pol II or another RNA polymerase(s). In contrast to mRNA, viral antisense transcript loads and formulas in the presence or absence of alphasatellite generally well reflect viral DNA loads and formulas with exception for DNAs R and S showing higher antisense transcription levels on the expense of DNAs C, M and N (**Fig 7B** versus **Fig 7D**). These effects of alphasatellite on antisense transcription may in turn modulate expression of respective viral genes at transcriptional and/or posttranscriptional levels. Indeed, antisense and other aberrant viral transcripts would induce the antiviral RNAi machinery generating viral siRNAs that potentially direct both transcriptional silencing of viral DNA and posttranscriptional silencing of viral mRNAs.

### DRC alphasatellite affects viral siRNA production

**Fig 9:**
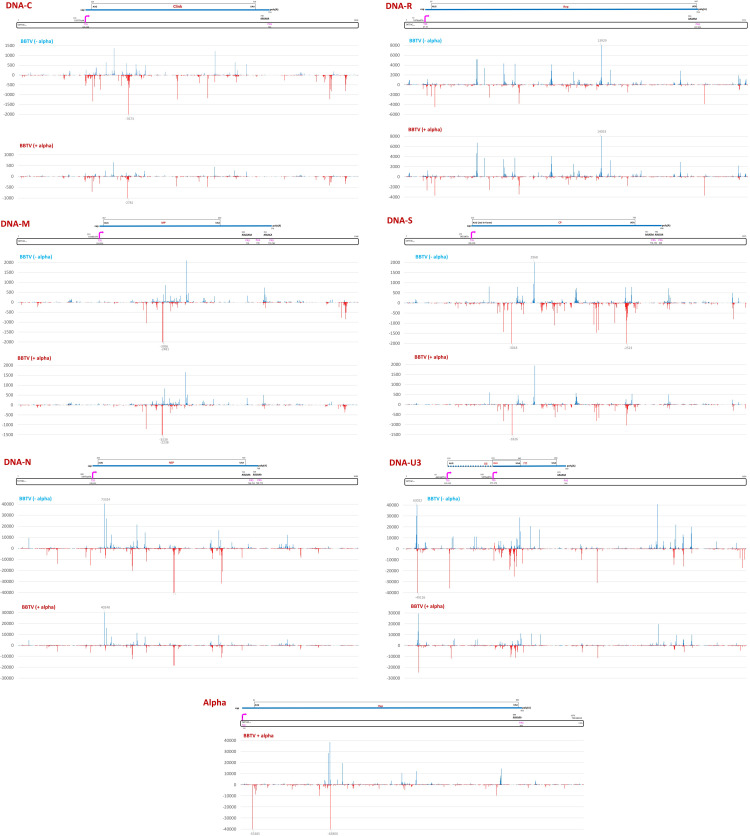
Single nucleotide resolution maps of Illumina sRNA-seq reads representing viral siRNAs from BBTV-infected Cavendish banana plants with or without DRC alphasatellite. For each of the two conditions, i.e. without (BBTV-alpha) and with (BBTV+alpha) alphasatellite, Illumina 20–25 nt reads of the three biological replicates (leaf tissues of three plants) were combined and mapped simultaneously onto the reference sequences of six BBTV components (-/+ alphasatellite). Histograms plot the numbers of viral 20–25 nt reads at each nucleotide position of the 1018-to-1111 nt BBTV genome components (DNAs C, M, N, R, S, U3) and 1105 nt alphasatellite (Alpha): blue bars above the axis represent sense reads starting at each respective position, while red bars below represent antisense reads ending at the respective position. The genome organizations of BBTV components and alphasatellite are shown schematically above the respective histograms, with the Pol II promoter (TATA-box and transcription start site, TSS) and terminator (polyA signal, PAS) elements indicated in pink, capped and polyadenylated mRNA shown as solid blue lines, viral protein-coding ORFs boxed and their nucleotide positions given.

**Fig 10:**
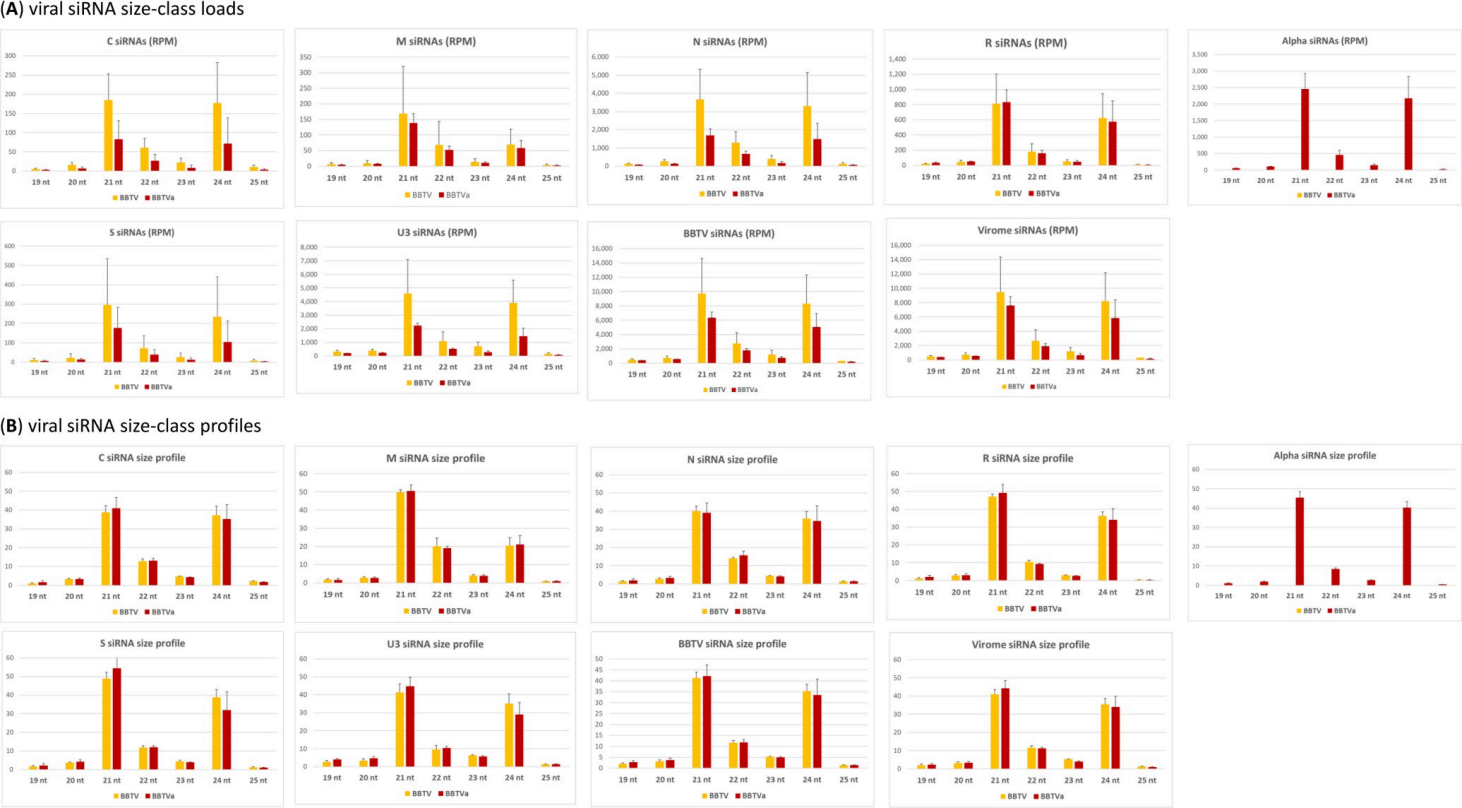
Impact of alphasatellite on accumulation and relative abundance of viral siRNA size-classes. For each of the two conditions, i.e. without (BBTV) and with (BBTVa) alphasatellite, Illumina sRNA-seq 19–25 nt reads of each of the three biological replicates (leaf tissues of three plants) were mapped with zero mismatches onto the reference sequences of complete viral genome components (DNAs C, M, N, R, S, U3, alpha). For each component the loads of each viral sRNA size class were counted in reads per million (RPM) of total plant+viral reads and plotted in panel (**A**), and the relative abundance (formula) of viral sRNA size classes was then calculated in percentage of total and plotted in panel (**B**). Error bars represent standard deviations.

Mapping Illumina sRNA-seq reads (15–34 nt range) onto viral reference sequences with zero mismatches revealed that viral siRNAs predominantly belong to 21, 22 and 24 nt classes and cover both strands of the entire BBTV genome and alphasatellite, with their hotspots being unequally distributed between viral components (**Figs 9** and **10** and **S6 Dataset**). In the presence of alphasatellite, the average load of helper virus-derived siRNAs (normalized per million of total plant+viral sRNA reads) was reduced by 50%, which is proportional to 2.1-fold reduction in its DNA load (**Fig 7C** versus **Fig 7D**). This overall reduction affected all the BBTV genome components except R whose siRNA loads are comparable in the presence and absence of alphasatellite despite a 1.7-fold reduction in the average load of DNA-R. Remarkably, relative abundance of helper virus components-derived siRNAs (siRNA formula) resembles its DNA formula in that DNAs U3 and N spawn the first and second most abundant siRNAs (47.4 and 39.1% of total), followed by DNA-R (7.5%) (**Fig 7C** versus **Fig 7D**). However, the least abundant DNA-S spawns siRNAs at a higher rate (calculated as an siRNA load divided by a DNA load) than other components (~2, 3, 4, 5 and 8 times higher than DNAs N, U3, R, M and C, respectively; **Fig 8C**) and as a result the relative abundance of DNA-S-derived siRNAs (2.6%) is higher than that of siRNAs derived from higher-abundance DNAs M (1.3%) and C (2.1%) (**Fig 7C** versus **Fig 7D**). In the presence of alphasatellite, siRNA production rates were increased for DNAs R and M but not other components (**Fig 8C**), which resulted in a pronounced increase in relative abundance of siRNAs derived from both DNA-R (from 7.5 to 14.5%) and DNA-M (1.3 to 2.3%) and a concomitant but much less pronounced decrease in relative abundances of siRNAs derived from other components except DNA-S (showing a slight increase from 2.6 to 2.8%) (**Fig 7C**). The increased siRNA production may in turn downregulate expression of the respective viral genes at both transcriptional and/or posttranscriptional levels and thereby interfere with viral replication (Rep gene) and movement (MP gene). Notably, the siRNA production rate for alphasatellite is somewhat lower than for any BBTV component except DNA-C (**Fig 7C** versus **Fig 7D**), but because its DNA is highly abundant, the load and relative abundance of alphasatellite siRNAs is comparable to and even slightly exceeding those of DNAs N and U3 (**Fig 7C** versus **Fig 7D**).

Alphasatellite-mediated increase in the production rates of siRNAs from DNAs M and R is proportional for all three major siRNA classes (21, 22 and 24 nt) whose average loads remain unaltered despite the reduction in loads of DNAs M and R. In contrast, average loads of 21, 22 and 24 nt siRNAs derived from other components are strongly and proportionally reduced in the presence of alphasatellite (**Fig 10A**). For each component, relative abundance of viral siRNA size classes (size profile) is comparable in the presence and absence of alphasatellite (**Fig 10B** and **S4D Dataset**). Thus, DRC alphasatellite does not appear to suppress or induce any specific plant DCL activity previously shown to generate 21 nt (DCL4), 22 nt (DCL2) or 24 nt (DCL3) viral siRNAs in ssDNA begomovirus-infected plants [55,56,59] (reviewed in [62] and [52]). Likewise, 5’-nucleotide identity profiles of the three major viral siRNA classes, dominated by 5’U (>50% in the 21 nt and 22 nt classes) and 5’A (>60% in the 24 nt class), were not significantly affected by alphasatellite (**S4D Dataset**), indicating unaltered activities of the AGO proteins recognizing respective 5’-nucleotides as previously established in other plants [54]. These findings are consistent with our above-described finding that transient expression of alphasatellite Rep did not interfere with transgene silencing in *N*. *benthamiana*.

Despite drastic differences in the hotspot profiles of sense vs antisense siRNAs (**Fig 9**), their overall accumulation levels are comparable for both alphasatellite and helper virus, although DNAs C and S spawn respectively 2- and 1.6-times more abundant antisense siRNAs, while DNAs R and U3 spawn respectively 1.7- and 1.3-times less abundant antisense siRNAs. Alphasatellite did not have any substantial impact on the hotspot profiles or sense/antisense ratios of siRNAs derived from any component or complete BBTV genome **(Fig 9** and **S4** and **S6 Datasets**). The siRNA hotspots are mostly concentrated within the mRNA transcription units on both sense and antisense strands but they also occur outside of them and on both strands, most notably in DNAs C and U3 (**Fig 9**). This suggests that dsRNA precursors of viral siRNAs are derived from the entire BBTV genome and alphasatellite and that the antisense transcripts may represent antisense strands of the dsRNA precursors that were not fully processed by DCLs into siRNAs. Consistent with this hypothesis, the loads and formulas of antisense transcripts roughly resemble those of siRNAs, with exception for DNAs R and N generating respectively higher and lower abundance antisense transcripts as could be expected from their relative DNA loads (**Fig 7**). Nonetheless, the most abundant virome components—alphasatellite and DNA-U3 (in the absence of alphasatellite)—produce both most abundant antisense RNAs and most abundant siRNAs.

### Conclusions and open questions

In this work, we report a new alphasatellite, representative of a new genus in the family *Alphasatellitidae*, which got associated with BBTV in DRC, the first country in Sub-Saharan Africa where BBTD was recorded in 1958. Recent emergence of this alphasatellite is emphasized by the fact that it was found only in one of 401 banana samples collected in DRC in 2012 and that it was absent in samples from Gabon, Nigeria, Benin and Togo collected in the following years. Tight association of BBTV with this alphasatellite is manifested by its presence in viruliferous aphids collected in DRC in 2016, 4 years after the first sampling (at a distance of ca. 100 km), as well as its ability to be transmitted by aphids under laboratory conditions in the following 5 years. This is despite its negative impact on helper virus accumulation in plants and aphids and on virus transmission by aphids. Nonetheless, we found that this alphasatellite can be lost during or after aphid transmission in a variable number of recipient plants depending on source plants and/or experimental conditions. Remarkably, the loss of alphasatellite correlated with a delay in BBTV symptom development, a phenomenon that remains to be further investigated.

Our molecular analyses revealed that DRC alphasatellite competes with helper virus for components of the host DNA replication and transcription machinery, thereby reducing viral DNA and mRNA accumulation and altering relative abundances and transcription rates of viral genome components. Most notable are its pronounced negative effects on accumulation of DNA-C encoding the replication enhancer protein and on transcription rate of DNA-N encoding a nuclear shuttle protein implicated in aphid transmission. The former effect would further contribute to reducing viral DNA replication efficiency, while the latter would further interfere with virus acquisition and/or transmission by aphids. Indeed, we found that aphids were not able to transmit the virus lacking DNA-N. Although alphasatellite DNA accumulates in plants at higher levels than any component of the helper virus, it appears to be less efficiently encapsidated by viral coat protein and, as a result, becomes only the third most abundant component in aphids. Thus, in the presence of alphasatellite, which reduces loads of all viral DNAs including DNA-S, the coat protein might also become a limiting factor for encapsidation of helper virus components and hence their acquisition by aphids. All these findings at the molecular level explain the observed negative impact of DRC alphasatellite on virus transmission by aphids, highlighting apparent costs for the helper virus to be in a complex with a self-replicating competitor for the host cell resources. What would then be the benefits (if any) for the helper virus to compensate for those costs, which could explain persistence of the DRC alphasatellite that we observed under the laboratory and field conditions? Since BBTV components engage the host nuclear and cytoplasmic machinery in virus multiplication and movement and thereby induce defense responses and disease symptoms, the alphasatellite-mediated reduction in loads of all viral components may prolong life of diseased plants under field conditions and in turn increase chances for both helper virus and alphasatellite to be transmitted to new plants. Another potential benefit would be that highly abundant alphasatellite DNA could serve as a decoy diverting antiviral RNAi machinery from the helper virus. Indeed, we found that DRC alphasatellite spawns 21, 22 and 24 nt siRNAs and antisense transcripts whose levels exceed those spawned by any component of the helper virus and that the transcription rate of alphasatellite mRNA is one of the lowest, indicating potent RNAi targeting alphasatellite DNA in the nucleus. In further support of the decoy hypothesis, the production rate of siRNAs from the least abundant DNA-S was decreased in the presence of alphasatellite, concomitant with slightly increased transcription rate of mRNA-S. This would compensate for the reduced load of DNA-S, which might become a limiting factor for helper virus encapsidation as argued above. On the other hand, production of 21, 22 and 24 nt siRNAs and antisense transcripts from DNA-R was not decreased in the presence of alphasatellite as their loads remain unaltered and their relative abundance becomes substantially higher. The same was observed for DNA-M, although at much lower levels of siRNA and antisense RNA production. In fact, the loads and transcription rates of DNA-R and DNA-M are least affected by alphasatellite, which explains unaltered RNAi activity targeting these components. Keeping in line with the decoy hypothesis, DRC alphasatellite does not interfere with DCL or AGO activities, as neither size-class nor 5’-nucleotide profiles of viral siRNAs are altered in its presence. Consistent with these findings, alphasatellite Rep protein does not suppress transgene silencing in *N*. *benthamiana*.

Our phylogenetic analysis of DRC alphasatellite, placing its genus deeply within the subfamily *Nanoalphasatellitinae* in close vicinity of the genus *Fabenesatellite* comprising alphasatellites infecting legumes and other dicot Fabaceae hosts, suggests its provenance from a dicot plant. In fact, all members of *Nanoalphasatellitinae* infect dicots. Moreover, our comparison of alphasatellite Rep proteins revealed that the DRC alphasatellite is also related to alphasatellites of the genus *Gosmusatellite* (subfamily *Geminialphasatellitinae*) which infect dicot Malvaceae and Asteraceae hosts. In further support of the dicot origin hypothesis, DRC alphasatellite and BBTV clones could co-infect *N*. *benthamiana*, a dicot plant of the family Solanaceae, and the alphasatellite DNA alone could persist in symptomless *N*. *benthamiana* following recovery from BBTV disease. Such persistence of alphasatellite in the absence of helper virus can be explained by its ability to self-replicate and to evade plant defense responses.

So far BBTV was not reported to infect any dicot plant, consistent with the fact that its specialized vector *P*. *nigronervosa* (banana aphid) is restricted to monocots of the families Musaceae, Araceae and Zingiberaceae, two of which (Musaceae and Zingiberaceae) contain known BBTV hosts. Our findings raise a possibility that BBTV can potentially be transmitted by banana aphids to some dicot plant(s), where it could have encountered a DRC alphasatellite progenitor being associated with a nanovirus. Even a short-term feeding of viruliferous banana aphids on such a nanovirus and alphasatellite co-infected plant would be sufficient to acquire an encapsidated alphasatellite DNA and transmit it back to a banana or other BBTV host plant. Alternatively, some other aphid species feeding on a nanovirus and alphasatellite host plant could have transmitted a DRC alphasatellite progenitor to a BBTV-infected banana or other plant. It should be noted that the origin of alphasatellites associated with BBTV in South-East Asia is not known, although phylogenetically they are all classified into the subfamily *Petromoalphasatellitinae* comprising alphasatellites of perennial tropical monocots. Rep proteins of these alphasatellites are only distantly related to those of DRC alphasatellite and other members of *Nanoalphasatellitinae*. It remains to be investigated by more comprehensive surveys of cultivated and non-cultivated plants how frequent in their evolution alphasatellites could swap genera (and perhaps even families) of helper viruses and thereby expand their host plant range. Likewise, further investigation of molecular mechanisms underlying alphasatellite-helper virus-host plant interactions and insect vector transmission of alphasatellite-helper virus complexes should shed more light on costs and benefits of alphasatellites for 6-to-8-component viruses of the family *Nanoviridae*, in comparison with 1-to-2-component viruses of the family *Geminiviridae*.

## Materials and methods

### Banana aphids and plants

The clonal populations of *Pentalonia nigronervosa* aphids established and maintained at PHIM originate from Democratic Republic of the Congo (DRC aphids) and Gabon (GAB aphids). The DRC aphids were collected separately from symptomless and BBTD-infected banana plantain plants (10 adult insects per plant) in the DRC province Congo Central (village Boko) in December 2016. The GAB aphids were kindly provided by Dr. Sebastien Massart in 2017, after their rearing on healthy banana plants for >15 years at the University of Liege (Gembloux, Belgium).

*In vitro* seedlings of *Musa acuminata* triploid (AAA) banana cultivar Cavendish obtained from Vitropic (Saint Mathieu de Treviers, France) were grown under the tropical conditions (26°C, 70–80% humidity) and then taken for aphid rearing and transmission tests.

### Aphid transmission tests

The DRC aphids collected from the BBTD-infected and symptomless banana plants were placed onto Cavendish seedlings (at a 4-leaf stage) in two separate insect-proof plastic boxes (box 4 for the infected plant aphids and box 5 for the symptomless plant aphids). The boxes were placed in a phytochamber at 23°C/19°C day/night temperature and 16/8 hours day/night length and after three months moved into another phytochamber at 24°C (day and night), 70% humidity, 12/12 hours day/night length. After about two-three weeks, one of the three plants in the box 4 (plant p4.3) started to develop the characteristic BBTD symptoms and later got severely infected (**S1A Fig**). This plant was used as a source plant for the transmission tests depicted in **S2 Fig**.

To perform BBTV transmission using viruliferous aphids grown on the source plant p4.3, about 15 adult insects from this plant were placed on healthy *M*. *acuminata* seedlings. The recipient plants were maintained in insect-proof plastic boxes in a phytochamber at 24°C (day and night), 70% humidity, 12/12 hrs day/night length.

To carry out BBTD transmission using virus-free DRC and GAB aphids reared on healthy Cavendish plants, about 30–40 adult insects were taken from those plants and placed on a detached leaf from the source plant p4.3 for virus acquisition. After 24 hours, the insects were transferred to healthy Cavendish seedlings for virus transmission as described above. The leaf tissues and aphids were sampled at different time points for (IC-)PCR and RCA analyses (**S1B, S1C** and **S1D Fig**).

To test if a single aphid can transmit BBTV, adult aphids were selected from BBTD-infected plants (plant p4.4 of the box 4 for DRC aphids and plant 1 of the box 2 for GAB aphids). Each aphid was placed on a banana leaf surface in a micro-container (aphid placed under the leaf) allowing it to feed (but not escape). After one day, each aphid was collected for PCR analysis of viral DNA-R. The recipient plants were taken from the 24°C chamber and, after chemical treatment to kill the aphids, placed in the greenhouse to monitor the development of BBTV symptoms. One month post-inoculation, upper leaf samples were taken for IC-PCR analysis of viral DNA-R (**S1D** and **S1E Fig**).

To study the impact of DRC alphasatellite on BBTV transmission, two consecutive large-scale transmission experiments (C and D) were performed. The BBTV-infected source plants with and without alphasatellite were selected using duplex IC-PCR with DNA-R and alphasatellite specific primers (**S1 Table**) from recipient plants of the above-described transmission experiments with DRC aphids. The transmission experiments were performed in a humid phytochamber under the tropical conditions as described above. Virus-free DRC aphids were placed on each source plant to feed for three weeks and build a colony of viruliferous aphids. Adult aphids born on the source plant were then taken from each plant to inoculate 12 recipient Cavendish seedlings (5 aphids per recipient plant) grown inside an insect-proof cage (one cage per source plant). After 4 days of feeding, all aphids were collected from each recipient plant in pools and frozen. The follow-up monitoring of the recipient plants as well as molecular analyses of the recipient plants and the viruliferous aphids are described in the Results section and detailed in **S2 Dataset**. To analyze statistical significance of alphasatellite impact on BBTV transmission, we used the R package lsmeans [76] to calculate least-square means using a linear model where the success of viral transmission was a function of the transmission experiment and the presence of alphasatellite in source plants. The impact of alphasatellite on delay in appearance of the first and systemic symptoms was evaluated using a non-parametric Kruskal-Wallis test [77].

### Immuno-capture (IC)-PCR analysis of encapsidated viral DNA

To detect viral DNA encapsidated in BBTV virions, an IC-PCR test was performed using the commercial coating antibody for BBTV (Agdia; http://www.agdia.com). Sterile polypropylene 0.2 ml microfuge tubes were coated with 25 μl of the BBTV antibody diluted at 1:100 in carbonate buffer [15 mM Na_2_CO_3_, 34 mM NaHCO_3_, pH 9.7] for 4 hrs at 37°C (or overnight at 4°C), followed by washing three times with 200 μl of 1X PBS-T buffer [8 mM Na_2_HPO_4_, 150 mM NaCl, 2 mM KH_2_PO_4_, 3 mM KCl, 0.05% Tween 20, pH 7.4]. Plant extracts were prepared by grinding 0.5 g fresh leaf tissue in 5 ml grinding buffer [2% polyvinylpyrrolidone (PVP), 0.2% sodium sulfite, 0.2% bovine serum albumin in 1X PBS-T buffer] in plastic grinding bags (Agdia). Aliquots of the plant extracts (25 μl) were centrifuged at 7000 rpm for 3 min at room temperature and the supernatant was loaded into the BBTV-antibody coated tubes. The tubes were incubated at 37°C for 4 hrs or at 4°C overnight and then washed three times with 200 μl of 1X PBS-T buffer and one time with 200 μl of RNase-free water, followed by brief air-drying. Then, 30 μl DNase solution [3 μl of 10X buffer (400 mM Tris-HCl pH 8.0, 100 mM MgSO_4_, 10 mM CaCl_2_), 3 μl of RQ1 RNase-Free DNase (1U/μl, Promega), and 24 μl water] was added to remove potential contaminant DNA, followed by incubation for 30 min to 1 hr at 37°C. The DNase was inactivated at 95°C 10 min and the solution was removed by pipetting. The PCR reactions were carried out directly in the tube in a total volume of 25 μl [5 μl of 5X Green GoTaq Reaction Buffer (Promega), 10 mM forward and reverse primers (or 2 pairs of primers in the case of duplex PCR; **S1 Table**), 10 mM dNTPs (2.5 mM each), 0.2 μl (5 U/μl) GoTaq DNA Polymerase (Promega), and milli-Q water to adjust the volume]. The reaction mixture was denatured at 98°C for 5 min, followed by 30 cycles of 30 sec at 95°C, 30 sec at 58°C and 30 sec at 72°C, and finally one cycle at 72°C for 10 min.

### Total DNA extraction from banana leaves

Fresh leaf tissue (100 mg) was ground in liquid nitrogen and 500 μl extraction buffer [100 mM Tris-HCl pH 8.0, 1.4 M NaCl, 20 mM EDTA, 2% alkyltrimethylamonium bromide (MATAB), 1% polyethyleneglycol 6000, 0.5% sodium sulfite] pre-heated at 74°C and supplemented with 0.4 μl RNase (100 mg/ml) was added to the frozen powder. The mixture was vortexed for 20 sec, incubated at 74°C for 20 min and then mixed vigorously with one volume of chloroform-isoamyl alcohol (24:1 v/v) (CIAA), followed by centrifugation at 13000 rpm for 30 min at 4°C. The supernatant was taken for a second round of extraction with CIAA followed by centrifugation as described above. The supernatant was mixed with one volume of isopropanol pre-cooled at -20°C. The mixture was shaken until the appearance of a hank and then spun at 13000 rpm and 4°C for 30 min. The pellet was washed twice with 500 μl of 70% ethanol, air dried and dissolved in 100 μl of mili-Q water.

### Total DNA extraction from aphids

Total DNA was extracted from alive aphids or aphids stored at -80°C. Aphids were grounded in 150 μl TNES buffer (50 mM Tris-HCl pH 7.5, 400 mM NaCl, 20 mM EDTA, 0.5% SDS) with a pestle. The resulting crude extract was mixed with 45 μl of cold 5 M NaCl, followed by centrifugation at 6500 rpm for 10 min at 4°C. The supernatant was mixed with 500 μl of cold absolute ethanol and incubated for 20 min at -80°C, followed by centrifugation at 14000 rpm for 20 min at 4°C. The pellet was washed with 250 μl of 70% ethanol, followed by centrifugation at 14000 rpm for 10 min at 4°C. The supernatant was discarded, and the pellet was dried and resuspended in 50 μl H_2_O.

### Rolling circle amplification (RCA) of viral DNA

Circular viral DNA components were amplified by RCA using a TempliPhi RCA kit (GE Healthcare) following the manufacturer protocol. Briefly, 5 μl Sample buffer and 1 μl total DNA extracted from aphids or banana leaf tissues were mixed and heated at 95°C for 3 min. The samples were cooled and 5 μl Reaction buffer and 0.2 μl enzyme mix were added, followed by incubation at 30°C for 18 hours. The enzyme was inactivated by heating at 65°C for 10 min.

### Restriction analysis and purification of RCA products

RCA products were digested with the restriction enzyme (RE) AvaI or DraI as follows: 4 μl RCA product were mixed with 1.5 μl of RE buffer and 1 μl (1 U/μl) of RE in a final volume of 15 μl and incubated at 37°C for 1 hr. The digested products were analyzed by agarose gel electrophoresis (**S1G** and **S1H Fig**). Undigested RCA products were purified using NucleoSpin Gel and PCR Clean-up kit (Macherey-Nagel, USA) following the manufacturer protocol. DNA concentration was measure by Qubit fluorimeter using Qubit dsDNA HS Assay Kit (Thermo Fischer Scientific).

### Illumina sequencing of RCA products and *de novo* reconstruction of viral genomes

Fifty ng of the cleaned undigested RCA products were taken for Illumina sequencing at Fasteris AG (www.fasteris.com). Libraries were prepared using Nextera XT standard DNA protocol and 11 libraries (JGF1-11, **S3 Fig**) were multiplexed and sequenced in one flowcell of HiSeq2500 with a 2x 125-nt paired-end run. Viral genomes were *de novo* reconstructed from the sequencing reads of each library by selecting unique inserts sequenced 5, 10, 20, 30, 40 or 50 times and assembling them using Velvet v. 1.2.10 [78] (https://www.ebi.ac.uk/~zerbino/velvet/) with k-mers 77, 79, 83, 87, 91, 95, 99, 103, 107, 111, 113 and 117. All the resulting Velvet contigs were scaffolded using SeqMan Pro v. 7.1.0 (DNASTAR Lasergene). SeqMan contigs of viral origin were identified by BLASTn analysis. The consensus viral genome sequences were verified using SeqMan scaffolds and validated by mapping back the Illumina reads using BWA [79] and visualization using MISIS-2 [80].

### PCR and qPCR analyses of viral DNA in plants and aphids

BBTV diagnostics in banana plants and aphids was performed by PCR using a pair of primers (5’-GGCGCGATATGTGGTATGCTGG and 5’-CCAAACTCGAAGGGACCTTCG) specific for a DNA-R region conserved in BBTV isolates from DRC [65] yielding a 285 bp product. The PCR reaction was performed in a volume of 25 μl containing 1 μl total DNA (ca. 40 ng plant DNA or ca. 10 ng of aphid DNA), 2 μl primer mix (10 μM each primer), 1 μl of 2.5 mM dNTPs, 5 μl of 5x GoTaq buffer and 1U GoTaq DNA polymerase (Promega). After denaturation at 94°C for 5 min, DNA was amplified for 30 cycles of 30 sec at 94°C, 30 sec at 60°C, 30 sec at 72°C, followed by a final extension at 72°C for 10 min.

Quality of total DNA extracted from banana samples, was verified by PCR with a pair of primers (5’-TCCTTTCGCTCTATGCCAGT and 5’-GCCCATCGGGAAGTTCATAG) specific the actin-101-like gene (NCBI Genbank accession XM_009410731).

For duplex and multiplex PCR analysis of the *de novo* reconstructed BBTV components and alphasatellite from the DRC-2016 isolate, the primers (**S1 Table**) were designed using the web-tool Multalin (http://multalin.toulouse.inra.fr/), considering amplicon sizes to be differentiated on 2% agarose gel, and verified using the NCBI Primer-Blast. Degenerate primers for alphasatellite detection (**S6 Fig** and **S1 Table**) were designed by multiple sequence alignment of DRC-2016 alphasatellite to all alphasatellites of the genus *Fabenesatellite* [41] using Seaview [81] to identify conserved regions for primer annealing sites with minimal SNPs.

Quantitative (q)PCR with specific primers designed with the web-tool Primers3 v.0.4.0 (https://bioinfo.ut.ee/primer3-0.4.0/) for each BBTV core component and DRC-alphasatellite as well as banana RPS2 or aphid EF1a housekeeping gene (**S1 Table**) was carried out in 384-well plates using a fluorescent label SYBR Green (Roche) and LightCycler 480 thermal cycler (Roche). The reaction mixture was denatured at 95°C for 5 min, followed by 40 cycles of 10 sec at 95°C, 10 sec at 56°C and 10 sec at 72°C. Each sample was replicated twice on the same plate. The fluorescence was converted into ng of DNA by referring to a standard curve carried out on each plate (one curve per amplicon). Each standard curve consisted of five dilutions of a mixture of the PCR products obtained for each BBTV component, DRC alphasatellite and housekeeping gene separately with their qPCR primer pairs. The qPCR data were analyzed using LinRegPCR 12.11.0.0 [82] to calculate DNA concentrations. The concentrations were normalized by internal housekeeping genes using Excel to calculate the loads of each BBTV component and alphasatellite. Statistical analysis of the data was performed with Rstudio [83] using a non-parametric Kruskal-Wallis test [77], because DNA concentrations and normalized loads of viral genome components in plant and aphid samples did not follow a normal distribution. Figures were prepared using the R packages ggplot2 [84].

### Construction of infectious clones of DRC-2016 alphasatellite and BBTV components

The BBTV components and alphasatellite were cloned from the total DNA extracted from viruliferous DRC-2016 aphids (sample JGF-5, **S2 Fig**) using inverse overlapping PCR primers designed on a unique restriction site in each viral component (**S1 Table**). The PCR products were digested with the respective restriction enzymes and cloned into pMiniT 2.0 vector using NEB PCR cloning kit. A bitmer fragment of each viral component (except DNA-R) containing the common-region stem-loop (CR-SL) was initially cloned from the pMiniT2.0 constructs into pBluescript II SK(-) and the full-length onemer fragment was subsequently cloned in the bitmer clone to construct the partial tandem dimer clones. In the case of DNA-R, the full-length insert was directly cloned from the pMiniT2.0 backbone into pBluescript II SK(-) and a clone with two copies of DNA-R in tandem repeat was chosen as the dimer clone. The partial dimer and the dimer inserts were subcloned from the pBluescript II SK(-) constructs into the binary vector pCAMBIA2300 using convenient restriction sites (**S9 Fig**). All the clones were verified by both restriction analysis and Sanger sequencing after each step of cloning. *Agrobacterium tumefaciens* strain EHA105 was transformed with these binary plasmids and the intactness of the viral inserts in the resulting agrobacterial strains was again confirmed by both restriction analysis and Sanger sequencing.

### Agroinoculation of *N*. *benthamiana* plants

*N*. *benthamiana* wild type and 16c line seeds were sown in a nursery pot and maintained in a phytochamber at 20–21°C, 16 hrs daylight and 8 hrs darkness. Then, seedlings were transplanted in individual pots and 3 weeks-old seedlings were used for agroinoculation. One fresh colony from each strain of EHA105 *A*. *tumefaciens* carrying the binary construct was inoculated into pre-culture of 2 ml Luria-broth (LB) medium supplied with appropriate antibiotics and grown for 8 hrs at 28°C. Then, all the pre-cultures were added in 50 ml of LB medium supplied with appropriate antibiotics and grown overnight at 28°C. Bacterial cells were pelleted and resuspended in agro-inoculation buffer [10 mM MES, 10 mM MgCl2, 100 μM acetosyringone] at optical density at 600 nm (OD600) = 1. Mixture of agrobacteria at OD600 = 1 containing all six BBTV components +/- alphasatellite in equal proportions was then prepared and placed in darkness at room temperature for 3 hrs and then 1 ml of the mixture was used for agro-infiltration of younger leaves of *N*. *benthamiana* with a syringe. The inoculated plants were kept in a phytochamber at the above-described conditions and leaf tissue samples were harvested at 8, 21 and 60 days post inoculation (dpi) for molecular analysis.

### Transient expression analysis of BBTV and alphasatellite proteins in *N*. *benthamiana*

The ORFs of the DRC-2016 alphasatellite and BBTV components were PCR amplified from either the pMiniT2.0 or the pBluescript II SK(-) constructs and cloned under the control of CaMV 35S promoter and terminator in the binary plasmid pB7WG2 as detailed in **S10A** and **S10B Fig**. These plasmids were transformed into *Agrobacterium tumefaciens* strain GV3101 and their intactness in the resulting agrobacterial strains was confirmed by restriction analysis and Sanger sequencing of the viral ORF inserts. Transient expression analysis of the viral ORF constructs was performed using a classical silencing suppression assay in leaves of *N*. *benthamiana* 16c line plants as we described in detail previously [85].

### Total RNA extraction from banana leaves

RNA extraction was performed as described previously [86]. Briefly, 0.5 g fresh leaf tissue was ground in liquid nitrogen and mixed with 2.5 ml extraction buffer [100 mM Tris-HCl (pH 7.5), 500 mM NaCl, 25 mM EDTA (pH 8.0), 1.5% SDS, 2% polyvinylpyrrolidone, 0.7% beta-mercaptoethanol (added just before use)]. The mixture was incubated for 10 min at room temperature and then centrifuged at 3700 rpm for 15 min at 4°C. The supernatant was taken and mixed with 1/3 volume of 5 M sodium acetate (pH 6.0) and kept on ice for 30 min, followed by centrifugation at 13000 rpm for 15 min at 4°C. The supernatant was taken and mixed vigorously with an equal volume of phenol/chloroform/isoamyl alcohol (25:24:1), followed by centrifugation at 13000 rpm for 10 min at 4°C. The aqueous phase was taken and mixed vigorously with one volume of chloroform/isoamyl alcohol (24:1), followed by centrifugation as described above. The aqueous phase was collected and mixed with one volume of isopropanol, followed by incubation for 5 min at room temperature and for 30 min at -80°C. RNA was pelleted by centrifugation for 15 min at a maximum speed at 4°C. The pellet was washed with 500 μl of 75% ethanol, air-dried and dissolved in 50 μl RNase-free water.

### Illumina sequencing and bioinformatics analysis of viral transcriptome and small RNA-ome

Integrity of high and low molecular weight RNA extracted from banana leaves was verified by capillary electrophoresis on LabChip GX (Perkin Elmer). Illumina sequencing was performed at Fasteris AG (www.fasteris.com) using the same total RNA extracts for library preparations with (i) Illumina stranded mRNA protocol (DNase treatment, poly-A selection, transcripts fragmentation, 1st strand cDNA synthesis with random primers, 2nd strand cDNA synthesis, ligation of indexed adapters, amplification of the library) and (ii) Illumina TruSeq small RNA protocol (acrylamide gel size selection of the 18–30 nt range, single strand ligation of the 3’ adapter, single strand ligation of the 5’ adapter, cDNA synthesis by reverse transcription, amplification of the library). The mRNA libraries were multiplexed and sequenced in one flowcell of NovaSeq 6000, yielding 28’568’938 to 42’820’006 75 nt paired-end reads with Q30 = 92.6 to 93.5. The sRNA libraries were multiplexed and sequenced in one flowcell of NovaSeq 6000 yielding 33’698’415 to 50’148’596 reads with Q30 = 95.5 to 95.9. In both cases, the libraries were de-multiplexed, followed by adapter trimming. The resulting reads were mapped using Burrow-Wheeler Aligner (BWA) 0.7.12 [79] onto the reference sequences of DRC-2016 alphasatellite and six BBTV components with and without mismatches. Mapped viral reads were sorted by polarity (forward, reverse) and, in the case of sRNAs, by size (15 to 34 nts) and 5´-terminal nucleotide identity (5’U, 5’A, 5’G, 5’C) and counted, followed by normalization in reads per million (RPM) of total (viral + non-viral) reads (**S3** and **S4 Datasets**). Additionally, mRNA-seq reads were mapped with BWA onto the reference sequences of the BBTV and alphasatellite mRNA units (spanning from the transcription start site and the polyadenylation site indicated in **Figs 6** and **S13**; note that in the case of U3 the shorter unit was taken). The mapped forward reads representing viral mRNAs, viral antisense transcripts (mapped on full-length references) and total viral siRNAs were counted in RPM and the resulting values were divided by viral DNA load values (qPCR concentrations/RPS2) for the respective components and alphasatellite to calculate transcription rates of viral mRNAs by Pol II and antisense transcription rates. Likewise, siRNA production rates were calculated as counts (in RPM) of viral sense+antisense siRNAs (mapped to full-length references) divided by viral DNA loads. Mean values with standard deviations of the mRNA, antisense transcript, total siRNA and siRNA size-class read counts (in RPM) and the mRNA transcription rates and siRNA production rates in three biological replicates per condition (+/- alphasatellite) were calculated and plotted in **Figs 7** and **10**. Relative abundances of viral mRNAs, viral antisense transcripts, total siRNAs and size-classes of siRNAs from six BBTV components and alphasatellite were calculated as percentage of reads representing each component in total BBTV and virome (BBTV + alphasatellite) and the mean values of three biological replicates with standard deviations plotted in “BBTV formula” and “Virome formula” panels of **Figs 7**, **8** and **10**.

Single-nucleotide resolution maps of viral mRNA reads (75 nt) and viral siRNAs (a 20–25 nt range) shown in **Figs 6** and **9** and **S13** and **S14** were generated using MISIS-2 [80] that analyses BAM files of the mapped reads yielding table files (**S5** and **S6 Datasets**) and visualizes mapped reads and SNPs at each position of the reference sequence (see **S13 Fig**).

### Phylogenetic analysis of alphasatellites

Reference sequences of all isolates of alphasatellites available in the NCBI Genbank in September 2021 were downloaded, adjusted to begin with the conserved nonanucleotide sequence TAGTATTAC and aligned to the DRC alphasatellite sequences (OK546211 and OK546212) using Sequence Demarcation Tool (SDT) v1.2 [87] with Muscles. A maximum likelihood phylogenetic tree was constructed using Seaview [81] with Muscles and FigTree v1.4.4 (https://github.com/rambaut/figtree/releases). Clustering analysis of alphasatellite Rep protein sequences was performed using CLANS [88].

## Supporting information

S1 Table Primers used for immune-capture (IC)-PCR, single, duplex and multiplex PCR and quantitative (q)PCR analyses. (PDF)

S1 Fig Banana bunchy top disease (BBTD) transmission to *Musa acuminata* Cavendish plants by the banana aphids from DRC (field DRC aphids) and Gabon (GAB aphids), followed by molecular analysis of recipient plants and aphids. (**A**) Pictures of healthy and BBTV-infected Cavendish banana plants, following disease transmission by the field aphids collected on a symptomatic banana plant in the DRC province Bas Congo (Congo-Central) in December 2016. (**B**) Aphid feeding on a detached leaf of BBTV-infected plant for 24 hrs to acquire the virus. (**C**) The symptomatic recipient plant colonized by aphids, following disease transmission by GAB aphids. (**D**) Immuno-capture (IC)-PCR of leaf tissues and PCR analysis of total DNA from respectively GAB aphids-inoculated recipient plants and aphids from the corresponding recipient plants (p2.1, p2.2 and p2.3). Position of DNA-R PCR product is indicated by arrow. (**E**) Virus transmission with a single viruliferous aphid placed on a recipient plant leaf. (**F**) Immuno-capture (IC)-PCR analysis of recipient plants upon transmission with single GAB and DRC aphids at 4 weeks post-inoculation. (**G**) Restriction analysis of RCA amplified viral DNA from the BBTD plant using AvaI and DraI enzymes. Positions of the undigested multimeric RCA product and the monomeric AvaI digestion products are indicated by arrows. (**H**) AvaI restriction analysis of RCA amplified viral DNA from BBTV-infected recipient plants (p1, p2, p3 and p4) and aphids taken from these plants. Positions of undigested and digested RCA products are indicated. In panels D, F, G and H, “M” stands for 1 Kb DNA ladder and positions of its 1018 and 298 bp bands are indicated.
(PDF)

S2 Fig Banana bunchy top disease transmission by viruliferous *P*. *nigronervosa* aphids from DR-Congo (DRC) and virus-free banana aphids from Gabon (GAB) (maintained in Belgium for >15 years) and the plant and aphid samples used for rolling circle amplification and Illumina sequencing of viral DNA. Scheme of the transmission experiments with the field DRC aphids and GAB aphids fed on a detached leaf the first infected plant (p4.3) is depicted with the collected samples of leaf tissues (JGF-1-4) and aphids (JGF-5-11) indicated with green and orange circles, respectively. T0 and T1 are two sampling time-points for the first infected plant p4.3.
(PDF)

S3 Fig Genetic variants of BBTV components and alphasatellite identified in Cavendish plants and aphids taken from these plants by rolling circle amplification and Illumina sequencing of viral DNA. For each of the 11 samples (JGF-1-11, see **S2 Fig**) of BBTD-infected plant leaf tissues and viruliferous aphids (indicated with green and orange circles, respectively), the Illumina sequencing reads were mapped on the *de novo* reconstructed sequences of six BBTV components (C, M, N, R, S, U3) and alphasatellite (α) and the mapped reads were analyzed using MISIS-2 [80] to identify single-nucleotide polymorphism (SNP) positions and calculate percentage (%) of each SNP variant (v1 and v2 for DNAs M and S, and v1, v2, v3 and v4 for DNA-U3) at each SNP position. Single variants of virome components (without SNPs exceeding 10%) are highlighted in green. Arcs with arrowheads indicate from which plants the aphid samples were collected.
(PDF)

S4 Fig Maps and relative abundance of Illumina sequencing reads representing RCA-amplified DNA of BBTV genome components and alphasatellite identified in BBTD-infected Cavendish plants and aphids taken from these plants. For each of the 11 samples (JGF-1-11, see **S2 Fig**) of BBTD-infected plant leaf tissues and viruliferous aphids (indicated with green and orange circles, respectively), the Illumina 125 nt reads were mapped on concatenated sequences of alphasatellite (α) and six BBTV components (C, M, N, R, S, U3) and the mapped reads were visualized using MISIS-2 [80] and counted. Histograms plot the numbers of viral 125 nt forward and reverse reads at each nucleotide position of the concatenated viral genome: blue bars above the axis represent forward reads ending at each respective position red bars below the axis represent reverse reads ending at each respective position. Numbers of reads mapped to each viral genome component are given below the histograms.
(PDF)

S5 Fig Multiplex, duplex and single PCR validation of the BBTV genome components and DRC alphasatellite reconstructed by Illumina sequencing of RCA-amplified viral DNA from BBTD-infected Cavendish plants and aphids taken from these plants. (**A**) Schematic representation of circular BBTV genome components (C, M, N, R, S, U3) and alphasatellite with position of diagnostic PCR primers (yellow arrows). (**B**) Multiplex, duplex and single PCR analysis of the 11 samples (JGF-1-11, see **S2 Fig**) of BBTD-infected plant leaf tissues and viruliferous aphids (indicated with green and orange circles, respectively). PCR products were separated on 2% agarose gel. Positions of each BBTV component and alphasatellite are indicated by arrows.
(PDF)

S6 Fig PCR and immuno-capture (IC)-PCR analyses with alphasatellite-specific primers of banana plant samples from PIO (Sub-Saharan Africa and New Caledonia). (**A**) PCR analysis of total DNA extracted from BBTV-infected plants from DRC (2012) using DRC-2016 alphasatellite-specific primers (**S1 Table**), followed by IC-PCR analysis of selected leaf samples. Position of the alphasatellite (α)-specific PCR product on each gel is indicated by an arrow. (**B**) Multiplex PCR analysis of RCA products of viral DNA from selected BBTV-infected plant samples from New Caledonia (NCL), Malawi (MLW), Benin (BEN), Gabon (GAB) and DRC using the primers for each BBTV component and DRC alphasatellite (**S5 Fig**) with positions of respective PCR products indicated by arrows. (**C**) Multiplex PCR analysis of RCA-amplified DNA of BBTV-infected plants from Togo (TGO), Benin (BEN) and Nigeria (NGA) (2019) using the primers for each BBTV component and DRC alphasatellite (**S5 Fig**) with positions of respective PCR products indicated by arrows, followed by single PCR analysis with degenerate PCR primers specific for DRC alphasatellite and all members of the genus *Fabenesatellite* (**S1 Table**). (**D**) Duplex and single PCR analysis of total DNA of BBTD-infected plants from Benin (BEN) (2020), using DRC- and *Fabenesatellite*-specific degenerate primers. Position of alphasatellite (α)-specific PCR product on each gel is indicated by an arrow. DRC alphasatellite-infected and healthy Cavendish plants were used as respectively positive “α (+)” and negative “α (-)” controls. Note that the PCR analysis of DNA samples from DRC revealed two alphasatellite-positive samples (368 and 381), while the follow-up IC-PCR of leaves confirmed the presence of encapsidated alphasatellite only in sample 381 (panel A), possibly due to cross-contamination during DNA extraction. Illumina sequencing of RCA amplified viral DNA (pre-analyzed by multiplex PCR, panel B) revealed that the DNA samples 368 and 381 contain identical sequences of the alphasatellite and six BBTV components.
(PDF)

S7 Fig Pairwise sequence comparison of DRC alphasatellite isolates DRC-2016 (OK546211) and DRC-2012 (OK546212) with all other alphasatellites. The nucleotide sequences of DRC alphasatellite isolates were compared with those of all alphasatellites from the subfamilies *Petromoalphasatellitinae* (**A**), *Nanoalphasatellitinae* (**B**) and *Geminialphasatellitinae* (**C**) available at the NCBI Genbank in September 2021 using Sequence Demarcation Tool (SDT) v1.2 [87] with Muscles (excluding indels) and their pairwise identities (in %) were plotted as heatmap diagrams and shown in Tables. Two isolates of DRC alphasatellite are indicated with red arrows.
(PDF)

S8 Fig Clustering analysis of Rep proteins encoded by DRC-2016 alphasatellite and alphasatellites associated with ssDNA viruses of the families *Nanoviridae*, *Metaxiviridae* and *Geminiviridae*. Rep proteins of the alphasatellites classified by Briddon et al. [41] (see Table 1 of that paper for the NCBI accession numbers) were compared “all-against-all” and clustered using CLANS [88]. DRC alphasatellite (DRC alpha) is indicated with a bright green cube close to the middle and its evolutionary relatedness (connection) to other alphasatellites is shown with solid grey lines whose color intensity—from lightest to darkest—indicates the strength of connections from worse (no direct connection) to best. Each alphasatellite genus is color-coded and named. Grey circles indicate unassigned alphasatellites. Note that the genus *Babusatellite* comprising four species (indicated with black diamonds) was recently split into two genera: *Babusatellite* and *Muscarsatellite*.
(PDF)

S9 Fig Construction of BBTV and alphasatellite infectious clones. The complete sequences of BBTV components and alphasatellite from the DRC-2016 aphids (sample JGF-5, **S2 Fig**) were cloned as partial (DNAs C, M_v1, N, S_v1, U3_v1) or complete (DNA-R) dimers first in pBluescript SK(-) (**A**) and then in pCambia2300 (**B**). For each construct, positions of the duplicated common region stem loop (CR-SL) are indicated with red quadrates.
(PDF)

S10 Fig Analysis of BBTV and alphasatellite proteins for silencing suppression and chlorosis induction activities in *Nicotiana benthamiana*. (**A-B**) Subcloning of BBTV and alphasatellite ORFs from partial dimer constructs (**A**) (see **S9 Fig**) into the binary vector pB7WG2 under the control of cauliflower mosaic virus 35S promoter (35S) and terminator (35S 3’) (**B**) by PCR amplification with viral ORF-specific primers carrying AttB1 and AttB2 recombination sites (**S1 Table**), followed by Gateway recombination of the PCR products into the vector. (**C-D**) Screening of BBTV and alphasatellite ORFs for silencing suppression and chlorosis induction following infiltration of the binary constructs in leaves of *N*. *benthamiana* GFP-transgenic (16c line) plants at 8 days post infiltration (dpi) under ultraviolet (UV) light (**C**) and day light (**D**). Pictures of representative leaves (from three infiltrated plants per construct, three leaves per plant) are shown. The experiment was repeated 3 times with similar results. Note that in addition to the BBTV Rep construct inducing strong chlorosis, the BBTV NSP construct induced weaker chlorosis, albeit it was barely visible in some of the infiltrated leaves.
(PDF)

S11 Fig Impact of DRC alphasatellite on viral DNA concentrations in plants and aphids. Viral DNA concentrations for each BBTV component (C, M, N, R, S, U3) and alphasatellite (a) and for total helper virus without (BBTV) or with (BBTVa) alphasatellite were measured by quantitative PCR using equal amounts of total DNA in (**A**) plants without (n = 77) and with (n = 47) alphasatellite (all experiments) and (**E**) aphids without (n = 58) and with (n = 56) alphasatellite (all experiments). Concentrations of total host DNA in the same samples were measured by qPCR with primers specific for the banana and aphid housekeeping genes (RPS2 and eEF1a, respectively). * Kruskal-Wallis P < 0.05. *** Kruskal-Wallis P < 0.005.
(PDF)

S12 Fig Viral DNA loads and formulas in BBTV-infected Cavendish banana plants with or without DRC alphasatellite selected for viral transcriptome and small RNA-ome profiling. Viral DNA loads for each BBTV component (C, M, N, R, S, U3) and alphasatellite (alpha) as well as for total helper virus (BBTV) were measured by quantitative PCR (using the banana RPS2 gene as internal control for normalization) in 3 plants infected with BBTV alone (ADVT-2, ADVT-5, ADVT-6) and 3 plants co-infected with BBTV and alphasatellite (ADVT-8, ADVT-9, ADVT-10). (**A**) Loads of helper virus (BBTV, yellow) and alphasatellite (alpha, red) DNA in plants without and with alphasatellite. (**B**) Mean loads of helper virus (BBTV, yellow) and alphasatellite (alpha, red) DNA in plants without (BBTV) and with (BBTVa) alphasatellite. (**C**) Mean loads of each BBTV component and alphasatellite (alpha) DNA in plants with (red) and without (yellow) alphasatellite. (**D**) Loads of each BBTV component and alphasatellite (alpha) in plants without (yellow) and with (red) alphasatellite. (**E**) Helper virus genome formula in plants without (yellow, BBTV) and with (red, BBTVa) alphasatellite. (**F**) Virome genome formula in plants without (yellow, BBTV) and with (red, BBTVa) alphasatellite. Error bars represent standard deviations.
(PDF)

S13 Fig Single nucleotide resolution maps of Illumina mRNA-seq reads representing viral mRNAs and other viral transcripts from BBTV-infected Cavendish banana plants with or without DRC alphasatellite and identification of poly(A) sites by analysis of the mapped reads. For each of the two conditions, i.e. without (BBTV-alpha) and with (BBTV+alpha) alphasatellite, Illumina 75 nt reads of the three biological replicates (leaf tissues of three plants) were combined and mapped simultaneously onto the reference sequences of six BBTV components (-/+ alphasatellite). Histograms plot the numbers of viral 75 nt sense and antisense reads at each nucleotide position of the 1018-to-1111 nt BBTV genome components (DNAs C, M, N, R, S, U3—subpanels **A1-F1**) and 1105 nt alphasatellite (Alpha–subpanel **G1**): blue bars above the axis represent sense reads starting at each respective position, while red bars below the axis represent antisense reads ending at each respective position. The genome organizations of BBTV components and alphasatellite are shown schematically above the respective histograms, with the Pol II promoter (TATA-box and transcription start site, TSS) and terminator (polyA signal, PAS) elements indicated in pink, capped and polyadenylated mRNA shown as solid blue lines, viral protein-coding ORFs boxed and their nucleotide positions given. In each panel (**A-G**), subpanels 2 and 3 show the Pol II terminator region with the mapped poly(A) sites and the respective poly(A) signals at the upstream positions visualized using MISIS-2 [80]. Note that the mapped mRNA reads ending with oligo(A) tails generate A-SNPs in the sequence logo just downstream of the poly (A) site (i.e., pre-mRNA cleavage and polyadenylation site).
(PDF)

S14 Fig Single nucleotide resolution maps of Illumina mRNA-seq reads representing viral antisense transcripts from BBTV-infected Cavendish banana plants with or without DRC alphasatellite. For each of the two conditions, i.e. without (BBTV-alpha) and with (BBTV+alpha) alphasatellite, Illumina 75 nt reverse reads of the three biological replicates (leaf tissues of three plants) were combined and mapped simultaneously onto the reference sequences of six BBTV components (-/+ alphasatellite). Histograms plot the numbers of viral 75 nt antisense reads at each nucleotide position of the 1018-to-1111 nt BBTV genome components (DNAs C, M, N, R, S, U3) and 1105 nt alphasatellite (Alpha): red bars below the axis represent antisense reads ending at each respective position. The genome organizations of BBTV components and alphasatellite are shown schematically above the histograms, with the Pol II promoter (TATA-box and transcription start site, TSS) and terminator (polyA signal, PAS) elements indicated in pink, capped and polyadenylated mRNA shown as solid blue lines, viral protein-coding ORFs boxed and their nucleotide positions given.
(PDF)

S1 Dataset Complete nucleotide sequences of the alphasatellite-BBTV isolates DRC-2016 and DRC-2012 from Democratic Republic of the Congo (DRC). (**A**) Nucleotide sequences of the aphid-borne alphasatellite-BBTV isolate DRC-2016 and its genetic variants in evolving quasispecies population in the *Musa acuminata* Cavendish recipient plants and in the progenies of the DRC and GAB aphids having transmitted the disease to the infected recipient plants (samples JGF-1-11; **S2, S3** and **S4 Figs**). (**B**) Nucleotide sequences of the alphasatellite-BBTV isolate DRC-2012 from a leaf sample collected from a BBTD-infected AAB banana plant in Bas Congo province in 2012. (**C**) Pairwise comparison of the DRC-2016 and DRC-2012 alphasatellite nucleotide sequences and encoded Rep proteins. (**D**) Protein and nucleotide sequence Blast analyses of DRC alphasatellite isolates.
(PDF)

S2 Dataset A summary spreadsheet of the aphid transmission experiments C and D with (i) viral DNA concentrations and normalized loads in the source plants, the aphid pools following a 4-days inoculation period and the recipient plants and (ii) delays (in days post-inoculation) in development of first and systemic disease symptoms in the recipient plants.
(XLSX)

S3 Dataset Counts of Illumina mRNA-seq reads from Cavendish banana plants infected with BBTV or BBTV and alphasatellite. The Illumina 75 nt paired-end reads from each library were mapped without (**A**) or with (**B**) mismatches to the reference sequences of DRC-2016 alphasatellite and BBTV genome components (DNAs C, M, N, R, S, U3), sorted by polarity (forward, reverse, total) and counted. The counts of viral reads mapped without mismatches were then normalized per million of total reads (RPM) in each library (**C**).
(XLSX)

S4 Dataset Counts of Illumina sRNA-seq reads from Cavendish banana plants infected with BBTV or BBTV and alphasatellite. The 15–34 nt reads from each library were mapped without (**A**) or with (**B**) mismatches to the reference sequences of DRC-2016 alphasatellite and BBTV genome components (DNAs C, M, N, R, S, U3), sorted by size (15 nt, 16 nt, 17 nt, 18 nt, 19 nt, 20 nt, 21 nt, 22 nt, 23 nt, 24 nt, 25 nt, 26 nt, 27 nt, 28 nt, 29 nt, 30 nt, 31 nt, 32 nt, 33 nt, 34 nt, total 15–34 nt) and polarity (forward, reverse, total) and counted. The viral reads mapped without mismatches were then normalized per million of total reads (RPM) in each library (**C**) and also sorted by 5’-terminal nucleotide identity (5’A, 5’C, 5’G, 5’U) (**D**).
(XLSX)

S5 Dataset Single nucleotide resolution maps of Illumina mRNA-seq reads representing viral transcripts from BBTV-infected Cavendish banana plants with or without DRC alphasatellite. For each of the two conditions, i.e. without (**BBTV**) and with (**BBTVa**) alphasatellite, Illumina 75 nt forward and reverse reads of the three biological replicates (leaf tissues of three plants) were combined and mapped simultaneously onto the reference sequences of six BBTV components (**C**, **M**, **N**, **R**, **S**, **U3**) and DRC-2016 alphasatellite (**alpha**). BAM files with mapped viral reads were analyzed by MISIS-2 [80] yielding the count tables of forward and reverse reads, starting or ending at respective positions of the reference sequences.
(XLSX)

S6 Dataset Single nucleotide resolution maps of Illumina sRNA-seq reads representing viral siRNAs from BBTV-infected Cavendish banana plants with or without DRC alphasatellite. For each of the two conditions, i.e. without (**BBTV**) and with (**BBTVa**) alphasatellite, Illumina 20–25 nt reads of the three biological replicates (leaf tissues of three plants) were combined and mapped simultaneously onto the reference sequences of (**C**, **M**, **N**, **R**, **S**, **U3**) and DRC-2016 alphasatellite (**alpha**), extended at the 3-end by 33 nts from the 5-terminal sequence to allow for mapping sRNAs derived from the circular viral genome. BAM files with mapped viral reads were analyzed by MISIS-2 [80] yielding the count tables of forward and reverse reads of each size-class, starting or ending at respective positions of the reference sequences.
(XLSX)
