# Supplementary material for: A newly emerging alphasatellite affects banana bunchy top virus replication, transcription, siRNA production and transmission by aphids
Source: PLoS Pathog. 2022 Apr 12;18(4):e1010448. doi: 10.1371/journal.ppat.1010448 (PMC9049520; doi:10.1371/journal.ppat.1010448)
Supplement: S1 Table — (PDF) [file ppat.1010448.s001.pdf]

S1 Table. Primers used for immune-capture (IC)-PCR, single, duplex and multiplex PCR and quantitative (q)PCR analyses.

Name original	Specific to / strand	Sequence (5' to 3')	Size of Amplicon
IC-PCR and duplex PCR for detection of DRC-2016 alphasatellite and BBTV			
BBTV a-sat_F	DRC alphasatellite forward	AATTCGGAGAAATTCTGCTGAAGG	187 bp
BBTV a-sat_R	DRC alphasatellite reverse	GGTTTTTTGCCACGAACGTA	
V_BBTV_R_F	DNA-R forward	AGATGAGAGGCTTCTTCCCAG	494 bp
V_BBTV_R_R	DNA-R reverse	AATATCCTCGTAATTATACAGTCTACA	
Diagnostic single PCR for detection of DRC-2016 and DRC-2012 alphasatellite			
Alpha_NcoI_F	DRC alphasatellite forward	GCCATGGATGAATCTCGGAA	285 bp
a-sat V_R	DRC alphasatellite reverse	CTTTCGTACACCTGGGGAAA	
Multiplex PCR for DRC-2016 alphasatellite and BBTV			
Alpha_NcoI_F	DRC alphasatellite forward	GCCATGGATGAATCTCGGAA	285 bp
a-sat V_R	DRC alphasatellite reverse	CTTTCGTACACCTGGGGAAA	
V_BBTV_C_F	DNA-C forward	CGAGATGCGAAAATGGAGGC	355 bp
V_BBTV_C_R	DNA-C reverse	TGATAAAAGAACACCATGCTGTTTA	
V_BBTV_M_F	DNA-M forward	GATATAAGGGACATAACGGGTTCAG	217 bp
V_BBTV_M_R	DNA-M reverse	ATGCCATTTAGCAGGGTCCTATT	
V_BBTV_N_F	DNA-N forward	GTCAGCAATGATTATAATGGGCTTT	540 bp
V_BBTV_N_R	DNA-N reverse	CTGTTACTAATACTAACTTCCATGTCT	
V_BBTV_R_F	DNA-R forward	AGATGAGAGGCTTCTTCCCAG	494 bp
V_BBTV_R_R	DNA-R reverse	AATATCCTCGTAATTATACAGTCTACA	
V_BBTV_S_F	DNA-S forward	GGGCTAATGGATTGTGGATATAG	184 bp
V_BBTV_S_R	DNA-S reverse	TTTGTTGTCTGAACATCTATCTAC	
V_BBTV_U3_F	DNA-U3 forward	CGGCCCAATAATTAAGAGAACG	412 bp
V_BBTV_U3_R	DNA-U3 reverse	TAAACATTAACACACCCAGG	
Degenerate primers for detection of fabenesatellites and DRC alphasatellite			
deg_Fabenesat_s	alphasatellite forward	CAYGACCAYMTHCAGGGAGT	296 bp
deg_Fabenesat_as	alphasatellite reverse	CGAGATKCDTCCATGGCGGA	
qPCR primers for DRC-2016 alphasatellite and BBTV			
DRC-alphasatellite	DRC alphasatellite forward	TCACGACCACCTACAGGGAG	85 bp
	DRC alphasatellite reverse	GTGTGGATTTCCCCCGATCA	
BBTV DNA-C	DNA-C forward	CGAGATGCGAAAATGGAGGC	96 bp
	DNA-C reverse	TGGCAGACGATTCCCAGAAC	
BBTV DNA-M	DNA-M forward	GGCTGTCATACCACAGGCAT	88 bp
	DNA-M reverse	CATAGGTCCGACGTTTCCTCG	
BBTV DNA-N	DNA-N forward	CTGAGATGCCTGTGGTATGAC	103 bp
	DNA-N reverse	CCGATAATCGACAATATGTGC	
BBTV DNA-R	DNA-R forward	CAACAATCCCACAACACTACC	103 bp
	DNA-R reverse	AACATAACCTTGCACATGACG	
BBTV DNA-S	DNA-S forward	CTTGGGAAATCAACCAGCCG	109 bp
	DNA-S reverse	CCTGCTTCAAGTTCCCCACT	
BBTV DNA-U3	DNA-U3 forward	ACGGACCGAAATACTCCTGC	88 bp
	DNA-U3 reverse	CAGCACAACCACCTTGACAG	
Banana RPS2	RPS2 forward	TAGGGATTCCGACGATTTGTTT	84 bp
	RPS2 reverse	TAGCGTCATCATTGGCTGGGA	
Aphid EF1α	EF1α forward	AGATTGGACAAACCCGTGAA	96 bp
	EF1α reverse	GCTGTATGGTGGTTCAGTAGAG	

Primers for cloning DRC-2016 alphasatellite and BBTV components

RR1 C-FL HincII s	DNA-C forward	actgGTCAACATCAATAAAGAAGAAGGAATATTC
RR2 C-FL HincII as	DNA-C reverse	actgGTTGACGTCTATCTTTCGCTGC
RR3 C-bit BglII s	DNA-C forward bitmer	actgAGATCTCTATTGTGATGAGGTTC
RR4 M-FL HindIII s	DNA-M forward	gtcaAAGCTTGGCAACCACCACTTTAG
RR5 M-FL HindIII as	DNA-M reverse	gtcaAAGCTTATCCGACACAAATATACTC
RR6 M-bit HincII as	DNA-M reverse bitmer	gtcaGTTAACCTGAGATGCCTGTGG
RR7 N-FL HindIII s	DNA-N forward	cagtAAGCTTCAGCGGAAATAATAGGAAC
RR8 N-FL HindIII as	DNA-N reverse	cagtAAGCTTCCGTTAAACACAGCTTCAA
RR9 N-bit ScaI s	DNA-N forward bitmer	cagtAGTACTCATAGTACCATTCGGTGG
RR10 R-FL Eco01 s	DNA-R forward	agtcAGGTCCCTTCGAGTTTGGTGCATTTA
RR11 R-FL Eco01 as	DNA-R reverse	agtcGGGACCTTCGATTCTTGTATCTTCC
RR13 S-FL XbaI s	DNA-S forward	ctagATCTAGAAGTCTATAAATACCAGTGTG
RR14 S-FL XbaI as	DNA-S reverse	ctagTCTAGATGGCTTGCAGGGCAAGC
RR15 S-bit SspI s	DNA-S forward bitmer	ctagAATATTGGTTCCTGAAAACACCGTC
RR16 U3-FL HincII s	DNA-U3 forward	agtcGTCAACATTATTCTGGCTTGCGC
RR17 U3-FL HincII as	DNA-U3 reverse	agtcGTTGACCGGTTACCTTGACCTTC
RR18 U3-bit XbaI s	DNA-U3 forward bitmer	agtcTCTAGAGAGGCGGTGAAGCAAAC
RR19 a-FL EcoRI s	DNA-alpha forward	gtcaGAATTCGGAGAAATTCTGCTGAAGG
RR20 a-FL EcoRI as	DNA-alpha reverse	gtcaGAATTCCCACGGACCTGCGATC
RR21 a-bit SspI s	DNA-alpha bitmer	gtcaAATATTCCTATTATACCCCCGAAGCTC

Primers for cloning ORFs of DRC-2016 alphasatellite and BBTV

RR50 attB1 a-Rep s	GGGGACAAGTTTGTACAAAAAAGCAGGCT**ATG**ACGTGTCATCAGCGAAATTG
RR51 attB2 a-Rep as	GGGGACCACTTTGTACAAGAAAGCTGGGT**TTA**GCAATTTATTATTATTAATCTGTCTCCGGAGATTTTTTC
RR52 attB1 Clink s	GGGGACAAGTTTGTACAAAAAAGCAGGCT**ATG**GAGTTCTGGGAATCGTCTG
RR53 attB2 Clink as	GGGGACCACTTTGTACAAGAAAGCTGGGT**TTA**GAGTAATGTTACATCATAGTCTGATATAACTATCTTCTGACAAT
RR54 attB1 MP s	GGGGACAAGTTTGTACAAAAAAGCAGGCT**ATG**GCATTAACAACAGAGCGGG
RR55 attB2 MP as	GGGGACCACTTTGTACAAGAAAGCTGGGT**TTA**AAACATAGGTCCGACGTTTCCTCGTC
RR56 attB1 NSP s	GGGGACAAGTTTGTACAAAAAAGCAGGCT**ATG**GATTGGGCGGAATCACAATTCAAGAC
RR57 attB2 NSP as	GGGGACCACTTTGTACAAGAAAGCTGGGT**TTA**TTCCTTGATTCTTAAAGAACAAACAACATAACACTTCAGA
RR58 attB1 Rep s	GGGGACAAGTTTGTACAAAAAAGCAGGCT**ATG**GCGCGATATGTGGTATGC
RR59 attB2 Rep as	GGGGACCACTTTGTACAAGAAAGCTGGGT**TCA**GCAAGAAACCAACTTTATTCGATCTTC
RR60 attB1 CP s	GGGGACAAGTTTGTACAAAAAAGCAGGCT**ATG**TTCAGACAACAAATGGCTAGGTAT
RR61 attB2 CP as	GGGGACCACTTTGTACAAGAAAGCTGGGT**TCA**AACATGATATGTAATTCTGTTCTGGTAGTTTATACTTACTCC
RR62 attB1 U3 s	GGGGACAAGTTTGTACAAAAAAGCAGGCT**ATG**ACCGAAGGTCAAGGTAACCGG
RR63 attB2 U3 as	GGGGACCACTTTGTACAAGAAAGCTGGGT**TCA**CCGCCTCTCTAGATGCACATC
