# Supplementary material for: A newly emerging alphasatellite affects banana bunchy top virus replication, transcription, siRNA production and transmission by aphids
Source: PLoS Pathog. 2022 Apr 12;18(4):e1010448. doi: 10.1371/journal.ppat.1010448 (PMC9049520; doi:10.1371/journal.ppat.1010448)
Supplement: S1 Fig — (A) Pictures of healthy and BBTV-infected Cavendish banana plants, following disease transmission by the field aphids collected on a symptomatic banana plant in the DRC province Bas Congo (Congo-Central) in December 2016. (B) Aphid feeding on a detached leaf of BBTV-infected plant for 24 hrs to acquire the virus. (C) The symptomatic recipient plant colonized by aphids, following disease transmission by GAB aphids. (D) Immuno-capture (IC)-PCR of leaf tissues and PCR analysis of total DNA from respectively GAB aphids-inoculated recipient plants and aphids from the corresponding recipient plants (p2.1, p2.2 and p2.3). Position of DNA-R PCR product is indicated by arrow. (E) Virus transmission with a single viruliferous aphid placed on a recipient plant leaf. (F) Immuno-capture (IC)-PCR analysis of recipient plants upon transmission with single GAB and DRC aphids at 4 weeks post-inoculation. (G) Restriction analysis of RCA amplified viral DNA from the BBTD plant using AvaI and DraI enzymes. Positions of the undigested multimeric RCA product and the monomeric AvaI digestion products are indicated by arrows. (H) AvaI restriction analysis of RCA amplified viral DNA from BBTV-infected recipient plants (p1, p2, p3 and p4) and aphids taken from these plants. Positions of undigested and digested RCA products are indicated. In panels D, F, G and H, “M” stands for 1 Kb DNA ladder and positions of its 1018 and 298 bp bands are indicated. (PDF) [file ppat.1010448.s002.pdf]

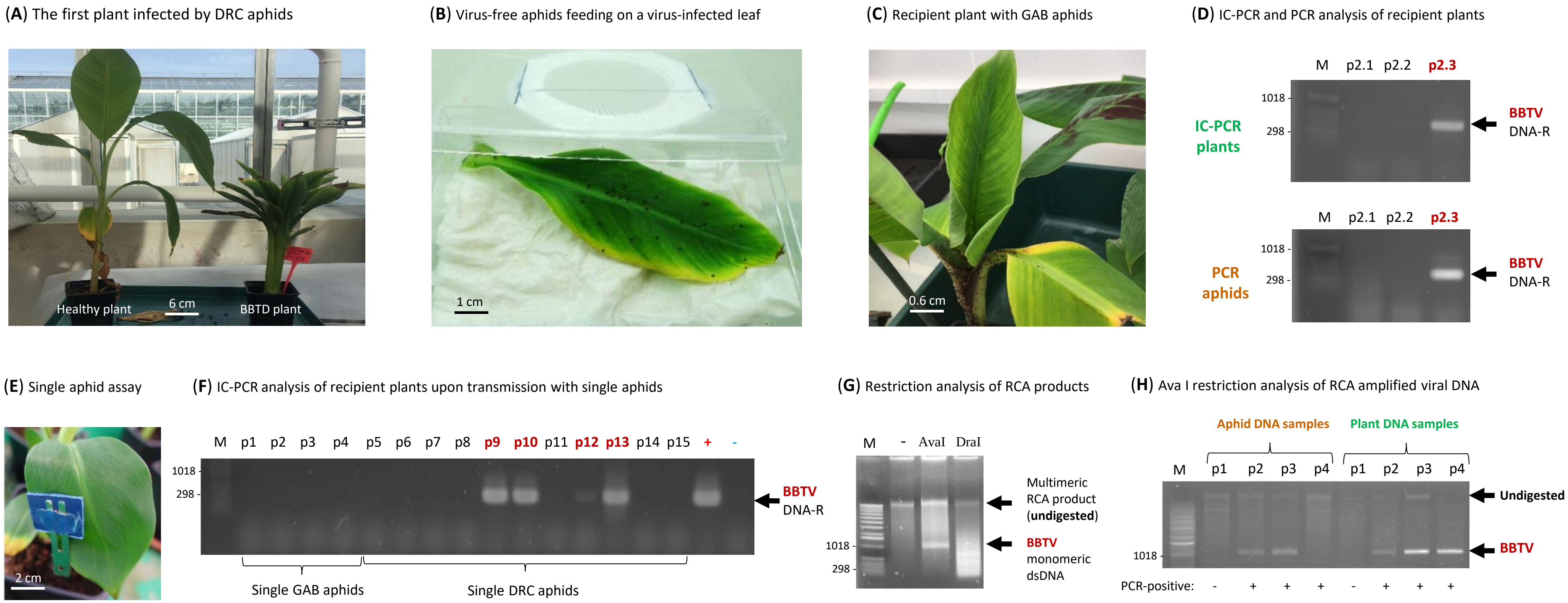

S1 Fig. Banana bunchy top disease (BBTD) transmission to *Musa acuminata* Cavendish plants by the banana aphids from DRC (field DRC aphids) and Gabon (GAB aphids), followed by molecular analysis of recipient plants and aphids. (**A**) Pictures of healthy and BBTV-infected Cavendish banana plants, following disease transmission by the field aphids collected on a symptomatic banana plant in the DRC province Bas Congo (now Congo-Central) in December 2016. (**B**) Aphid feeding on a detached leaf of BBTV-infected plant for 24 hrs to acquire the virus. (**C**) The symptomatic recipient plant colonised by aphids, following disease transmission by GAB aphids. (**D**) Immuno-capture (IC)-PCR of leaf tissues and PCR analysis of total DNA from respectively GAB aphids-inoculated recipient plants and aphids from the corresponding recipient plants (p2.1, p2.2 and p2.3). Position of DNA-R PCR product is indicated by arrow. (**E**) Virus transmission with a single viruliferous aphid placed on a recipient plant leaf. (**F**) Immuno-capture (IC)-PCR analysis of recipient plants upon transmission with single GAB and DRC aphids at 4 weeks post-inoculation. (**G**) Restriction analysis of RCA amplified viral DNA from the BBTD plant using AvaI and DraI enzymes. Positions of the undigested multimeric RCA product and the monomeric AvaI digestion products are indicated by arrows. (**H**) AvaI restriction analysis of RCA amplified viral DNA from BBTV-infected recipient plants (p1, p2, p3 and p4) and aphids taken from these plants. Positions of undigested and digested RCA products are indicated. In panels D, F, G and H, "M" stands for 1 Kb DNA ladder and positions of its 1018 and 298 bp bands are indicated.
