# Supplementary material for: A newly emerging alphasatellite affects banana bunchy top virus replication, transcription, siRNA production and transmission by aphids
Source: PLoS Pathog. 2022 Apr 12;18(4):e1010448. doi: 10.1371/journal.ppat.1010448 (PMC9049520; doi:10.1371/journal.ppat.1010448)
Supplement: S2 Fig — Scheme of the transmission experiments with the field DRC aphids and GAB aphids fed on a detached leaf the first infected plant (p4.3) is depicted with the collected samples of leaf tissues (JGF-1-4) and aphids (JGF-5-11) indicated with green and orange circles, respectively. T0 and T1 are two sampling time-points for the first infected plant p4.3. (PDF) [file ppat.1010448.s003.pdf]

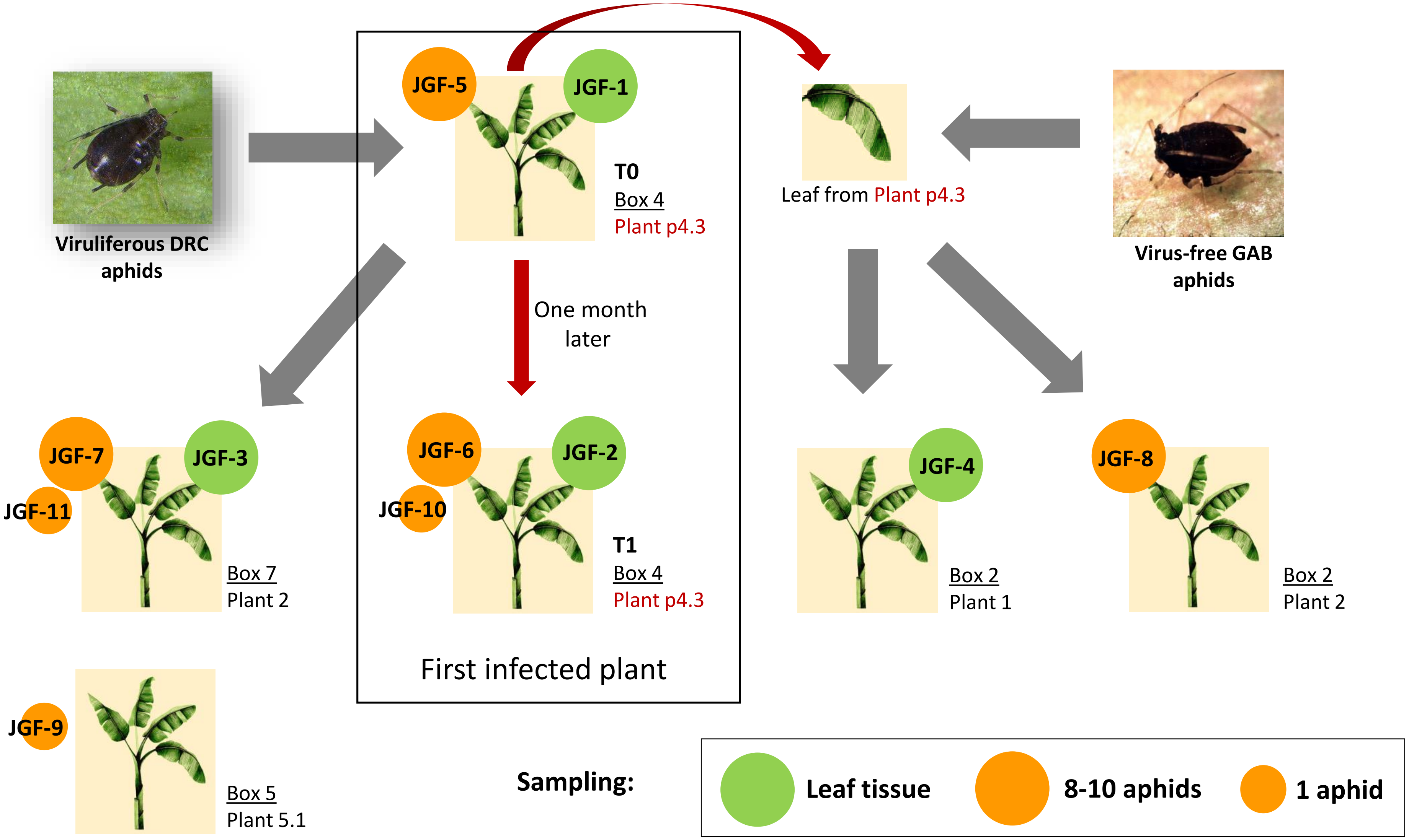

S2 Fig. Banana bunchy top disease transmission by viruliferous *P. nigronervosa* aphids from Democratic Republic of the Congo (DRC) and virus-free banana aphids from Gabon (GAB) (maintained in Belgium for >15 years) and the plant and aphid samples used for rolling circle amplification and Illumina sequencing of viral DNA . Scheme of the transmission experiments with the field DRC aphids and GAB aphids fed on a detached leaf the first infected plant (p4.3) is depicted with the collected samples of leaf tissues (JGF-1-4) and aphids (JGF-5-11) indicated with green and orange circles, respectively. T0 and T1 are two sampling time-points for the first infected plant p4.3.
