# Supplementary material for: A newly emerging alphasatellite affects banana bunchy top virus replication, transcription, siRNA production and transmission by aphids
Source: PLoS Pathog. 2022 Apr 12;18(4):e1010448. doi: 10.1371/journal.ppat.1010448 (PMC9049520; doi:10.1371/journal.ppat.1010448)
Supplement: S3 Fig — For each of the 11 samples (JGF-1-11, see S2 Fig) of BBTD-infected plant leaf tissues and viruliferous aphids (indicated with green and orange circles, respectively), the Illumina sequencing reads were mapped on the de novo reconstructed sequences of six BBTV components (C, M, N, R, S, U3) and alphasatellite (α) and the mapped reads were analyzed using MISIS-2 [80] to identify single-nucleotide polymorphism (SNP) positions and calculate percentage (%) of each SNP variant (v1 and v2 for DNAs M and S, and v1, v2, v3 and v4 for DNA-U3) at each SNP position. Single variants of virome components (without SNPs exceeding 10%) are highlighted in green. Arcs with arrowheads indicate from which plants the aphid samples were collected. (PDF) [file ppat.1010448.s004.pdf]

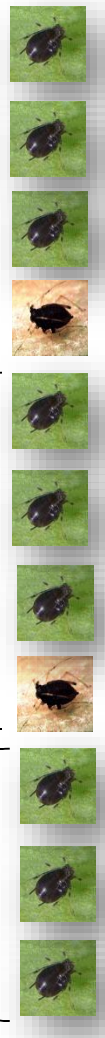

Group	Sample	α	C	M	N	R	S	U3
Leaf samples	JGF-1 MaBBTVrdc 4.3t0	single	single	v1 (70 %) v2 (30 %)	single	single	v1 (70 %) v2 (30 %)	v1 (80 %) v2 (20 %)
	JGF-2 MaBBTVrdc 4.3t1	single	single	v1 (60 %) v2 (40 %)	single	single	v1 (70 %) v2 (30 %)	v1 (70 %) v2 (30 %)
	JGF-3 MaBBTVrdc 7.2	? (730 reads)	single	v1 (100%)	single	single	v1 (100%)	v2 (60 %) v1 (40 %)
	JGF-4 MaBBTVgmb 2.1	single	single	v2 (75 %) v1 (25 %)	single	single	v2 (50 %) v1 (50 %)	v3 (100 %)
8-10 aphids' pools	JGF-5 PnBBTVrdc 4.3t0	single	single	v1 (70 %) v2 (30 %)	single	single	v1 (60 %) v2 (40 %)	v1 (70 %) v2 (30 %)
	JGF-6 PnBBTVrdc4.3t1	single	single	v1 (60 %) v2 (40 %)	single	single	v1 (70 %) v2 (30 %)	v1 (70 %) v2 (30 %)
	JGF-7 PnBBTVrdc7.2	single	single	v1 (100%)	single	single	v1 (100%)	v2 (60 %) v1 (40 %)
	JGF-8 PnBBTVgmb2.2	single	single	v2 (85 %) v1 (15 %)	single	single	v2 (50 %) v1 (50 %)	v3 (100 %)
Single aphids	JGF-9 PnBBTVrdc5.1*	? (922 reads)	single	v1 (100%)	single	single	v1 (100%)	v1 (50 %) v4 (50 %)
	JGF-10 PnBBTVrdc4.3t1*	single	single	v1 (50 %) v2 (50 %)	single	single	v1 (50 %) v2 (50 %)	v1 (70 %) v2 (30 %)
	JGF-11 PnBBTVrdc7.2*	? (441 reads)	single	v1 (100%)	single	single	v1 (100%)	v2 (60 %) v1 (40 %)

S3 Fig. Genetic variants of BBTV components and alphasatellite identified in Cavendish plants and aphids taken from these plants by rolling circle amplification and Illumina sequencing of viral DNA. For each of the 11 samples (JGF-1-11, see Supplementary Figure S2) of BBTD-infected plant leaf tissues and viruliferous aphids (indicated with green and orange circles, respectively), the Illumina sequencing reads were mapped on the *de novo* reconstructed sequences of six BBTV components (C, M, N, R, S, U3) and alphasatellite (α) and the mapped reads were analysed using MISIS-2 (Seguin et al. 2016) to identify single-nucleotide polymorphism (SNP) positions and calculate percentage (%) of each SNP variant (v1 and v2 for DNAs M and S, and v1, v2, v3 and v4 for DNA-U3) at each SNP position. Single variants of virome components (without SNPs exceeding 10%) are highlighted in green. Arcs with arrowheads indicate from which plants the aphid samples were collected.
