# Supplementary material for: A newly emerging alphasatellite affects banana bunchy top virus replication, transcription, siRNA production and transmission by aphids
Source: PLoS Pathog. 2022 Apr 12;18(4):e1010448. doi: 10.1371/journal.ppat.1010448 (PMC9049520; doi:10.1371/journal.ppat.1010448)
Supplement: S4 Fig — For each of the 11 samples (JGF-1-11, see S2 Fig) of BBTD-infected plant leaf tissues and viruliferous aphids (indicated with green and orange circles, respectively), the Illumina 125 nt reads were mapped on concatenated sequences of alphasatellite (α) and six BBTV components (C, M, N, R, S, U3) and the mapped reads were visualized using MISIS-2 [80] and counted. Histograms plot the numbers of viral 125 nt forward and reverse reads at each nucleotide position of the concatenated viral genome: blue bars above the axis represent forward reads ending at each respective position red bars below the axis represent reverse reads ending at each respective position. Numbers of reads mapped to each viral genome component are given below the histograms. (PDF) [file ppat.1010448.s005.pdf]

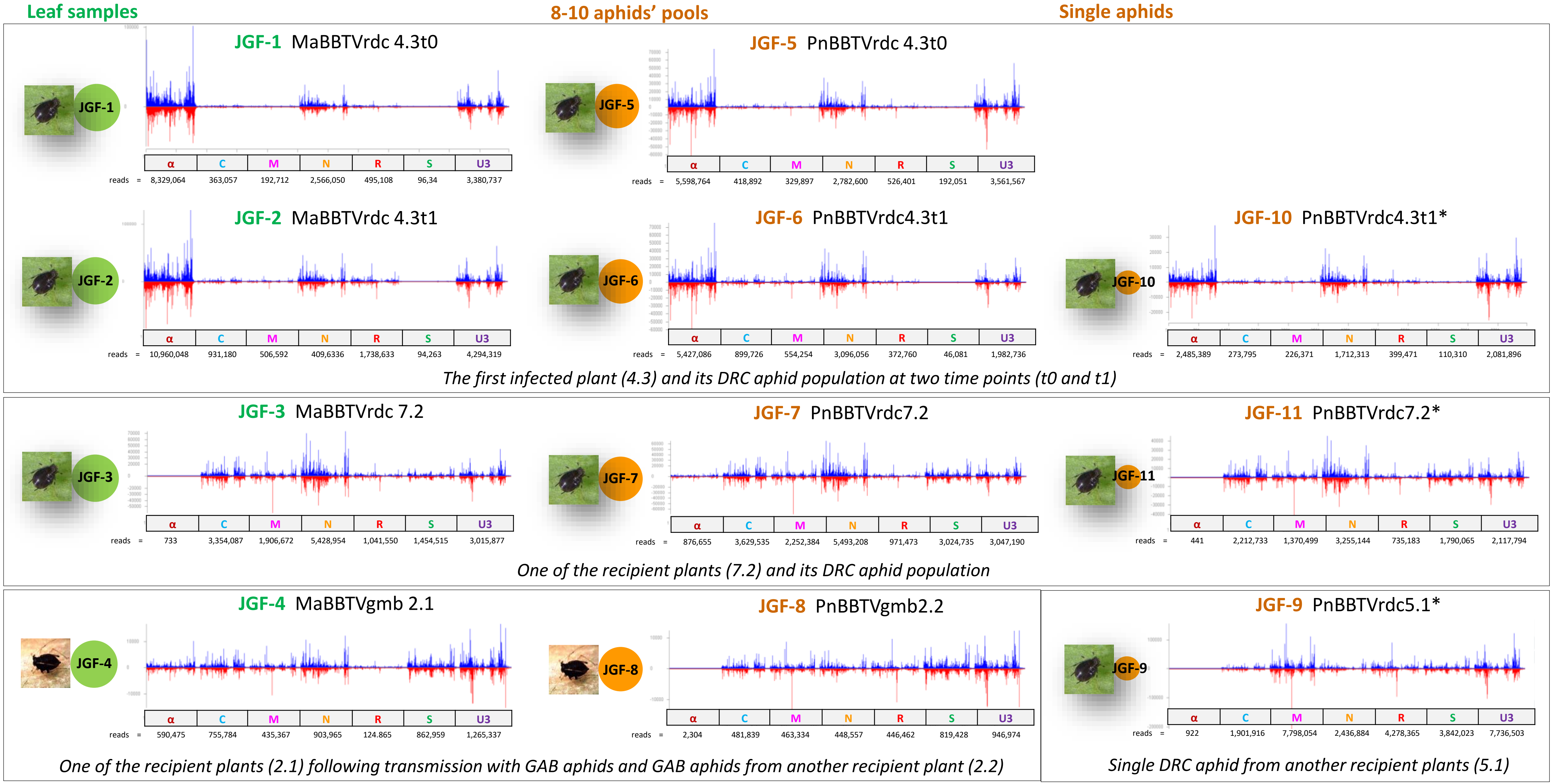

S4 Fig. Maps and relative abundance of Illumina sequencing reads representing RCA-amplified DNA of BBTV genome components and alphasatellite identified in BBTD-infected Cavendish plants and aphids taken from these plants. For each of the 11 samples (JGF-1-11, see Supplementary Figure S2) of BBTD-infected plant leaf tissues and viruliferous aphids (indicated with green and orange circles, respectively), the Illumina 125 nt reads were mapped on concatenated sequences of alphasatellite (α) and six BBTV components (C, M, N, R, S, U3) and the mapped reads were visualized using MISIS-2 (Seguin et al. 2016) and counted. Histograms plot the numbers of viral 125 nt forward and reverse reads at each nucleotide position of the concatenated viral genome: blue bars above the axis represent forward reads ending at each respective position red bars below the axis represent reverse reads ending at each respective position. Numbers of reads mapped to each viral genome component are given below the histograms.
