# Supplementary material for: A newly emerging alphasatellite affects banana bunchy top virus replication, transcription, siRNA production and transmission by aphids
Source: PLoS Pathog. 2022 Apr 12;18(4):e1010448. doi: 10.1371/journal.ppat.1010448 (PMC9049520; doi:10.1371/journal.ppat.1010448)
Supplement: S5 Fig — (A) Schematic representation of circular BBTV genome components (C, M, N, R, S, U3) and alphasatellite with position of diagnostic PCR primers (yellow arrows). (B) Multiplex, duplex and single PCR analysis of the 11 samples (JGF-1-11, see S2 Fig) of BBTD-infected plant leaf tissues and viruliferous aphids (indicated with green and orange circles, respectively). PCR products were separated on 2% agarose gel. Positions of each BBTV component and alphasatellite are indicated by arrows. (PDF) [file ppat.1010448.s006.pdf]

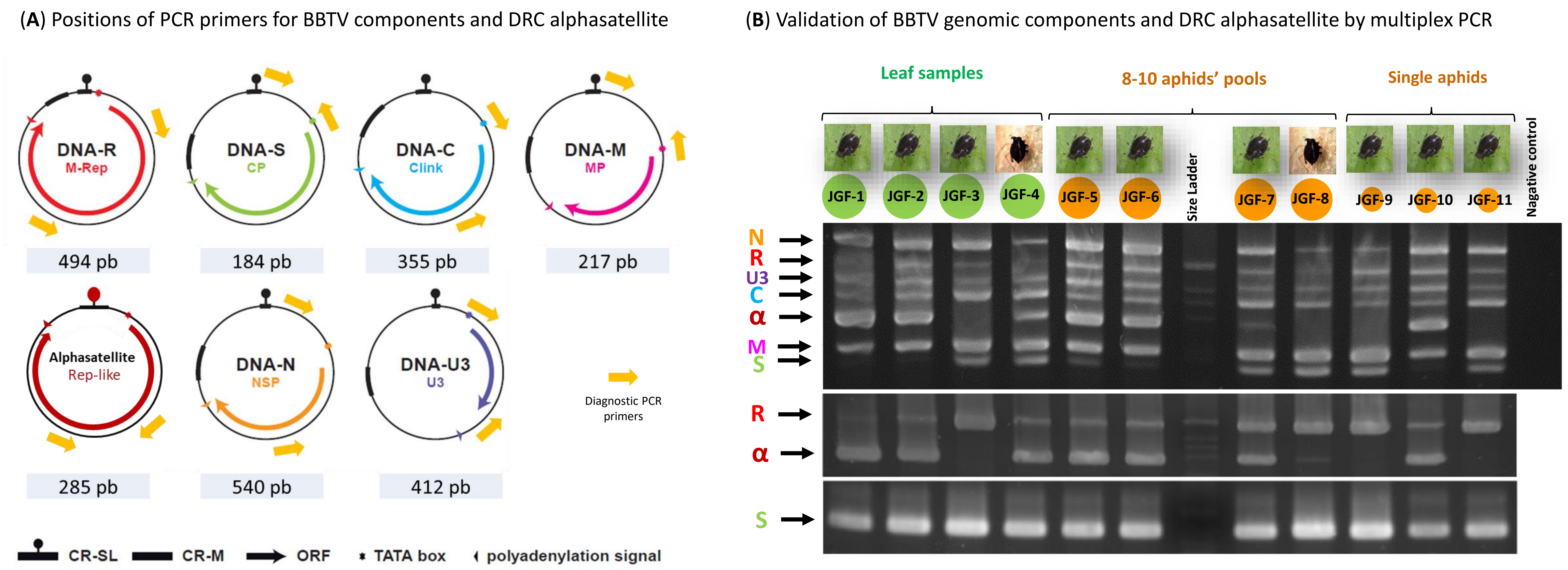

S5 Fig. Multiplex, duplex and single PCR validation of the BBTV genome components and DRC alphasatellite reconstructed by Illumina sequencing of RCA-amplified viral DNA from BBTD-infected Cavendish plants and aphids taken from these plants. (**A**) Schematic representation of circular BBTV genome components (C, M, N, R, S, U3) and alphasatellite (α) with position of diagnostic PCR primers (yellow arrows). (**B**) Multiplex, duplex and single PCR analysis of the 11 samples (JGF-1-11, see Supplementary Figure S2) of BBTD-infected plant leaf tissues and viruliferous aphids (indicated with green and orange circles, respectively). PCR products were separated on 2% agarose gel. Positions of each BBTV component and alphasatellite are indicated by arrows.
