# Supplementary material for: A newly emerging alphasatellite affects banana bunchy top virus replication, transcription, siRNA production and transmission by aphids
Source: PLoS Pathog. 2022 Apr 12;18(4):e1010448. doi: 10.1371/journal.ppat.1010448 (PMC9049520; doi:10.1371/journal.ppat.1010448)
Supplement: S6 Fig — (A) PCR analysis of total DNA extracted from BBTV-infected plants from DRC (2012) using DRC-2016 alphasatellite-specific primers (S1 Table), followed by IC-PCR analysis of selected leaf samples. Position of the alphasatellite (α)-specific PCR product on each gel is indicated by an arrow. (B) Multiplex PCR analysis of RCA products of viral DNA from selected BBTV-infected plant samples from New Caledonia (NCL), Malawi (MLW), Benin (BEN), Gabon (GAB) and DRC using the primers for each BBTV component and DRC alphasatellite (S5 Fig) with positions of respective PCR products indicated by arrows. (C) Multiplex PCR analysis of RCA-amplified DNA of BBTV-infected plants from Togo (TGO), Benin (BEN) and Nigeria (NGA) (2019) using the primers for each BBTV component and DRC alphasatellite (S5 Fig) with positions of respective PCR products indicated by arrows, followed by single PCR analysis with degenerate PCR primers specific for DRC alphasatellite and all members of the genus Fabenesatellite (S1 Table). (D) Duplex and single PCR analysis of total DNA of BBTD-infected plants from Benin (BEN) (2020), using DRC- and Fabenesatellite-specific degenerate primers. Position of alphasatellite (α)-specific PCR product on each gel is indicated by an arrow. DRC alphasatellite-infected and healthy Cavendish plants were used as respectively positive “α (+)” and negative “α (-)” controls. Note that the PCR analysis of DNA samples from DRC revealed two alphasatellite-positive samples (368 and 381), while the follow-up IC-PCR of leaves confirmed the presence of encapsidated alphasatellite only in sample 381 (panel A), possibly due to cross-contamination during DNA extraction. Illumina sequencing of RCA amplified viral DNA (pre-analyzed by multiplex PCR, panel B) revealed that the DNA samples 368 and 381 contain identical sequences of the alphasatellite and six BBTV components. (PDF) [file ppat.1010448.s007.pdf]

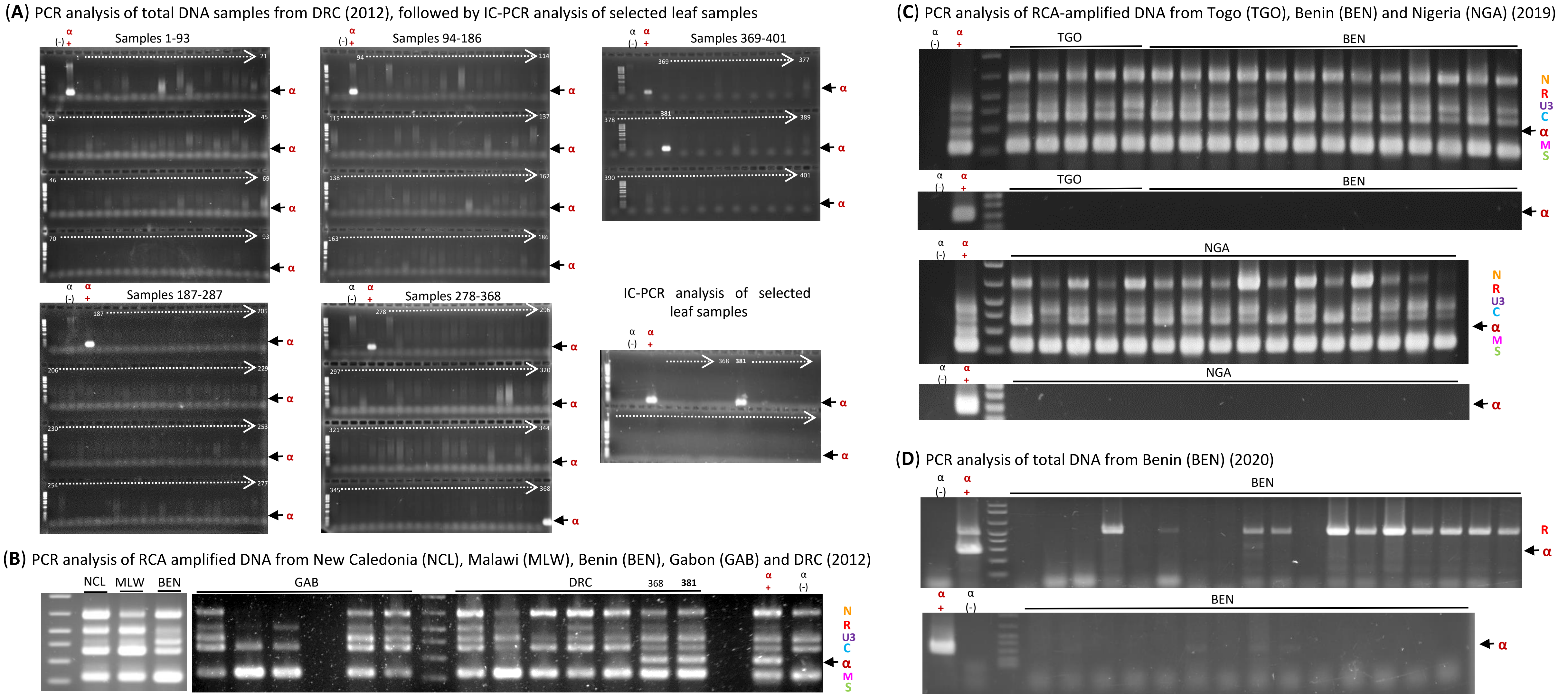

S6 Fig. PCR and immuno-capture (IC)-PCR analyses with alphasatellite-specific primers of banana plant samples from PIO (Sub-Saharan Africa and New Caledonia). (A) PCR analysis of total DNA extracted from BBTV-infected plants from DRC (2012) using DRC-2016 alphasatellite-specific primers (S1 Table), followed by IC-PCR analysis of selected leaf samples. Position of the alphasatellite (α)-specific PCR product on each gel is indicated by an arrow. **(B)** Multiplex PCR analysis of RCA products of viral DNA from selected BBTV-infected plant samples from New Caledonia (NCL), Malawi (MLW), Benin (BEN), Gabon (GAB) and DRC using the primers for each BBTV component and DRC alphasatellite (S5 Fig) with positions of respective PCR products indicated by arrows. **(C)** Multiplex PCR analysis of RCA-amplified DNA of BBTV-infected plants from Togo (TGO), Benin (BEN) and Nigeria (NGA) (2019) using the primers for each BBTV component and DRC alphasatellite (S5 Fig) with positions of respective PCR products indicated by arrows, followed by single PCR analysis with degenerate PCR primers specific for DRC alphasatellite and all members of the genus *Fabenesatellite* (S1 Table). **(D)** Duplex and single PCR analysis of total DNA of BBTD-infected plants from Benin (BEN) (2020), using DRC- and *Fabenesatellite*-specific degenerate primers. Position of alphasatellite (α)-specific PCR product on each gel is indicated by an arrow. DRC alphasatellite-infected and healthy Cavendish plants were used as respectively positive "α (+)" and negative "α (-)" controls. Note that the PCR analysis of DNA samples from DRC revealed two alphasatellite-positive samples (368 and 381), while the follow-up IC-PCR of leaves confirmed the presence of encapsidated alphasatellite only in sample 381 (panel **A**), possibly due to cross-contamination during DNA extraction. Illumina sequencing of RCA amplified viral DNA (pre-analysed by multiplex PCR, panel **B**) revealed that the DNA samples 368 and 381 contain identical sequences of the alphasatellite and six BBTV components.
