# Supplementary material for: A newly emerging alphasatellite affects banana bunchy top virus replication, transcription, siRNA production and transmission by aphids
Source: PLoS Pathog. 2022 Apr 12;18(4):e1010448. doi: 10.1371/journal.ppat.1010448 (PMC9049520; doi:10.1371/journal.ppat.1010448)
Supplement: S7 Fig — The nucleotide sequences of DRC alphasatellite isolates were compared with those of all alphasatellites from the subfamilies Petromoalphasatellitinae (A), Nanoalphasatellitinae (B) and Geminialphasatellitinae (C) available at the NCBI Genbank in September 2021 using Sequence Demarcation Tool (SDT) v1.2 [87] with Muscles (excluding indels) and their pairwise identities (in %) were plotted as heatmap diagrams and shown in Tables. Two isolates of DRC alphasatellite are indicated with red arrows. (PDF) [file ppat.1010448.s008.pdf]

S7 Fig. Pairwise sequence comparison of DRC alphasatellite isolates DRC-2016 (OK546211) and DRC-2012 (OK546212) with other alphasatellites. The nucleotide sequences of DRC alphasatellite isolates were compared with those of all alphasatellites from the subfamilies *Petromoalphasatellitinae* (**A**), *Nanoalphasatellitinae* (**B**) and *Geminialphasatellitinae* (**C**) available at the NCBI Genbank in September 2021 using Sequence Demarcation Tool (SDT) v1.2 with Muscles (excluding indels) (Muhire et al. 2014) and their pairwise identities (in %) were plotted as heatmap diagrams and shown in Tables. Two isolates of DRC alphasatellite are indicated with red arrows.

(A) DRC alphasatellite vs *Petromoalphasatellitinae*

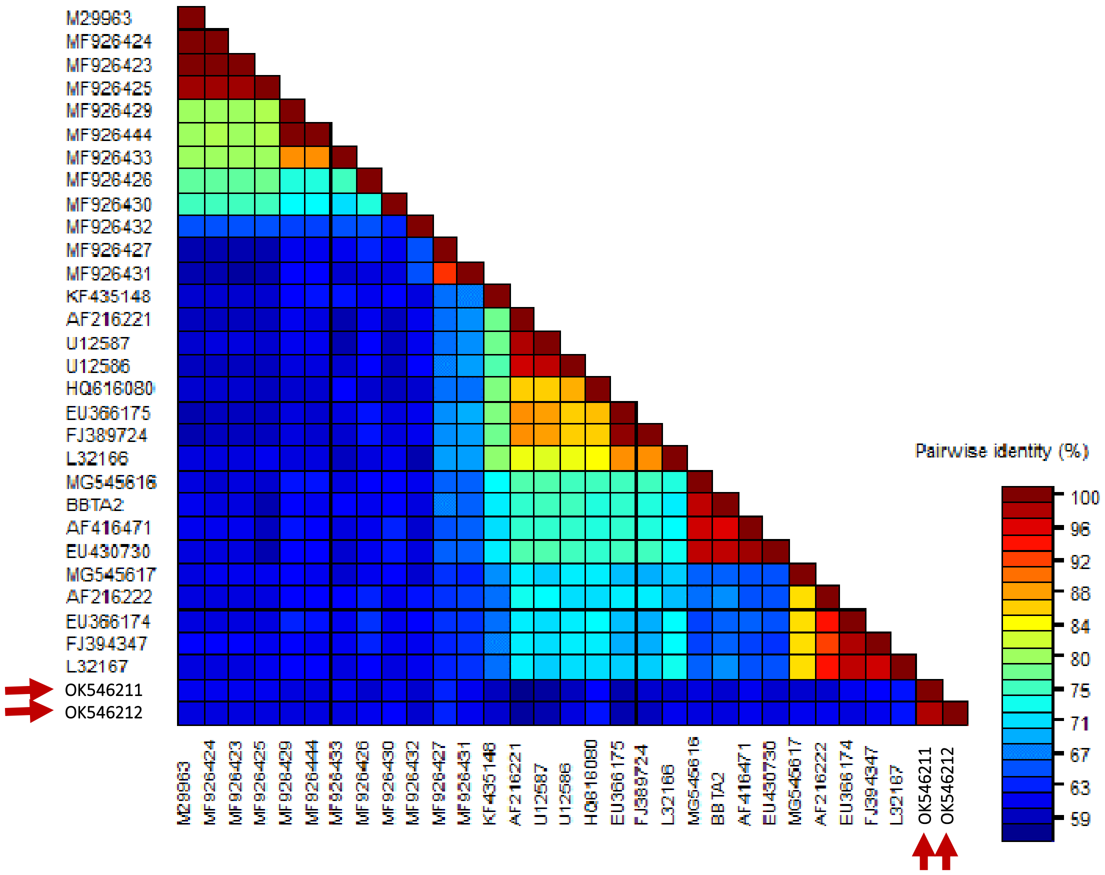

First Sequence	Second Sequenc	Identity Score	%
>OK546211	>OK546212	0,9808044	98,1
>M29963	>OK546211	0,6062917	60,6
>MF926424	>OK546211	0,607245	60,7
>MF926423	>OK546211	0,6081983	60,8
>MF926425	>OK546211	0,6051379	60,5
>MF926429	>OK546211	0,6021297	60,2
>MF926444	>OK546211	0,6011617	60,1
>MF926433	>OK546211	0,6052889	60,5
>MF926426	>OK546211	0,5960785	59,6
>MF926430	>OK546211	0,608365	60,8
>MF926432	>OK546211	0,5919153	59,2
>MF926427	>OK546211	0,6282306	62,8
>MF926431	>OK546211	0,6101861	61,0
>KF435148	>OK546211	0,5901961	59,0
>AF216221	>OK546211	0,5666667	56,7
>U12587	>OK546211	0,5718446	57,2
>U12586	>OK546211	0,5852216	58,5
>HQ616080	>OK546211	0,6168699	61,7
>EU366175	>OK546211	0,5829195	58,3
>FJ389724	>OK546211	0,5912263	59,1
>L32166	>OK546211	0,5916334	59,2
>MG545616	>OK546211	0,5994036	59,9
>BBTA2	>OK546211	0,5974155	59,7
>AF416471	>OK546211	0,606	60,6
>EU430730	>OK546211	0,5954725	59,5
>MG545617	>OK546211	0,5918368	59,2
>AF216222	>OK546211	0,5974283	59,7
>EU366174	>OK546211	0,6072508	60,7
>FJ394347	>OK546211	0,6141249	61,4
>L32167	>OK546211	0,622449	62,2

First Sequence	Second Sequenc	Identity Score	%
>M29963	>OK546212	0,5977121	59,8
>MF926424	>OK546212	0,5986654	59,9
>MF926423	>OK546212	0,5977121	59,8
>MF926425	>OK546212	0,5996187	60,0
>MF926429	>OK546212	0,6021093	60,2
>MF926444	>OK546212	0,6011505	60,1
>MF926433	>OK546212	0,6036822	60,4
>MF926426	>OK546212	0,6045142	60,5
>MF926430	>OK546212	0,611379	61,1
>MF926432	>OK546212	0,5917387	59,2
>MF926427	>OK546212	0,6257485	62,6
>MF926431	>OK546212	0,6056752	60,6
>KF435148	>OK546212	0,5970297	59,7
>AF216221	>OK546212	0,5735294	57,4
>U12587	>OK546212	0,582762	58,3
>U12586	>OK546212	0,5998004	60,0
>HQ616080	>OK546212	0,6223132	62,2
>EU366175	>OK546212	0,5837513	58,4
>FJ389724	>OK546212	0,5903614	59,0
>L32166	>OK546212	0,6053719	60,5
>MG545616	>OK546212	0,603	60,3
>BBTA2	>OK546212	0,6037924	60,4
>AF416471	>OK546212	0,6087399	60,9
>EU430730	>OK546212	0,602	60,2
>MG545617	>OK546212	0,605315	60,5
>AF216222	>OK546212	0,6059063	60,6
>EU366174	>OK546212	0,6028226	60,3
>FJ394347	>OK546212	0,6096311	61,0
>L32167	>OK546212	0,6193416	61,9
>OK546211	>OK546212	0,9808044	98,1

(B) DRC alphasatellite vs *Nanoalphasatellitinae*

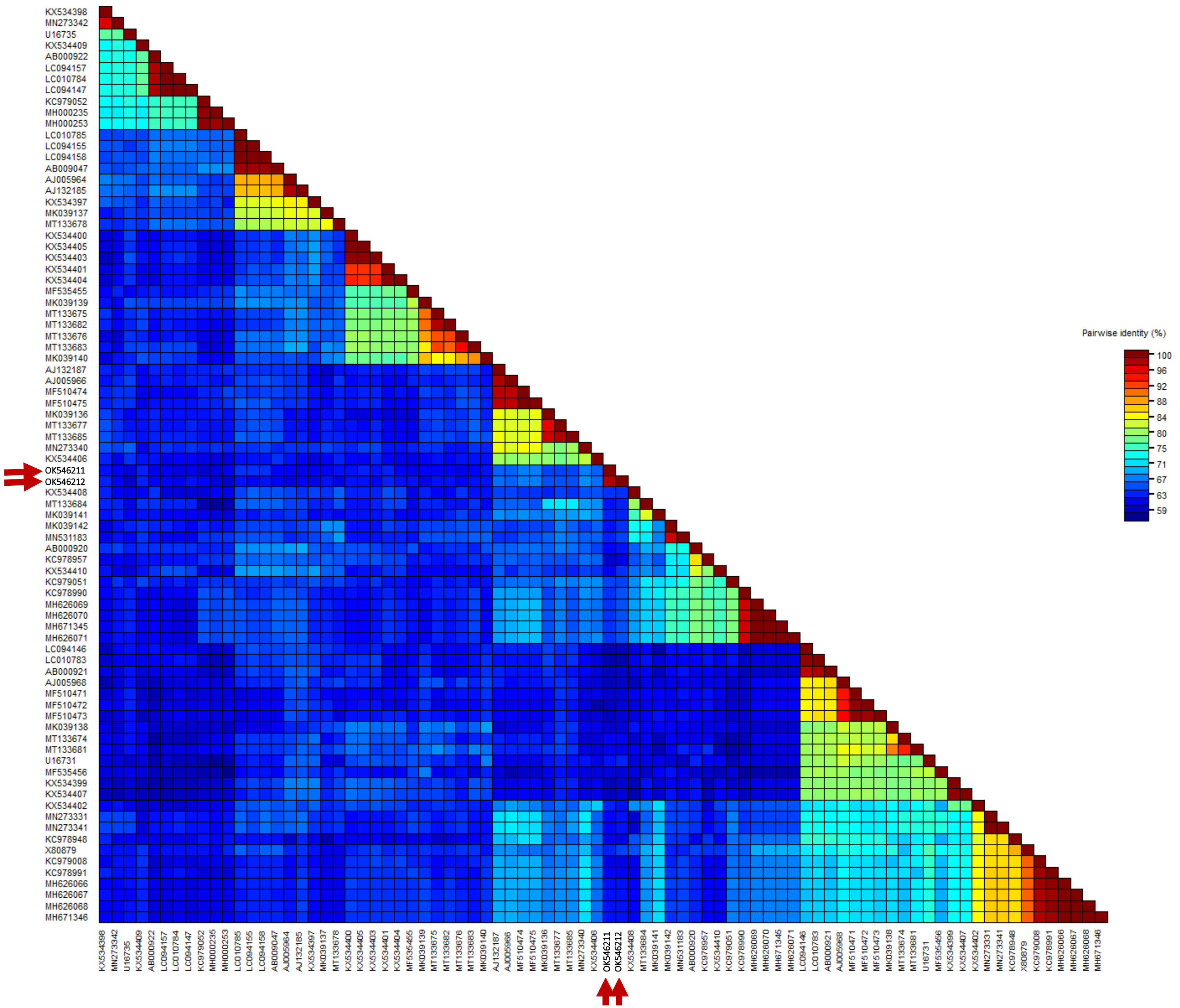

First Sequence	Second Sequence	Identity Score	%
>OK546211	>OK546212	0,9808044	98,1
>OK546211	>KX534408	0,6354057	63,5
>OK546211	>MT133684	0,6182396	61,8
>OK546211	>MK039141	0,629118	62,9
>OK546211	>MK039142	0,6244541	62,4
>OK546211	>MN531183	0,6310044	63,1
>OK546211	>AB000920	0,6130064	61,3
>OK546211	>KC978957	0,5964546	59,6
>OK546211	>KX534410	0,6139785	61,4
>OK546211	>KC979051	0,6427819	64,3
>OK546211	>KC978990	0,6343042	63,4
>OK546211	>MH626069	0,6346983	63,5
>OK546211	>MH626070	0,6346983	63,5
>OK546211	>MH671345	0,6346983	63,5
>OK546211	>MH626071	0,6357759	63,6
>OK546211	>LC094146	0,5835095	58,4
>OK546211	>LC010783	0,5819328	58,2
>OK546211	>AB000921	0,5891719	58,9
>OK546211	>AJ005968	0,5856531	58,6
>OK546211	>MF510471	0,590958	59,1
>OK546211	>MF510472	0,588172	58,8
>OK546211	>MF510473	0,5869099	58,7
>OK546211	>MK039138	0,5935484	59,4
>OK546211	>MT133674	0,6163934	61,6
>OK546211	>MT133681	0,6086487	60,9
>OK546211	>U16731	0,6237732	62,4
>OK546211	>MF535456	0,6010363	60,1
>OK546211	>KX534399	0,5897436	59,0
>OK546211	>KX534407	0,5867944	58,7
>OK546211	>KX534402	0,6260504	62,6
>OK546211	>MN273331	0,6137787	61,4
>OK546211	>MN273341	0,6137787	61,4
>OK546211	>KC978948	0,604888	60,5
>OK546211	>X80879	0,6302966	63,0
>OK546211	>KC979008	0,6108786	61,1
>OK546211	>KC978991	0,6145833	61,5
>OK546211	>MH626066	0,6208333	62,1
>OK546211	>MH626067	0,6239583	62,4
>OK546211	>MH626068	0,6239583	62,4
>OK546211	>MH671346	0,6229166	62,3
>KX534398	>OK546211	0,621421	62,1
>MN273342	>OK546211	0,6132771	61,3
>U16735	>OK546211	0,5987526	59,9
>KX534409	>OK546211	0,6293104	62,9
>AB000922	>OK546211	0,6066946	60,7
>LC094157	>OK546211	0,6056485	60,6
>LC010784	>OK546211	0,5995829	60,0
>LC094147	>OK546211	0,6108786	61,1
>KC979052	>OK546211	0,5968254	59,7
>MH000235	>OK546211	0,6027689	60,3
>MH000253	>OK546211	0,6151351	61,5
>LC010785	>OK546211	0,6344969	63,4
>LC094155	>OK546211	0,6382323	63,8
>LC094158	>OK546211	0,6355236	63,6
>AB009047	>OK546211	0,617558	61,8
>AJ005964	>OK546211	0,6165803	61,7
>AJ132185	>OK546211	0,6103093	61,0
>KX534397	>OK546211	0,6072508	60,7
>MK039137	>OK546211	0,6148069	61,5
>MT133678	>OK546211	0,6243272	62,4
>KX534400	>OK546211	0,6207265	62,1
>KX534405	>OK546211	0,6255319	62,6
>KX534403	>OK546211	0,6193001	61,9
>KX534401	>OK546211	0,6255274	62,6
>KX534404	>OK546211	0,6161935	61,6
>MF535455	>OK546211	0,6107106	61,1
>MK039139	>OK546211	0,6144958	61,4
>MT133675	>OK546211	0,6111696	61,1
>MT133682	>OK546211	0,6115789	61,2
>MT133676	>OK546211	0,6060606	60,6
>MT133683	>OK546211	0,6119874	61,2
>MK039140	>OK546211	0,6218221	62,2
>AJ132187	>OK546211	0,6556225	65,6
>AJ005966	>OK546211	0,6545821	65,5
>MF510474	>OK546211	0,6697062	67,0
>MF510475	>OK546211	0,668693	66,9
>MK039136	>OK546211	0,6404495	64,0
>MT133677	>OK546211	0,6445352	64,5
>MT133685	>OK546211	0,6424924	64,2
>MN273340	>OK546211	0,6720812	67,2
>KX534406	>OK546211	0,6569417	65,7

Fabenesatellite (last nine rows)

>OK546212	>KX534408	0,6348195	63,5
>OK546212	>MT133684	0,6280814	62,8
>OK546212	>MK039141	0,6280814	62,8
>OK546212	>MK039142	0,6354626	63,5
>OK546212	>MN531183	0,644839	64,5
>OK546212	>AB000920	0,6192017	61,9
>OK546212	>KC978957	0,6078224	60,8
>OK546212	>KX534410	0,6335878	63,4
>OK546212	>KC979051	0,642328	64,2
>OK546212	>KC978990	0,6484716	64,8
>OK546212	>MH626069	0,6474429	64,7
>OK546212	>MH626070	0,6474429	64,7
>OK546212	>MH671345	0,6474429	64,7
>OK546212	>MH626071	0,648531	64,9
>OK546212	>LC094146	0,5858369	58,6
>OK546212	>LC010783	0,5774947	57,7
>OK546212	>AB000921	0,5933477	59,3
>OK546212	>AJ005968	0,5932755	59,3
>OK546212	>MF510471	0,6032787	60,3
>OK546212	>MF510472	0,6037118	60,4
>OK546212	>MF510473	0,6002179	60,0
>OK546212	>MK039138	0,5989071	59,9
>OK546212	>MT133674	0,6135105	61,4
>OK546212	>MT133681	0,6105611	61,1
>OK546212	>U16731	0,6273429	62,7
>OK546212	>MF535456	0,6014799	60,1
>OK546212	>KX534399	0,5982628	59,8
>OK546212	>KX534407	0,5982628	59,8
>OK546212	>KX534402	0,6244681	62,4
>OK546212	>MN273331	0,6240602	62,4
>OK546212	>MN273341	0,6240602	62,4
>OK546212	>KC978948	0,606405	60,6
>OK546212	>X80879	0,6430868	64,3
>OK546212	>KC979008	0,6092925	60,9
>OK546212	>KC978991	0,6162791	61,6
>OK546212	>MH626066	0,6226215	62,3
>OK546212	>MH626067	0,6205074	62,1
>OK546212	>MH626068	0,6205074	62,1
>OK546212	>MH671346	0,6194503	61,9
>KX534398	>OK546212	0,6252654	62,5
>MN273342	>OK546212	0,6162791	61,6
>U16735	>OK546212	0,5914316	59,1
>KX534409	>OK546212	0,6293104	62,9
>AB000922	>OK546212	0,6114044	61,1
>LC094157	>OK546212	0,6166843	61,7
>LC010784	>OK546212	0,608421	60,8
>LC094147	>OK546212	0,6198522	62,0
>KC979052	>OK546212	0,6153846	61,5
>MH000235	>OK546212	0,6034115	60,3
>MH000253	>OK546212	0,5955414	59,6
>LC010785	>OK546212	0,6206544	62,1
>LC094155	>OK546212	0,616172	61,6
>LC094158	>OK546212	0,6216769	62,2
>AB009047	>OK546212	0,6146789	61,5
>AJ005964	>OK546212	0,6006192	60,1
>AJ132185	>OK546212	0,6118627	61,2
>KX534397	>OK546212	0,6087845	60,9
>MK039137	>OK546212	0,6182213	61,8
>MT133678	>OK546212	0,6092077	60,9
>KX534400	>OK546212	0,6205788	62,1
>KX534405	>OK546212	0,6215352	62,2
>KX534403	>OK546212	0,6170213	61,7
>KX534401	>OK546212	0,6315218	63,2
>KX534404	>OK546212	0,6208017	62,1
>MF535455	>OK546212	0,6209424	62,1
>MK039139	>OK546212	0,6086956	60,9
>MT133675	>OK546212	0,6181818	61,8
>MT133682	>OK546212	0,6216506	62,2
>MT133676	>OK546212	0,6136606	61,4
>MT133683	>OK546212	0,6227224	62,3
>MK039140	>OK546212	0,6196581	62,0
>AJ132187	>OK546212	0,659919	66,0
>AJ005966	>OK546212	0,6568528	65,7
>MF510474	>OK546212	0,6703854	67,0
>MF510475	>OK546212	0,6713996	67,1
>MK039136	>OK546212	0,6456612	64,6
>MT133677	>OK546212	0,6518595	65,2
>MT133685	>OK546212	0,6487603	64,9
>MN273340	>OK546212	0,6765914	67,7
>KX534406	>OK546212	0,6602434	66,0
>OK546211	>OK546212	0,9808044	98,1

Fabenesatellite (rows AJ132187 to KX534406)

(C) DRC alphasatellite vs *Geminialphasatellitinae*

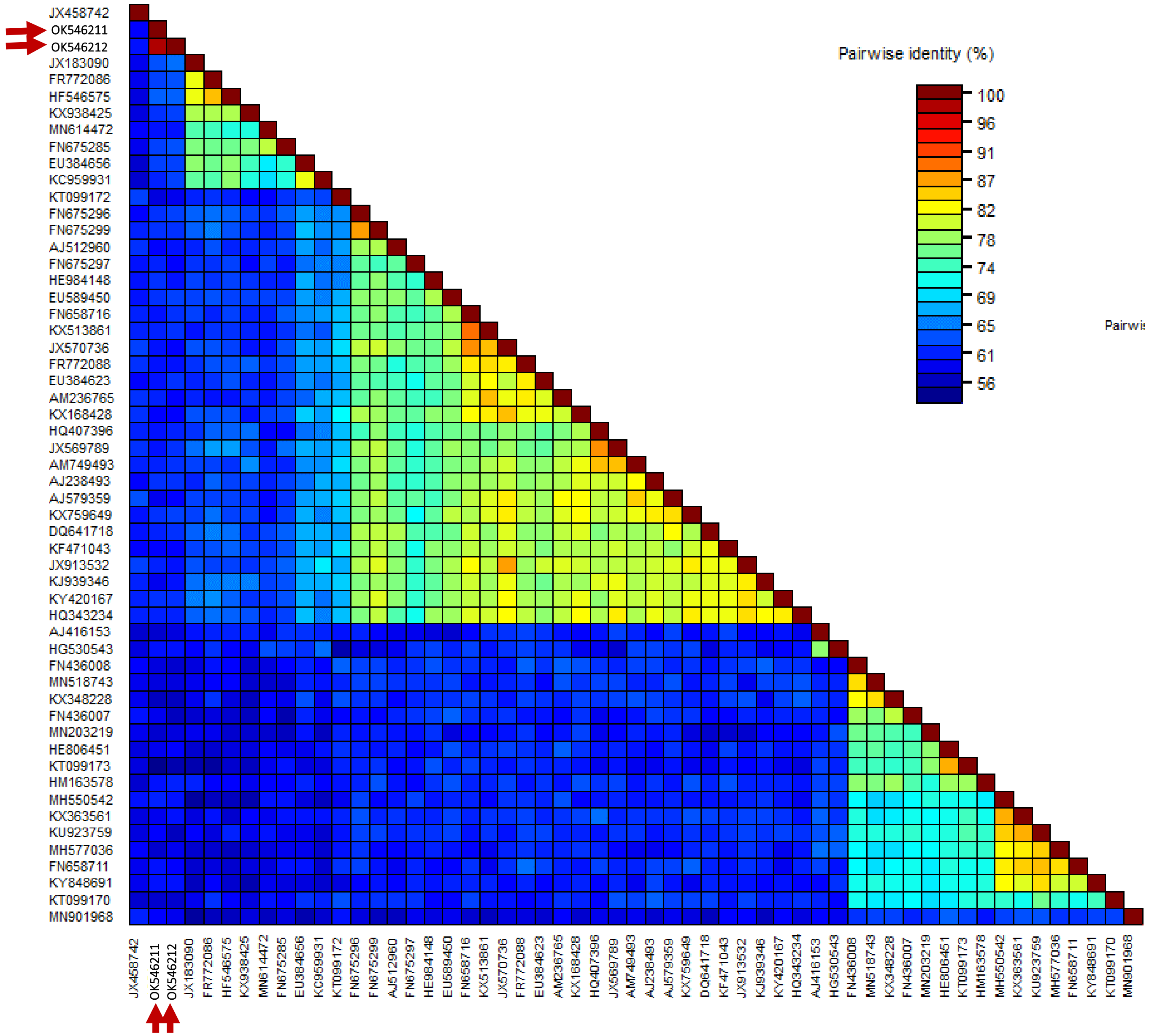

Pairwis

First Sequence	Second Sequence	Identity Score	%
>OK546211	>OK546212	0,9808044	98,08044
>OK546211	>JX183090	0,6316271	63,16271
>OK546211	>FR772086	0,6223648	62,23648
>OK546211	>HF546575	0,6363636	63,63636
>OK546211	>KX938425	0,6195256	61,95256
>OK546211	>MN614472	0,601476	60,1476
>OK546211	>FN675285	0,6203704	62,03704
>OK546211	>EU384656	0,6270066	62,70066
>OK546211	>KC959931	0,6113657	61,13657
>OK546211	>KT099172	0,5786948	57,86948
>OK546211	>FN675296	0,6190926	61,90926
>OK546211	>FN675299	0,614435	61,4435
>OK546211	>AJ512960	0,5943396	59,43396
>OK546211	>FN675297	0,6094339	60,94339
>OK546211	>HE984148	0,6146527	61,46527
>OK546211	>EU589450	0,6185958	61,85958
>OK546211	>FN658716	0,6037209	60,37209
>OK546211	>KX513861	0,6067415	60,67415
>OK546211	>JX570736	0,6043233	60,43233
>OK546211	>FR772088	0,6009259	60,09259
>OK546211	>EU384623	0,6027778	60,27778
>OK546211	>AM236765	0,6071094	60,71094
>OK546211	>KX168428	0,5917603	59,17603
>OK546211	>HQ407396	0,6027907	60,27907
>OK546211	>JX569789	0,6050186	60,50186
>OK546211	>AM749493	0,6074767	60,74767
>OK546211	>AJ238493	0,6199616	61,99616
>OK546211	>AJ579359	0,5901943	59,01943
>OK546211	>KX759649	0,6168401	61,68401
>OK546211	>DQ641718	0,606403	60,6403
>OK546211	>KF471043	0,5983225	59,83225
>OK546211	>JX913532	0,6093156	60,93156
>OK546211	>KJ939346	0,5847145	58,47145
>OK546211	>KY420167	0,6133584	61,33584
>OK546211	>HQ343234	0,6098239	60,98239
>OK546211	>AJ416153	0,571161	57,1161
>OK546211	>HG530543	0,6007569	60,07569
>OK546211	>FN436008	0,5798479	57,98479
>OK546211	>MN518743	0,5775862	57,75862
>OK546211	>KX348228	0,5667627	56,67627
>OK546211	>FN436007	0,5877712	58,77712
>OK546211	>MN203219	0,5744076	57,44076
>OK546211	>HE806451	0,5907336	59,07336
>OK546211	>KT099173	0,5442561	54,42561
>OK546211	>HM163578	0,6047198	60,47198
>OK546211	>MH550542	0,6075581	60,75581
>OK546211	>KX363561	0,5883477	58,83477
>OK546211	>KU923759	0,5977482	59,77482
>OK546211	>MH577036	0,573501	57,3501
>OK546211	>FN658711	0,5990431	59,90431
>OK546211	>KY848691	0,603235	60,3235
>OK546211	>KT099170	0,5734733	57,34733
>OK546211	>MN901968	0,5923445	59,23445
>JX458742	>OK546211	0,5925581	59,25581

First Sequence	Second Sequence	Identity Score	%
>OK546212	>JX183090	0,6436567	64,36567
>OK546212	>FR772086	0,6315789	63,15789
>OK546212	>HF546575	0,6418605	64,18605
>OK546212	>KX938425	0,624198	62,4198
>OK546212	>MN614472	0,6053604	60,53604
>OK546212	>FN675285	0,6283847	62,83847
>OK546212	>EU384656	0,6226591	62,26591
>OK546212	>KC959931	0,6211982	62,11982
>OK546212	>KT099172	0,5896686	58,96686
>OK546212	>FN675296	0,6203966	62,03966
>OK546212	>FN675299	0,620155	62,0155
>OK546212	>AJ512960	0,6005639	60,05639
>OK546212	>FN675297	0,595884	59,5884
>OK546212	>HE984148	0,6026365	60,26365
>OK546212	>EU589450	0,6238095	62,38095
>OK546212	>FN658716	0,6145251	61,45251
>OK546212	>KX513861	0,6170412	61,70412
>OK546212	>JX570736	0,597561	59,7561
>OK546212	>FR772088	0,5998143	59,98143
>OK546212	>EU384623	0,6158878	61,58878
>OK546212	>AM236765	0,5981482	59,81482
>OK546212	>KX168428	0,5918944	59,18944
>OK546212	>HQ407396	0,610438	61,0438
>OK546212	>JX569789	0,6155285	61,55285
>OK546212	>AM749493	0,6156752	61,56752
>OK546212	>AJ238493	0,6137866	61,37866
>OK546212	>AJ579359	0,592215	59,2215
>OK546212	>KX759649	0,623216	62,3216
>OK546212	>DQ641718	0,6151659	61,51659
>OK546212	>KF471043	0,5981221	59,81221
>OK546212	>JX913532	0,6049383	60,49383
>OK546212	>KJ939346	0,6013011	60,13011
>OK546212	>KY420167	0,6141509	61,41509
>OK546212	>HQ343234	0,6110591	61,10591
>OK546212	>AJ416153	0,5785513	57,85513
>OK546212	>HG530543	0,6017192	60,17192
>OK546212	>FN436008	0,5745489	57,45489
>OK546212	>MN518743	0,5815534	58,15534
>OK546212	>KX348228	0,5633397	56,33397
>OK546212	>FN436007	0,5628572	56,28572
>OK546212	>MN203219	0,5797517	57,97517
>OK546212	>HE806451	0,5957447	59,57447
>OK546212	>KT099173	0,5570342	55,70342
>OK546212	>HM163578	0,6092978	60,92978
>OK546212	>MH550542	0,6038835	60,38835
>OK546212	>KX363561	0,6028985	60,28985
>OK546212	>KU923759	0,5688623	56,88623
>OK546212	>MH577036	0,5860735	58,60735
>OK546212	>FN658711	0,5929119	59,29119
>OK546212	>KY848691	0,6050339	60,50339
>OK546212	>KT099170	0,5764023	57,64023
>OK546212	>MN901968	0,5846007	58,46007
>JX458742	>OK546212	0,6052142	60,52142
>OK546211	>OK546212	0,9808044	98,08044
