# Supplementary material for: A newly emerging alphasatellite affects banana bunchy top virus replication, transcription, siRNA production and transmission by aphids
Source: PLoS Pathog. 2022 Apr 12;18(4):e1010448. doi: 10.1371/journal.ppat.1010448 (PMC9049520; doi:10.1371/journal.ppat.1010448)
Supplement: S8 Fig — Rep proteins of the alphasatellites classified by Briddon et al. [41] (see Table 1 of that paper for the NCBI accession numbers) were compared “all-against-all” and clustered using CLANS [88]. DRC alphasatellite (DRC alpha) is indicated with a bright green cube close to the middle and its evolutionary relatedness (connection) to other alphasatellites is shown with solid grey lines whose color intensity—from lightest to darkest—indicates the strength of connections from worse (no direct connection) to best. Each alphasatellite genus is color-coded and named. Grey circles indicate unassigned alphasatellites. Note that the genus Babusatellite comprising four species (indicated with black diamonds) was recently split into two genera: Babusatellite and Muscarsatellite. (PDF) [file ppat.1010448.s009.pdf]

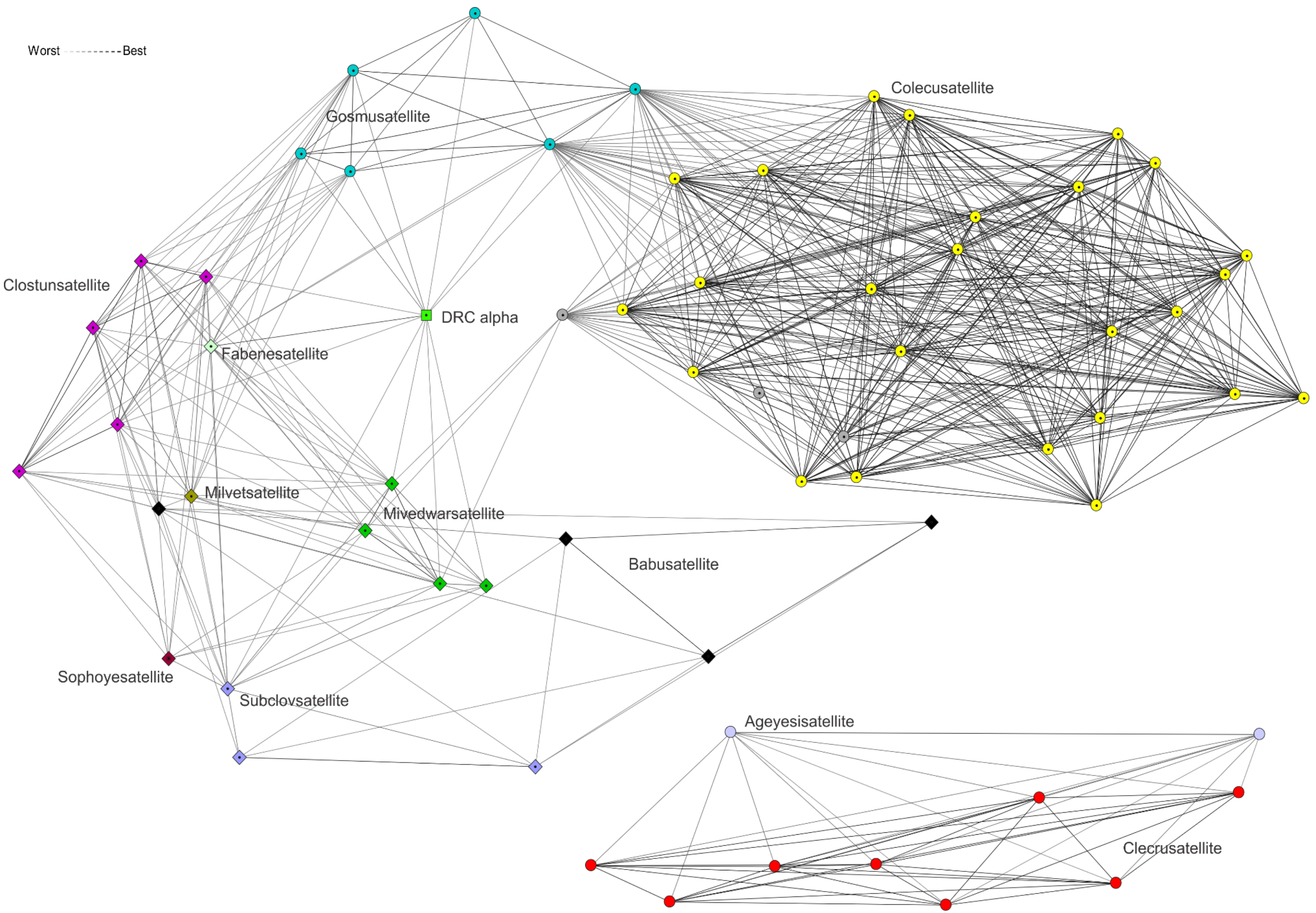

S8 Fig. Clustering analysis of Rep proteins encoded by DRC-2016 alphasatellite and alphasatellites associated with ssDNA viruses of the families *Nanoviridae, Metaxiviridae* and *Geminiviridae*. Rep proteins of the alphasatellites classified by Briddon et al. (2018) (see Table 1 of that paper for the NCBI accession numbers) were compared "all-against-all" and clustered using CLANS (Frickey and Lupas 2004). DRC alphasatellite (DRC alpha) is indicated with a bright green cube close to the middle and its evolutionary relatedness (connection) to other alphasatellites is shown with solid grey lines whose color intensity - from lightest to darkest - indicates the strength of connections from worse (no direct connection) to best. Each alphasatellite genus is color-coded and named. Grey circles indicate unassigned alphasatellites. Note that the genus *Babusatellite* comprising four species (indicated with black diamonds) was recently split into two genera: *Babusatellite* and *Muscarsatellite.*
