# Supplementary material for: A newly emerging alphasatellite affects banana bunchy top virus replication, transcription, siRNA production and transmission by aphids
Source: PLoS Pathog. 2022 Apr 12;18(4):e1010448. doi: 10.1371/journal.ppat.1010448 (PMC9049520; doi:10.1371/journal.ppat.1010448)
Supplement: S9 Fig — The complete sequences of BBTV components and alphasatellite from the DRC-2016 aphids (sample JGF-5, S2 Fig) were cloned as partial (DNAs C, M_v1, N, S_v1, U3_v1) or complete (DNA-R) dimers first in pBluescript SK(-) (A) and then in pCambia2300 (B). For each construct, positions of the duplicated common region stem loop (CR-SL) are indicated with red quadrates. (PDF) [file ppat.1010448.s010.pdf]

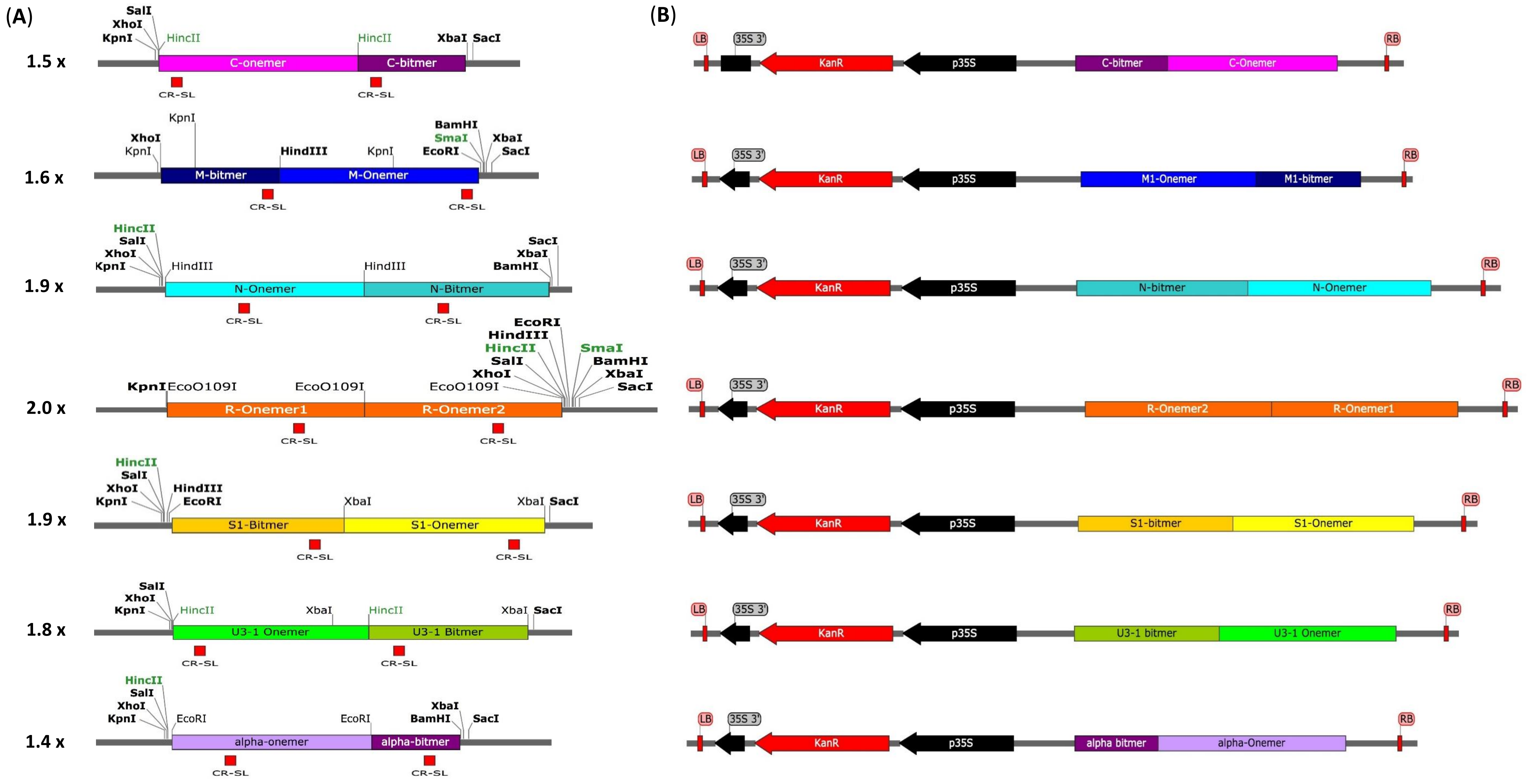

S9 Fig. **Construction of BBTV and alphasatellite infectious clones.** The complete sequences of BBTV components and alphasatellite from the DRC-2016 aphids (sample JGF-5, Supplementary Figure S2) were cloned as partial (DNAs C, M_v1, N, S_v1, U3_v1) or complete (DNA-R) dimers first in pBluescript SK(-) (**A**) and then in pCambia2300 (**B**). For each construct, positions of the duplicated common region stem loop (CR-SL) are indicated with red quadrates.
