# Supplementary material for: A newly emerging alphasatellite affects banana bunchy top virus replication, transcription, siRNA production and transmission by aphids
Source: PLoS Pathog. 2022 Apr 12;18(4):e1010448. doi: 10.1371/journal.ppat.1010448 (PMC9049520; doi:10.1371/journal.ppat.1010448)

S10 Fig. Analysis of BBTV and alphasatellite proteins for silencing suppression and chlorosis induction activities in *Nicotiana benthamiana*. (**A-B**) Subcloning of BBTV and alphasatellite ORFs from partial dimer constructs (**A**) (see Supplementary Figure S9) into the binary vector pB7WG2 under the control of cauliflower mosaic virus 35S promoter (35S) and terminator (35S 3') (**B**) by PCR amplification with viral ORF-specific primers carrying AttB1 and AttB2 recombination sites (Supplementary Table S1), followed by Gateway recombination of the PCR products into the vector. (**C-D**) Screening of BBTV and alphasatellite ORFs for silencing suppression and chlorosis induction following infiltration of the binary constructs in leaves of *N. benthamiana* GFP-transgenic (16c line) plants at 8 days post infiltration (dpi) under ultraviolet (UV) light (**C**) and day light (**D**). Pictures of representative leaves (from three infiltrated plants per construct, three leaves per plant) are shown. The experiment was repeated 3 times with similar results. Note that in addition to the BBTV Rep construct inducing strong chlorosis, the BBTV NSP construct induced weaker chlorosis, albeit it was barely visible in some of the infiltrated leaves.

(**A**) Positions of viral ORFs and PCR primers carrying AttB1 and AttB2 recombination sites

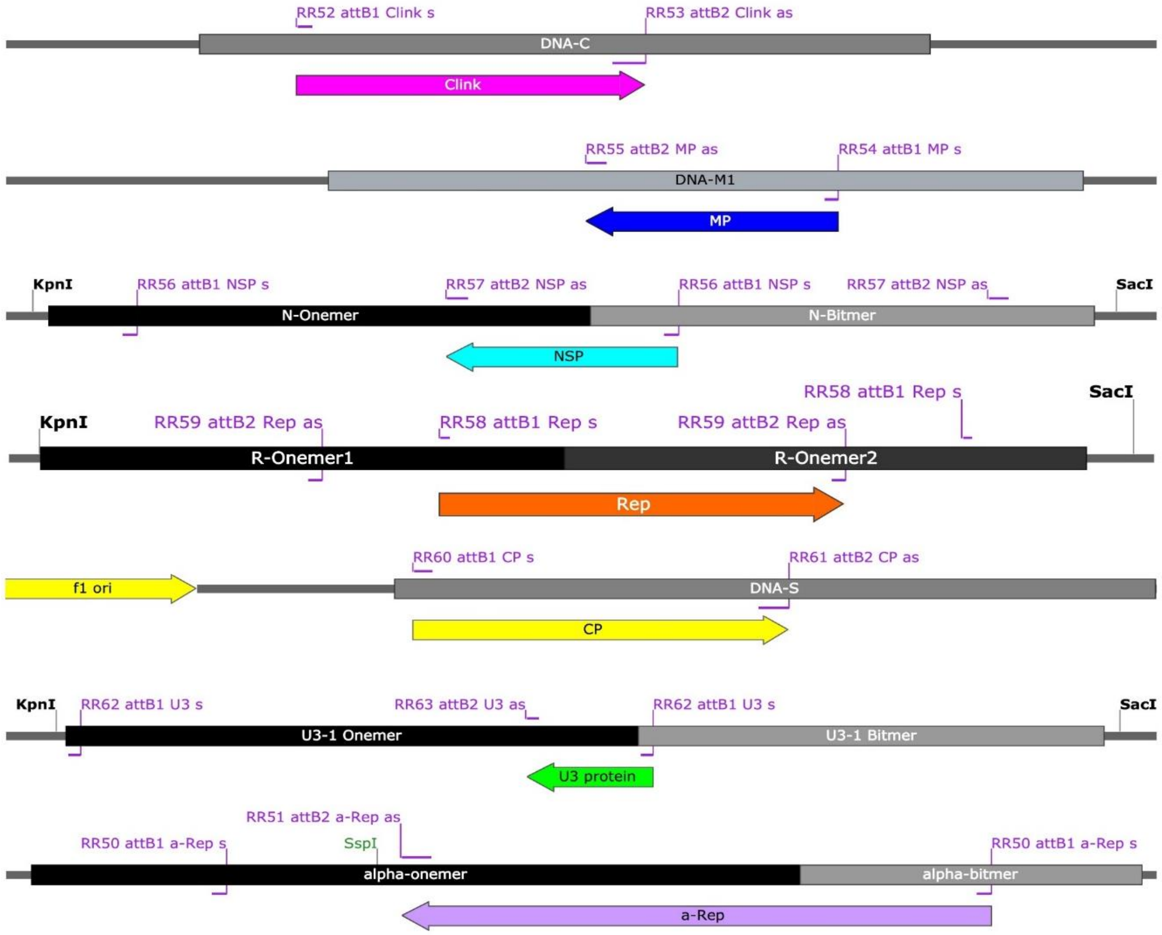

(**B**) Organization of viral ORF expression cassettes within T-DNA of the binary vector pB7WG2

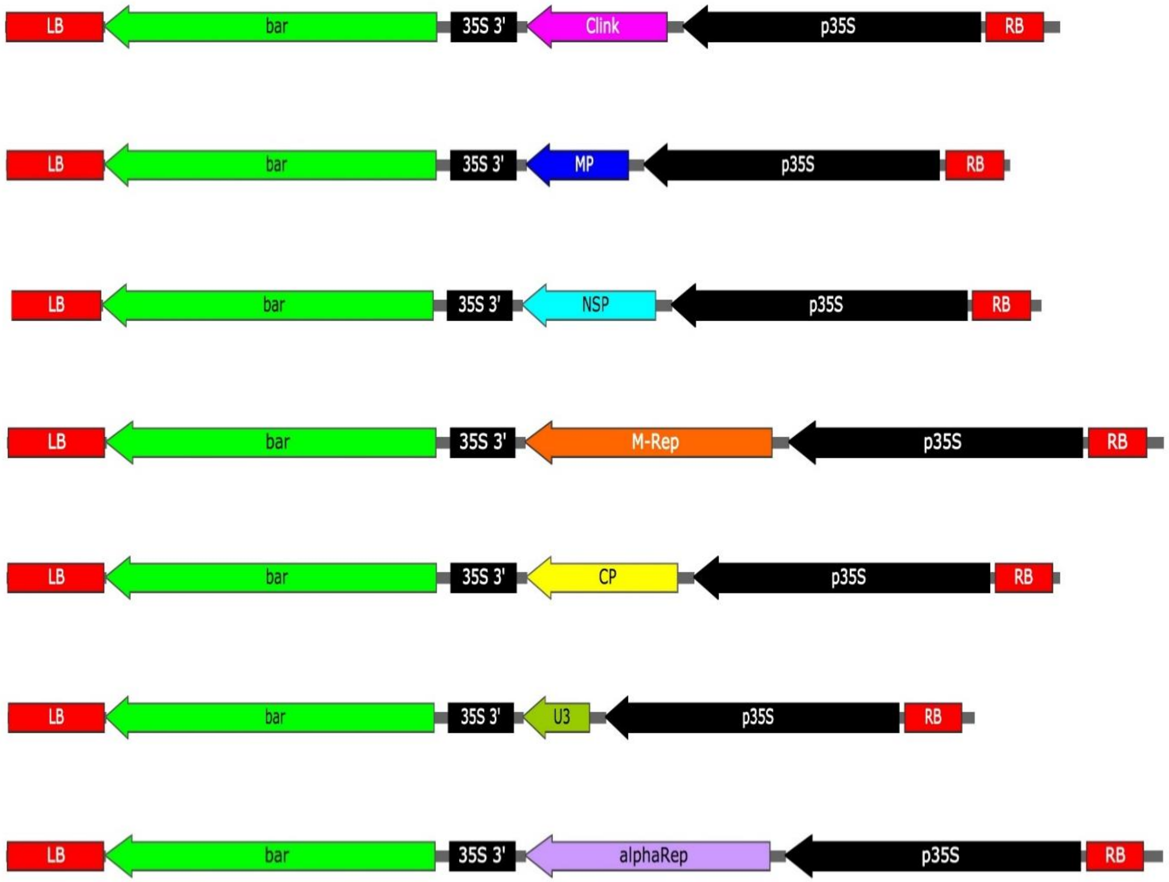

(**C**) Infiltrated leaves of *N. benthamiana* under UV light

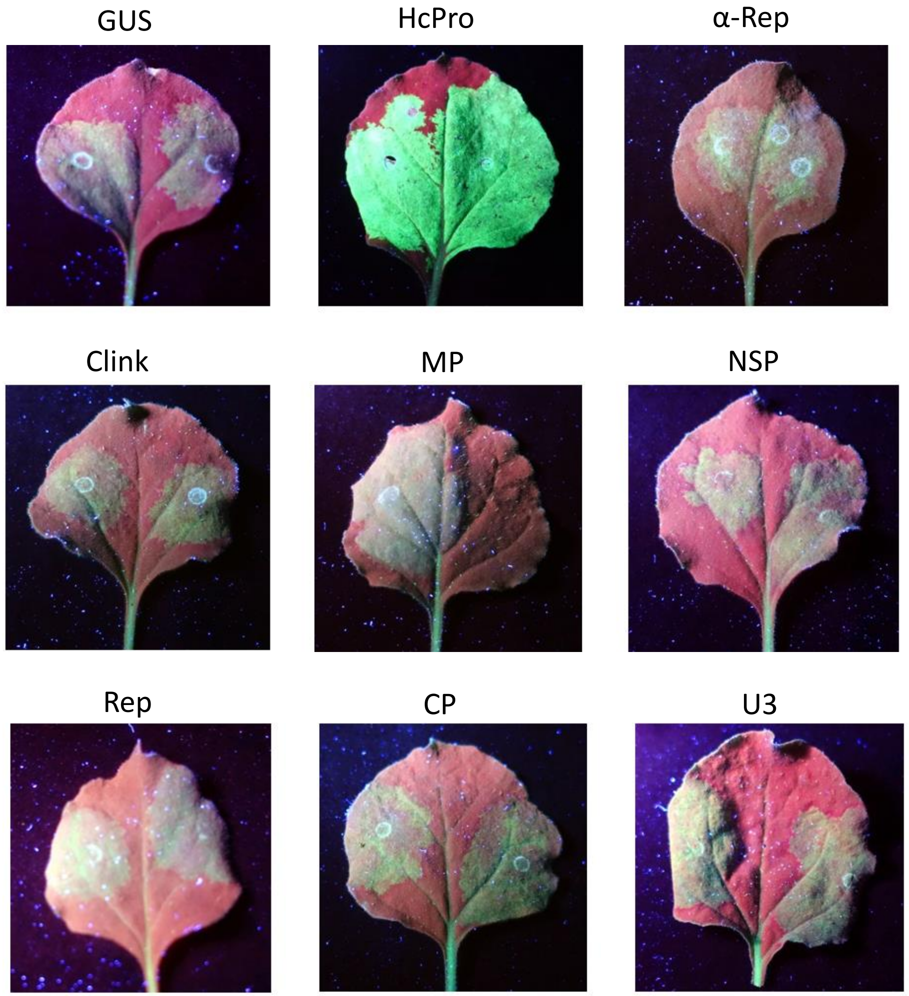

(**D**) Infiltrated leaves of *N. benthamiana* under day light

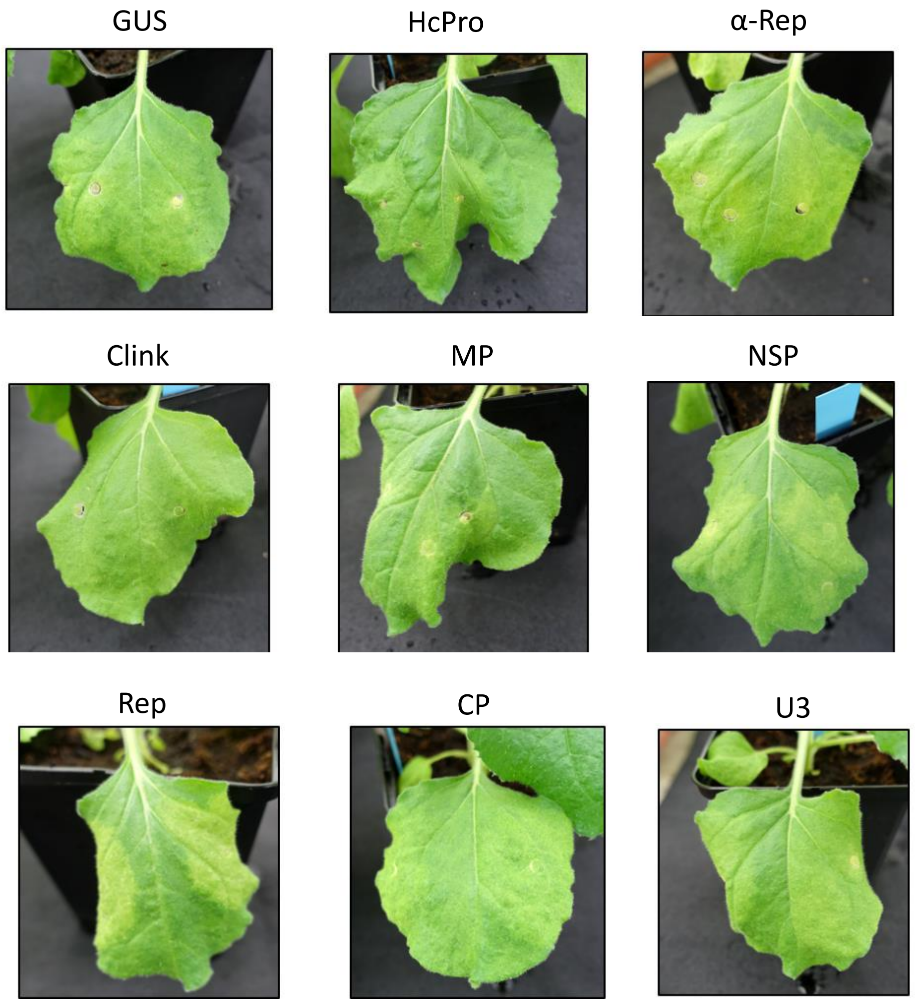

Supplement: S10 Fig — (A-B) Subcloning of BBTV and alphasatellite ORFs from partial dimer constructs (A) (see S9 Fig) into the binary vector pB7WG2 under the control of cauliflower mosaic virus 35S promoter (35S) and terminator (35S 3’) (B) by PCR amplification with viral ORF-specific primers carrying AttB1 and AttB2 recombination sites (S1 Table), followed by Gateway recombination of the PCR products into the vector. (C-D) Screening of BBTV and alphasatellite ORFs for silencing suppression and chlorosis induction following infiltration of the binary constructs in leaves of N. benthamiana GFP-transgenic (16c line) plants at 8 days post infiltration (dpi) under ultraviolet (UV) light (C) and day light (D). Pictures of representative leaves (from three infiltrated plants per construct, three leaves per plant) are shown. The experiment was repeated 3 times with similar results. Note that in addition to the BBTV Rep construct inducing strong chlorosis, the BBTV NSP construct induced weaker chlorosis, albeit it was barely visible in some of the infiltrated leaves. (PDF) [file ppat.1010448.s011.pdf]
