# Supplementary material for: A newly emerging alphasatellite affects banana bunchy top virus replication, transcription, siRNA production and transmission by aphids
Source: PLoS Pathog. 2022 Apr 12;18(4):e1010448. doi: 10.1371/journal.ppat.1010448 (PMC9049520; doi:10.1371/journal.ppat.1010448)
Supplement: S11 Fig — Viral DNA concentrations for each BBTV component (C, M, N, R, S, U3) and alphasatellite (a) and for total helper virus without (BBTV) or with (BBTVa) alphasatellite were measured by quantitative PCR using equal amounts of total DNA in (A) plants without (n = 77) and with (n = 47) alphasatellite (all experiments) and (E) aphids without (n = 58) and with (n = 56) alphasatellite (all experiments). Concentrations of total host DNA in the same samples were measured by qPCR with primers specific for the banana and aphid housekeeping genes (RPS2 and eEF1a, respectively). * Kruskal-Wallis P < 0.05. *** Kruskal-Wallis P < 0.005. (PDF) [file ppat.1010448.s012.pdf]

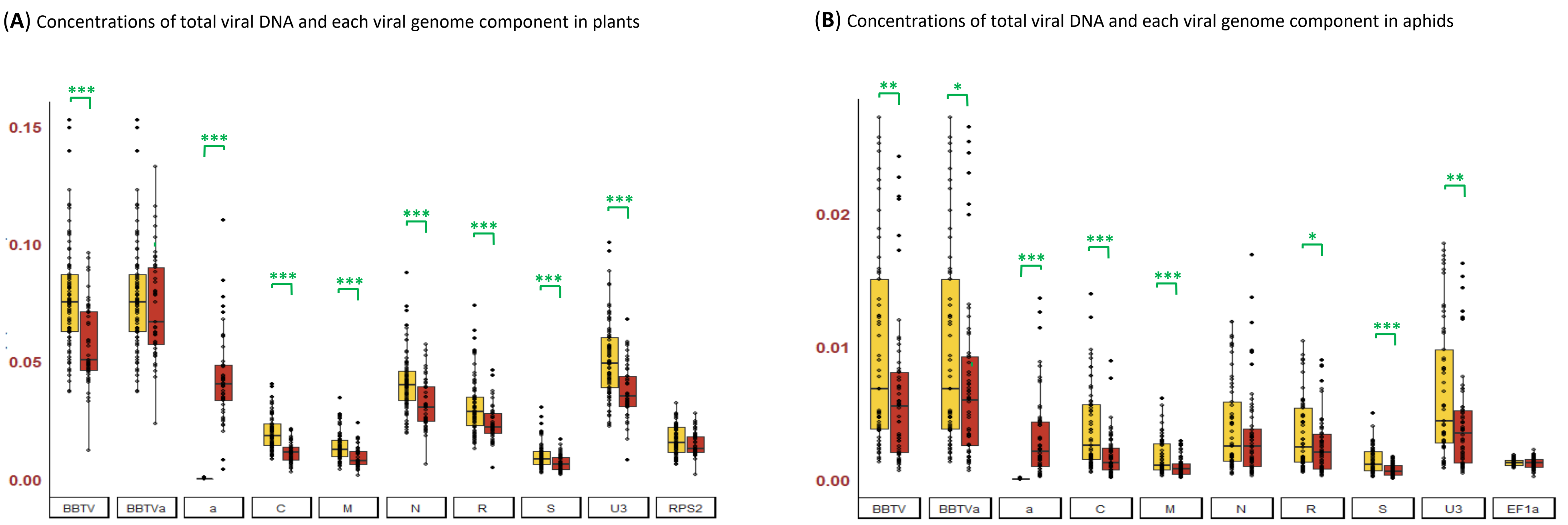

S11 Fig. Impact of DRC alphasatellite on viral DNA concentrations in plants and aphids. Viral DNA concentrations for each BBTV component (C, M, N, R, S, U3) and alphasatellite (a) and for total helper virus without (BBTV) or with (BBTVa) alphasatellite were measured by quantitative PCR using equal amounts of total DNA in (**A**) plants without (n = 77) and with (n = 47) alphasatellite (all experiments) and (**E**) aphids without (n = 58) and with (n = 56) alphasatellite (all experiments). Concentrations of total host DNA in the same samples were measured by qPCR with primers specific for the banana and aphid housekeeping genes (RPS2 and eEF1a, respectively). * Kruskal-Wallis $P < 0.05$. *** Kruskal-Wallis $P < 0.005$.
