# Supplementary material for: A newly emerging alphasatellite affects banana bunchy top virus replication, transcription, siRNA production and transmission by aphids
Source: PLoS Pathog. 2022 Apr 12;18(4):e1010448. doi: 10.1371/journal.ppat.1010448 (PMC9049520; doi:10.1371/journal.ppat.1010448)
Supplement: S12 Fig — Viral DNA loads for each BBTV component (C, M, N, R, S, U3) and alphasatellite (alpha) as well as for total helper virus (BBTV) were measured by quantitative PCR (using the banana RPS2 gene as internal control for normalization) in 3 plants infected with BBTV alone (ADVT-2, ADVT-5, ADVT-6) and 3 plants co-infected with BBTV and alphasatellite (ADVT-8, ADVT-9, ADVT-10). (A) Loads of helper virus (BBTV, yellow) and alphasatellite (alpha, red) DNA in plants without and with alphasatellite. (B) Mean loads of helper virus (BBTV, yellow) and alphasatellite (alpha, red) DNA in plants without (BBTV) and with (BBTVa) alphasatellite. (C) Mean loads of each BBTV component and alphasatellite (alpha) DNA in plants with (red) and without (yellow) alphasatellite. (D) Loads of each BBTV component and alphasatellite (alpha) in plants without (yellow) and with (red) alphasatellite. (E) Helper virus genome formula in plants without (yellow, BBTV) and with (red, BBTVa) alphasatellite. (F) Virome genome formula in plants without (yellow, BBTV) and with (red, BBTVa) alphasatellite. Error bars represent standard deviations. (PDF) [file ppat.1010448.s013.pdf]

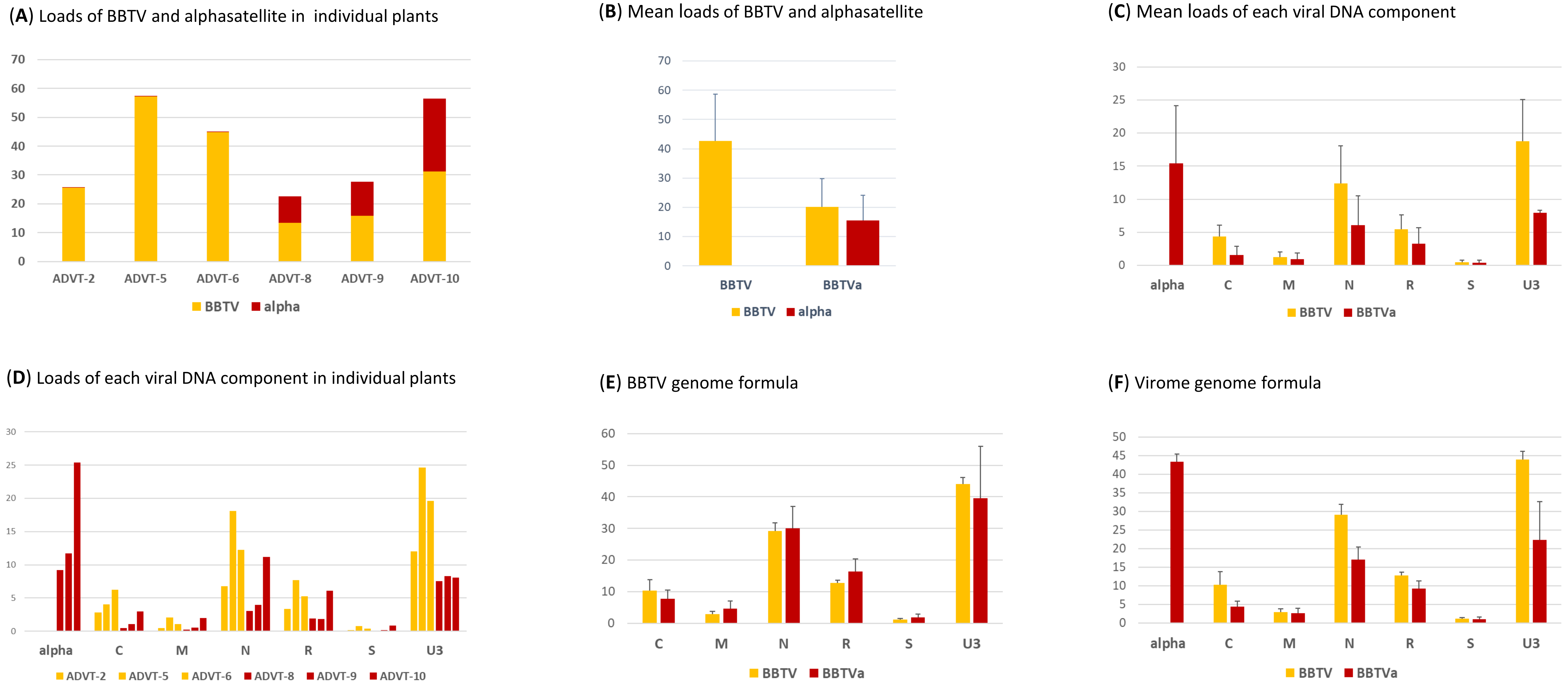

S12 Fig. Viral DNA loads and formulas in BBTV-infected Cavendish banana plants with or without DRC alphasatellite selected for viral transcriptome and small RNA-ome profiling. Viral DNA loads for each BBTV component (C, M, N, R, S, U3) and alphasatellite (alpha) as well as for total helper virus (BBTV) were measured by quantitative PCR (using the banana RPS2 gene as internal control for normalization) in 3 plants infected with BBTV alone (ADVT-2, ADVT-5, ADVT-6) and 3 plants co-infected with BBTV and alphasatellite (ADVT-8, ADVT-9, ADVT-10). (**A**) Loads of helper virus (BBTV, yellow) and alphasatellite (alpha, red) DNA in plants without and with alphasatellite. (**B**) Mean loads of helper virus (BBTV, yellow) and alphasatellite (alpha, red) DNA in plants without (BBTV) and with (BBTVa) alphasatellite. (**C**) Mean loads of each BBTV component and alphasatellite (alpha) DNA in plants with (red) and without (yellow) alphasatellite. (**D**) Loads of each BBTV component and alphasatellite (alpha) in plants without (yellow) and with (red) alphasatellite. (**E**) Helper virus genome formula in plants without (yellow, BBTV) and with (red, BBTVa) alphasatellite. (**F**) Virome genome formula in plants without (yellow, BBTV) and with (red, BBTVa) alphasatellite. Error bars represent standard deviations.
