# Supplementary material for: A newly emerging alphasatellite affects banana bunchy top virus replication, transcription, siRNA production and transmission by aphids
Source: PLoS Pathog. 2022 Apr 12;18(4):e1010448. doi: 10.1371/journal.ppat.1010448 (PMC9049520; doi:10.1371/journal.ppat.1010448)

S13 Fig. Single nucleotide resolution maps of Illumina mRNA-seq reads representing viral mRNAs and other viral transcripts from BBTV-infected Cavendish banana plants with or without DRC alphasatellite and identification of poly(A) sites by analysis of the mapped reads. For each of the two conditions, i.e. without (BBTV-alpha) and with (BBTV+alpha) alphasatellite, Illumina 75 nt reverse reads of the three biological replicates (leaf tissues of three plants) were combined and mapped simultaneously onto the reference sequences of six BBTV components (-/+ alphasatellite). Histograms plot the numbers of viral 75 nt sense and antisense reads at each nucleotide position of the 1018-to-1111 nt BBTV genome components (DNAs C, M, N, R, S, U3 - subpanels **A1-F1**) and 1105 nt alphasatellite (Alpha – subpanel **G1**): blue bars above the axis represent sense reads starting at each respective position, while red bars below the axis represent antisense reads ending at each respective position. The genome organizations of BBTV components and alphasatellite are shown schematically above the respective histograms, with the Pol II promoter (TATA-box and transcription start site, TSS) and terminator (polyA signal, PAS) elements indicated in pink, capped and polyadenylated mRNA shown as solid blue lines, viral protein-coding ORFs boxed and their nucleotide positions given. In each panel (**A-G**), subpanels 2 and 3 show the Pol II terminator region with the mapped poly(A) sites and the respective poly(A) signals at the upstream positions visualized using MISIS-2 (Seguin et al. 2016). Note that the mapped mRNA reads ending with oligo(A) tails generate A-SNPs in the sequence logo just downstream of the poly (A) site (i.e., pre-mRNA cleavage and polyadenylation site).

Figure S13 **A1**

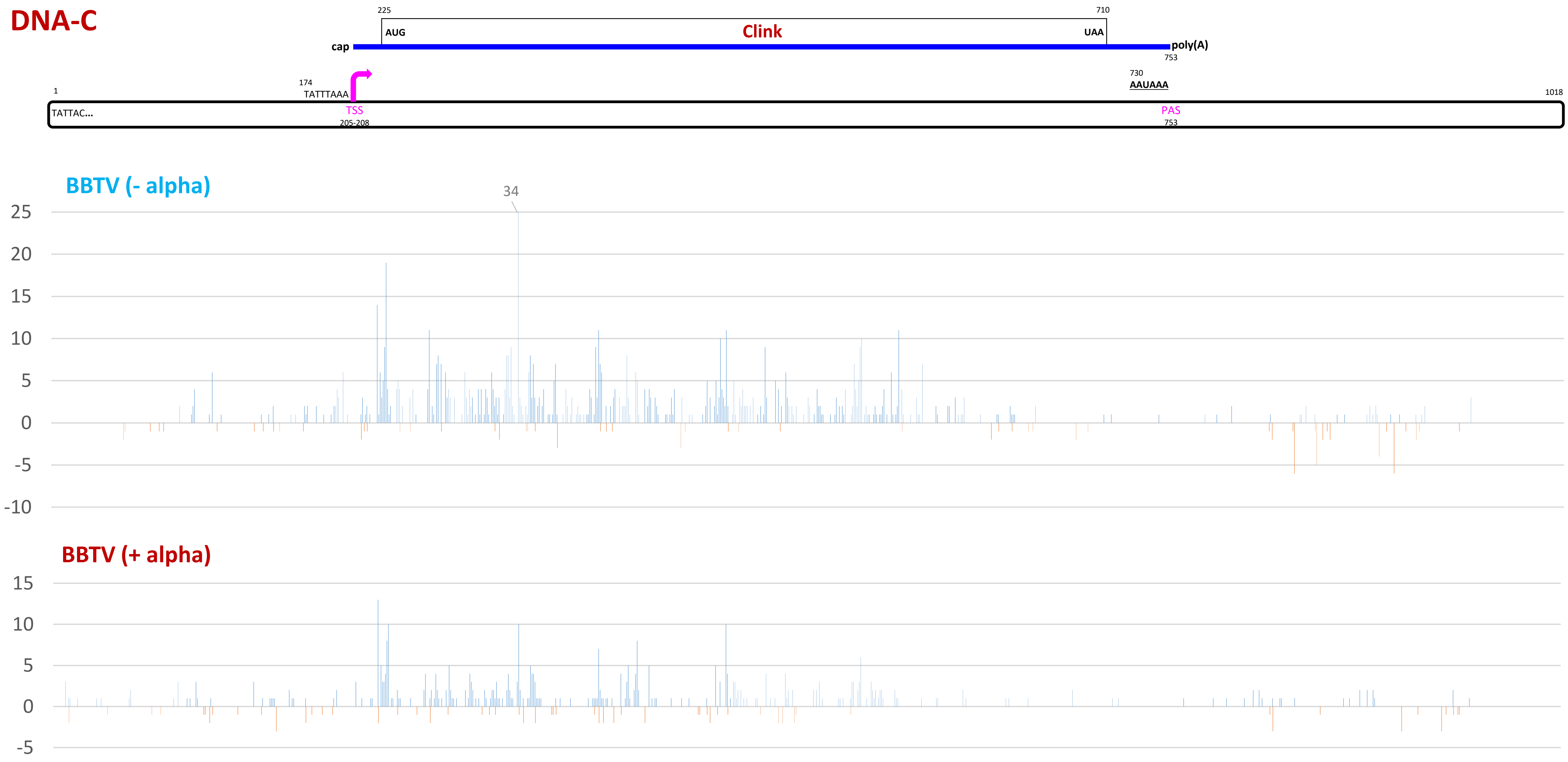

Figure S13 **A2**

DNA-C

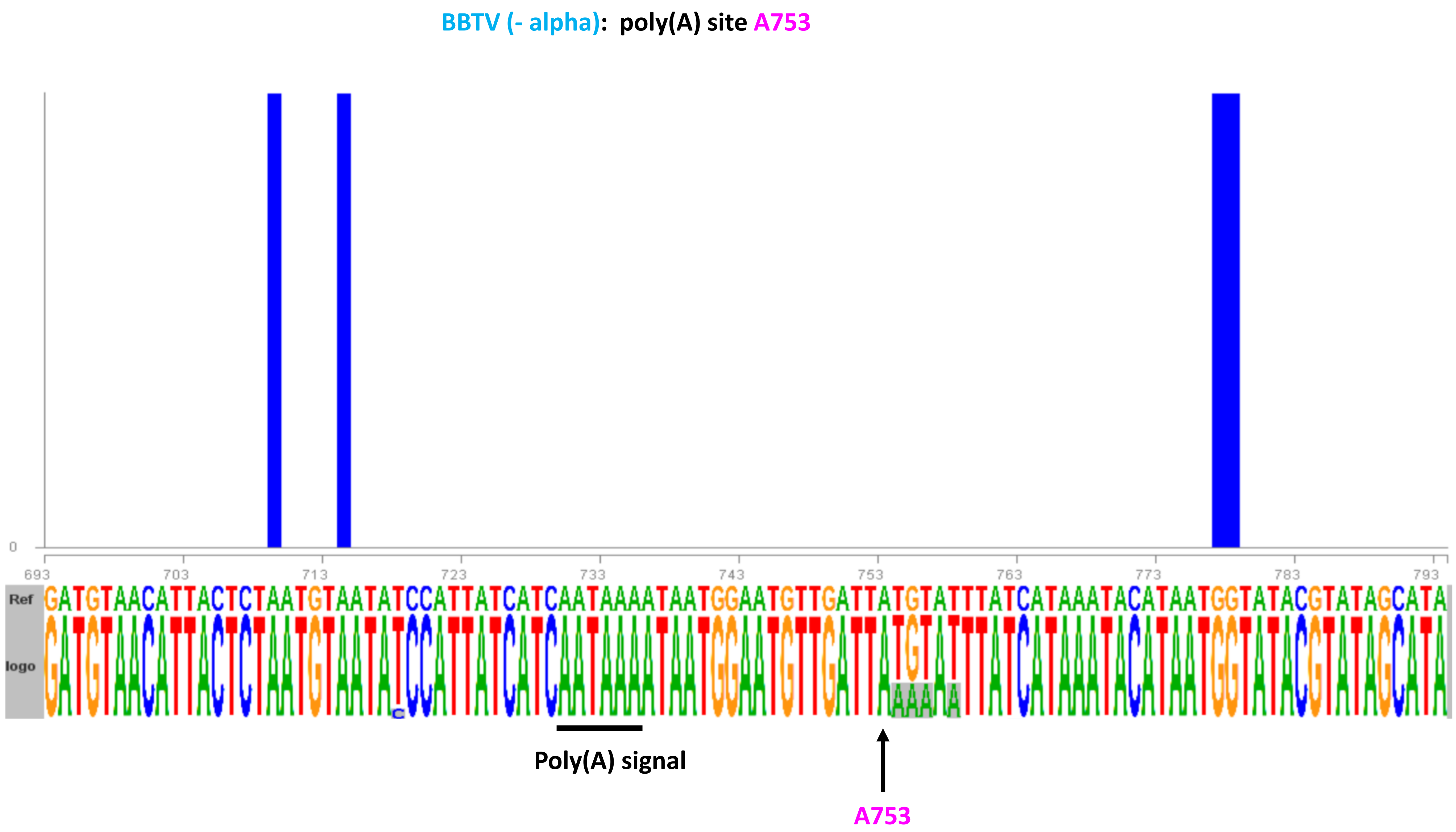

Figure S13 **B1**

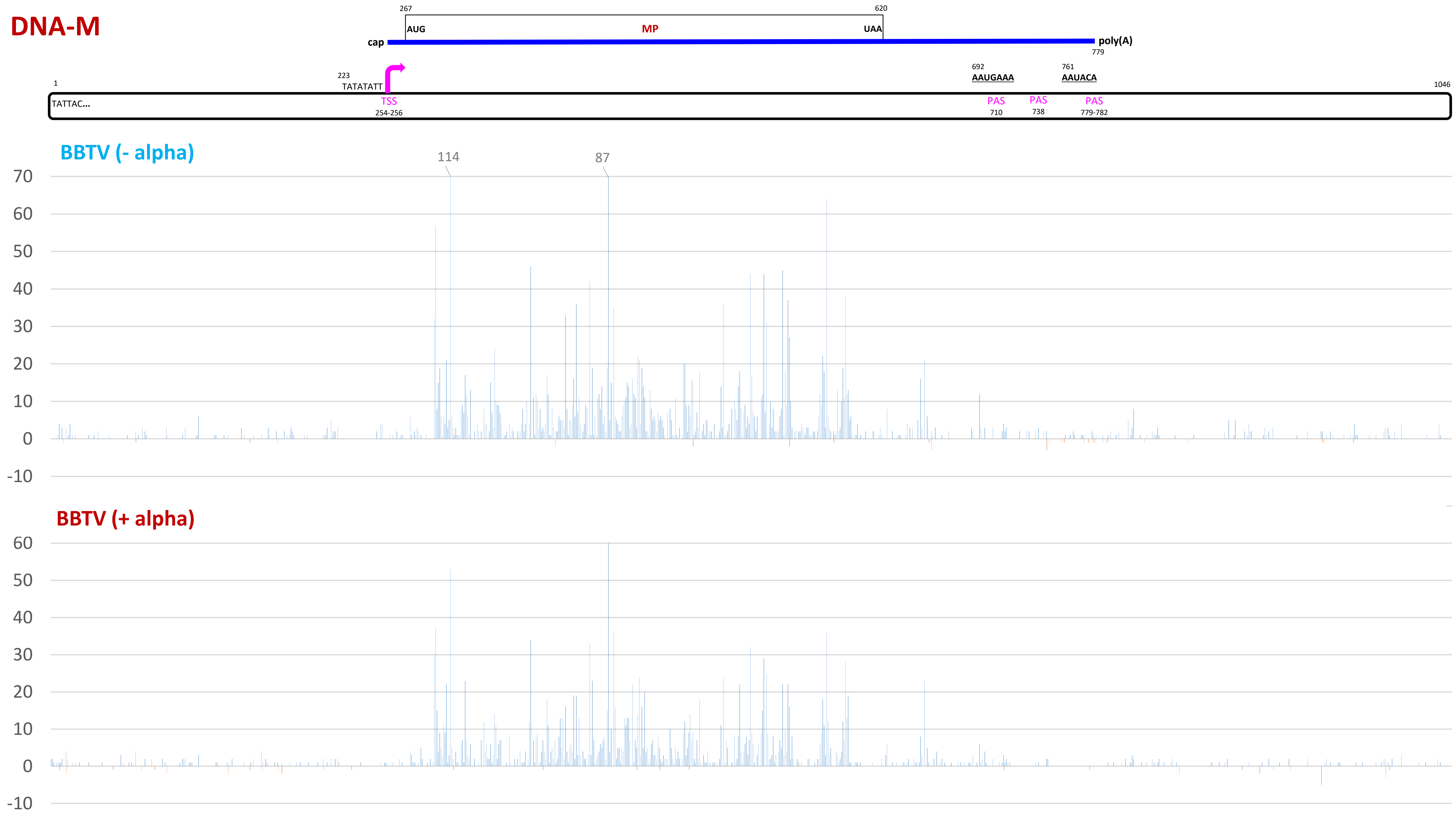

Figure S13 **B2**

DNA-M

BBTV (- alpha): poly(A) sites A710 and A779

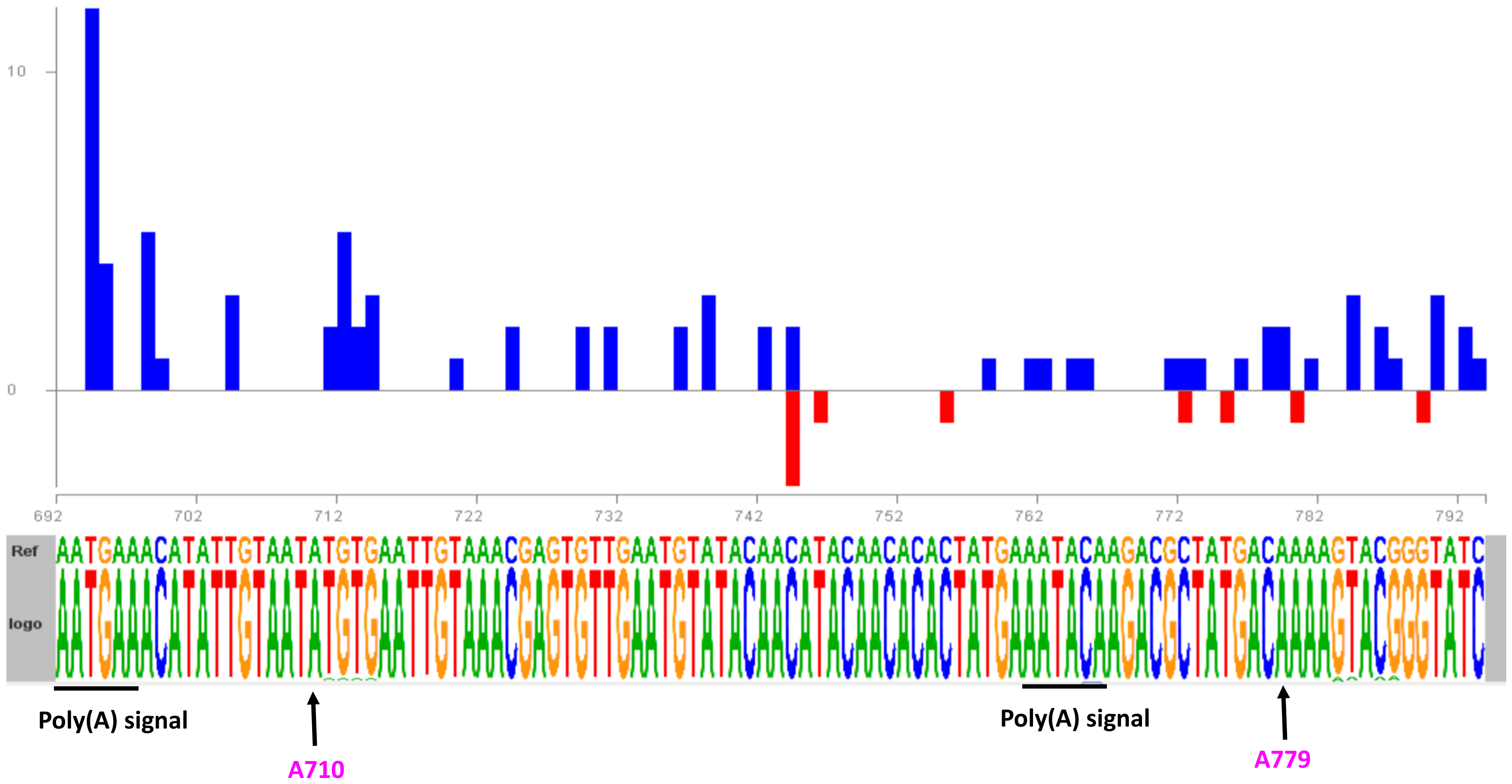

Figure S13 **B3**

DNA-M

BBTV all (with alpha): poly(A) sites A738 and A779

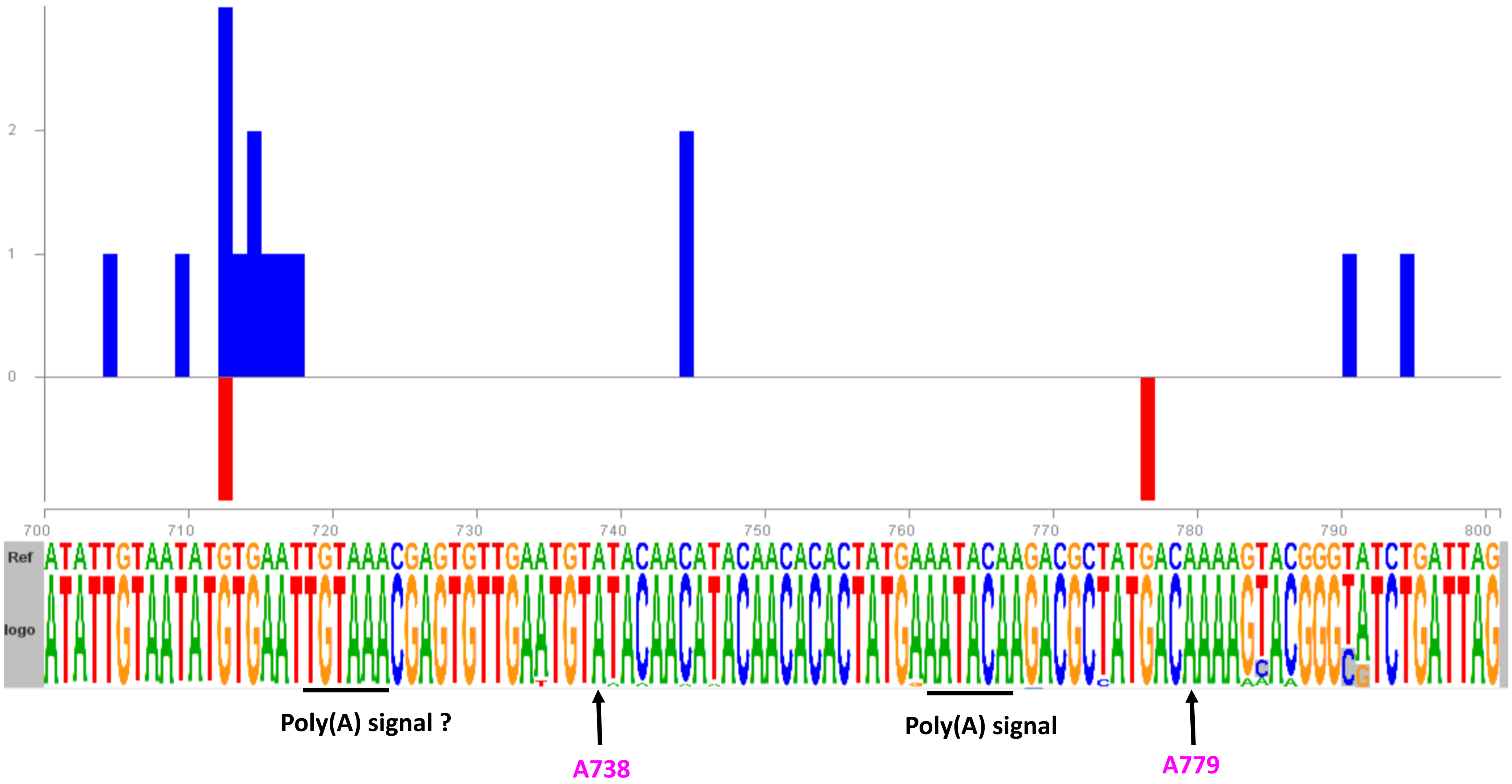

Figure S13 **C1**

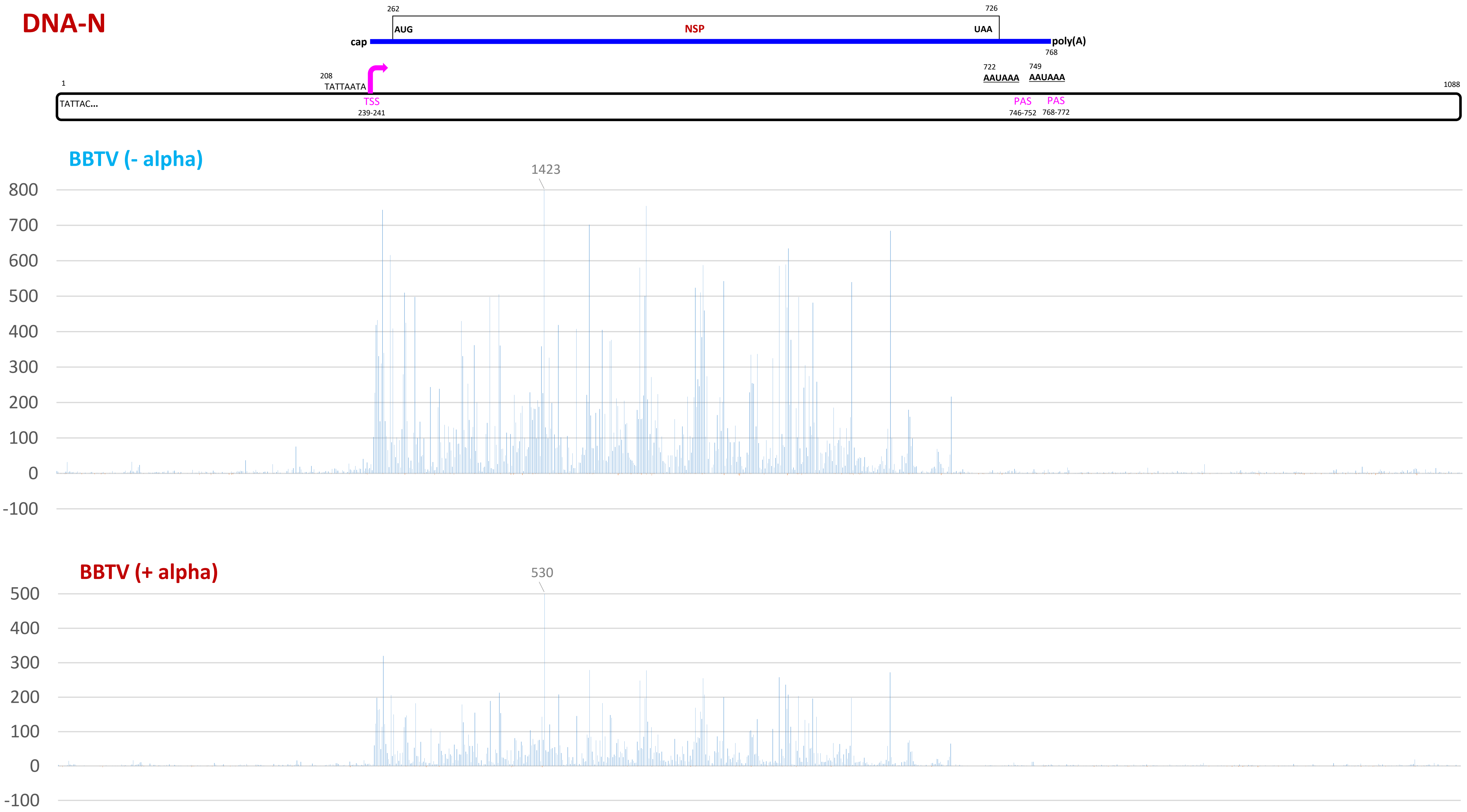

Figure S13 **B2**

DNA-M

BBTV (- alpha): poly(A) sites A749, A752 and A768

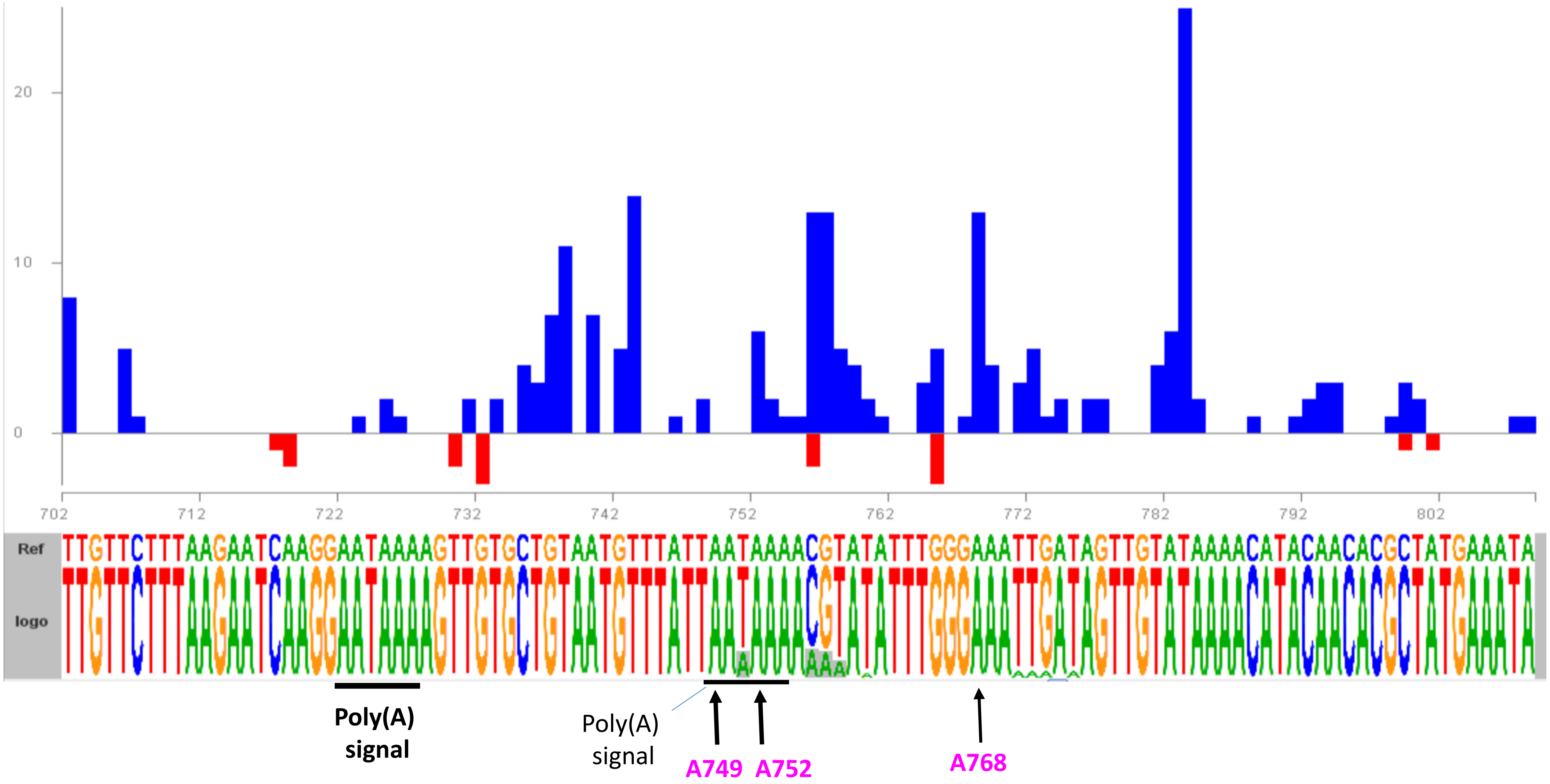

Figure S13 **B2**

DNA-M

BBTV (+ alpha): poly(A) sites A746, A749, A752 and T772

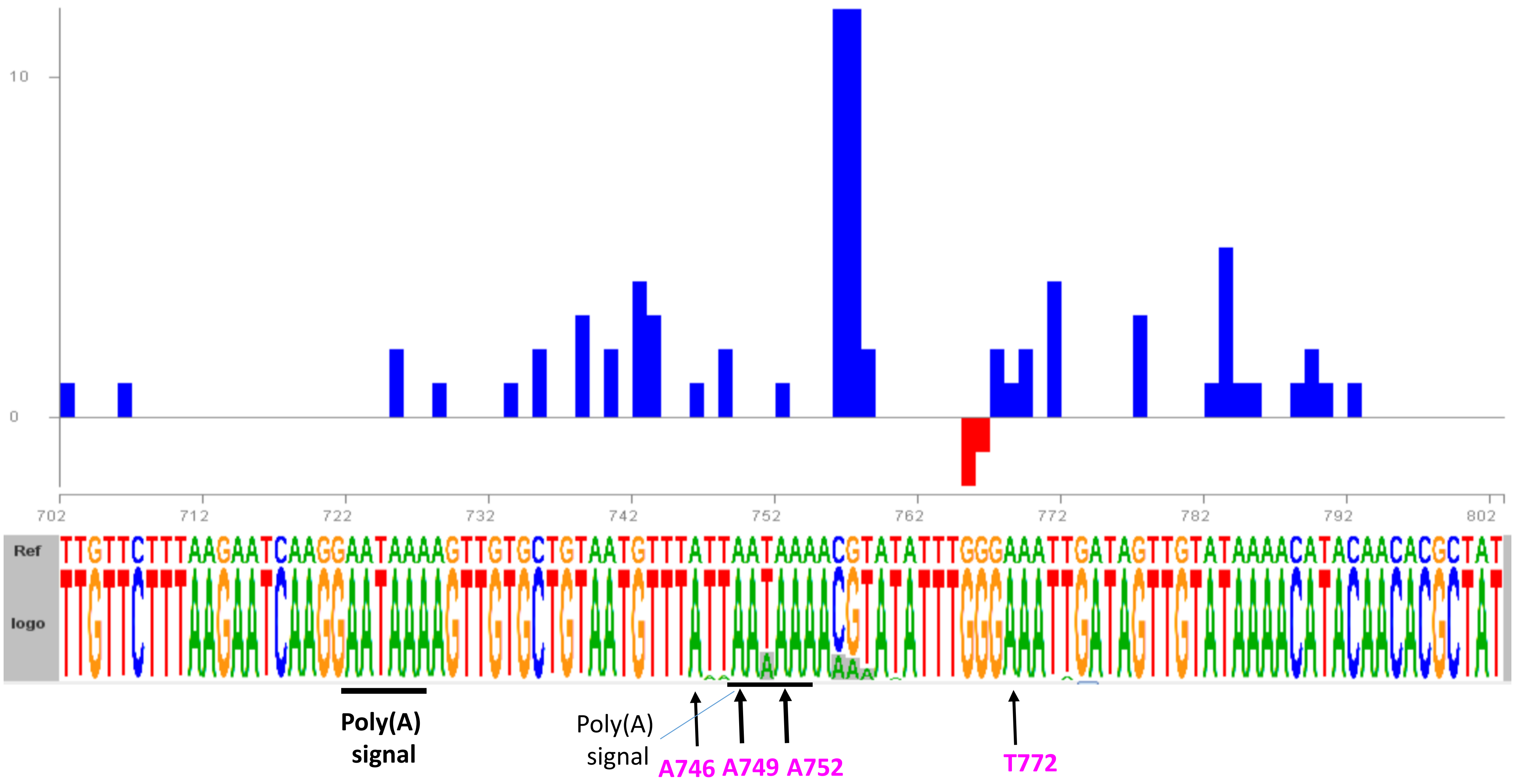

Figure S13 **D1**

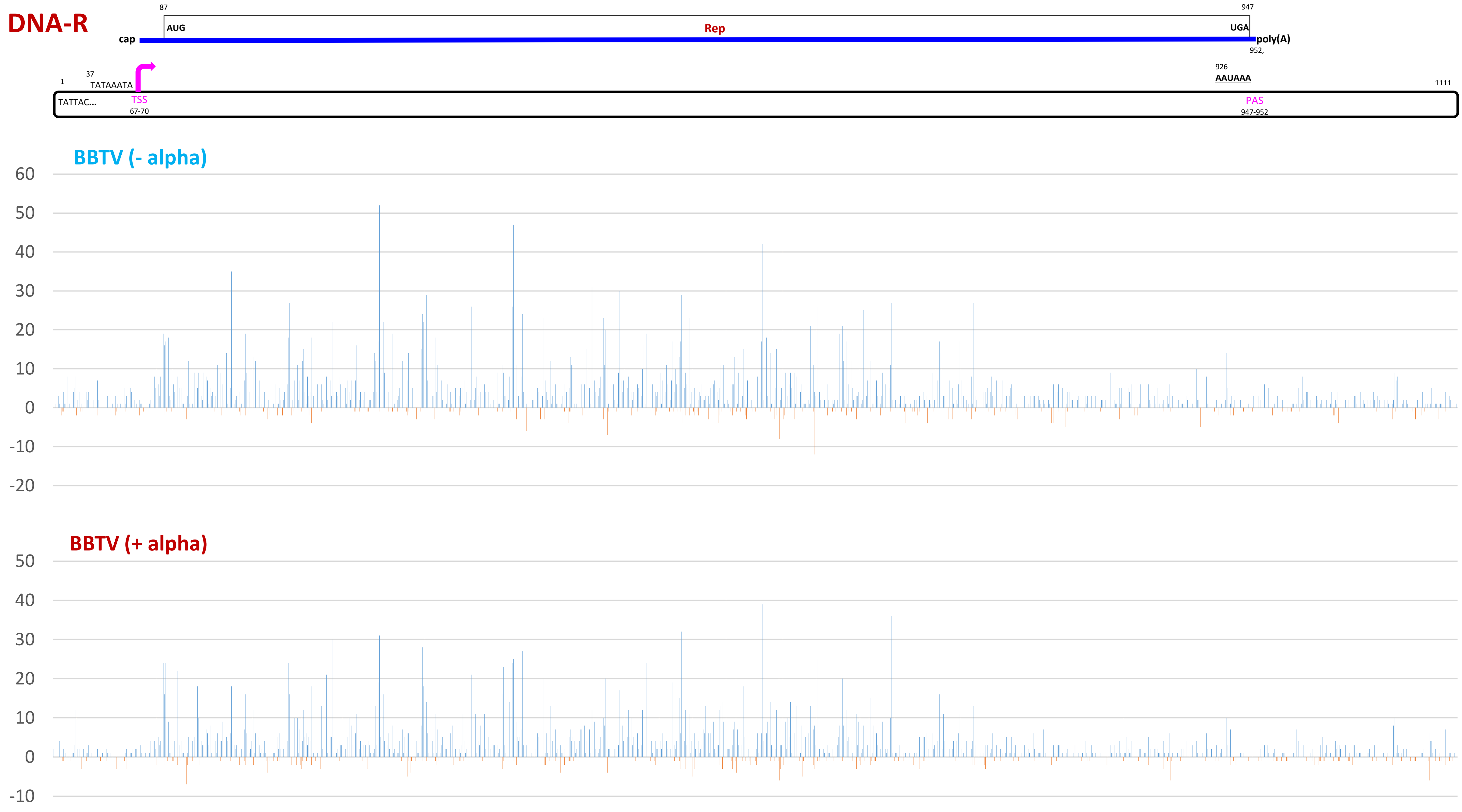

Figure S13 **D2**

DNA-R

BBTV (- alpha): poly(A) site A947

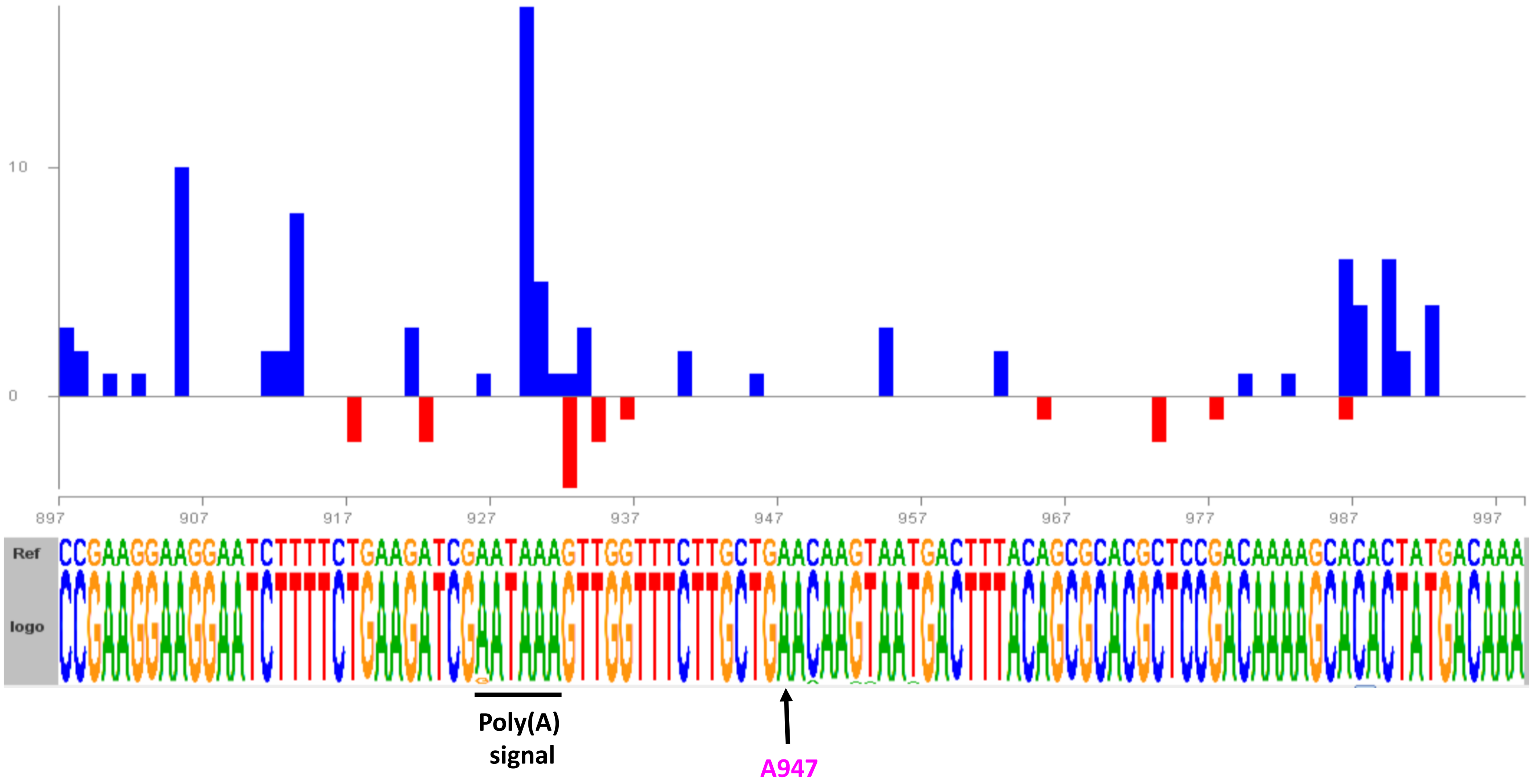

Figure S13 **D3**

DNA-R

BBTV (+ alpha): poly(A) site A950

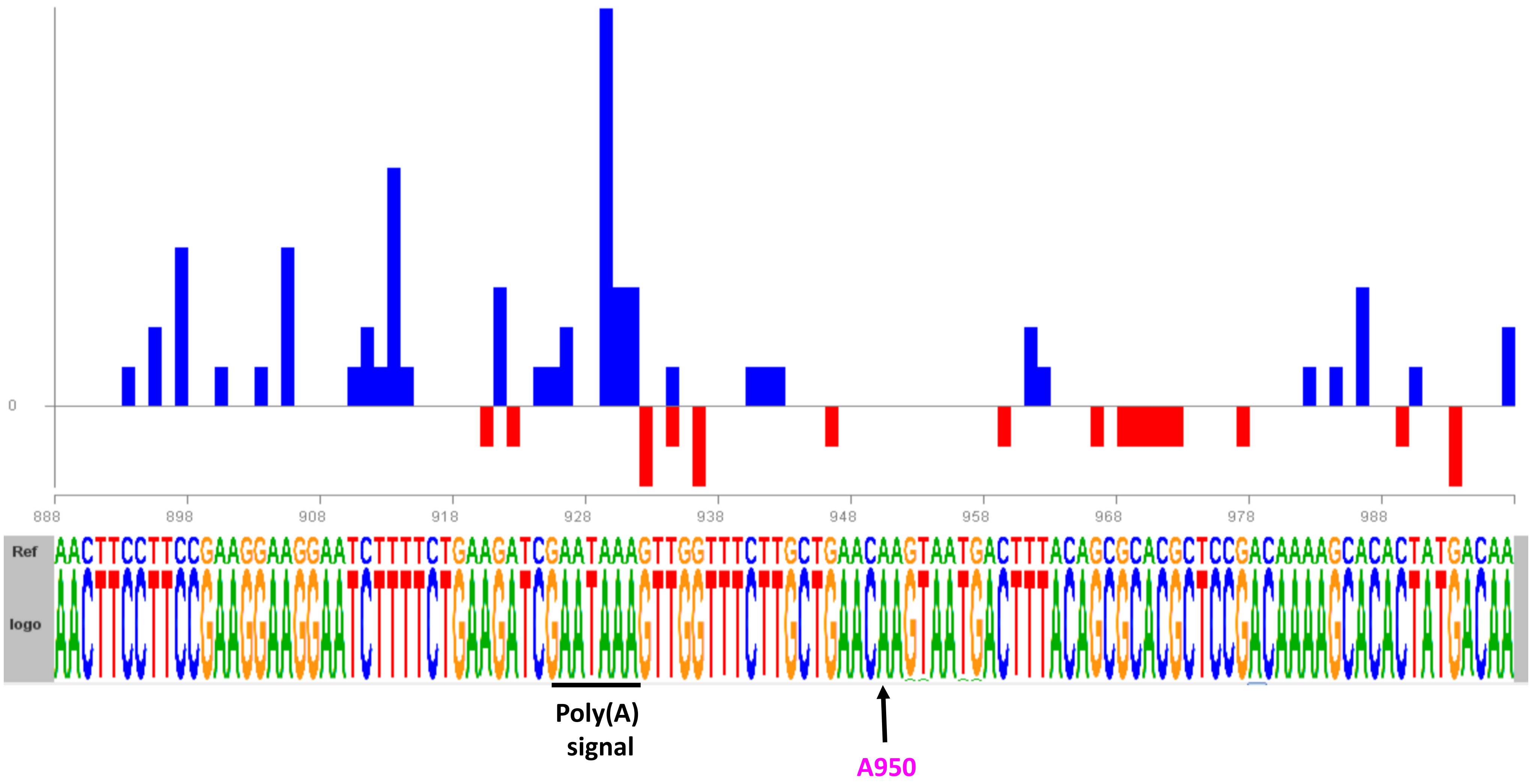

Figure S13 **E1**

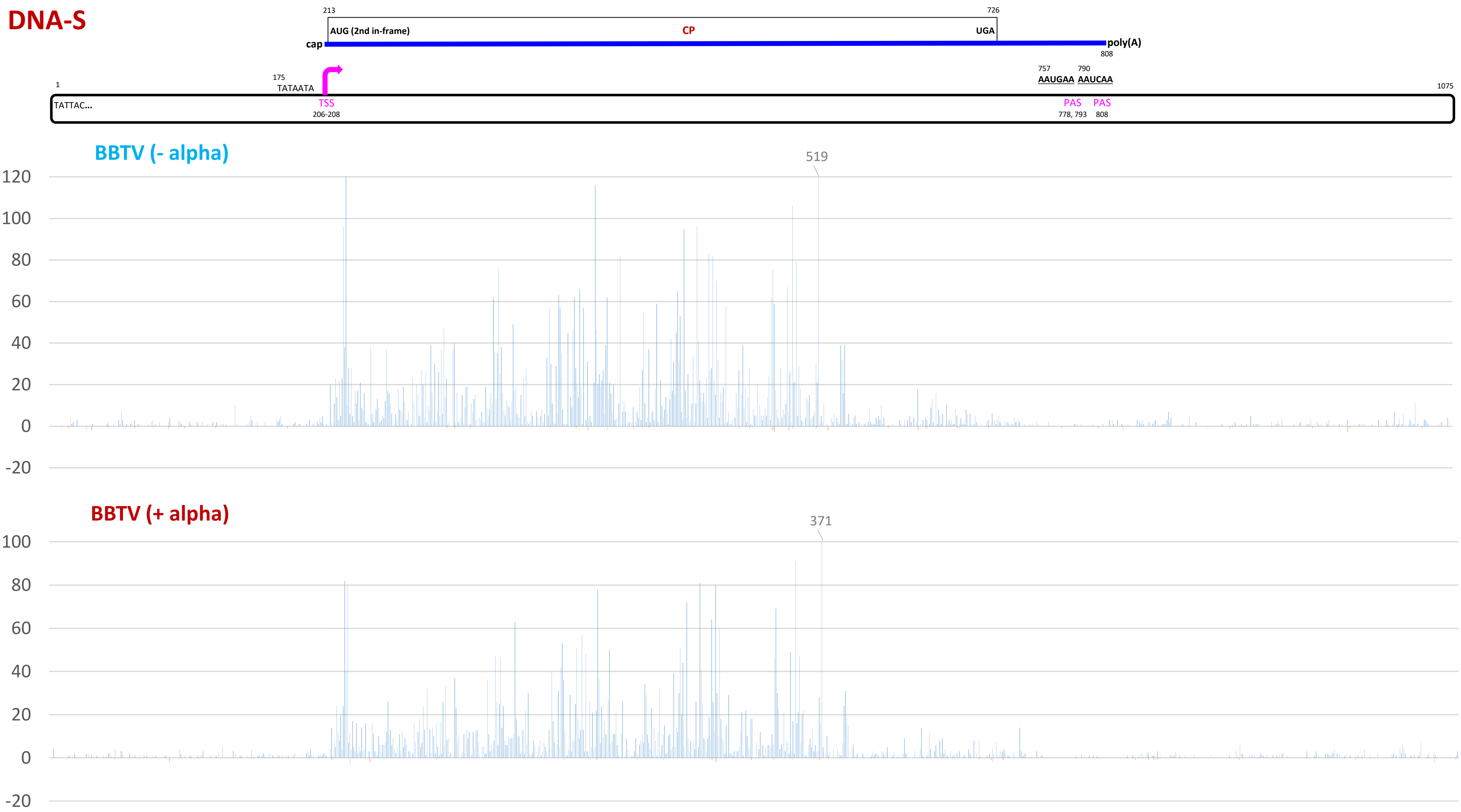

Figure S13 **E2**

DNA-S

BBTV (- alpha): Poly(A) sites A778, G808

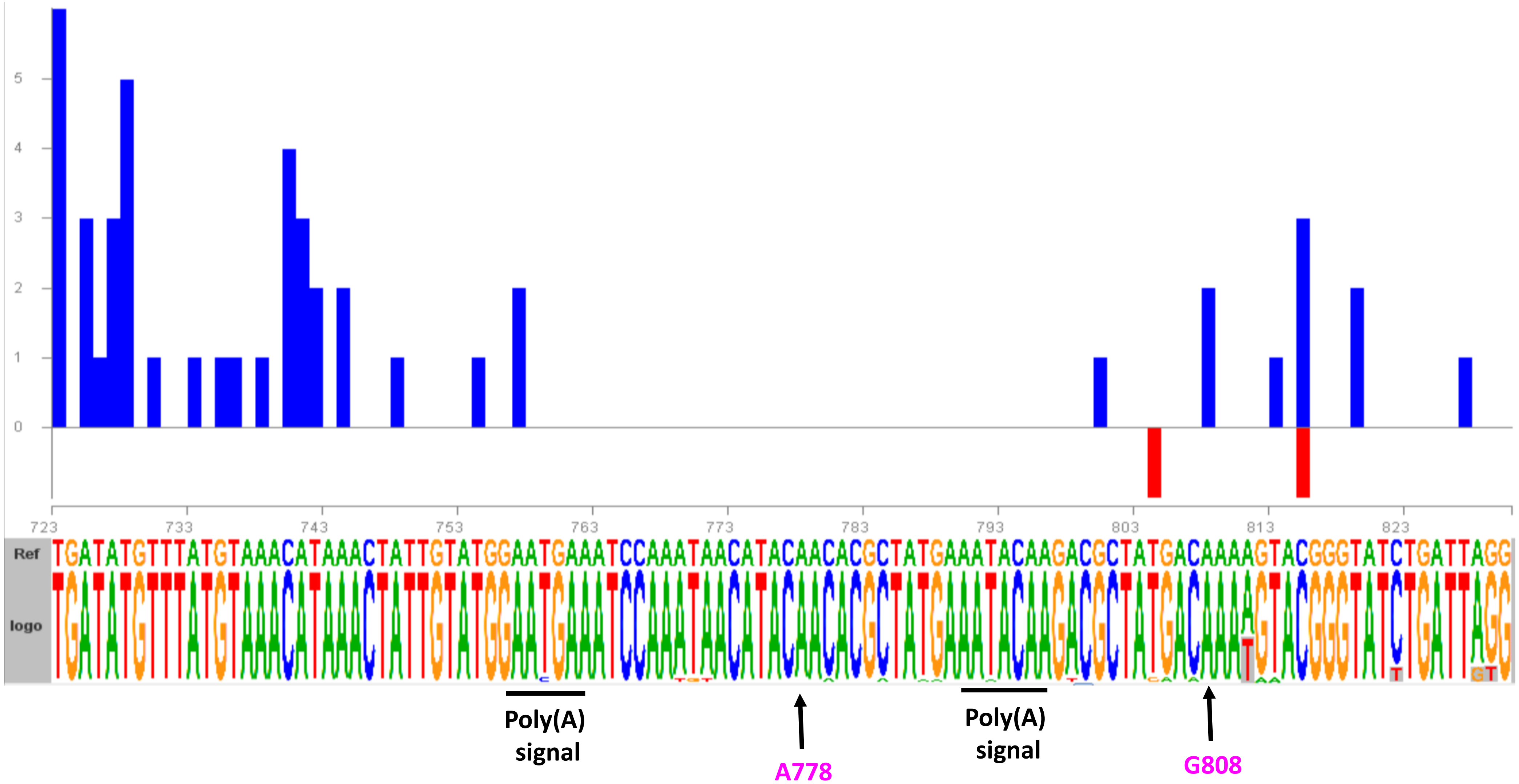

Figure S13 **E3**

DNA-S

BBTV (+ alpha): Poly(A) sites A793, A808

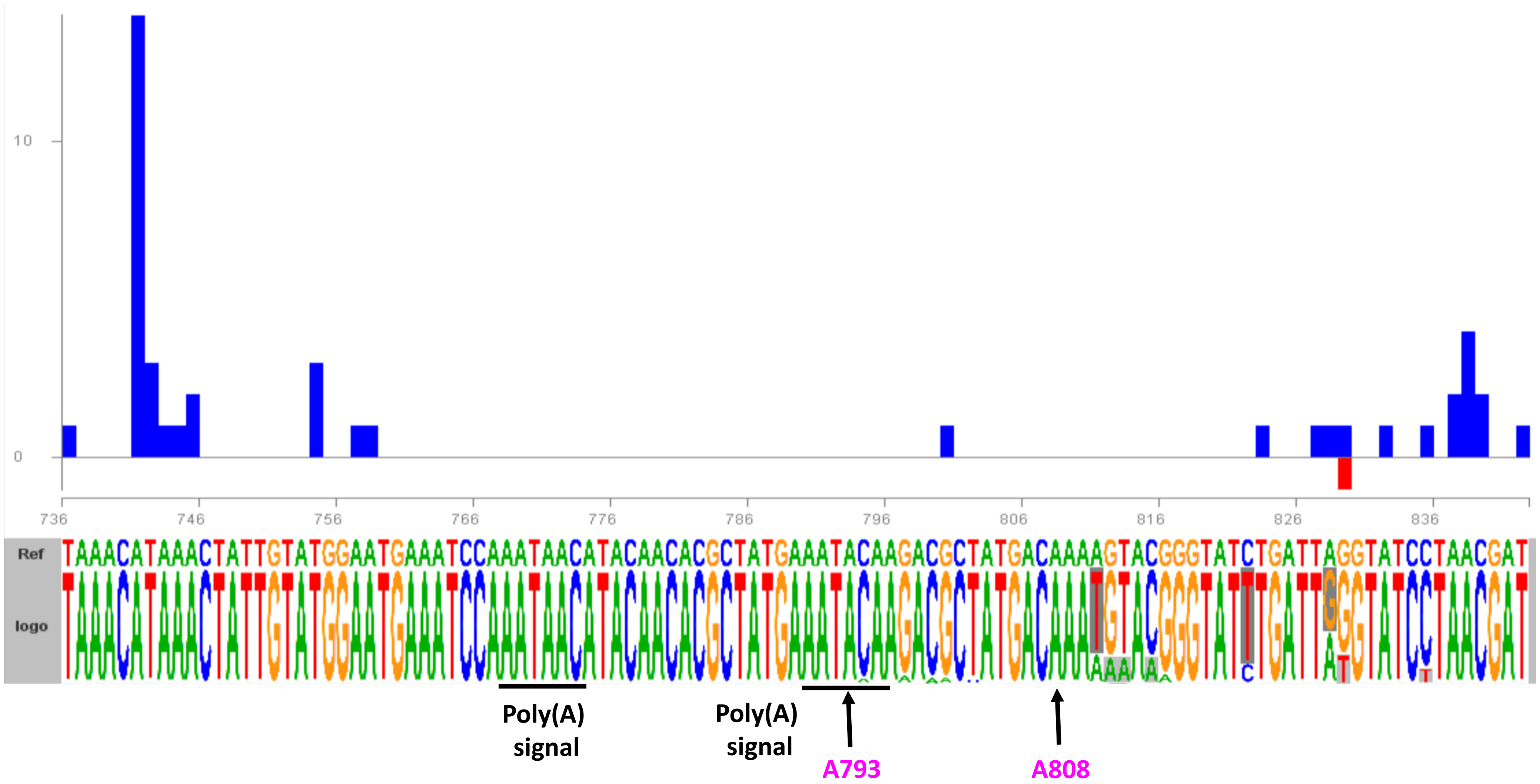

Figure S13 **F1**

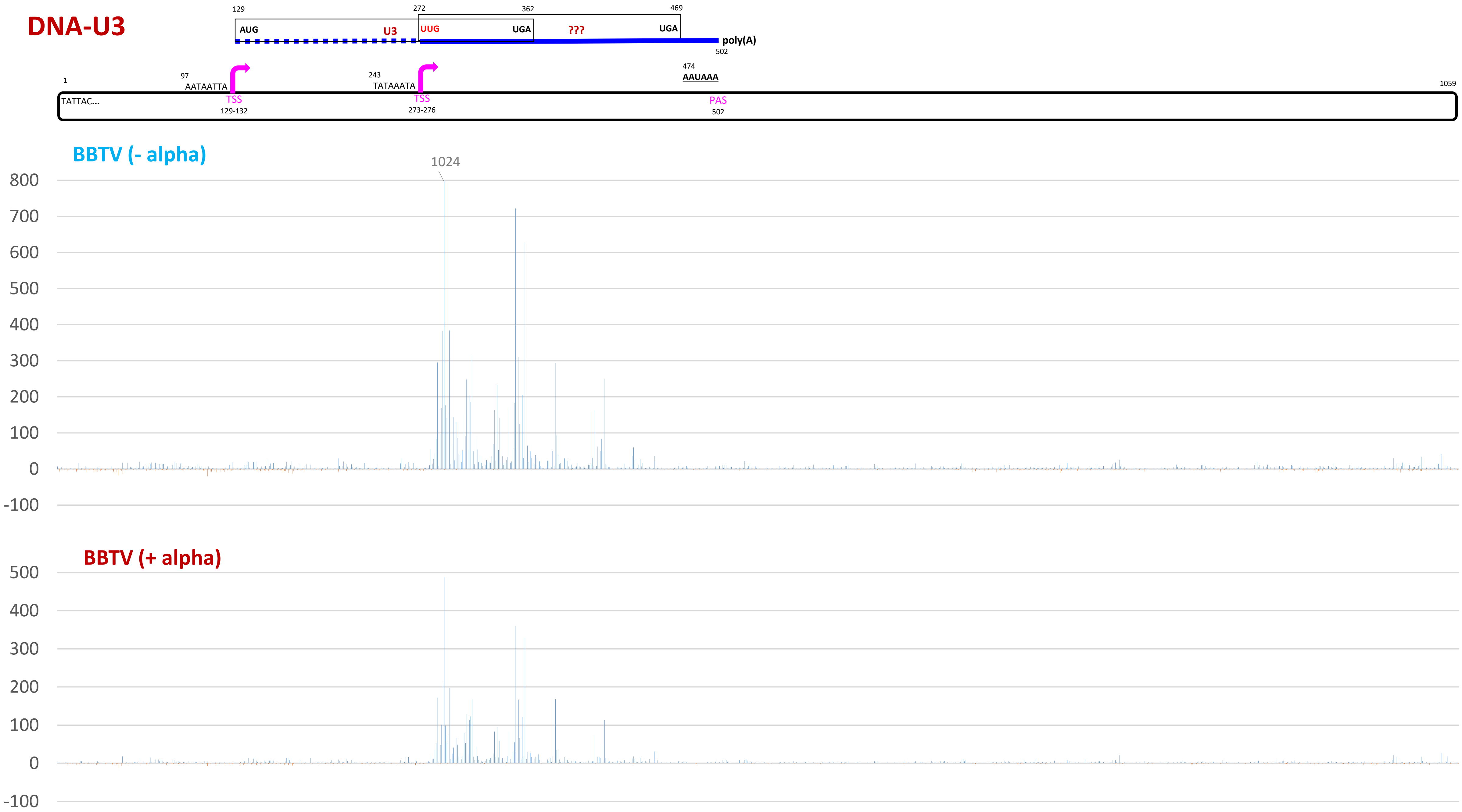

Figure S13 **F2**

DNA-U3

BBTV (- alpha): Poly(A) site A502

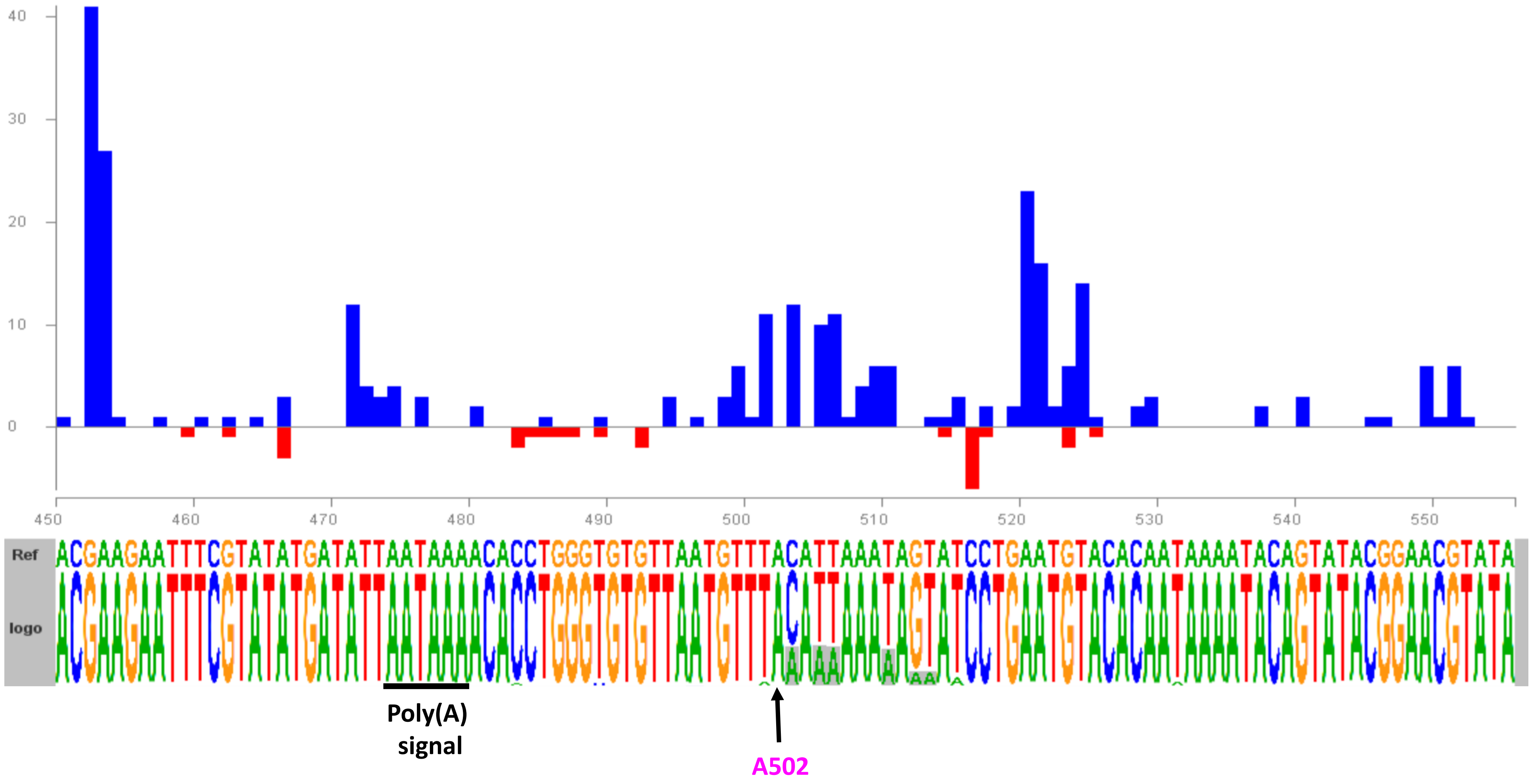

Figure S13 **F3**

DNA-U3

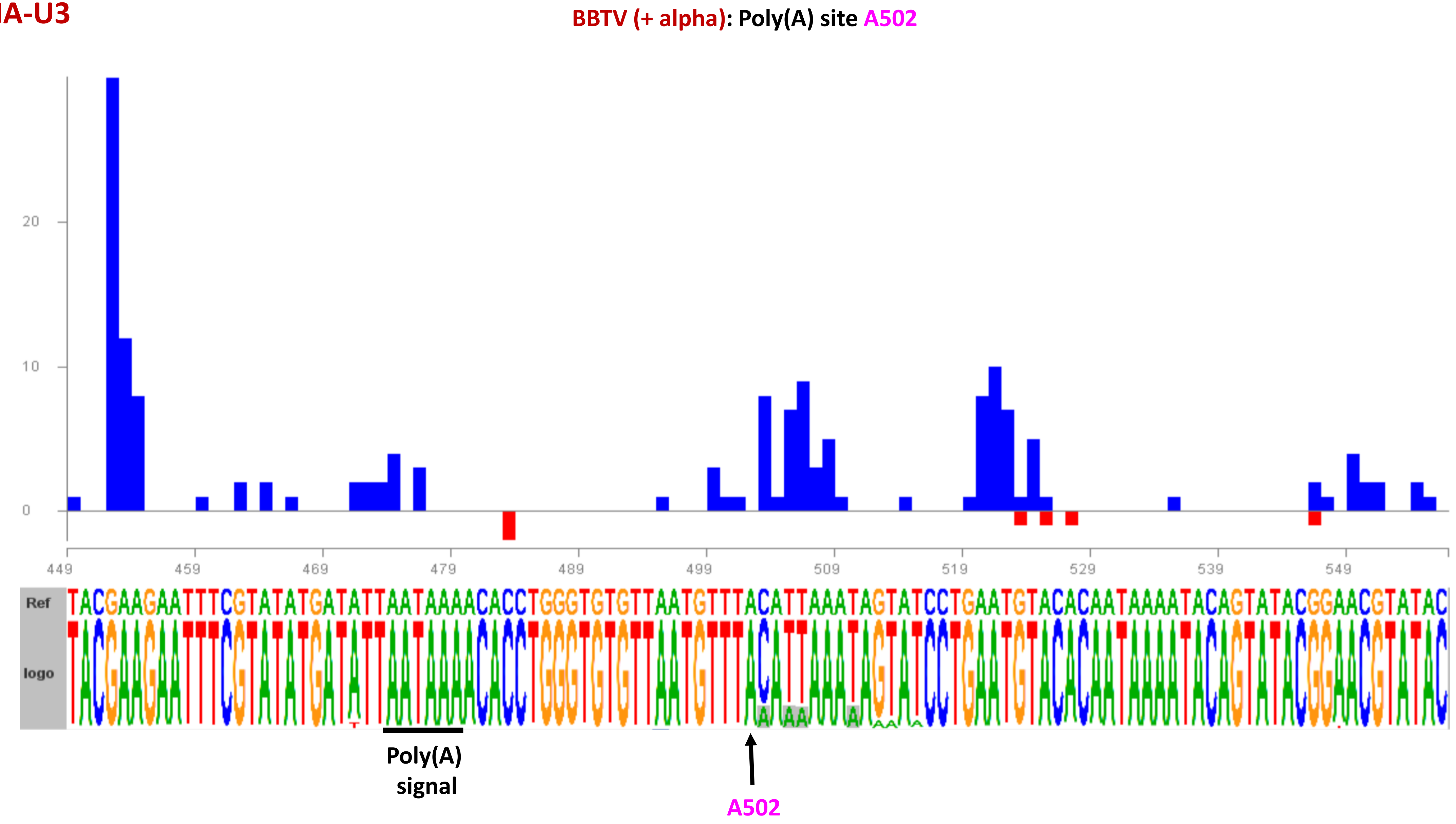

Figure S13 **G1**

Alpha

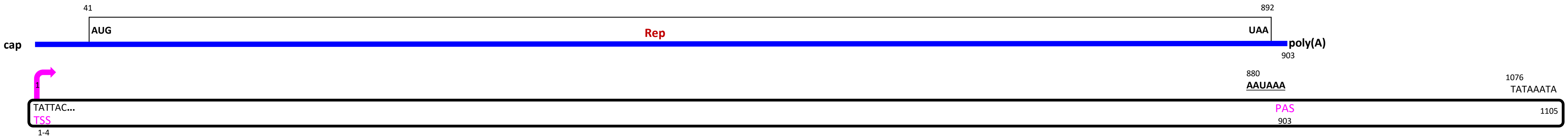

BBTV + alpha

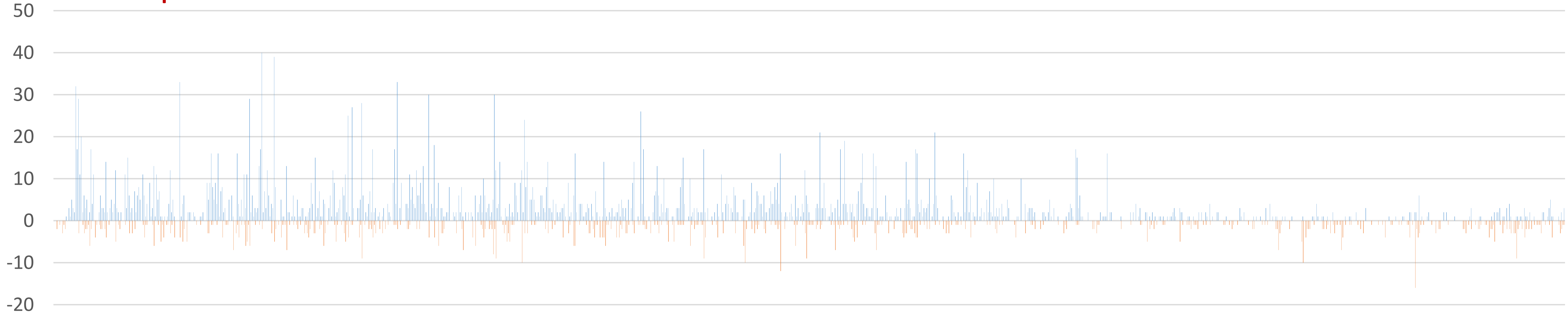

Figure S13 **G2**

Alpha

Poly(A) site A903

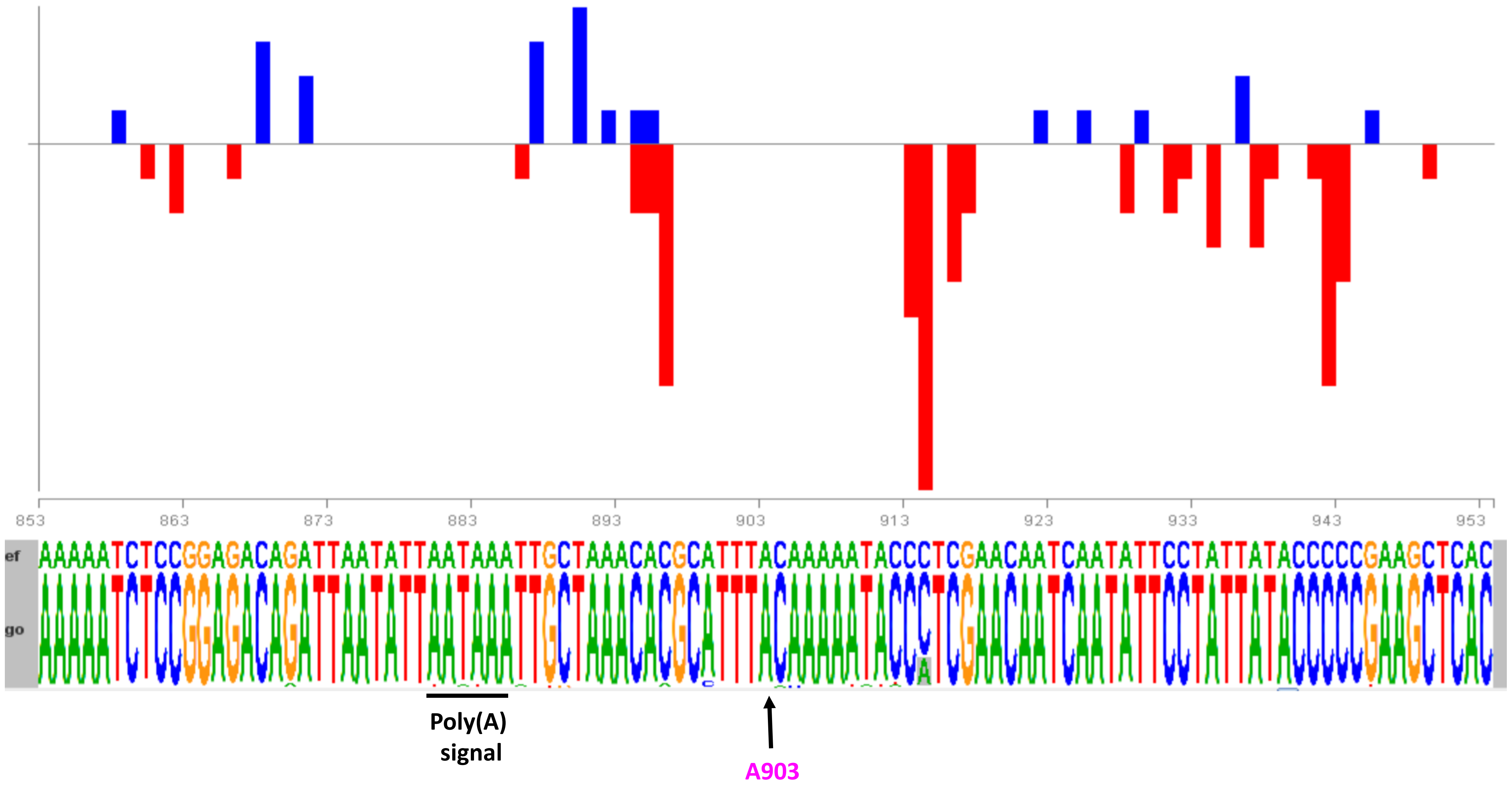

Supplement: S13 Fig — For each of the two conditions, i.e. without (BBTV-alpha) and with (BBTV+alpha) alphasatellite, Illumina 75 nt reads of the three biological replicates (leaf tissues of three plants) were combined and mapped simultaneously onto the reference sequences of six BBTV components (-/+ alphasatellite). Histograms plot the numbers of viral 75 nt sense and antisense reads at each nucleotide position of the 1018-to-1111 nt BBTV genome components (DNAs C, M, N, R, S, U3—subpanels A1-F1) and 1105 nt alphasatellite (Alpha–subpanel G1): blue bars above the axis represent sense reads starting at each respective position, while red bars below the axis represent antisense reads ending at each respective position. The genome organizations of BBTV components and alphasatellite are shown schematically above the respective histograms, with the Pol II promoter (TATA-box and transcription start site, TSS) and terminator (polyA signal, PAS) elements indicated in pink, capped and polyadenylated mRNA shown as solid blue lines, viral protein-coding ORFs boxed and their nucleotide positions given. In each panel (A-G), subpanels 2 and 3 show the Pol II terminator region with the mapped poly(A) sites and the respective poly(A) signals at the upstream positions visualized using MISIS-2 [80]. Note that the mapped mRNA reads ending with oligo(A) tails generate A-SNPs in the sequence logo just downstream of the poly (A) site (i.e., pre-mRNA cleavage and polyadenylation site). (PDF) [file ppat.1010448.s014.pdf]
