# Supplementary material for: A newly emerging alphasatellite affects banana bunchy top virus replication, transcription, siRNA production and transmission by aphids
Source: PLoS Pathog. 2022 Apr 12;18(4):e1010448. doi: 10.1371/journal.ppat.1010448 (PMC9049520; doi:10.1371/journal.ppat.1010448)
Supplement: S14 Fig — For each of the two conditions, i.e. without (BBTV-alpha) and with (BBTV+alpha) alphasatellite, Illumina 75 nt reverse reads of the three biological replicates (leaf tissues of three plants) were combined and mapped simultaneously onto the reference sequences of six BBTV components (-/+ alphasatellite). Histograms plot the numbers of viral 75 nt antisense reads at each nucleotide position of the 1018-to-1111 nt BBTV genome components (DNAs C, M, N, R, S, U3) and 1105 nt alphasatellite (Alpha): red bars below the axis represent antisense reads ending at each respective position. The genome organizations of BBTV components and alphasatellite are shown schematically above the histograms, with the Pol II promoter (TATA-box and transcription start site, TSS) and terminator (polyA signal, PAS) elements indicated in pink, capped and polyadenylated mRNA shown as solid blue lines, viral protein-coding ORFs boxed and their nucleotide positions given. (PDF) [file ppat.1010448.s015.pdf]

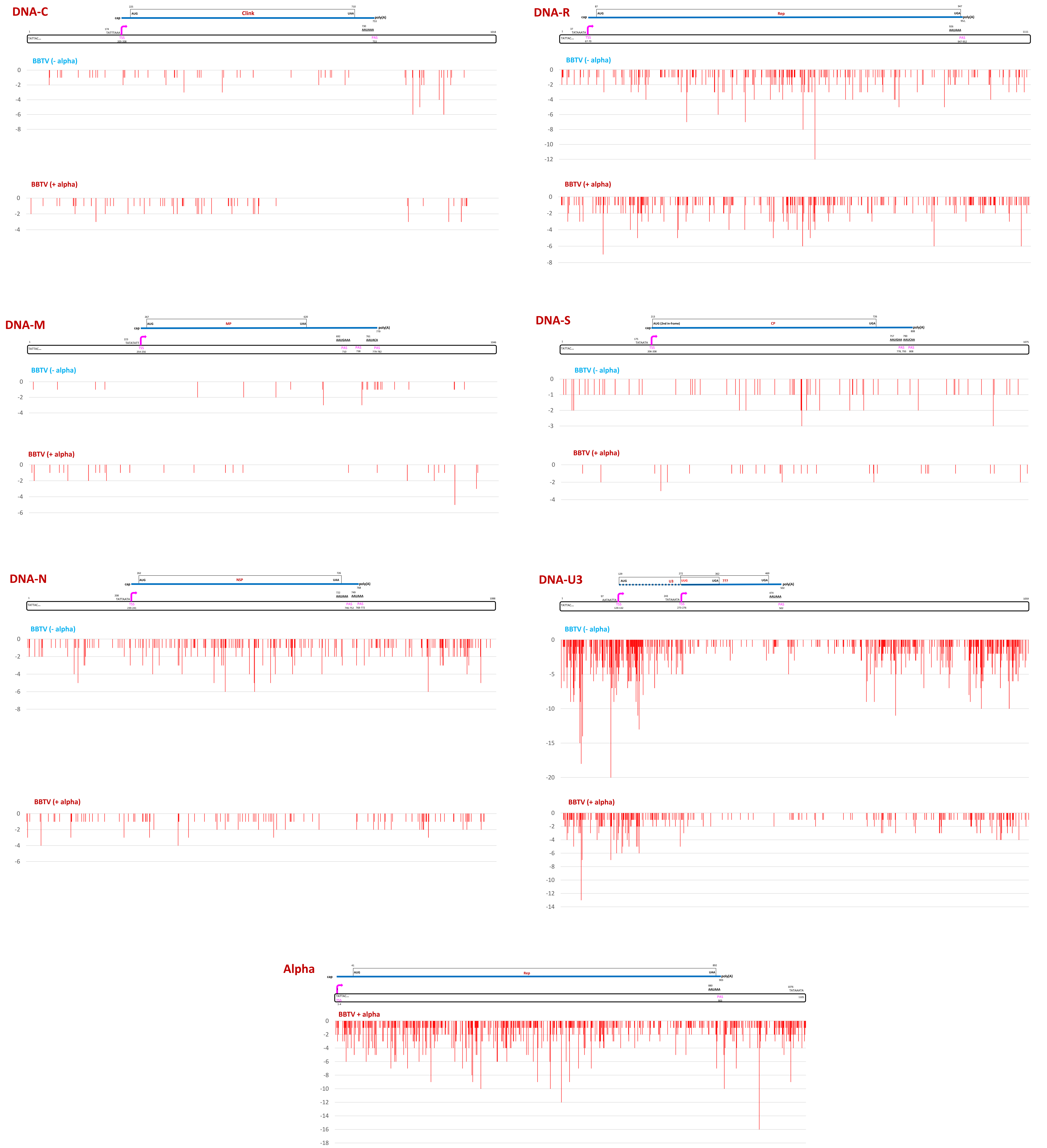


S14 Fig. **Single nucleotide resolution maps of Illumina mRNA-seq reads representing viral antisense transcripts from BBTV-infected Cavendish banana plants with or without DRC alphasatellite.** For each of the two conditions, i.e. without (BBTV-alpha) and with (BBTV+alpha) alphasatellite, Illumina 75 nt reverse reads of the three biological replicates (leaf tissues of three plants) were combined and mapped simultaneously onto the reference sequences of six BBTV components (-/+ alphasatellite). Histograms plot the numbers of viral 75 nt antisense reads at each nucleotide position of the 1018-to-1111 nt BBTV genome components (DNAs C, M, N, R, S, U3) and 1105 nt alphasatellite (Alpha): red bars below the axis represent antisense reads ending at each respective position. The genome organizations of BBTV components and alphasatellite are shown schematically above the histograms, with the Pol II promoter (TATA-box and transcription start site, TSS) and terminator (polyA signal, PAS) elements indicated in pink, capped and polyadenylated mRNA shown as solid blue lines, viral protein-coding ORFs boxed and their nucleotide positions given.
