# Supplementary material for: A newly emerging alphasatellite affects banana bunchy top virus replication, transcription, siRNA production and transmission by aphids
Source: PLoS Pathog. 2022 Apr 12;18(4):e1010448. doi: 10.1371/journal.ppat.1010448 (PMC9049520; doi:10.1371/journal.ppat.1010448)
Supplement: S1 Dataset — (A) Nucleotide sequences of the aphid-borne alphasatellite-BBTV isolate DRC-2016 and its genetic variants in evolving quasispecies population in the Musa acuminata Cavendish recipient plants and in the progenies of the DRC and GAB aphids having transmitted the disease to the infected recipient plants (samples JGF-1-11; S2, S3 and S4 Figs). (B) Nucleotide sequences of the alphasatellite-BBTV isolate DRC-2012 from a leaf sample collected from a BBTD-infected AAB banana plant in Bas Congo province in 2012. (C) Pairwise comparison of the DRC-2016 and DRC-2012 alphasatellite nucleotide sequences and encoded Rep proteins. (D) Protein and nucleotide sequence Blast analyses of DRC alphasatellite isolates. (PDF) [file ppat.1010448.s016.pdf]

S1 Dataset. Complete nucleotide sequences of the alphasatellite-BBTV isolates DRC-2016 (viruliferous aphids from Bas Congo province collected in 2016) and DRC-2012 (a leaf sample of BBTD-infected banana plant in Bas Congo province in 2012) from Democratic Republic of Congo (DRC).

(A) Nucleotide sequences of the aphid-borne alphasatellite-BBTV isolate DRC-2016 and its genetic variants in evolving quasispecies population in the *Musa acuminata* Cavendish recipient plants and in the progenies of the DRC and GAB aphids having transmitted the disease to the infected recipient plants (samples JGF-1-11; see Supplementary Figures S2-S4)

(B) Nucleotide sequences of the alphasatellite-BBTV isolate DRC-2012 from a leaf sample collected from BBTD-infected AAB banana plant in Bas Congo province in 2012

(C) Pairwise comparison of the DRC-2016 and DRC-2012 alphasatellite nucleotide sequences and encoded Rep proteins

(D) Protein and nucleotide sequence Blast analyses of DRC alphasatellite isolates

---

Dataset S1A. Nucleotide sequences of the aphid-borne alphasatellite-BBTV isolate DRC-2016 and its genetic variants in evolving quasispecies population in the *Musa acuminata* Cavendish recipient plants and in the progenies of the DRC and GAB aphids having transmitted the disease to the infected recipient plants (samples JGF-1-11; see Supplementary Figures S4-S5).

DRC-alpha

```
>alphaDRC2016_jgf
TATTACCCCTCCTTGGCACACTGGCACAGCTGACGTACAGATGACGTGTCATCAGCGAAATTGGGTGTTCACACTCAATTTCACAGGAGAGACTCCTATCCTTTCCTTTGGAGAGGAAAC
ACAATATGCGTGTTGGCAACACGAGAAGGTGGATCACGACCACCTACAGGGAGTGATCCAATTGAAGAAGAAGACCCGATTAAACGGAGCGAAGCGACTGATCGGGGGAAATCCACACCT
CGAACCCATGCGAGGTTCGATTACAGAGGCGAAAGCCTACTGTACGAAAGAACAATCACGGATCGCAGGTCCGTGGGAATTCGGAGAAATTCTGCTGAAGGGGTCGAACAGACGGAAGCT
CGCAGAGCTTCTGGATGATCCCGATAACGAAATAAATGAACCTCAAAAATATAGACGAGCGATGGCTAAGTCCGCCATGGATGAATCTCGGAAGCTTGCTGAAGAGTATGATTTCCCTCA
CGAACTACGTTCGTGGCAAAAAACCCTAATTTCATGCCTCGAAGAGGAACCGGATGATCGTACTATTTACTGGGTCTATGGTCCTAATGGAGGAGAAGGTAAAACCCAGTTCGGTAAACA
CCTAGGATTAAAAAAAGGATGGACCTATTTACCCGGAGGGGAACTGAAAGACATGATGTATCTATACAGTAAGGAGTTAAAAAACCATGTGGTTATTGATTTCCCCAGGTGTACGAAAGA
CTTTGTAAGTTATAAATTCCTAGAGATGGTAAAAAACAGAACTGTATATAGTTATAAATACGAACCGATAGGTTCTATAGTCAGTAACAAAGTTCATGTAGTGGTCTTATGTAATTTTAT
GCCGGAAGAAGAAAAAATCTCCGGAGACAGATTAATAATAATAAATTGCTAAACACGCATTTACAAAAATACCCTCGAACAATCAATATTCCTATTATACCCCCGAAGCTCACTTCGGAA
CAACAGCTGTCGTTATCAATGACAGCTGTCATGTGGTCCCAACATAGCGTATGTAATACGCGGACTTGAAAGGATTATTAAATCCGCTTTGCTGCTACAGTGAAGCTTGGCACACTATAA
ATACCTGTGCCAAGGAGAGGGCTAG
```

DNA-C

8 SNPs to KM607057 (**Congo-DRC,** Stainton et al. 2015); **9 SNPs** to JQ820457 (**Malawi;** James et al. 2012). **9 SNPs** to JQ820469 (**Rwanda;** James et al. 2012). **10 SNPs** to JF957676 (**Tonga;** Stainton et al. 2015). **12 SNPs** to KM607023 (**Congo-Brazza;** Stainton et al. 2015). **12 SNPs** to KM607094 (**Samoa;** Stainton et al. 2015); **12 SNPs** to KM607080 (**Hawaii;** Stainton et al. 2015). **12 SNPs** to KM607074 (**Australia;** Stainton et al. 2015). **15 SNPs** to KM607022 (**Burundi;** Stainton et al. 2015). **15 SNPs** to KM607029 (**India;** Stainton et al. 2015).

C protein (161 aa): 99.4% identity (**1 aa**) to 5 isolates: JQ820457 (**Malawi;** James et al. 2012) KM607005 KM607008 KM607043 (**Australia;** Stainton et al. 2015). JN250597 (**Sri Lanka**)

```
>C_jgf
TATTACCCCCAGCGCTCAGGACGGGACATCACGTGCGACTAACAGACGCACGTGAGAATGCAGTAGCTTGCAGCGAAAGATAGACGTCAACATCAATAAAGAAGAAGGAATATTCTTTGC
TTCGGCACGAAGCAAAGGGTATAGATATTTGTTCGAGATGCGAAAATGGAGGCTATTTAAACCTGATGGTTTTGTGATTTCCGAAATCACTCGACGGAAGAGAAATGGAGTTCTGGGAAT
CGTCTGCCATGCCTGACGATGTCAAGAGAGAGATTAAGGAAATATATTGGGAAGATCGGAAGAAACTTCTGTTCTGTCAGAAGTTGAAGAGCTATGTCAGAAGGATTCTTGTTTATGGAG
ATCAAGAGGATGCCCTTGCCGGAGTGAAGGATATGAAGACTTCTATTATTCGCTATAGCGAATACTTGAAGAAACCATGTGTGGTAATCTGTTGTGTTAGCAATAAATCAATTGTGTATA
GGTTAAACAGCATGGTGTTCTTTTATCATGAATACCTTGAAGAACTAGGTGGTGATTACTCAGTATATCAAGATCTCTATTGTGATGAGGTTCTCTCTTCTTCATCGACAGAGGAAGAAG
ATGTAGGAGTAATATATAGGAATGTTATCATGGCATCGACAGAAGAGAAGTTCTCTTGGAGTGATTGTCAGAAGATAGTTATATCAGACTATGATGTAACATTACTCTAATGTAATATCC
ATTATCATCAATAAAATAATGGAATGTTGATTATGTATTTATCATAAATACATAATGGTATACGTATAGCATAAAATACATTAACCAACATACAACACACTATAAAATACAACACACTAT
GACAAAAGTACGGGTATCTGATTGGGCTATATTAACCCCTTAAGGGCCGAAGGCCCGTTTAAATATGTGTTGGACGAAGTCCAAGCACAAAAAAGTAAGCAGAACAACGGAATAATATGA
GCTGGCAACGTAGGGTCCATGTCCCGAGTTAGTGCGCCACGTAAGCGCTGGGGCTTAT
```

DNA-M

7 SNPs to KU759878 (**Congo-DRC,** Mukwa et al. 2016). **10 SNPs** to KM607167 (**Congo-Brazza,** Stainton et al. 2015). **11 SNPs** to JQ820462 (**Rwanda;** James et al. 2012). **13 SNPs** to JQ820456 (**Malawi;** James et al. 2012). **13 SNPs** to KM607237 (**India;** Stainton et al. 2015). **13 SNPs** to KM607241 (**Sri Lanka;** Stainton et al. 2015). **13 SNPs** to KM607222 (**Hawaii;** Stainton et al. 2015). **15 SNPs** to KM607236 (**Samoa;** Stainton et al. 2015). **17 SNPs** to KM607161 (**Burundi;** Stainton et al. 2015). **17 SNPs** to KM607220 (**Australia;** Stainton et al. 2015). **18 SNPs** to JF957669 (**Tonga;** Stainton et al. 2015).

M protein (117 aa) 2 aa to v2: **1 aa** to 5 isolates: JQ820462 JQ820468 (**Rwanda;** James et al. 2012) JQ820456 (**Malawi;** James et al. 2012) FJ609642 EF153738 (**India**) **2 aa** to AF102783 (**Egypt**)

```
>M_jgf_v1
TATTACCCCCAGCGCTCAGGACGGGACATCACGTGCTTCGACAAATGCACGTGAGTGATATAAGGGACATAACGGGTTCAGATAACGGTATCTTTGGTTTAGAATATAACGTCACGTGTG
AAAGTGATAGGCACGTGACTAAGTCAAATGTATTGAATAAACATTTGACGTCCGGTAGCTTCCGAAGGAAGTAAGGATTGCTTCGTGGCGAAGCAAACCATTTATATATTGCCTAGGCTT
GCGGCCTATAAATAGGACCCTGCTAAATGGCATTAACAACAGAGCGGGTTAAACTATTCTTTGAATGGTTTTTGTTCTTTGCAGCAATATTTATTGCGATTACAATATTATATATATTGT
TGGTTTTGCTCTTTGATGTACCGAAGTATATTAAGCAGCTCGTGAGGTATTTGGTAGAATACCTGACCAGAAGACGTGTATGGATGCAGAGAACGCAGTTATCGGAGGCAACTGGAGACG
TAGAGTTCGGCAGAGGTATTGTGGAAGACAGACGGGATCAACAACCGGCTGTCATACCACAGGCATCTCAGGTTAACCCTTCTCAACAAATTAGAAGGGATGATCAAGGAAGACGAGGAA
ACGTCGGACCTATGTTTTAATACACGGTATTGTAATATATGAAATATAAATGGGGATTGATGTATAAGGTCATACATACTATATGTATGTTAATGAAACATATTGTAATATGTGAATTGT
AAACGAGTGTTGAATGTATACAACATACAACACACTATGAAATACAAGACGCTATGACAAAAGTACGGGTATCTGATTAGGTATCCTAACGATCTAGGGCCGAAGGCCCGTGAGCAATAT
GCGTCGAAATAATGTTTAACAAACAAATATACATGATACGGATAGTTGAATACATAAACAACTAGGTTTACAATACAACAAACTGTTGTAAAGAAATAAAAAATAAGAAGGGAGAGTATA
TTTGTGTCGGATAAGCTTGGCAACCACCACTTTAGTGGTGGGTCAGATGTCCCGAGTTAGTGCGCCACGTAAGCGCTGGGGCTTAT
```

M protein (117 aa) 2 aa to v1: **1 aa** to 5 isolates: JQ820462 JQ820468 (**Rwanda;** James et al. 2012) JQ820456 (**Malawi;** James et al. 2012) FJ609642 EF153738 (**India**) **2 aa** to AF102783 (**Egypt**)

```
>M_jgf_v2
TATTACCCCCAGCGCTCAGGACGGGACATCACGTGCTTCGACAAATGCACGTGAGTGATATAAGGGACATAACGGGTTCAGATAACGGTATCTTTGGTTTAGAATATAACGTCACGTGTG
AAAGTGATAGGCACGTGACTAAGTCAAATGTATTGAATAAACATTTGACGTCCGGTAGCTTCCGAAGGAAGTAAGGATTGCTTCGTGGCGAAGCAAACCATTTATATATTGCCTAGGCTT
GCGGCCTATAAATAGGACCCTGCTAAATGGCATTAACAACAGAGCGGGTTAAACTATTCTTTGAATGGTTTTTGTTCTTTGCAGCAATATTTATTGCGATTACAATATTATATATATTGT
TGGTTTTGCTCTTTGAGGTACCGAAGTATATTAAGCAGCTCGTGAGGTATTCGGTAGAATACCTGACAAGAAGACGTGTATGGATGCAGAGAACGCAGTTATCGGAGGCAACTGGAGACG
```

TAGAGTTCGGCAGAGGTATTGTGGAAGACAGACGGGATCAACAACCGGCTGTCATACCACAGGCATCTCAGGTTAACCCTTCTCAACAAATTAGAAGGGATGATCAAGGAAGACGAGGAA
ACGTCGGACCTATGTTTTAATACACGGTATTGTAATATATGAAATATAAATGGGGATTGATGTATAAGGTCATACATACTATATGTATGTTAATGAAACATATTGTAATATGTGAATTGT
AAACGAGTGTTGAATGTATACAACATACAACACACTATGAAATACAAGACGCTATGACAAAAGTACGGGTATCTGATTAGGTATCCTAACGATCTAGGGCCGAAGGCCCGTGAGCAATAT
GCGTCGAAATAATGTTTAACAAACAAATATACATGATACGGATAGTTGAATACATAAACAACTAGGTTTACAATACAACAAACTGTTGTAAAGAAATAAAAAATAAGAAGGGAGAGTATA
TTTGTGTCGGATAAGCTTGGCAACCACCACTTTAGTGGTGGGTCAGATGTCCCGAGTTAGTGCGCCACGTAAGCGCTGGGGCTTAT

DNA-N

6 SNPs to KU759888 (**Congo-DRC,** Mukwa et al. 2016); **10 SNPs** to KM607312 (**Congo-Brazza;** Stainton et al. 2015); **10 SNPs** to KX592200 (**India;** Baldodiya et al. 2015); **11 SNPs** to KM607324 (**Egypt;** Stainton et al. 2015). **11/11/11 SNPs** to KM607352, KM607349, KM607343 (**Congo-DRC;** Stainton et al. 2015);

N protein (154 aa): identical 0 aa to 7 isolates: NC_003476 NP_604479 L41577 (?, **Australia** , ? Burns et al 1995 JGV) AY273170 AY948438 (**India**) EF529519 (**Pakistan**) JF957688 (? Stainton et al. 2012) **1 aa** to KU759884 (**Congo-DRC**), KM607364 (**Sri Lanka**), JF957695 KM607409 (**Tonga**), KM607336 (**Australia**)

>N_jgf
TATTACCCCCGTGCTCGGGACGGGACATGACGTCAGCAATGATTATAATGGGCTTTTTATTAGCCCATCTATTGAATTGGGCCGGGTTTTGTCATTTTACAAAAGCCCGGTCCAGGATA
AGTATAATGTCACGTGCCGAATAAAAGGTTGCTTCGCCTCGAAGAAACCTAATTTGAGGTTGCGTATTCAATACGCTACCGAGTATCTATTAATATGTGAGTCTCTGCCGAAACAATCAG
AGCGAAAGCAAAGCAGAAGCGATGGATTGGGCGGAATCACAATTCAAGACTTGTACTCATGGATGCGATTGGAAGAAGATATCATCGGATTCAGCCGATAATCGACAATATGTGCCATGC
GTCGATTCTGGAGCTGGAAGAAAGTCGCCTCGCAAGGTACTTCTTAGATCTATTGAAGCTGTGTTTAACGGAAGCTTCAGCGGAAATAATAGGAACGTTCGTGGATTTCTCTACGTATCG
ATCAGAGACGATGACGGAGAAATGCGTCCAGTACTCATAGTACCATTCGGTGGATATGGATATCATAATGATTTCTATTATTTCGAAGGAAAGGGGAAAGTTGAATGTGATATATCATCA
GATTATGTTGCGCCAGGAATAGATTGGAGCAGAGACATGGAAGTTAGTATTAGTAACAGCAACAACTGTAATGAATTATGTGATCTGAAGTGTTATGTTGTTTGTTCTTTAAGAATCAAG
GAATAAAAGTTGTGCTGTAATGTTTATTAATAAAACGTATATTTGGGAAATTGATAGTTGTATAAAACATACAACACGCTATGAAATACAAGACGCTATGACAAATGTACGGGTATCTGA
ATGAGTTTTAGTATCGCTTAAGGGCCGCAGGCCCGTTGAAAAATAATAATCGAATTATAAACGTTAGATAATAATCAGAGATAGGTGATCAGATAACATAAACATAAACGAAGTATATGG
CGGTACAATAATAAAATAAGTTAAAAAAAAAAAACATAATGAATACTAATCTCTGATTGGTTCAGAAGAAAGCCCCACCAACTAAAAGGTGGGGAGAATGTCCCGATGACGTAAGCACGG
GGGACTAT

DNA-R

2 SNPs to KU687087 (**Congo-DRC,** Mukwa et al. 2016); **6 SNPs** to JQ820459 (**Rwanda;** James et al. 2012). **6 SNPs** to KM607674 (**India;** Stainton et al. 2015); **7 SNPs** to KM607598 (**Burundi;** Stainton et al. 2015); **8 SNPs** to JQ820453 (**Malawi;** James et al. 2012). **8 SNPs** to KM607658 (**Australia;** Stainton et al. 2015), **9 SNPs** to KM607603 (**Congo-Brazza;** Stainton et al. 2015). **11 SNPs** to KM607680 (**Sri Lanka;** Stainton et al. 2015)

R protein (286 aa): identical 0 aa to 5 isolates: JQ820453 JQ820465 (**Rwanda**) DQ640742 EF584545 GU085264 (**India**) **1 aa** to multiple isolates

>R_jgf
TATTACCCCCAGCGCTCGGGACGGGACATTTGCATCTATAAATAGACCTCCCCCCTCTCCATTACAAGATCATCATCGACGACAGAATGGCGCGATATGTGGTATGCTGGATGTTCACCA
TCAACAATCCCACAACACTACCAGTGATGAGGGATGAGATCAAATATATGGTATATCAAGTGGAGAGGGGACAGGAGGGTACTCGTCATGTGCAAGGTTATGTTGAGATGAAGAGACGAA
GCTCTCTGAAGCAGATGAGAGGCTTCTTCCCAGGCGCACACCTTGAGAAACGAAAGGGAAGCCAAGAAGAAGCGCGGTCATACTGTATGAAGGAAGATACAAGAATCGAAGGTCCCTTCG
AGTTTGGTGCATTTAAATTGTCATGTAATGATAATTTATTTGATGTCATACAGGATATGCGTGAAACGCACAAAAGGCCTTTGGAGTATTTATATGATTGTCCTAACACCTTCGATAGAA
GTAAGGATACATTATACAGAGTACAAGCAGAGATGAATAAAACGAAGGCGATGAATAGCTGGAGAACGTCTTTCAGCGCATGGACATCAGAGGTGGAGAATATCATGGCGCAGCCATGTC
ATCGGAGAATAATTTGGGTCTATGGACCAAATGGAGGAGAAGGAAAGACAACGTATGCAAAACATCTAATGAAGACGAGAAATGCGTTTTATTCTCCAGGAGGAAAATCATTGGATATAT
GTAGACTGTATAATTACGAGGATATTGTTATATTTGATATTCCAAGATGCAAAGAGGATTATTTAAATTATGGGTTATTAGAGGAATTTAAGAATGGAATAATTCAAAGCGGGAAATATG
AACCCGTTTTGAAGATAGTAGAATATGTCGAAGTCATTGTAATGGCTAACTTCCTTCCGAAGGAAGGAATCTTTTCTGAAGATCGAATAAAGTTGGTTTCTTGCTGAACAAGTAATGACT
TTACAGCGCACGCTCCGACAAAAGCACACTATGACAAAAGTACGGGTATCTGATTGGTTTATCTTAACGATCTAGGGCCGTAGGCCCGTGAGCAATGAACGGCGAGATCAGATGTCCCGA
GTTAGTGCGCCACGTAAGCGCTGGGGCTTAT

DNA-S

5 SNPs to KU759877 (**Congo-DRC,** Mukwa et al. 2016); **5/6 SNPs** to JQ820461/JQ820467 (**Rwanda;** James et al. 2012). **6 SNPs** to KM607632 (**India;** Stainton et al. 2015). **6 SNPs** to KM607503 (**Congo-DRC;** Stainton et al. 2015); **7 SNPs** to KM607537 (**Sri Lanka;** Stainton et al. 2015). **8 SNPs** to JQ820456 (**Malawi;** James et al. 2012).

S protein (175 aa) v1=v2: **identical 0 aa** to 5 isolates: EF095164 (**Taiwan**) EF593169 FJ859735 FJ859736 FJ859737 (**Pakistan**) **1 aa** to several isolates incl. JF755985 (**Congo-DRC**)

>S_jgf_v1
TATTACCCCCAGCGCTCGGGACGGGACATGGGCTAATGGATTGTGGATATAGGGCCCAAAGGGCCCGTTTAGATGGGTTTTGGGCTTATGGGCTTTATCCAGAAGACCAAAAACAGGCGG
GAACCGTCCCAAATTCAAACTTCGATTGCTTGCCCTGCAAGCCATCTAGAAGTCTATAAATACCAGTGTGTAGATAGATGTTCAGACAACAAATGGCTAGGTATCCGAAGAAATCCATCA
AGAAGAGGCGGGTTGGGCGCCGGAAGTATGGCAGCAAGGCGGCAACGAGCCACGACTACTCGTCGTTAGGGTCAATATTGGTTCCTGAAAACACCGTCAAGGTATTTCGGATTGAGCCTA
CTGATAAAACATTACCCAGATATTTTATCTGGAAAATGTTTATGCTTCTTGTGTGCAAGGTGAAGCCCGGAAGAATACTTCATTGGGCTATGATCAAGAGTTCTTGGGAAATCAACCAGC
CGACAACCTGTCTGGAAGCTCCAGGTTTATTTATTAAACCTGAACATAGCCATCTGGTTAAACTGGTATGTAGTGGGGAACTTGAAGCAGGAGTCGCAACAGGGACATCAGATGTTGAAT
GTCTTTTGAGGAAGACAACCGTGTTGAGGAAGAATGTAACAGAGGTGGATTATTTATATTTGGCATTCTATTGTAGTTCTGGAGTAAGTATAAACTACCAGAACAGAATTACATATCATG
TTTGATATGTTTATGTAAACATAAACTATTGTATGGAATGAAATCCAAATAACATACAACACGCTATGAAATACAAGACGCTATGACAAAAGTACGGGTATCTGATTAGGTATCCTAACG
ATCTAGGGCCGAAGGCCCGTGAGCAATATGCGTCGAAATAATGTTTAACAAACAAATATACATGATACGGATAGTTGAATACATAAACAACGAGGTATACAATACAACAAACTGTTGTAA
AGAAATAAAAAATAAGAAGAGAGAGTATATTTGTGTCGGATAAGCATGAAACCCACCACTTTAGTGGTGGGTCAGATGTCCCGAGTTAGTGCGCCACGTAAGCGCTGGGGCTTAT

>S_v2
TATTACCCCCAGCGCTCGGGACGGGACATGGGCTAATGGATTGTGGATATAGGGCCCAAAGGGCCCGTTTAGATGGGTTTTGGGCTTATGGGCTTTATCCAGAAGACCAAAAACAGGCGG
GAACCGTCCCAAATTCAAACTTCGATTGCTTGCCCTGCAAGCCATCTAGAAGTCTATAAATACCAGTGTGTAGATAGATGTTCAGACAACAAATGGCTAGGTATCCGAAGAAATCCATCA
AGAAGAGGCGGGTTGGGCGCCGGAAGTATGGCAGCAAGGCGGCAACGAGCCACGACTACTCGTCGTTAGGGTCAATATTGGTTCCTGAAAACACCGTCAAGGTATTTCGGATTGAGCCTA
CTGATAAAACATTACCCAGATATTTTATCTGGAAAATGTTTATGCTTCTTGTGTGCAAGGTGAAGCCCGGAAGAATACTTCATTGGGCTATGATCAAGAGTTCTTGGGAAATCAACCAGC
CGACAACCTGTCTGGAAGCTCCAGGTTTATTTATTAAACCTGAACATAGCCATCTGGTTAAACTGGTATGTAGTGGGGAACTTGAAGCAGGAGTCGCAACAGGGACATCAGATGTTGAAT
GTCTTTTGAGGAAGACAACCGTGTTGAGGAAGAATGTAACAGAGGTGGATTATTTATATTTGGCATTCTATTGTAGTTCTGGAGTAAGTATAAACTACCAGAACAGAATTACATATCATG
TTTGATATGTTTATGTAAACATAAACTATTGTATGGAATGAAATCCAAATAACATACAACACGCTATGAAATACAAGACGCTATGACAAATGTACGGGTATTTGATTGTGTATCTTAACG
ATCTAGGGCCGTAGGCCCGTGAGCAATATGCGTCGAAATAATGTTTAACAAACAAATATACATGATACGGATAGTTGAATACATAAACAACGAGGTATACAATACAACAAACTGTTGTAA
AGAAATAAAAAATAAGAAGAGAGAGTATATTTGTGTCGGATAAGCATGAAACCCACCACTTTAGTGGTGGGTCAGATGTCCCGAGTTAGTGCGCCACGTAAGCGCTGGGGCTTAT

DNA-U3

12 SNPs to KU759869 (**Congo-DRC,** Mukwa et al. 2016); **21 SNPs** to KM607738 (**Congo-Brazza;** Stainton et al. 2015); **22 SNPs** to KM607732 (**Burundi;** Stainton et al. 2015). **22 SNPs** to JQ820454 (**Malawi;** James et al. 2012). **25 SNPs** to JQ820460 (**Rwanda;** James et al. 2012). **25 SNPs** to KM607809 (**Sri Lanka;** Stainton et al. 2015). **29 SNPs** to KM607731 (**India;** Stainton et al. 2015). **33 SNPs** to GQ214699 (**Pakistan;** Hyder et al. 2011); **>50 SNPs** to KM607808 (**China;** Stainton et al. 2015)

U3 protein (77 aa) v1=v2=v3=v4: **identical (0 aa)** to FJ009239 MT109277 (**India**) **1 aa** to several isolates

>U3_jgf_v1

```
TATTACCCCCGTGCTCGGGACGGGACATGGGCTTTTTAAATGGGCCTTGAGAGTTTGAACAGTTCAGTATCTTCGTTATTGGGCCAACTCGGCCCAATAATTAAGAGAACGTGTTCAAA
TTCGTGGTATGACCGAAGGTCAAGGTAACCGGTCAACATTATTCTGGCTTGCGCAGCAAGATACACGAATTAAATTATTAATTCGTAGGACACGTGGACGGACCGAAATACTCCTGCATC
TCTATAAATACCCTAGTCCTGTGAAGGATAATTGCTCTCTCTCTTCTGTCAAGGTGGTTGTGCTGAGGCGGAAGATCGCCAGCGGCGATCGTCGGAACGATGTGCATCTAGAGAGGCGGT
GAAGCAAACTACGAAGCGTATATCGGGTATTTATAGACTTATAGCGCAGCTAGAAGTATACACTGTACAGATTATGTATTTTGTAAATTACGAAGAATTTCGTATATGATATTAATAAAA
CACCTGGGTGTGTTAATGTTTACATTAAATAGTATCCTGAATGTACACAATAAAATACAGTATACGGAACGTATACTGACAAAGTAATAAATGATAGGCGAAGCATGATTAACAGGTGTT
TAGGTATAATTAACATAATTATGAGAAGTAATAATAATACGGAAAATGAATAAGTATGAGGTGAAAGAGGAGATATTAGAATATTTAAAAACCCAATTATATTATTTTGGAACGAAATAC
AACACGCTATGAAATACAAGACGCTATGACAAATGTACGGGTATCTGATTGTATATCTTAACGATCTAGGGCCGTAGGCCCGTGAGCAATGAACGGTCCAGATTAATTCCTTAGCGACGA
AGAAAGGAATCTTAATAGGGACCACATTAAAGACAGCTGTCATTGATTAAATAAATAATATAATATACAAAAGACTTTTGTACCCCTGATAATGATGACGTGTAGGGGTGTCCCGATGTA
ATATAGCATAGCTATGTGAAGAGATAAGCATGTGGCCGACAACTTTAGTGGTGGGCCAGATGTCCCGAGTTAGTGCGCCACGTAAGCACGGGGGACTAT
```

```
>U3_jgf_v2
TATTACCCCCGTGCTCGGGACGGGACATGGGCTTTTTAAATGGGCCTTGAGAGTTTGAACAGTTCAGTATCTTCGTTATTGGGCCAACTCGGCCCAATAATTAAGAGAACGTGTTCAAA
TTCGTGGTATGACCGAAGGTCAAGGTAACCGGTCAACATTATTCTGGCTTGCGCAGCAAGATACACGAATTAAATTATTAATTCGTAGGACACGTGGACGGACCGAAATACTCCTGCATC
TCTATAAATACCCTAGTCCTGTGAAGGATAATTGCTCTCTCTCTTCTGTCAAGGTGGTTGTGCTGAGGCGGAAGATCGCCAGCGGCGATCGTCGGAACGATGTGCATCTAGAGAGGCGGT
GAAGCAAACTACGAAGCGTATATCGGGTATTTATAGACTTATAGCGCAGCTAGAAGTATACACTGTACAGATTATGTATTTTGTAAATTACGAAGAATTTCGTATATGATATTAATAAAA
CACCTGGGTGTGTTAATGTTTACATTAAATAGTATCCTGAATGTACACAATAAAATACAGTATACGGAACGTATACTGACAAAGTAATAAATGATAGGCGAAGCATGATTAACAGGTGTT
TAGGTATAATTAACATAATTATGAGAAGTAATAATAATACGGAAAATGAATAAGTATGAGGTGAAAGAGGAGATATTAGAATATTTAAAAACCCAATTATATTATTTTGGAACGAAATAC
AACACGCTATGAAATACAAGACGCTATGACAAATGTACGGGTATCTGATTGTATATCTTAACGATCTAGGGCCGTAGGCCCGTGAGCAATGAACGGTATAGATTAATTCCTTAGCGACGA
AGAAAGGAATCTTAATAGGGACCACATTAAAGACAGCTGTCATTGATTAAATAAATAATATAATATACAAAAGACTTTTGTACCCCTGATAATGATGACGTGTAGGGGTGTCCCGATGTA
ATATAGCATAGCTATGTGAAGAGATAAGCATGTGGCCGACAACTTTAGTGGTGGGCCAGATGTCCCGAGTTAGTGCGCCACGTAAGCACGGGGGACTAT
```

```
>U3_jgf_v3
TATTACCCCAGCGCTCAGGACGGGACATGGGCTTTTTAAATGGGCCTTGAGAGTTTGAACAGTTCAGTATCTTCGTTATTGGGCCAACTCGGCCCAATAATTAAGAGAACGTGTTCAAA
TTCGTGGTATGACCGAAGGTCAAGGTAACCGGTCAACATTATTCTGGCTTGCGCAGCAAGATACACGAATTAAATTATTAATTCGTAGGACACGTGGACGGACCGAAATACTCCTGCATC
TCTATAAATACCCTAGTCCTGTGAAGGATAATTGCTCTCTCTCTTCTGTCAAGGTGGTTGTGCTGAGGCGGAAGATCGCCAGCGGCGATCGTCGGAACGATGTGCATCTAGAGAGGCGGT
GAAGCAAACTACGAAGCGTATATCGGGTATTTATAGACTTATAGCGCAGCTAGAAGTATACACTGTACAGATTATGTATTTTGTAAATTACGAAGAATTTCGTATATGATATTAATAAAA
CACCTGGGTGTGTTAATGTTTACATTAAATAGTATCCTGAATGTACACAATAAAATACAGTATACGGAACGTATACTGACAAAGTAATAAATGATAGGCGAAGCATGATTAACAGGTGTT
TAGGTATAATTAACATAATTATGAGAAGTAATAATAATACGGAAAATGAATAAGTATGAGGTGAAAGAGGAGATATTAGAATATTTAAAAACCCAATTATATTATTTTGGAACGAAATAC
AACACGCTATGAAATACAAGACGCTATGACAAATGTACGGGTATCTGATTGTATATCTTAACGATCTAGGGCCGTAGGCCCGTGAGCAATGAACGGTCCAGATTAATTCCTTAGCGACGA
AGAAAGGAATCTTAATAGGGACCACATTAAAGACAGCTGTCATTGATTAAATAAATAATATAATATACAAAAGACTTTTGTACCCCTGATAATGATGACGTGTAGGGGTGTCCCGATGTA
ATATAGCATAGCTATGTGAAGAGATAAGCATTTGGCCGACAACTTTAGTGGTGGGCCAGATGTCCCGAGTTAGTGCGCCACGTAAGCGCTGGGGCTTAT
```

```
>U3_jgf_v4
TATTACCCCCGTGCTCGGGACGGGACATGGGCTTTTTAAATGGGCCTTGAGAGTTTGAACAGTTCAGTATCTTCGTTATTGGGCCAACCCGGCCCAATAATTAAGAGAACGTGTTCAAA
TTCGTGGTATGACCGAAGGTCAAGGTAACCGGTCAACATTATTCTGGCTTGCGCAGCAAGATACACGAATTAAATTATTAATTCGTAGGACACGTGGACGGACCGAAATACTCCTGCATC
TCTATAAATACCCTAGTCCTGTGAAGGATAATTGCTCTCTCTCTTCTGTCAAGGTGGTTGTGCTGAGGCGGAAGATCGCCAGCGGCGATCGTCGGAACGATGTGCATCTAGAGAGGCGGT
GAAGCAAACTACGAAGCGTATATCGGGTATTTATAGACTTATAGCGCAGCTAGAAGTATACACTGTACAGATTATGTATTTTGTAAATTACGAAGAATTTCGTATATGATATTAATAAAA
CACCTGGGTGTGTTAATGTTTACATTAAATAGTATCCTGAATGTACACAATAAAATACAGTATACGGAACGTATACTGACAAAGTAATAAATGATAGGCGAAGCATGATTAACAGGTGTT
TAGGTATAATTAACATAATTATGAGAAGTAATAATAATACGGAAAATGAATAAGTATGAGGTGAAAGAGGAGATATTAGAATATTTAAAAACCCAATTATATTATTTTGGAACGAAATAC
AACACGCTATGAAATACAAGACGCTATGACAAATGTACGGGTATTTGATTGTGTATCTTAACGATCTAGGGCCGTAGGCCCGTGAGCAATGAACGGTATAGATTAATTCCTTAGCGACGA
AGAAAGGAATCTTAATAGGGACCACATTAAAGACAGCTGTCATTGATTAAATAAATAATATAATATACAAAAGACGTTTGTACCCCTGATAATGATGACGTGTAGGGGTGTCCCGATGTA
ATATAGCATAGCTATGTGAAGAGATAAGCATGTGGCCGACAACTTTAGTGGTGGGCCAGATGTCCCGAGTTAGTGCGCCACGTAAGCACGGGGGACTAT
```

Dataset S1B. Nucleotide sequences of the alphasatellite-BBTV isolate DRC-2012 from a leaf sample collected from BBTD-infected AAB banana plant in village Kimpoko (Bas Congo province) in 2012 (sample ALYU-21 and its technical replicate ALYU-20).

DNA-alpha

```
>alphaDRC2012_alyu21
TATTACCCCTCCTTGGCACACTGGCACAGATGACGTGTCATCAGCGAAATTGGGTGTTCACACTCAATTTCACAGGAGAGACTCCTATCCTTTCCTTTGGAGAGGAAACACAATATGCGT
GTTGGCAACACGAGAAGGTGGATCACGACCACCTACAGGGAGTGATCCAATTGAAGAAGAAGACCCGATTAAACGGAGCGAAGCGACTGATCGGGGGAAATCCACACCTCGAACCCATGC
GAGGTTCAATTACAGAGGCGAAAGCCTACTGTACGAAAGAACAATCACGGATCGCAGGTCCGTGGGAATTCGGAGAAATGCTGCTGAAGGGGTCGAACAGACGGAAGCTCGCAGAGCTTC
TGGATGATCCCGATAACGAAATAAATGAACCTCAAAAATATAGACGAGCGATGGCTAAGTCCGCCATGGATGAATCTCGGAAGCTTGCTGAAGAGTATGATTTCCCTCACGAACTACGCT
CGTGGCAAAAAACCCTAATCTCATTCCTCGAAGAGGAACCGGATGATCGTACTATTTACTGGGTCTATGGTCCTAATGGAGGAGAAGGTAAAACCCAGTTCGGTAAACACCTGGGATTAA
AAAAAGGATGGACCTATTTACCCGGAGGGGAACTGAAAGACATGATGTATCTATACAGTAAGGAGTTAAAAAACCATGTGGTTATTGATTTCCCCAGATGTACGAAAGACTTTGTAAGTT
ATAAATTCCTAGAGATGGTAAAAAATAGAACTGTATATAGTTATAAATACGAACCGATAGGTTCTATAGTCAGTAACAAAGTTCATGTAGTGGTATTATGTAATTTCCTGCCGGAAGAAG
AAAAAATCTCCGGAGACAGATTAATAATAATAAATTGCTAAACACGCATTTACAAATAACACCCTCGAACAATCAATATTTCTATTATACCCCCGAAGCTCACTTCGGAACAACAGCGAC
AGCTGTCAACATCAGCGTTAGCTGTCATGTGGGCCCAACATAGCGTATGTAATACGCGGACTTGAAAGGATTATTAAATCCGCTTTGCTGCTACAGTGAAGCTTGGCACACTATAAATAC
CTGTGCCAAGGAGAGGGCTAG
```

DNA-C

7 SNPs to KU759879 (**Congo-DRC,** Mukwa et al. 2015); **9 SNPs** to JQ820469 (**Rwanda;** James et al. 2012). **9 SNPs** to JQ820457 (**Malawi;** James et al. 2012)**, 10 SNPs** to KM607029 (**India;** Stainton et al. 2015). **12 SNPs** to KM607090 (**Tonga;** Stainton et al. 2015). **11 SNPs** to KM607023 (**Congo-Brazza;** Stainton et al. 2015). **13 SNPs** to KM607020 (**Burundi;** Stainton et al. 2015). **14 SNPs** to KM607074 (**Australia;** Stainton et al. 2015). **16 SNPs** to KM607094 (**Samoa;** Stainton et al. 2015); **16 SNPs** to KM607080 (**Hawaii;** Stainton et al. 2015)

C protein (161 aa): 100% identity (**0 aa**) to 5 isolates: JQ820457 (**Malawi;** James et al. 2012), KM607005, KM607008, KM607043 (**Australia;** Stainton et al. 2015), JN250597 (**Sri Lanka**)

```
>C_alyu21
TATTACCCCCAGCGCTCAGGACGGGACATCACGTGCGACTAACAGACGCACGTGAGAATGCAGTAGCTTGCAGCGAAAGATAGACGTCAACATCAATAAAGAAGGAGGAATATTCTTTGC
TTCGGCACGAAGCAAAGGGTATAGATATTTGTTCGAGATGCGAAAATGGAGGCTATTTAAACCTGATGGTATTGTGATTTCCGAAATCACTCGACGGAAGAGAAATGGAGTTCTGGGAAT
CGTCTGCCATGCCTGACGATGTCAAGAGAGAGATTAAGGAAATATATTGGGAAGATCGGAAGAAACTTCTGTTCTGTCAGAAGTTGAAGAGCTATGTCAGAAGGATTCTTGTTTATGGAG
ATCAAGAGGATGCCCTTGCCGGAGTGAAGGATATGAAGACTTCTATTATTCGCTATAGCGAATACTTGAAGAAACCATGTGTGGTAATTTGTTGTGTTAGCAATAAATCAATTGTGTATA
GGTTAAACAGCATGGTGTTCTTTTATCATGAATACCTTGAAGAACTAGGTGGTGATTACTCAGTATATCAAGATCTCTATTGTGATGAGGTTCTTTCTTCTTCATCGACAGAGGAAGAAG
ATGTAGGAGTAATATATAGGAATGTTATCATGGCATCGACACAAGAGAAGTTCTCTTGGAGTGATTGTCAGAAGATAGTTATATCAGACTATGATGTAACATTACTCTAATGTAATATCC
ATTATCATCAATAAAATAATGGAATGTTGATTATGTATTTATCATAAATACATAATGGTATACGTATAGCATAAAATACATTATCCAACATACAACACACTATAAAATACAACACACTAT
AACAAATGTAAGGGTATTTGATTGGTTTATATTAACCCCTTAAGGGCCGAAGGCCCGTTTAAATATGTGTTGGACGAAGTCCAAGCACAAAAAAGTAAGCAGAACAACGGAATAATATGA
GCTGGCAACGTAGGGTCCATGTCCCGAGTTAGTGCGCCACGTAAGCGCTGGGGCTTAT
```

DNA-M

7 SNPs to JQ820462 (**Rwanda;** James et al. 2012)**, 8 SNPs** to KM607205 (**Congo-DRC,** Stainton et al. 2015); **8 SNPs** to KM607167 (**Congo-Brazza;** Stainton et al. 2015); **9 SNPs** to JQ820456 (**Malawi;** James et al. 2012)**, 9 SNPs** to KM607241 (**Sri Lanka;** Stainton et al. 2015); **9 SNPs** to KM607222 (**Hawaii;** Stainton et al. 2015). **11 SNPs** to KM607237 (**India;** Stainton et al. 2015). **11 SNPs** to KM607236 (**Samoa;** Stainton et al. 2015). **13 SNPs** to KM607220 (**Australia;** Stainton et al. 2015). **13 SNPs** to KM607161 (**Burundi;** Stainton et al. 2015). **14 SNPs** to JF957669 (**Tonga;** Stainton et al. 2015)

M protein (117 aa): identical **0 aa** to 5 isolates: JQ820462 JQ820468 (**Rwanda**; James et al. 2012) JQ820456 (**Malawi**; James et al. 2012) FJ609642 EF153738 (**India**) **1 aa** to AF102783 (**Egypt**)

>M_alyu21
TATTACCCCCAGCGCTCAGGACGGGACATCACGTGCTTCGACAAATGCACGTGAGTGATATAAGGGACATAACGGGTTCAGATAACGGTATCTTTGGTTTAGAATATAACGTCACGTGTG
AAAGTGATAGGCACGTGACTAAGTCAAATGTATTGAATAAACATTTGACGTCCGGTAGCTTCCGAAGGAAGTAAGGATTGCTTCGTGGCGAAGCAAACCATTTATATATTGCCTAGGCTT
GCGGCCTATAAATAGGACCCTGCTAAATGGCATTAACAACAGAGCGGGTTAAACTATTCTTTGAATGGTTTTTGTTCTTTGCAGCAATATTTATTGCGATTACAATATTATATATATTGT
TGGTTTTGCTCTTTGAGGTACCGAAGTATATTAAGCAGCTCGTGAGGTATTTGGTAGAATACCTGACCAGAAGACGTGTATGGATGCAGAGAACGCAGTTATCGGAGGCAACTGGAGACG
TAGAGTTCGGCAGAGGTATTGTGGAAGACAGACGGGATCAACAACCGGCTGTCATACCACAGGCATCTCAGGTTAACCCTTCTCAACAAATTAGAAGGGATGATCAAGGAAGACGAGGAA
ACGTCGGACCTATGTTTTAATACACGGTATTGTAATATATGAAATATAAATGGGGATTGATGTATAAGGTCATACATACTATATGTATGATAATGAAACATATTGTAATATGTGAATTGT
AAACGAGTGTTGAATGTATACAACATACAACACACTATGAAATACAAGACGCTATGACAAATGTATGGGTATCTGATTAGGTATCCTAACGATCTAGGGCCGAAGGCCCGTGAGCAATAT
GCGTCGAAATAATGTTTAACAAACAAATATACATGATACGGATAGTTGAATACATAAACAAcTAGGTATACAATACAACAAACTGTTGTAAAGAAATTAAAAATAAGAAGAGAGAGTATA
TTTGTGTCGGATAAGCTTGGCAACCACCACTTTAGTGGTGGGTCAGATGTCCCGAGTTAGTGCGCCACGTAAGCGCTGGGGCTTAT

DNA-N

9 SNPs to KU759880 (**Congo-DRC**, Mukwa et al. 2016); **10 SNPs** to KM607312 (**Congo-Brazza**; Stainton et al. 2015); **10 SNPs** to JQ820470 (**Rwanda**; James et al. 2012). **11 SNPs** to JQ820458 (**Malawi**; James et al. 2012), **11 SNPs** to KM607311 (**Burundi**; Stainton et al. 2015). **12 SNPs** to KX592200 (**India**; Baldodiya et al. 2017); **12 SNPs** to KM607324 (**Egypt**; Stainton et al. 2015). **14 SNPs** to KM607388 (**Sri Lanka**; Stainton et al. 2015); **18 SNPs** to JX170761 (**Pakistan**; Ali et al. 2012); **27 SNPs** to KM607359 (**Australia**; Stainton et al. 2015)

N protein (154 aa): identical 0 aa to 7 isolates: NC_003476 NP_604479 L41577 (?, **Australia** , ? Burns et al 1995 JGV) AY273170 AY948438 (**India**) EF529519 (**Pakistan**) JF957688 (? Stainton et al. 2012) **1 aa** to KU759884 (**Congo-DRC**) KM607364 (**Sri Lanka**) JF957695 KM607409 (**Tonga**) KM607336 (**Australia**)

>N_alyu21
TATTACCCCCCGTGCTCGGGACGGGACATGACGTCAGCAAGGAATATAATGGGCTTTTTATTAGCCCATGTATTGAATTGGGCCGGGTTTTGTCATTTTACAAAAGCCCGGTCCAGGATA
TGTATAATGTCACGTGCCGAATAAAAGGTTGCTTCGCCTCGAAGAAACCTAATTTGAGGTTGCGTATTCAATACGCTACCGAGTATCTATTAATATGTGAGTCTCTGCCGAAACAATCAG
AGCGAAAGCAAAGCAGAAGCGATGGATTGGGCGGAATCACAATTCAAGACTTGTACTCATGGATGCGATTGGAAGAAGATATCATCGGATTCAGCCGATAATCGACAATATGTGCCATGC
GTCGATTCTGGAGCTGGAAGAAAGTCGCCTCGCAAGGTACTTCTTAGATCTATTGAAGCTGTGTTTAACGGAAGCTTCAGCGGAAATAATAGGAACGTTCGTGGATTTCTCTACGTATCG
ATCAGAGACGATGACGGAGAAATGCGTCCAGTACTCATAGTACCATTCGGAGGATATGGATATCATAATGATTTCTATTATTTCGAAGGAAAGGGGAAAGTTGAATGTGATATATCATCA
GATTATGTTGCGCCAGGAATAGATTGGAGCAGAGACATGGAAGTTAGTATTAGTAACAGCAACAACTGTAATGAATTATGTGATCTGAAGTGTTATGTTGTTTGTTCTTTAAGAATCAAG
GAATAAAAGTTGTGCTGTAATGTTTATTAATAAAACGTATATTTGGGAAATTGATAGTTGTATAAAACATACAACACGCTATGAAATACAAGACGCTATGACAAATGTACGGGTATCTGA
ATGAGTTTTAGTATCGCTTAAGGGCCGCAGGCCCGTTGAAAAATAATAATCGAATTATAAACGTTAGATAATAATCAGAGATAGGTGATCAGATAACATAAACATAAACGAAGTATATGG
CGGTACAATAATATAATAAGTTAAAAATAAAAAACATAATGAATACTAATCTCTGATTGGTTCAGAAGAAAGGCCCACCAACTAAAAGGTGGGGAGAATGTCCCGATGACGTAAGCACGG
GGGACTAT

DNA-R

3 SNPs to KU687068 (**Congo-DRC**, Mukwa et al. 2016); **4 SNPs** to KM607674 (**India**; Stainton et al. 2015); **5 SNPs** to KM607598 (**Burundi**; Stainton et al. 2015); **6 SNPs** to JQ820459 (**Rwanda**; James et al. 2012). **6 SNPs** to KM607658 (**Australia**; Stainton et al. 2015), **7 SNPs** to KM607603 (**Congo-Brazza**; Stainton et al. 2015). **9 SNPs** to KM607680 (**Sri Lanka**; Stainton et al. 2015);

R protein (286 aa): identical 0 aa to 5 isolates JQ820453 JQ820465 (**Rwanda**) DQ640742 EF584545 GU085264 (**India**) **1 aa** to KU687088 KU687089 (**Congo-DRC**) JQ820459 (**Rwanda**) KP876489 KP876490 KP876491 GU125417 DQ656118 DQ656119 (**India**) …

>R_alyu21
TATTACCCCCAGCGCTCGGGACGGGACATTTGCATCTATAAATAGACCTCCCCCCTCTCCATTACAAGATCATCATCGACGACAGAATGGCGCGATATGTGGTATGCTGGATGTTCACCA
TCAACAATCCCACAACACTACCAGTGATGAGGGATGAGATCAAATATATGGTATATCAAGTGGAGAGGGGACAGGAGGGTACTCGTCATGTGCAAGGTTATGTCGAGATGAAGAGACGAA
GCTCTCTGAAGCAGATGAGAGGCTTCTTCCCAGGCGCACACCTTGAGAAACGAAAGGGAAGCCAAGAAGAAGCGCGGTCATACTGTATGAAGGAAGATACAAGAATCGAAGGTCCCTTCG
AGTTTGGTGCATTTAAATTGTCATGTAATGATAATTTATTTGATGTCATACAGGATATGCGTGAAACGCACAAAAGGCCTTTGGAGTATTTATATGATTGTCCTAACACCTTCGATAGAA
GTAAGGATACATTATACAGAGTACAAGCAGAGATGAATAAAACGAAGGCGATGAATAGCTGGAGAACTTCTTTCAGTGCATGGACATCAGAGGTGGAGAATATCATGGCGCAGCCATGTC
ATCGGAGAATAATTTGGGTCTATGGACCAAATGGAGGAGAAGGAAAGACGACGTATGCAAAACATCTAATGAAGACGAGAAATGCGTTTTATTCTCCAGGAGGAAAATCATTGGATATAT
GTAGACTGTATAATTACGAGGATATTGTTATATTTGATATTCCAAGATGCAAAGAGGATTATTTAAATTATGGGTTATTAGAGGAATTTAAGAATGGAATAATTCAAAGCGGGAAATATG
AACCCGTTTTGAAGATAGTAGAATATGTCGAAGTCATTGTAATGGCTAACTTCCTTCCGAAGGAAGGAATCTTTTCTGAAGATCGAATAAAGTTGGTTTCTTGCTGAACAAGTAATGACT
TTACAGCGCACGCTCCGACAAAAGCACACTATGACAAAAGTAGGGGTATCTGATTGGGTTATCTTAACGATCTAGGGCCGTAGGCCCGTGAGCAATGAACGGCGAGATCAGATGTCCCGA
GTTAGTGCGCCACGTAAGCGCTGGGGCTTAT

DNA-S

6 SNPs to KU759889 (**Congo-DRC**, Mukwa et al. 2016); **6 SNPs** to JQ820461 (**Rwanda**; James et al. 2012). **7 SNPs** to JQ820455 (**Malawi**; James et al. 2012). **7 SNPs** to KM607532 (**India**; Stainton et al. 2015). **8 SNPs** to KM607514 (**Hawaii**; Stainton et al. 2015); **10 SNPs** to KM607451 (**Burundi**; Stainton et al. 2015). **10 SNPs** to KM607537 (**Sri Lanka**; Stainton et al. 2015). **10 SNPs** to KM607528 (**Samoa**; Stainton et al. 2015); **12 SNPs** to KM607510 (**Australia**; Stainton et al. 2015); **12 SNPs** to KM607471 (**Egypt**; Stainton et al. 2015). **14 SNPs** to KM607573 (**Tonga**; Stainton et al. 2015);

S protein (175 aa): identical 0 aa to 5 isolates: EF095164 (**Taiwan**) EF593169 FJ859735 FJ859736 FJ859737 (**Pakistan**), **1 aa** to several isolates

>S_alyu20_v1
TATTACCCCCAGCGCTCGGGACGGGACATGGGCTAATGGATTGTGGATATAGGGCCCAAAGGGCCCGTTTAGATGGGTTTTGGGCTTATGGGCTTTATCCAGAAGACCAAAAACAGGCGG
GAACCGTCCCAAATTCAAACTTCGATTGCTTGCCCTGCAAGCCATCTAGAAGTCTATAAATACCAGTGTCTAGATAGATGTTCAGACAACAAATGGCTAGGTATCCGAAGAAATCCATCA
AGAAGAGGCGGGTTGGGCGCCGGAAGTATGGCAGCAAGGCGGCAACGAGCCACGACTACTCGTCGTTAGGGTCAATATTGGTTCCTGAAAACACCGTCAAGGTATTTCGGATTGAGCCTA
CTGATAAAACATTACCCAGATATTTTATCTGGAAAATGTTTATGCTTCTTGTGTGCAAGGTGAAGCCCGGAAGAATACTTCATTGGGCTATGATCAAGAGTTCTTGGGAAATCAACCAGC
CGACAACCTGTCTGGAAGCCCCAGGTTTATTTATTAAACCTGAACATAGCCATCTGGTTAAACTGGTATGTAGTGGGGAACTTGAAGCAGGAGTCGCAACAGGGACATCAGATGTTGAAT
GTCTTTTGAGGAAGACAACCGTGTTGAGGAAGAATGTAACAGAGGTGGATTATTTATATTTGGCATTCTATTGTAGTTCTGGAGTAAGTATAAACTACCAGAACAGAATTACATATCATG
TTTGATATGTTTATGTAAACATAAACTATTGTATGGAATGAAATCCAAATAACATACAACACGCTATGAAATACAAGACGCTATGACAAAAGTACGGGTATCTGATTAGGTATCCTAACG
ATCTAGGGCCGAAGGCCCGTGAGCAATATGCGTCGAAATAATGTTTAACAAACAAATATACATGATACGGATAGTTGAATACATAAACAACGAGGTATACAATACAACAAACTGTTGTAA
AGAAATAAAAAACAAGAAGAGAGAGTATATTTGTGTCGGATAAGCATGAAACCCACCACTTTAGTGGTGGGTCAGATGTCCCGAGTTAGTGCGCCACGTAAGCGCTGGGGACTAT

DNA-U3

29 SNPs to KM607738 (**Congo-Brazza**; Stainton et al. 2015); **30 SNPs** to KM607773 (**Congo-DRC**, Stainton et al. 2015); **30 SNPs** to JQ820454 (**Malawi**; James et al. 2012). **31 SNPs** to KM607732 (**Burundi**; Stainton et al. 2015). **32 SNPs** to KM607809 (**Sri Lanka**; Stainton et al. 2015). **37 SNPs** to KM607731 (**India**; Stainton et al. 2015). **36 SNPs** to JQ820466 (**Rwanda**; James et al. 2012). **45 SNPs** to FJ859750 (**Pakistan**; Hyder et al. 2011); **>60 SNPs** to KM607808 (**China**; Stainton et al. 2015)

U3 protein (77 aa): 1 aa to FJ009239 MT109277 (**India**) **2 aa** to several isolates

>U3_alyu21
TATTACCCCCAGCGCTCGGGACGGGACATGGGCTTTTTAAATGGGCCTTGAGAGTTTGAACAGTTCAGTATCTTCGTTATTGGGCCAACTCGGCCCAATAATTAAGAGAACGTGTTCAAA
TTCGTGGTATGACCGAAGGTCAAGGTAACCGGTCAACATTATTCTGGCTTGCGCAGCAAGATACACGAATTAAATTATTAATTCGTAGGACACGTGGACGGACCGAAATACTCCTGCATC
TCTATAAATACCCTAGTCATGTGAAGGATAATTGCTCTCTCTCTTCTGTCAAGGTGGTTGTGCTGAGGCGGAAGATCGCCAGCGGCGATCGTCGGAACGATGTGCATCTAGAGAGGCGGT

```
GAAGCAAACTACGAAGCATATATCGGGTATTTATAGACTTATAGCGCAGCTAGAAGTATACACTGTACAGATTTTGTATTTTGTAAATTACGAAGAATTTCGTATATGATATTAATAAAA
CACCTGGGTGTGTTAATGTTTACATTAAATTGTATCCTGAATGTACACAATAAAATACAGTATACGGAACGTATACTGACAAAGTAATAAATGATAGGCGAAGCATGATTAACAGGTGTT
TAGGTATAATTAACATAATTATGAGAAGTAATAATAATACGGAAAATGAATAAGTATGAGGTGAAAGAGGAGATATTAGAATATTTAAAAACCCAATTATATTATTTTGGAACGAAATAC
AACACACTATGAAATACAAGGCGCTATGACAAATGTACGGGTATTTGATTGTGTATCTTAACGCTTAAGGGCCGCAGGCCCGTCAAGTTGAATGAACGGTCTAGATTAATTCCTTAGCGA
CGAAGAAAGGAATCTTAGTGGGGACCATATTAAAGACAGCTGTCATTGATTAAATAAATAATATAATATaCAAAAGACTATTGTACCCCTGATAATGATGACGTGTAGGGGTGTCCCGAT
GTAATATAGCATAGCTATGTGAAGAGATAAGCATGTGGCCGACAACTTTAGTGGTGGGCCAGATGTCCCGAGTTAGTGCGCCACGTAAGCGCTGGGGACTAT
```

Acinetobacter plasmid-like

Rep protein (423 aa) best match to 421 aa rep of Acinetobacter soli plasmid-like

```
>Acinetobacter plasmid-like_alyu21
TTACTTGATACTATTAATTAACTCGGGGACAAAATTTGATGCAAAAAGACATTAAAAAACCCCTGTTATCTCACAGTTTGGCGACCAGCGATAACAAGGGTTTGGAATCTGCTAAGGCAG
ATCAACATAGGGATAGAATAACACGTTTTGGCATTTTGAAACATAGATCGAAGCAACAAGAAAATTATCTATGGACACTAGCCAAATACAAGGAAAACTATCAAAACGATAAGCCAAACA
ACGAGTCCATTAGGGCCACTAAAGCAGCTCATAAATTGCAGTCCTGCGGCAATTTTCTTTTGTTCAAAAATTTTTACACAATTGACCAAGTCAAGCTCGCAAAATTTCATGTTTGTGGTC
AGCACTTACTTTGCCCATTTTGTGCGGGTATTCGCGCATCTAAAGCAATTCAAAAATATACTGAACGAGTTAATGAGGTTCTTTCTAAAAATCGTAAACTCAAGCCTGTTTTAATCACTT
TCACAGTAAAAAATGGTTCCGATCTCGATGAGCGATCATCTCATTTAATGAAGTCATTCAGAACTCTTCTCGATCGACGTAGGGATTTTTTAAAAAAAGGTCGTGGTTTAATGAGTTCT
GCAAAATTAATGGTGCTATGTATTCGTATGAAAATACTTTTAATCCAGATACCAAAGAATGGCATCCACATGTTCACATGTTTGCCCTTTTAGATTCTTGGATTGATCAAGATGAGCTAT
CACAGTATTGGCATTCTATTACTGGCGATTCAATGATTGTCGATATACGACGAGTTAAAAAACAAAAAGATGTTGGTTATGCAAAAGCTGCCGCTGAAGTTTGTAAATACGCCCTTAAGT
TCGGCGATTTATCTGTTGAAAATACTTGGGAAGCATTCAAGGTACTCAAAGGCAAGCGTTTAAGCGGTGCATTTGGATCTCTATACGGTGTAAAAATTCCTGACAATCTTGCTGACGAAA
TGCCAGATGAAAACGATCTGCCATATTTAGAAATGCTCTACAAATTTGTTTTTGGAAAACAGTCCTACTATGACTTAACCATGACACGACACGTAGAGCCGCAATGCAAGGACATGCATG
ACGAGGATGGAGTAGGAGGCACGACGCCCGACCGACGAGTTGTGCATGGGCGAAGCAGTGAGGCGATAGAGGACGATGCGAGGACGTTGCCGGTGCGAGGGCCCGCAGTCACGGAACGAC
CACAGCGAGTCCGCAAAAAGCAACATTGGCAACTACCACCAACAACCCGTGTGAGGGTTCGGCAACGAATCCGAAGATGGGACGGTTATTTGTGTGTTATACACATGTGATTTTAAAAAC
GTATGTTAAAATTTAGTCGCTGAATAGCAGGACGGCCATCCTACTACCCAGCTAACCAAAACGATCTATTAAGGAGATCATCATGGCTGTACCGAATACTAATGCTTTAGATTATTTAGG
CAAGTATGTTTCATTTAATCACGACGATATTTTTGAAATATACGGAGTTATTTCCAATGTAATTTTTAATATGGATGGCTCTGTTGAAATGTCAACTGGTTGGGATAATTTTTACGAGCT
CTCTAAAATGAGCAATTTAAAAATTTTGGGCGAAATTAGACTCTATTGATTTTATTGAGCCGCACATAAGTACTGCACATAATAGCTGTTATGTGCAGTATTTTTTTGCCCGTTTCTGGA
GAAACGCGTAAGCGTGCATTCTGACAAAAATTGCCGAACGAGTGTCTACGAGTGACGACTCCAGAATAAAAATTTCAAACCTTCCTTTCCCTGATTACAAGCTCCCATTTGCATTTGACG
GCAAGTACAGAGCCATCCCGCATGGGTGGCGAAGTAATGATATTGATTTAATATCATTAGTGATATATAAATATAAAACCAATTATGTCTATGAACATATCAATTTAATATTAGGTGATA
AAATGGAACCAACTAATTTACAAATCTTCGTTGCTGAAGTTAAAGGCACTGGTGAATCGGAATATATCGGCGTTTATAAGCAAGTGCCTTTGCGTCTTCGTGCAGGTGTTTTTGCTGAAG
TTGAAGCACTTCAGGAAATGATGGCTCGAACTCAGAAGGTATCTCGAAATAAGGTTATCAATGATCTCCTTCAAATCGCTATTGATCAGGTTAAAGTCTCACTGGATGAGAAATCTCTAG
AAGATTTCAATATGTTTGCTTCCCATCACTACAGTGATTTCACTGGCTCAGGTGACTTGTCAGATGAATCGTAAAATTGAAGTTTTAGGCGGTGGTCCAATGGAATCTCTTGAAGAAGCT
GCTGCTTACTTCAAGAAGGTTCTCCAGGAACATTTGGATAATAAAAGAGACATAACACTCTTTGCGATTGCCGTTGTAGGCGGTCGCTTAAAAGTAGTTGAGGGATTACCGAAATGATTA
AATTGGAAGGCACAATTATTAATGTTTTCACCCAACAGGGTGGTAAAGATAAAAAGACTGGAGAGGCATTTGCCGATCGAGACAAAATTCAACTTTTAGGTGAGCTTGACTTGCCGAATG
GAGATAAAAAAACACGAGTTGATTGATCTTACTGTCGAAGACGGTAAGCATTATGAGAGCTTAAAAAATAAACGAGTTAGTATTCCATGCGGTGCTTTGGCATCTGGAAGAAATGTCATTT
TTTATGTTGCAAAAGGCGCAACTCCAAAACTACTGAGTTCAGCATGATACTCGATAAAAAACAGCAACTTACGAAGTAAGTTCGCTGTTTTTTCTT
```

Dataset S1C. Pairwise comparison of the DRC-2016 and DRC-2012 alphasatellite nucleotide sequences and encoded Rep proteins.

EMBOSS Needle

```
# Aligned_nucleotide_sequences: 2
# 1: alphaDRC2016
# 2: alphaDRC2012
# Matrix: EDNAFULL
# Gap_penalty: 10.0
# Extend_penalty: 0.5
# Length: 1112
# Identity:     1073/1112 (96.5%)
# Similarity:   1073/1112 (96.5%)
# Gaps:           18/1112 ( 1.6%)
# Score: 5243.5

alphaDRC2016       1 TATTACCCCTCCTTGGCACACTGGCACAGCTGACGTACAGATGACGTGTC     50    Rep ORF start codon
                     |||||||||||||||||||||||||           ||||||||||||||
alphaDRC2012       1 TATTACCCCTCCTTGGCACACTGGC-----------ACAGATGACGTGTC     39    Rep ORF start codon

alphaDRC2016      51 ATCAGCGAAATTGGGTGTTCACACTCAATTTCACAGGAGAGACTCCTATC    100
                     ||||||||||||||||||||||||||||||||||||||||||||||||||
alphaDRC2012      40 ATCAGCGAAATTGGGTGTTCACACTCAATTTCACAGGAGAGACTCCTATC     89

alphaDRC2016     101 CTTTCCTTTGGAGAGGAAACACAATATGCGTGTTGGCAACACGAGAAGGT    150
                     ||||||||||||||||||||||||||||||||||||||||||||||||||
alphaDRC2012      90 CTTTCCTTTGGAGAGGAAACACAATATGCGTGTTGGCAACACGAGAAGGT    139

alphaDRC2016     151 GGATCACGACCACCTACAGGGAGTGATCCAATTGAAGAAGAAGACCCGAT    200
                     ||||||||||||||||||||||||||||||||||||||||||||||||||
alphaDRC2012     140 GGATCACGACCACCTACAGGGAGTGATCCAATTGAAGAAGAAGACCCGAT    189

alphaDRC2016     201 TAAACGGAGCGAAGCGACTGATCGGGGGAAATCCACACCTCGAACCCATG    250
                     ||||||||||||||||||||||||||||||||||||||||||||||||||
alphaDRC2012     190 TAAACGGAGCGAAGCGACTGATCGGGGGAAATCCACACCTCGAACCCATG    239

alphaDRC2016     251 CGAGGTTCGATTACAGAGGCGAAAGCCTACTGTACGAAAGAACAATCACG    300
                     ||||||||.|||||||||||||||||||||||||||||||||||||||||
alphaDRC2012     240 CGAGGTTCAATTACAGAGGCGAAAGCCTACTGTACGAAAGAACAATCACG    289

alphaDRC2016     301 GATCGCAGGTCCGTGGGAATTCGGAGAAATTCTGCTGAAGGGGTCGAACA    350
                     ||||||||||||||||||||||||||||||.|||||||||||||||||||
alphaDRC2012     290 GATCGCAGGTCCGTGGGAATTCGGAGAAATGCTGCTGAAGGGGTCGAACA    339

alphaDRC2016     351 GACGGAAGCTCGCAGAGCTTCTGGATGATCCCGATAACGAAATAAATGAA    400
                     ||||||||||||||||||||||||||||||||||||||||||||||||||
alphaDRC2012     340 GACGGAAGCTCGCAGAGCTTCTGGATGATCCCGATAACGAAATAAATGAA    389

alphaDRC2016     401 CCTCAAAAATATAGACGAGCGATGGCTAAGTCCGCCATGGATGAATCTCG    450
                     ||||||||||||||||||||||||||||||||||||||||||||||||||
alphaDRC2012     390 CCTCAAAAATATAGACGAGCGATGGCTAAGTCCGCCATGGATGAATCTCG    439

alphaDRC2016     451 GAAGCTTGCTGAAGAGTATGATTTCCCTCACGAACTACGTTCGTGGCAAA    500
                     |||||||||||||||||||||||||||||||||||||||.||||||||||
alphaDRC2012     440 GAAGCTTGCTGAAGAGTATGATTTCCCTCACGAACTACGCTCGTGGCAAA    489
```

```
alphaDRC2016     501 AAACCCTAATTTCATGCCTCGAAGAGGAACCGGATGATCGTACTATTTAC    550
                     ||||||||||.||||.||||||||||||||||||||||||||||||||||
alphaDRC2012     490 AAACCCTAATCTCATTCCTCGAAGAGGAACCGGATGATCGTACTATTTAC    539

alphaDRC2016     551 TGGGTCTATGGTCCTAATGGAGGAGAAGGTAAAACCCAGTTCGGTAAACA    600
                     ||||||||||||||||||||||||||||||||||||||||||||||||||
alphaDRC2012     540 TGGGTCTATGGTCCTAATGGAGGAGAAGGTAAAACCCAGTTCGGTAAACA    589

alphaDRC2016     601 CCTAGGATTAAAAAAAGGATGGACCTATTTACCCGGAGGGGAACTGAAAG    650
                     |||.||||||||||||||||||||||||||||||||||||||||||||||
alphaDRC2012     590 CCTGGGATTAAAAAAAGGATGGACCTATTTACCCGGAGGGGAACTGAAAG    639

alphaDRC2016     651 ACATGATGTATCTATACAGTAAGGAGTTAAAAAACCATGTGGTTATTGAT    700
                     ||||||||||||||||||||||||||||||||||||||||||||||||||
alphaDRC2012     640 ACATGATGTATCTATACAGTAAGGAGTTAAAAAACCATGTGGTTATTGAT    689

alphaDRC2016     701 TTCCCCAGGTGTACGAAAGACTTTGTAAGTTATAAATTCCTAGAGATGGT    750
                     ||||||||.|||||||||||||||||||||||||||||||||||||||||
alphaDRC2012     690 TTCCCCAGATGTACGAAAGACTTTGTAAGTTATAAATTCCTAGAGATGGT    739

alphaDRC2016     751 AAAAAACAGAACTGTATATAGTTATAAATACGAACCGATAGGTTCTATAG    800
                     ||||||.|||||||||||||||||||||||||||||||||||||||||||
alphaDRC2012     740 AAAAAATAGAACTGTATATAGTTATAAATACGAACCGATAGGTTCTATAG    789

alphaDRC2016     801 TCAGTAACAAAGTTCATGTAGTGGTCTTATGTAATTTTATGCCGGAAGAA    850
                     |||||||||||||||||||||||||.|||||||||||..|||||||||||
alphaDRC2012     790 TCAGTAACAAAGTTCATGTAGTGGTATTATGTAATTTCCTGCCGGAAGAA    839

alphaDRC2016     851 GAAAAAATCTCCGGAGACAGATTAATAATAATAAATTGCTAAACACGCAT    900   Rep ORF stop codon
                     ||||||||||||||||||||||||||||||||||||||||||||||||||
alphaDRC2012     840 GAAAAAATCTCCGGAGACAGATTAATAATAATAAATTGCTAAACACGCAT    889   Rep ORF stop codon

alphaDRC2016     901 TTACAAA-AATACCCTCGAACAATCAATATTCCTATTATACCCCCGAAGC    949
                     ||||||| ||.||||||||||||||||||||.||||||||||||||||||
alphaDRC2012     890 TTACAAATAACACCCTCGAACAATCAATATTTCTATTATACCCCCGAAGC    939

alphaDRC2016     950 TCACTTCGGAACA------ACAGCTGTCGTTATCAATGACAGCTGTCATG    993
                     |||||||||||||      |||||||||...||||..|..||||||||||
alphaDRC2012     940 TCACTTCGGAACAACAGCGACAGCTGTCAACATCAGCGTTAGCTGTCATG    989

alphaDRC2016     994 TGGTCCCAACATAGCGTATGTAATACGCGGACTTGAAAGGATTATTAAAT   1043
                     |||.||||||||||||||||||||||||||||||||||||||||||||||
alphaDRC2012     990 TGGGCCCAACATAGCGTATGTAATACGCGGACTTGAAAGGATTATTAAAT   1039

alphaDRC2016    1044 CCGCTTTGCTGCTACAGTGAAGCTTGGCACACTATAAATACCTGTGCCAA   1093    TATA-box
                     ||||||||||||||||||||||||||||||||||||||||||||||||||
alphaDRC2012    1040 CCGCTTTGCTGCTACAGTGAAGCTTGGCACACTATAAATACCTGTGCCAA   1089    TATA-box

alphaDRC2016    1094 GGAGAGGGCTAG   1105
                     ||||||||||||
alphaDRC2012    1090 GGAGAGGGCTAG   1101


# Aligned_protein_sequences: 2
# 1: Rep_alphaDRC2016
# 2: Rep_alphaDRC2012
# Matrix: EBLOSUM62
# Gap_penalty: 10.0
# Extend_penalty: 0.5
#
# Length: 283
# Identity:     280/283 (98.9%)
# Similarity:   282/283 (99.6%)
# Gaps:           0/283 ( 0.0%)

Rep_alphaDRC2       1 MTCHQRNWVFTLNFTGETPILSFGEETQYACWQHEKVDHDHLQGVIQLKK     50
                      ||||||||||||||||||||||||||||||||||||||||||||||||||
Rep_alphaDRC2       1 MTCHQRNWVFTLNFTGETPILSFGEETQYACWQHEKVDHDHLQGVIQLKK     50

Rep_alphaDRC2      51 KTRLNGAKRLIGGNPHLEPMRGSITEAKAYCTKEQSRIAGPWEFGEILLK    100
                      ||||||||||||||||||||||||||||||||||||||||||||||:|||
Rep_alphaDRC2      51 KTRLNGAKRLIGGNPHLEPMRGSITEAKAYCTKEQSRIAGPWEFGEMLLK    100

Rep_alphaDRC2     101 GSNRRKLAELLDDPDNEINEPQKYRRAMAKSAMDESRKLAEEYDFPHELR    150
                      ||||||||||||||||||||||||||||||||||||||||||||||||||
Rep_alphaDRC2     101 GSNRRKLAELLDDPDNEINEPQKYRRAMAKSAMDESRKLAEEYDFPHELR    150

Rep_alphaDRC2     151 SWQKTLISCLEEEPDDRTIYWVYGPNGGEGKTQFGKHLGLKKGWTYLPGG    200
                      ||||||||.|||||||||||||||||||||||||||||||||||||||||
Rep_alphaDRC2     151 SWQKTLISFLEEEPDDRTIYWVYGPNGGEGKTQFGKHLGLKKGWTYLPGG    200

Rep_alphaDRC2     201 ELKDMMYLYSKELKNHVVIDFPRCTKDFVSYKFLEMVKNRTVYSYKYEPI    250
                      ||||||||||||||||||||||||||||||||||||||||||||||||||
Rep_alphaDRC2     201 ELKDMMYLYSKELKNHVVIDFPRCTKDFVSYKFLEMVKNRTVYSYKYEPI    250

Rep_alphaDRC2     251 GSIVSNKVHVVVLCNFMPEEEKISGDRLIIINC    283
                      ||||||||||||||||:||||||||||||||||
Rep_alphaDRC2     251 GSIVSNKVHVVVLCNFLPEEEKISGDRLIIINC    283
```

Supplementary Dataset S1D. Protein and Nucleotide sequence Blast analyses of DRC alphasatellite isolates.

Protein BLAST Rep_alphaDRC2016

Replication-associated protein of non-essential DNA C9 [**Faba bean necrotic yellows virus (isolate SV292-88)**]
Sequence ID: P0CK61.1 Length: 281 Number of Matches: 1

**Identities**	**Positives**	**Gaps**
180/277(**65%**)	227/277(**81%**)	0/277(**0%**)

```
Query  7    NWVFTLNFTGETPILSFGEETQYACWQHEKVDHDHLQGVIQLKKKTRLNGAKRLIGGNPH  66
            NWVFTLNF GE P+LSF E  QYA WQHE+V+HDH+QGVIQLKKK ++N  K +IGGNPH
Sbjct  5    NWVFTLNFAGEVPVLSFDERVQYAVWQHERVNHDHIQGVIQLKKKAKMNTVKNIIGGNPH  64

Query  67   LEPMRGSITEAKAYCTKEQSRIAGPWEFGEILLKGSNRRKLAELLDDPDNEINEPQKYRR  126
            LE M+GSI EA AY  KE+SR+AGPW +GE+L KGS++RK+ EL+ DP+NE+ EPQKYRR
Sbjct  65   LEKMKGSIEEASAYAQKEESRVAGPWSYGELLKKGSHKRKIMELIKDPENELEEPQKYRR  124

Query  127  AMAKSAMDESRKLAEEYDFPHELRSWQKTLISCLEEEPDDRTIYWVYGPNGGEGKTQFGK  186
            AMA SAMDESRKLAEE  FP+   SWQ+T++  LEEEP+DRTI WVYGPNG EGK+QFGK
Sbjct  125  AMAWSAMDESRKLAEEEGFPYMFYSWQETVLGLLEEEPNDRTIIWVYGPNGNEGKSQFGK  184

Query  187  HLGLKKGWTYLPGGELKDMMYLYSKELKNHVVIDFPRCTKDFVSYKFLEMVKNRTVYSYK  246
             LGLKK + YLPGG+ +DM Y+  K  K +VV+D PRC  ++++Y+F+E++KNRT+YSYK
Sbjct  185  FLGLKKDYLYLPGGKTQDMTYMLMKNPKANVVMDIPRCNSEYLNYQFMELIKNRTIYSYK  244

Query  247  YEPIGSIVSNKVHVVVLCNFMPEEEKISGDRLIIINC  283
            YEP+G I++NK+HV+VL N +P+ EKIS DR+ II C
Sbjct  245  YEPVGCIINNKIHVIVLANVLPDYEKISQDRIKIIYC  281
```

Replication-associated protein [**Faba bean necrotic yellows virus associated alphasatellite 2**]
Sequence ID: ATU31571.1 Length: 281 Number of Matches: 1

Identities	Positives	Gaps
179/277(**65%**)	228/277(**82%**)	0/277(0%)

```
Query  7    NWVFTLNFTGETPILSFGEETQYACWQHEKVDHDHLQGVIQLKKKTRLNGAKRLIGGNPH  66
            NWVFTLNF GE P+LSF E  QYA WQHE+V+HDH+QGVIQLKKK ++N  K +IGGNPH
Sbjct  5    NWVFTLNFAGEVPVLSFDERVQYAVWQHERVNHDHIQGVIQLKKKAKMNTVKNIIGGNPH  64

Query  67   LEPMRGSITEAKAYCTKEQSRIAGPWEFGEILLKGSNRRKLAELLDDPDNEINEPQKYRR  126
            LE M+GSI EA AY  KE+SR+AGPW +GE+L KGS++RK+ EL+ DP+NE+ EPQKYRR
Sbjct  65   LEKMKGSIEEASAYAQKEESRVAGPWSYGELLKKGSHKRKIMELIKDPENELEEPQKYRR  124

Query  127  AMAKSAMDESRKLAEEYDFPHELRSWQKTLISCLEEEPDDRTIYWVYGPNGGEGKTQFGK  186
            AMA SAMDESRKLAEE  FP+ L SWQ+T++  L+EEP+DRTI WVYGPNG EGK+QFGK
Sbjct  125  AMAWSAMDESRKLAEEEGFPYSLYSWQETVLGLLDEEPNDRTIIWVYGPNGNEGKSQFGK  184

Query  187  HLGLKKGWTYLPGGELKDMMYLYSKELKNHVVIDFPRCTKDFVSYKFLEMVKNRTVYSYK  246
             LGLKK + YLPGG+ +DM Y+  K  K +VV+D PRC  ++++Y+F+E++KNRT++SYK
Sbjct  185  FLGLKKDYLYLPGGKTQDMTYMLMKNPKANVVMDIPRCNSEYLNYQFMELIKNRTIFSYK  244

Query  247  YEPIGSIVSNKVHVVVLCNFMPEEEKISGDRLIIINC  283
            YEP+G I++NK+HV+VL N +P+ EKIS DR+ II C
Sbjct  245  YEPVGCIINNKIHVIVLANVLPDYEKISQDRIKIIYC  281
```

Rep protein [**Faba bean necrotic yellows C9 alphasatellite**]
Sequence ID: NP_619574.1 Length: 281 Number of Matches: 1

Identities	Positives	Gaps
179/277(65%)	227/277(81%)	0/277(0%)

```
Query  7    NWVFTLNFTGETPILSFGEETQYACWQHEKVDHDHLQGVIQLKKKTRLNGAKRLIGGNPH  66
            NWVFTLNF GE P+LSF E  QYA WQHE+V+HDH+QGVIQLKKK ++N  K +IGGNPH
Sbjct  5    NWVFTLNFAGEVPVLSFDERVQYAVWQHERVNHDHIQGVIQLKKKAKMNTVKNIIGGNPH  64

Query  67   LEPMRGSITEAKAYCTKEQSRIAGPWEFGEILLKGSNRRKLAELLDDPDNEINEPQKYRR  126
            LE M+GSI EA AY  KE+SR+AGPW +GE+L KGS++RK+ EL+ DP+NE+ EPQKYRR
Sbjct  65   LEKMKGSIEEASAYAQKEESRVAGPWSYGELLKKGSHKRKIMELIKDPENELEEPQKYRR  124

Query  127  AMAKSAMDESRKLAEEYDFPHELRSWQKTLISCLEEEPDDRTIYWVYGPNGGEGKTQFGK  186
            AMA SAMDESRKLAEE  FP+ L SWQ+T++  LEEEP+DR I WVYGPNG EGK+QFGK
Sbjct  125  AMAWSAMDESRKLAEEGGFPYTLYSWQETVLGLLEEEPNDRIIIWVYGPNGNEGKSQFGK  184

Query  187  HLGLKKGWTYLPGGELKDMMYLYSKELKNHVVIDFPRCTKDFVSYKFLEMVKNRTVYSYK  246
             LGLKK + YLPGG+ +DM Y+  K  K +VV+D PRC  ++++Y+F+E++KNRT++SYK
Sbjct  185  FLGLKKDYLYLPGGKTQDMTYMLMKNPKANVVMDIPRCNSEYLNYQFMELIKNRTIFSYK  244

Query  247  YEPIGSIVSNKVHVVVLCNFMPEEEKISGDRLIIINC  283
            YEP+G I++NK+HV+VL N +P+ EKIS DR+ II C
Sbjct  245  YEPVGCIINNKIHVIVLANVLPDYEKISQDRIKIIYC  281
```

Satellite replication initiator protein [**Sophora alopecuroides yellow stunt alphasatellite 3**]
Sequence ID: ARI50297.1 Length: 281 Number of Matches: 1

Identities	Positives	Gaps
181/277(65%)	222/277(80%)	0/277(0%)

```
Query  7    NWVFTLNFTGETPILSFGEETQYACWQHEKVDHDHLQGVIQLKKKTRLNGAKRLIGGNPH  66
            NWVFT NF GE PILSF E  QYA WQHE+V HDHLQGVIQLKKK RLN  K +IGGNPH
Sbjct  5    NWVFTRNFAGEVPILSFDERVQYAVWQHERVTHDHLQGVIQLKKKARLNTVKAMIGGNPH  64
```

```
Query  67   LEPMRGSITEAKAYCTKEQSRIAGPWEFGEILLKGSNRRKLAELLDDPDNEINEPQKYRR  126
            LE MR +I EA  YC K  SR++GPWEFG +L KGS++RK+ ELL+DPDNEI EPQKYRR
Sbjct  65   LEKMRATIEEAAEYCRKPDSRVSGPWEFGTMLKKGSHKRKILELLEDPDNEIEEPQKYRR  124

Query  127  AMAKSAMDESRKLAEEYDFPHELRSWQKTLISCLEEEPDDRTIYWVYGPNGGEGKTQFGK  186
            AMA SAM+ SR++A    FPH L SWQ+T++  LEEEP+DRTI WVYGPNG EGK+QFGK
Sbjct  125  AMAFSAMEASREIASREGFPHSLYSWQETVLGLLEEEPNDRTIIWVYGPNGNEGKSQFGK  184

Query  187  HLGLKKGWTYLPGGELKDMMYLYSKELKNHVVIDFPRCTKDFVSYKFLEMVKNRTVYSYK  246
            HLGLKK + YLPGG+ +DM Y+  K  ++HVV+D PRC  ++++Y+F+E++KNRT+YSYK
Sbjct  185  HLGLKKDYLYLPGGKTQDMTYMLMKCPESHVVLDIPRCNSEYLNYQFMELIKNRTIYSYK  244

Query  247  YEPIGSIVSNKVHVVVLCNFMPEEEKISGDRLIIINC  283
            YEP+G+I+ NK+HVVVL N +P  EKIS DR+ +I C
Sbjct  245  YEPLGAIIKNKIHVVVLANILPNYEKISQDRIKLIYC  281
```

…

…

Protein Blast Rep alphaDRC2012

Replication-associated protein of non-essential DNA C9 [**Faba bean necrotic yellows virus (isolate SV292-88)**]
Sequence ID: P0CK61.1   Length: 281 Number of Matches: 1

Identities	Positives	Gaps
181/277(**65%**)	227/277(**81%**)	0/277(0%)

```
Query  7    NWVFTLNFTGETPILSFGEETQYACWQHEKVDHDHLQGVIQLKKKTRLNGAKRLIGGNPH  66
            NWVFTLNF GE P+LSF E  QYA WQHE+V+HDH+QGVIQLKKK ++N  K +IGGNPH
Sbjct  5    NWVFTLNFAGEVPVLSFDERVQYAVWQHERVNHDHIQGVIQLKKKAKMNTVKNIIGGNPH  64

Query  67   LEPMRGSITEAKAYCTKEQSRIAGPWEFGEMLLKGSNRRKLAELLDDPDNEINEPQKYRR  126
            LE M+GSI EA AY  KE+SR+AGPW +GE+L KGS++RK+ EL+ DP+NE+ EPQKYRR
Sbjct  65   LEKMKGSIEEASAYAQKEESRVAGPWSYGELLKKGSHKRKIMELIKDPENELEEPQKYRR  124

Query  127  AMAKSAMDESRKLAEEYDFPHELRSWQKTLISFLEEEPDDRTIYWVYGPNGGEGKTQFGK  186
            AMA SAMDESRKLAEE  FP+   SWQ+T++  LEEEP+DRTI WVYGPNG EGK+QFGK
Sbjct  125  AMAWSAMDESRKLAEEEGFPYMFYSWQETVLGLLEEEPNDRTIIWVYGPNGNEGKSQFGK  184

Query  187  HLGLKKGWTYLPGGELKDMMYLYSKELKNHVVIDFPRCTKDFVSYKFLEMVKNRTVYSYK  246
             LGLKK + YLPGG+ +DM Y+  K  K +VV+D PRC  ++++Y+F+E++KNRT+YSYK
Sbjct  185  FLGLKKDYLYLPGGKTQDMTYMLMKNPKANVVMDIPRCNSEYLNYQFMELIKNRTIYSYK  244

Query  247  YEPIGSIVSNKVHVVVLCNFLPEEEKISGDRLIIINC  283
            YEP+G I++NK+HV+VL N LP+ EKIS DR+ II C
Sbjct  245  YEPVGCIINNKIHVIVLANVLPDYEKISQDRIKIIYC  281
```

Satellite replication initiator protein [Sophora alopecuroides yellow stunt alphasatellite 3]
Sequence ID: ARI50297.1   Length: 281 Number of Matches: 1

Identities	Positives	Gaps
183/277(66%)	222/277(80%)	0/277(0%)

```
Query  7    NWVFTLNFTGETPILSFGEETQYACWQHEKVDHDHLQGVIQLKKKTRLNGAKRLIGGNPH  66
            NWVFT NF GE PILSF E  QYA WQHE+V HDHLQGVIQLKKK RLN  K +IGGNPH
Sbjct  5    NWVFTRNFAGEVPILSFDERVQYAVWQHERVTHDHLQGVIQLKKKARLNTVKAMIGGNPH  64

Query  67   LEPMRGSITEAKAYCTKEQSRIAGPWEFGEMLLKGSNRRKLAELLDDPDNEINEPQKYRR  126
            LE MR +I EA  YC K  SR++GPWEFG ML KGS++RK+ ELL+DPDNEI EPQKYRR
Sbjct  65   LEKMRATIEEAAEYCRKPDSRVSGPWEFGTMLKKGSHKRKILELLEDPDNEIEEPQKYRR  124

Query  127  AMAKSAMDESRKLAEEYDFPHELRSWQKTLISFLEEEPDDRTIYWVYGPNGGEGKTQFGK  186
            AMA SAM+ SR++A    FPH L SWQ+T++  LEEEP+DRTI WVYGPNG EGK+QFGK
Sbjct  125  AMAFSAMEASREIASREGFPHSLYSWQETVLGLLEEEPNDRTIIWVYGPNGNEGKSQFGK  184

Query  187  HLGLKKGWTYLPGGELKDMMYLYSKELKNHVVIDFPRCTKDFVSYKFLEMVKNRTVYSYK  246
            HLGLKK + YLPGG+ +DM Y+  K  ++HVV+D PRC  ++++Y+F+E++KNRT+YSYK
Sbjct  185  HLGLKKDYLYLPGGKTQDMTYMLMKCPESHVVLDIPRCNSEYLNYQFMELIKNRTIYSYK  244

Query  247  YEPIGSIVSNKVHVVVLCNFLPEEEKISGDRLIIINC  283
            YEP+G+I+ NK+HVVVL N LP  EKIS DR+ +I C
Sbjct  245  YEPLGAIIKNKIHVVVLANILPNYEKISQDRIKLIYC  281
```

Replication-associated protein [**Faba bean necrotic yellows virus associated alphasatellite 2]**
Sequence ID: ATU31571.1   Length: 281 Number of Matches: 1

Identities	Positives	Gaps
180/277(65%)	228/277(82%)	0/277(0%)

```
Query  7    NWVFTLNFTGETPILSFGEETQYACWQHEKVDHDHLQGVIQLKKKTRLNGAKRLIGGNPH  66
            NWVFTLNF GE P+LSF E  QYA WQHE+V+HDH+QGVIQLKKK ++N  K +IGGNPH
Sbjct  5    NWVFTLNFAGEVPVLSFDERVQYAVWQHERVNHDHIQGVIQLKKKAKMNTVKNIIGGNPH  64

Query  67   LEPMRGSITEAKAYCTKEQSRIAGPWEFGEMLLKGSNRRKLAELLDDPDNEINEPQKYRR  126
            LE M+GSI EA AY  KE+SR+AGPW +GE+L KGS++RK+ EL+ DP+NE+ EPQKYRR
Sbjct  65   LEKMKGSIEEASAYAQKEESRVAGPWSYGELLKKGSHKRKIMELIKDPENELEEPQKYRR  124

Query  127  AMAKSAMDESRKLAEEYDFPHELRSWQKTLISFLEEEPDDRTIYWVYGPNGGEGKTQFGK  186
            AMA SAMDESRKLAEE  FP+ L SWQ+T++  L+EEP+DRTI WVYGPNG EGK+QFGK
```

```
Sbjct  125  AMAWSAMDESRKLAEEEGFPYSLYSWQETVLGLLDEEPNDRTIIWVYGPNGNEGKSQFGK  184

Query  187  HLGLKKGWTYLPGGELKDMMYLYSKELKNHVVIDFPRCTKDFVSYKFLEMVKNRTVYSYK  246
             LGLKK + YLPGG+ +DM Y+  K  K +VV+D PRC  ++++Y+F+E++KNRT++SYK
Sbjct  185  FLGLKKDYLYLPGGKTQDMTYMLMKNPKANVVMDIPRCNSEYLNYQFMELIKNRTIFSYK  244

Query  247  YEPIGSIVSNKVHVVVLCNFLPEEEKISGDRLIIINC  283
            YEP+G I++NK+HV+VL N LP+ EKIS DR+ II C
Sbjct  245  YEPVGCIINNKIHVIVLANVLPDYEKISQDRIKIIYC  281
```

Rep protein [Faba bean necrotic yellows C9 alphasatellite]

```
Sequence ID: NP_619574.1  Length: 281  Number of Matches: 1

Identities               Positives                Gaps
180/277(65%)             227/277(81%)             0/277(0%)

Query  7    NWVFTLNFTGETPILSFGEETQYACWQHEKVDHDHLQGVIQLKKKTRLNGAKRLIGGNPH  66
            NWVFTLNF GE P+LSF E  QYA WQHE+V+HDH+QGVIQLKKK ++N  K +IGGNPH
Sbjct  5    NWVFTLNFAGEVPVLSFDERVQYAVWQHERVNHDHIQGVIQLKKKAKMNTVKNIIGGNPH  64

Query  67   LEPMRGSITEAKAYCTKEQSRIAGPWEFGEMLLKGSNRRKLAELLDDPDNEINEPQKYRR  126
            LE M+GSI EA AY  KE+SR+AGPW +GE+L KGS++RK+ EL+ DP+NE+ EPQKYRR
Sbjct  65   LEKMKGSIEEASAYAQKEESRVAGPWSYGELLKKGSHKRKIMELIKDPENELEEPQKYRR  124

Query  127  AMAKSAMDESRKLAEEYDFPHELRSWQKTLISFLEEEPDDRTIYWVYGPNGGEGKTQFGK  186
            AMA SAMDESRKLAEE  FP+ L SWQ+T++  LEEEP+DR I WVYGPNG EGK+QFGK
Sbjct  125  AMAWSAMDESRKLAEEGGFPYTLYSWQETVLGLLEEEPNDRIIIWVYGPNGNEGKSQFGK  184

Query  187  HLGLKKGWTYLPGGELKDMMYLYSKELKNHVVIDFPRCTKDFVSYKFLEMVKNRTVYSYK  246
             LGLKK + YLPGG+ +DM Y+  K  K +VV+D PRC  ++++Y+F+E++KNRT++SYK
Sbjct  185  FLGLKKDYLYLPGGKTQDMTYMLMKNPKANVVMDIPRCNSEYLNYQFMELIKNRTIFSYK  244

Query  247  YEPIGSIVSNKVHVVVLCNFLPEEEKISGDRLIIINC  283
            YEP+G I++NK+HV+VL N LP+ EKIS DR+ II C
Sbjct  245  YEPVGCIINNKIHVIVLANVLPDYEKISQDRIKIIYC  281
```

…

…

BlastN analysis of aphaDRC2016 alpfasatellite nucleotide sequence

Faba bean necrotic yellows virus associated alphasatellite 2 isolate TN-Tuf9_2-2015, complete sequence

```
Sequence ID: MF510475.1   Length: 1005 Number of Matches: 1
Range: 136 to 898
#1 Score                 Expect       Identities               Gaps         Strand
267 bits(295)            4e-66        532/774(69%)             22/774(2%)   Plus/Plus

Query  134  TGGCAACACGAGAAGGTGGATCACGACCACCTACAGGGAGTGATCCAATTGAAGAAGAAG  193
            ||||||||||| |  ||  ||||||||||  | ||||||||||| |||||||||||||||
Sbjct  136  TGGCAACACGAAAGAGTAAATCACGACCATATTCAGGGAGTGATTCAATTGAAGAAGAAG  195

Query  194  ACCCGATTAAACGGAGCGAAGCGACTGATCGGGGGAAATCCACACCTCGAACCCATGCGA  253
             |     | |||  || ||||    | ||||| |||||||| || || ||    |||
Sbjct  196  GCAAAGATGAACACAGTGAAGAACATAATCGGAGGAAATCCTCATCTGGAGAAGATGAAG  255

Query  254  GGTTCGATTACAGAGGCGAAAGCCTACTGTAC-GAAAGAACAATCACGGATCGCAGGTCC  312
            ||||||||   ||| ||    || || ||  | ||||||| ||||| |  |||| || ||
Sbjct  256  GGTTCGATAGAAGAAGCTTCTGCGTA-TGCCCAGAAAGAAGAATCAAGAGTCGCCGGACC  314

Query  313  GTGGGAATTCGGAGAAATTCTGCTGAAGGG--GTCGAACAGACGGAAGCTCGCAGAGCTT  370
             |||   | ||| ||| | |||  ||| ||  |||  ||| ||||||| |    ||| |
Sbjct  315  CTGGAGTTACGGTGAATTACTGAAGAAAGGTAGTC--ACAAACGGAAGATTATGGAGTTA  372

Query  371  CTGGATGATCCCGATAACGAAATAAATGAACCTCAAAAATATAGACGAGCGATGGCTAAG  430
             |  | ||||| || |||||| |  | ||||| || ||||| ||| |||||||||||  |
Sbjct  373  ATTAAAGATCCGGAGAACGAATTGGAAGAACCCCAGAAATACAGAAGAGCGATGGCTTGG  432

Query  431  TCCGCCATGGATGAATCTCGGAAGCTTGCTGAAG-AGTATGATTTCCCTCACGAACTACG  489
            ||||||||||| |||||||||||||||||||||| || | | ||||||| |   |||
Sbjct  433  TCCGCCATGGACGAATCTCGGAAGCTTGCTGAAGAAGAAGGGTTTCCCT-ATTCACTTTA  491

Query  490  TTCGTGGCAAAAAACCCTAATTTCATGCC---TCGAAGAGGAACCGGATGATCGTACTAT  546
                |||||| ||||   | | |   |||   | || || || ||  |||| ||||||||
Sbjct  492  CAGCTGGCAAGAAAC---AGTGTTGGGCCTATTAGATGAAGAGCCCAATGACCGTACTAT  548

Query  547  TTACTGGGTCTATGGTCCTAATGGAGGAGAAGGTAAAACCCAGTTCGGTAAACACCTAGG  606
            |   |||||||| || || |||||    ||||| ||| | ||||| ||||||  | | ||
Sbjct  549  TATTTGGGTCTACGGCCCAAATGGTAATGAAGGAAAATCACAGTTTGGTAAATTCTTGGG  608

Query  607  ATTaaaaaaaGGATGGACC--TATTTACCCGGAGGGGAACTGAAAGACATGATGTATCTA  664
            |||||||||| |||  |||  |||||||| |||||  ||    |||| ||||  ||| |
Sbjct  609  ATTAAAAAAA-GAT-TACCTTTATTTACCTGGAGGTAAAACCCAAGATATGACATATATG  666

Query  665  TACAGTAAGGAGTTAAAAAACCATGTGGTTATTGATTTCCCCAGGTGTACGAAAGACTTT  724
            |  |  ||  |   |||     |||| || || ||| | ||  ||||||    ||| | |
Sbjct  667  TTAATGAAAAATCCAAAGGCAAATGTTGTGATGGATATTCCTCGGTGTAATTCAGAATAT  726

Query  725  GTAAGTTATAAATTCCTAGAGATGGTAAAAAACAGAACTGTATATAGTTATAAATACGAA  784
             ||| |||  ||||  | ||  |  | ||||| |||||  ||| |||||||||||| |||
Sbjct  727  TTAAATTACCAATTTATGGAATTAATTAAAAATAGAACAATATTTAGTTATAAATATGAA  786
```

```
Query  785   CCGATAGGTTCTATAGTCAGTAACAAAGTTCATGTAGTGGTCTTATGTAATTTTATGCCG  844
             ||   | |||| |||   | |  ||| ||| | |||||| | || |||   |||| |   ||||
Sbjct  787   CCAGTTGGTTGTATTATAAATAATAAAATACATGTAATTGTATTAGCTAATGTATTGCCT  846

Query  845   GAAGAAGAAAAAAT--CTCCGGAGACAGATTAATAATAATAAATTGCTAAACAC  896
             ||   | ||||||||    || |  |||||| ||| ||||||  |||| |||| ||
Sbjct  847   GATTATGAAAAAAATTAGTCAG--GACAGAATAAAAATAATTTATTGTTAAATAC  898
```

Faba bean necrotic yellows virus associated alphasatellite 2 isolate TN-Tuf10_2-2015, complete sequence
Sequence ID: MF510474.1   Length: 1005 Number of Matches: 1
Range 1: 136 to 898

#1 Score	Expect	Identities	Gaps	Strand
267 bits(295)	4e-66	532/774(69%)	22/774(2%)	Plus/Plus

```
Query  134   TGGCAACACGAGAAGGTGGATCACGACCACCTACAGGGAGTGATCCAATTGAAGAAGAAG  193
             ||||||||||| |  ||  ||||||||||   | ||||||||||| ||||||||||||||
Sbjct  136   TGGCAACACGAAAGAGTAAATCACGACCATATTCAGGGAGTGATTCAATTGAAGAAGAAG  195

Query  194   ACCCGATTAAACGGAGCGAAGCGACTGATCGGGGGAAATCCACACCTCGAACCCATGCGA  253
              |      | |||  || ||||     | ||||| |||||||| || || ||     |||
Sbjct  196   GCAAAGATGAACACAGTGAAGAACATAATCGGAGGAAATCCTCATCTGGAGAAGATGAAG  255

Query  254   GGTTCGATTACAGAGGCGAAAGCCTACTGTAC-GAAAGAACAATCACGGATCGCAGGTCC  312
             ||||||||    ||| ||      || || ||   | ||||||| ||||| |   |||| || ||
Sbjct  256   GGTTCGATAGAAGAAGCTTCTGCGTA-TGCCCAGAAAGAAGAATCAAGAGTCGCCGGACC  314

Query  313   GTGGGAATTCGGAGAAATTCTGCTGAAGGG--GTCGAACAGACGGAAGCTCGCAGAGCTT  370
              |||    | ||| |||  | |||    ||| ||   |||   ||| |||||||  |     ||| |
Sbjct  315   CTGGAGTTACGGTGAATTACTGAAGAAAGGTAGTC--ACAAACGGAAGATTATGGAGTTA  372

Query  371   CTGGATGATCCCGATAACGAAATAAATGAACCTCAAAAATATAGACGAGCGATGGCTAAG  430
              |   | ||||| || |||||| |   | ||||| || ||||| ||| |||||||||||   |
Sbjct  373   ATTAAAGATCCGGAGAACGAATTGGAAGAACCCCAGAAATACAGAAGAGCGATGGCTTGG  432

Query  431   TCCGCCATGGATGAATCTCGGAAGCTTGCTGAAG-AGTATGATTTCCCTCACGAACTACG  489
             ||||||||||| |||||||||||||||||||||| || | | ||||||| |    |||
Sbjct  433   TCCGCCATGGACGAATCTCGGAAGCTTGCTGAAGAAGAAGGGTTTCCCT-ATTCACTTTA  491

Query  490   TTCGTGGCAAAAAACCCTAATTTCATGCC---TCGAAGAGGAACCGGATGATCGTACTAT  546
                 |||||| ||||    | | |    |||    | || || || ||   |||| ||||||||
Sbjct  492   CAGCTGGCAAGAAAC---AGTGTTGGGCCTATTAGATGAAGAGCCCAATGACCGTACTAT  548

Query  547   TTACTGGGTCTATGGTCCTAATGGAGGAGAAGGTAAAACCCAGTTCGGTAAACACCTAGG  606
             |    |||||||| || || |||||     ||||| ||| | ||||| ||||||   | | ||
Sbjct  549   TATTTGGGTCTACGGCCCAAATGGTAATGAAGGAAAATCACAGTTTGGTAAATTCTTGGG  608

Query  607   ATTaaaaaaaGGATGGACC--TATTTACCCGGAGGGGAACTGAAAGACATGATGTATCTA  664
             |||||||||| |||  |||  |||||||| |||||   ||     |||| ||||   ||| |
Sbjct  609   ATTAAAAAAA-GAT-TACCTTTATTTACCTGGAGGTAAAACCCAAGATATGACATATATG  666

Query  665   TACAGTAAGGAGTTAAAAAACCATGTGGTTATTGATTTCCCCAGGTGTACGAAAGACTTT  724
             |   |   ||   |    |||       |||| || || ||| | ||  ||||||    ||| |  |
Sbjct  667   TTAATGAAAAATCCAAAGGCAAATGTTGTGATGGATATTCCTCGGTGTAATTCAGAATAT  726

Query  725   GTAAGTTATAAATTCCTAGAGATGGTAAAAAACAGAACTGTATATAGTTATAAATACGAA  784
              ||| |||   ||||   | ||   |   | ||||| |||||   ||| ||||||||||| |||
Sbjct  727   TTAAATTACCAATTTATGGAATTAATTAAAAATAGAACAATATTTAGTATAAATATGAA  786

Query  785   CCGATAGGTTCTATAGTCAGTAACAAAGTTCATGTAGTGGTCTTATGTAATTTTATGCCG  844
             ||   | |||| |||   | |  ||| ||| | |||||| | || |||   |||| |   ||||
Sbjct  787   CCAGTTGGTTGTATTATAAATAATAAAATACATGTAATTGTATTAGCTAATGTATTGCCT  846

Query  845   GAAGAAGAAAAAAT--CTCCGGAGACAGATTAATAATAATAAATTGCTAAACAC  896
             ||   | ||||||||    || |  |||||| ||| ||||||  |||| |||| ||
Sbjct  847   GATTATGAAAAAAATTAGTCAG--GACAGAATAAAAATAATTTATTGTTAAATAC  898
```

Faba bean necrotic yellows C9 alphasatellite C9-Eg gene, isolate Egyptian EV1-93
Sequence ID: AJ132187.1   Length: 1007 Number of Matches: 1
Range 1: 85 to 918

#1 Score	Expect	Identities	Gaps	Strand
245 bits(271)	1e-59	571/846(67%)	24/846(2%)	Plus/Plus

```
Query  59    AATTGGGTGTTCACACTCAATTTCACAGGAGAGACTCCTATCCTTTCCTTTGGAGAGGAA  118
             || ||||| |||||   | || ||| | || ||    |||| | || || || |   |||   |
Sbjct  85    AACTGGGTTTTCACGTTGAACTTCGCCGGCGAAGTTCCTGTTCTCTCGTTCGACGAGAGA  144

Query  119   ACACAATATGCGTGTTGGCAACACGAGAAGGTGGATCACGACCACCTACAGGGAGTGATC  178
                 ||||| ||      ||||||||||| |  ||  ||||||||||   | |||||||||||
Sbjct  145   GTTCAATACGCCGTCTGGCAACACGAAAGAGTAAATCACGACCATATTCAGGGAGTGATT  204

Query  179   CAATTGAAGAAGAAGACCCGATTAAACGGAGCGAAGCGACTGATCGGGGGAAATCCACAC  238
             ||||| ||||||||| |      | |||  || ||||     | || || |||||||| ||
Sbjct  205   CAATTAAAGAAGAAGGCAAAGATGAACACAGTGAAGAACATCATTGGTGGAAATCCTCAT  264

Query  239   CTCGAACCCATGCGAGGTTCGATTACAGAGGCGAAAGCCTACTGTAC-GAAAGAACAATC  297
             || ||     |||    ||||||||    ||| ||      || || ||   | ||||||| ||||
Sbjct  265   CTGGAGAAGATGAAGGGTTCGATAGAAGAAGCTTCTGCGTA-TGCCCAGAAAGAAGAATC  323

Query  298   ACGGATCGCAGGTCCGTGGGAATTCGGAGAAATTCTGCTGAAGGG--GTCGAACAGACGG  355
             | |   |||| || || |||    | ||| |||  |   ||   ||| ||   |||   | | ||||
Sbjct  324   AAGAGTCGCCGGACCCTGGAGTTACGGTGAATTATTGAAGAAAGGTAGTC--ATAAACGG  381

Query  356   AAGCTCGCAGAGCTTCTGGATGATCCCGATAACGAAATAAATGAACCTCAAAAATATAGA  415
```

```
            ||| |    ||| |  |  | ||||| || |||||| |  | ||||| || ||||| |||
Sbjct  382  AAGATTATGGAGTTAATTAAAGATCCGGAGAACGAATTGGAAGAACCCCAGAAATACAGA  441

Query  416  CGAGCGATGGCTAAGTCCGCCATGGATGAATCTCGGAAGCTTGCTGAAG-AGTATGATTT  474
             |||||||||||  |||||||||||| |||||||||||||||||||||| || | | |||
Sbjct  442  AGAGCGATGGCTTGGTCCGCCATGGACGAATCTCGGAAGCTTGCTGAAGAAGGAGGCTTT  501

Query  475  CCCT-CACGAACTACGTTCGTGGCAAAAAACCCTAATTTCATGCC---TCGAAGAGGAAC  530
            ||||  |||   |||     |||||| ||||   | | |   |||   | ||||| || |
Sbjct  502  CCCTATACGCTTTAC--AGCTGGCAAGAAAC---AGTGTTGGGCCTATTAGAAGAAGAGC  556

Query  531  CGGATGATCGTACTATTTACTGGGTCTATGGTCCTAATGGAGGAGAAGGTAAAACCCAGT  590
            |  |||| |||| ||||   |||||||| || || |||||    ||||| ||| | ||||
Sbjct  557  CCAATGACCGTATTATTATTTGGGTCTACGGCCCAAATGGTAACGAAGGAAAATCACAGT  616

Query  591  TCGGTAAACACCTAGGATTaaaaaaaGGATGGACC--TATTTACCCGGAGGGGAACTGAA  648
            | ||||||  ||| |||||||||||| |||  |||  |||||||| |||||  ||    |
Sbjct  617  TTGGTAAATTCCTGGGATTAAAAAAA-GAT-TACCTTTATTTACCTGGAGGTAAAACCCA  674

Query  649  AGACATGATGTATCTATACAGTAAGGAGTTAAAAAACCATGTGGTTATTGATTTCCCCAG  708
            ||| ||||  ||| | |  |  ||  |   |||     |||| || || ||| | ||  |
Sbjct  675  AGATATGACATATATGTTAATGAAAAATCCAAAGGCAAATGTTGTGATGGATATTCCTCG  734

Query  709  GTGTACGAAAGACTTTGTAAGTTATAAATTCCTAGAGATGGTAAAAAACAGAACTGTATA  768
             ||||     || | | ||| |||| ||||  | ||  |  | ||||| |||||  |||
Sbjct  735  TTGTAATTCTGAATATTTAAATTATCAATTTATGGAATTAATTAAAAATAGAACCATATT  794

Query  769  TAGTTATAAATACGAACCGATAGGTTCTATAGTCAGTAACAAAGTTCATGTAGTGGTCTT  828
            |||||||||||| |||||  | || | |||  | | ||| ||| | |||||| | || ||
Sbjct  795  TAGTTATAAATATGAACCAGTTGGATGTATTATAAATAATAAAATACATGTAATTGTATT  854

Query  829  ATGTAATTTTATGCCGGAAGAAGAAAAAAT--CTCCGGAGACAGATTAATAATAATAAAT  886
            |  |||| |  |||| ||  | ||||||||   || |  |||||| | | ||||||  ||
Sbjct  855  AGCTAATGTATTGCCTGATTATGAAAAAATTAGTCAG--GACAGAATTAAAATAATTTAT  912

Query  887  TGCTAA  892
            || |||
Sbjct  913  TGTTAA  918
```

Faba bean necrotic yellows C9 alphasatellite C9 gene
Sequence ID: AJ005966.1 Length: 1004 Number of Matches: 1
Range 1: 85 to 918

#1 Score	Expect	Identities	Gaps	Strand
236 bits(261)	6e-57	570/846(67%)	24/846(2%)	Plus/Plus

```
Query  59   AATTGGGTGTTCACACTCAATTTCACAGGAGAGACTCCTATCCTTTCCTTTGGAGAGGAA  118
            || ||||| |||||  | || ||| | || ||   |||| | || || || |  |||  |
Sbjct  85   AACTGGGTTTTCACGTTGAACTTCGCCGGCGAAGTTCCTGTTCTCTCGTTCGACGAGAGA  144

Query  119  ACACAATATGCGTGTTGGCAACACGAGAAGGTGGATCACGACCACCTACAGGGAGTGATC  178
               ||||| ||    ||||||||||| |  ||  ||||||||||  | |||||||||||
Sbjct  145  GTTCAATACGCCGTCTGGCAACACGAAAGAGTAAATCACGACCATATTCAGGGAGTGATT  204

Query  179  CAATTGAAGAAGAAGACCCGATTAAACGGAGCGAAGCGACTGATCGGGGGAAATCCACAC  238
            ||||| ||||||||| |     | |||  || ||||    | ||||| |||||||| ||
Sbjct  205  CAATTAAAGAAGAAGGCAAAGATGAACACAGTGAAGAATATCATCGGTGGAAATCCTCAT  264

Query  239  CTCGAACCCATGCGAGGTTCGATTACAGAGGCGAAAGCCTACTGTAC-GAAAGAACAATC  297
            || ||    |||   ||||||||   ||| ||    || || ||  | ||||||| ||||
Sbjct  265  CTGGAGAAGATGAAGGGTTCGATAGAAGAAGCTTCTGCGTA-TGCCCAGAAAGAAGAATC  323

Query  298  ACGGATCGCAGGTCCGTGGGAATTCGGAGAAATTCTGCTGAAGGG--GTCGAACAGACGG  355
            | |  |||| || || |||   | ||| ||| |  ||  ||| ||  |||  | | |||
Sbjct  324  AAGAGTCGCCGGACCCTGGAGTTACGGTGAATTATTGAAGAAAGGTAGTC--ATAAACGA  381

Query  356  AAGCTCGCAGAGCTTCTGGATGATCCCGATAACGAAATAAATGAACCTCAAAAATATAGA  415
            ||| |    ||| |  |  | ||||| || |||||| |  | ||||| || ||||| |||
Sbjct  382  AAGATTATGGAGTTAATTAAAGATCCGGAGAACGAATTGGAAGAACCCCAGAAATACAGA  441

Query  416  CGAGCGATGGCTAAGTCCGCCATGGATGAATCTCGGAAGCTTGCTGAAG-AGTATGATTT  474
             |||| ||||||  |||||||||||| ||||||||||||||||| |||| || | | |||
Sbjct  442  AGAGCTATGGCTTGGTCCGCCATGGACGAATCTCGGAAGCTTGCGGAAGAAGAAGGCTTT  501

Query  475  CCCT-CACGAACTACGTTCGTGGCAAAAAACCCTAATTTCATGCC---TCGAAGAGGAAC  530
            ||||  | |   |||     |||||| ||||   | | |   |||   | ||||| || |
Sbjct  502  CCCTATATGTTTTAC--AGCTGGCAAGAAAC---AGTGTTGGGCCTATTAGAAGAAGAGC  556

Query  531  CGGATGATCGTACTATTTACTGGGTCTATGGTCCTAATGGAGGAGAAGGTAAAACCCAGT  590
            |  |||| |||||||||   |||||||| || || |||||    ||||| ||| | ||||
Sbjct  557  CCAATGACCGTACTATTATTTGGGTCTACGGCCCAAATGGTAATGAAGGAAAATCACAGT  616

Query  591  TCGGTAAACACCTAGGATTaaaaaaaGGATGGACC--TATTTACCCGGAGGGGAACTGAA  648
            | ||||||  ||| |||||||||||| |||  |||  |||||||| |||||  ||    |
Sbjct  617  TTGGTAAATTCCTGGGATTAAAAAAA-GAT-TACCTTTATTTACCTGGAGGTAAAACCCA  674

Query  649  AGACATGATGTATCTATACAGTAAGGAGTTAAAAAACCATGTGGTTATTGATTTCCCCAG  708
            ||| ||||  ||| | |  |  ||  |   |||     |||| || || ||| | ||  |
Sbjct  675  AGATATGACATATATGTTAATGAAAAATCCAAAGGCAAATGTTGTGATGGATATTCCTCG  734

Query  709  GTGTACGAAAGACTTTGTAAGTTATAAATTCCTAGAGATGGTAAAAAACAGAACTGTATA  768
             ||||     || | | ||| |||  ||||  | ||  |  | ||||| |||||  ||||
Sbjct  735  TTGTAATTCTGAATATTTAAATTACCAATTTATGGAATTAATTAAAAATAGAACCATATA  794

Query  769  TAGTTATAAATACGAACCGATAGGTTCTATAGTCAGTAACAAAGTTCATGTAGTGGTCTT  828
```

```
            |||||||||||| |||||  | || | |||  | | ||| ||| | |||||| | || ||
Sbjct  795  TAGTTATAAATATGAACCAGTTGGATGTATTATAAATAATAAAATACATGTAATTGTATT  854

Query  829  ATGTAATTTTATGCCGGAAGAAGAAAAAAT--CTCCGGAGACAGATTAATAATAATAAAT  886
            |  |||| |  |||| ||  | ||||||||   || ||| | | ||||| ||||||  ||
Sbjct  855  AGCTAATGTATTGCCTGATTATGAAAAAATTAGTCAGGATAGA-ATTAA-AATAATTTAT  912

Query  887  TGCTAA  892
            || |||
Sbjct  913  TGTTAA  918
```

Milk vetch chlorotic dwarf virus alphasatellite 1 isolate G53, complete sequence
Sequence ID: MN273340.1 Length: 999 Number of Matches: 1
Range 1: 62 to 885

```
#1 Score               Expect      Identities             Gaps        Strand
231 bits(255)          3e-55       550/827(67%)           6/827(0%)   Plus/Plus

Query  59   AATTGGGTGTTCACACTCAATTTCACAGGAGAGACTCCTATC-CTTTCCTTTGGAGAGGA  117
            |||||||| || || || ||||||||||| |   ||||  || || || || |  |||  
Sbjct  62   AATTGGGTTTTTACGCTGAATTTCACAGGCGCT-CTCCCTTCTCTCTCGTTCGACGAGAG  120

Query  118  AACACAATATGCGTGTTGGCAACACGAGAAGGTGGATCACGACCACCTACAGGGAGTGAT  177
            |   ||||||||   ||||||||| ||||  ||  | || ||||| || |||||||| ||
Sbjct  121  AGTTCAATATGCAGCTTGGCAACATGAGAGAGTTAACCATGACCATCTCCAGGGAGTAAT  180

Query  178  CCAATTGAAGAAGAAGACCCGATTAAACGGAGCGAAGCGACTGATCGGGGGAAATCCACA  237
             ||||||||||| ||  | ||  | |||   |  |||    |||| ||||||||||| ||
Sbjct  181  TCAATTGAAGAATAAAGCTCGCCTTAACACTGTTAAGGCTATGATTGGGGGAAATCCCCA  240

Query  238  CCTCGAACCCATGCGAGGTTCGATTACAGAGGCGAAAGCCTACTGTACGAAAGAACAATC  297
             || ||    ||| ||||    |||  ||| ||   ||  |||   | ||||||  | ||
Sbjct  241  TCTGGAGAAGATGAGAGGGAAAATTGAAGAAGCATCAGAATACGCCATGAAAGAGGAGTC  300

Query  298  ACGGATCGCAGGTCCGTGGGAATTCGGAGAAATTCTGCTGAAGGG--GTCGAACAGACGG  355
              |  | ||||| || |||   |  || || ||  ||  ||| ||  |||  ||| ||| 
Sbjct  301  GAGAGTTGCAGGACCTTGGATTTATGGCGAGATGTTGAAGAAAGGAAGTC--ACAAACGT  358

Query  356  AAGCTCGCAGAGCTTCTGGATGATCCCGATAACGAAATAAATGAACCTCAAAAATATAGA  415
            ||| |    ||| |  | || ||||| || |||||| |  | ||||| |||||||  |||
Sbjct  359  AAGATTATGGAGTTAATTGAAGATCCTGAGAACGAATTGGAAGAACCCCAAAAATTCAGA  418

Query  416  CGAGCGATGGCTAAGTCCGCCATGGATGAATCTCGGAAGCTTGCTGAAGAGTATGATTTC  475
             |||||||||||  |||||||||||| |||||||||||||||||||  |||| | |  || 
Sbjct  419  AGAGCGATGGCTTGGTCCGCCATGGAAGAATCTCGGAAGCTTGCTTCAGAGGAAGGGTTT  478

Query  476  CCTCACGAACTACGTTCGTGGCAAAAAACCCTAATTTCATGCCTCGAAGAGGAACCGGAT  535
            |||||   |||       ||||||| ||| |    | |||    | ||| | || || |||
Sbjct  479  CCTCATACACTTTACAGCTGGCAAGAAAACGCGTTGTCACTGTTGGAAAACGACCCTGAT  538

Query  536  GATCGTACTATTTACTGGGTCTATGGTCCTAATGGAGGAGAAGGTAAAACCCAGTTCGGT  595
            |||||||| ||   |||||| ||||| ||  | ||    ||||| ||| | ||||| |||
Sbjct  539  GATCGTACAATCATCTGGGTGTATGGGCCCCACGGTAATGAAGGAAAATCACAGTTTGGT  598

Query  596  AAACACCTAGGATTaaaaaaaGGATGGACCTATTTACCCGGAGGGGAACTGAAAGACATG  655
            |||  ||| || ||||||||||  |    ||||||||| || ||  ||    |||| |||
Sbjct  599  AAATTCCTGGGGTTAAAAAAAGATTACCTCTATTTACCAGGGGGTAAAACACAAGATATG  658

Query  656  ATGTATCTATACAGTAAGGAGTTAAAAAACCATGTGGTTATTGATTTCCCCAGGTGTACG  715
            | |||  |    |  ||| |   ||||    |||| || ||||||| | ||  | ||||  
Sbjct  659  ACGTACATGCTGATGAAGAACCCAAAAGCTAATGTTGTAATTGATATTCCACGCTGTAAT  718

Query  716  AAAGACTTTGTAAGTTATAAATTCCTAGAGATGGTAAAAAACAGAACTGTATATAGTTAT  775
              ||| | | | | ||| ||||   | ||  |  ||||||| |||||  | | |||||||
Sbjct  719  TCAGAATATCTGAATTATCAATTTATGGAATTAATAAAAAATAGAACAATTTTTAGTTAT  778

Query  776  AAATACGAACCGATAGGTTCTATAGTCAGTAACAAAGTTCATGTAGTGGTCTTATGTAAT  835
            ||||| || ||  | |||| ||| || | ||| ||| | |||||| | || ||    |||
Sbjct  779  AAATATGAGCCAGTTGGTTGTATTGTAAATAATAAAATACATGTAATTGTATTGGCCAAT  838

Query  836  TTTATGCCGGAAGAAGAAAAAATCTCCGGAGACAGATTAATAATAAT  882
             |  |||| ||  | ||||||||      ||||||| ||| ||| ||
Sbjct  839  GTATTGCCAGATTATGAAAAAATAAGTCAAGACAGAATAAAAATGAT  885
```

…

BlastN analysis of aphaDRC2012 alpfasatellite nucleotide sequence

Faba bean necrotic yellows virus associated alphasatellite 2 isolate TN-Tuf9_2-2015, complete sequence
Sequence ID: MF510475.1 Length: 1005 Number of Matches: 1
Range 1: 136 to 898

```
#1 Score               Expect      Identities             Gaps        Strand
274 bits(303)          3e-68       531/771(69%)           16/771(2%)  Plus/Plus

Query  123  TGGCAACACGAGAAGGTGGATCACGACCACCTACAGGGAGTGATCCAATTGAAGAAGAAG  182
            ||||||||||| |  ||  ||||||||||  | ||||||||||| |||||||||||||||
Sbjct  136  TGGCAACACGAAAGAGTAAATCACGACCATATTCAGGGAGTGATTCAATTGAAGAAGAAG  195

Query  183  ACCCGATTAAACGGAGCGAAGCGACTGATCGGGGGAAATCCACACCTCGAACCCATGCGA  242
             |     | |||  || ||||    | ||||| |||||||| || || ||    |||
Sbjct  196  GCAAAGATGAACACAGTGAAGAACATAATCGGAGGAAATCCTCATCTGGAGAAGATGAAG  255

Query  243  GGTTCAATTACAGAGGCGAAAGCCTACTGTAC-GAAAGAACAATCACGGATCGCAGGTCC  301
            ||||| ||    ||| ||    || || ||    | ||||||| ||||| |  |||| || ||
Sbjct  256  GGTTCGATAGAAGAAGCTTCTGCGTA-TGCCCAGAAAGAAGAATCAAGAGTCGCCGGACC  314
```

```
Query  302  GTGGGAATTCGGAGAAATGCTGCTGAAGGG--GTCGAACAGACGGAAGCTCGCAGAGCTT  359
             |||    | ||| ||| | |||  ||| ||  |||  ||| ||||||| |     ||| |
Sbjct  315  CTGGAGTTACGGTGAATTACTGAAGAAAGGTAGTC--ACAAACGGAAGATTATGGAGTTA  372

Query  360  CTGGATGATCCCGATAACGAAATAAATGAACCTCAAAAATATAGACGAGCGATGGCTAAG  419
             |  | ||||| || |||||| |  | ||||| || ||||| ||| |||||||||||  |
Sbjct  373  ATTAAAGATCCGGAGAACGAATTGGAAGAACCCCAGAAATACAGAAGAGCGATGGCTTGG  432

Query  420  TCCGCCATGGATGAATCTCGGAAGCTTGCTGAAG-AGTATGATTTCCCTCACGAACTACG  478
            ||||||||||| |||||||||||||||||||||| || | | ||||||| |    |||
Sbjct  433  TCCGCCATGGACGAATCTCGGAAGCTTGCTGAAGAAGAAGGGTTTCCCT-ATTCACTTTA  491

Query  479  CTCGTGGCAAAAAACCCTAATCTCATTCCTCGAAGAGGAACCGGATGATCGTACTATTTA  538
            |    |||||| ||||   |   |      |   | || || || ||  |||| |||||||||
Sbjct  492  CAGCTGGCAAGAAACAGTGTTGGGCCTATTAGATGAAGAGCCCAATGACCGTACTATTAT  551

Query  539  CTGGGTCTATGGTCCTAATGGAGGAGAAGGTAAAACCCAGTTCGGTAAACACCTGGGATT  598
             |||||||| || || |||||     ||||| ||| | ||||| ||||||   | |||||||
Sbjct  552  TTGGGTCTACGGCCCAAATGGTAATGAAGGAAAATCACAGTTTGGTAAATTCTTGGGATT  611

Query  599  aaaaaaaGGATGGACC--TATTTACCCGGAGGGGAACTGAAAGACATGATGTATCTATAC  656
            ||||||| |||  |||  |||||||| |||||   ||     |||| ||||   ||| |  |
Sbjct  612  AAAAAAA-GAT-TACCTTTATTTACCTGGAGGTAAAACCCAAGATATGACATATATGTTA  669

Query  657  AGTAAGGAGTTAAAAAACCATGTGGTTATTGATTTCCCCAGATGTACGAAAGACTTTGTA  716
            |  ||  |    |||       |||| || || ||| | ||   | ||||     ||| | | ||
Sbjct  670  ATGAAAAATCCAAAGGCAAATGTTGTGATGGATATTCCTCGGTGTAATTCAGAATATTTA  729

Query  717  AGTTATAAATTCCTAGAGATGGTAAAAAATAGAACTGTATATAGTTATAAATACGAACCG  776
            | |||  ||||   | ||   |   | |||||||||||   ||| |||||||||||| |||||
Sbjct  730  AATTACCAATTTATGGAATTAATTAAAAATAGAACAATATTTAGTTATAAATATGAACCA  789

Query  777  ATAGGTTCTATAGTCAGTAACAAAGTTCATGTAGTGGTATTATGTAATTTCCTGCCGGAA  836
             | |||| |||   | | ||| ||| | |||||| | ||||||   |||| |   |||| ||
Sbjct  790  GTTGGTTGTATTATAAATAATAAAATACATGTAATTGTATTAGCTAATGTATTGCCTGAT  849

Query  837  GAAGAAAAAAT--CTCCGGAGACAGATTAATAATAATAAATTGCTAAACAC  885
             | ||||||||    || |  |||||| ||| ||||||   |||| |||| ||
Sbjct  850  TATGAAAAAAATTAGTCAG--GACAGAATAAAAATAATTTATTGTTAAATAC  898
```

Faba bean necrotic yellows virus associated alphasatellite 2 isolate TN-Tuf10_2-2015, complete sequence
Sequence ID: MF510474.1   Length: 1005 Number of Matches: 1
Range 1: 136 to 898

#1 Score	Expect	Identities	Gaps	Strand
274 bits(303)	3e-68	531/771(69%)	16/771(2%)	Plus/Plus

```
Query  123  TGGCAACACGAGAAGGTGGATCACGACCACCTACAGGGAGTGATCCAATTGAAGAAGAAG  182
            ||||||||||| |  ||  ||||||||||   | ||||||||||| |||||||||||||||
Sbjct  136  TGGCAACACGAAAGAGTAAATCACGACCATATTCAGGGAGTGATTCAATTGAAGAAGAAG  195

Query  183  ACCCGATTAAACGGAGCGAAGCGACTGATCGGGGGAAATCCACACCTCGAACCCATGCGA  242
             |      | |||  || ||||     | ||||| |||||||| || || ||     |||
Sbjct  196  GCAAAGATGAACACAGTGAAGAACATAATCGGAGGAAATCCTCATCTGGAGAAGATGAAG  255

Query  243  GGTTCAATTACAGAGGCGAAAGCCTACTGTAC-GAAAGAACAATCACGGATCGCAGGTCC  301
            ||||| ||    ||| ||     || || ||  | ||||||| ||||| |  |||| || ||
Sbjct  256  GGTTCGATAGAAGAAGCTTCTGCGTA-TGCCCAGAAAGAAGAATCAAGAGTCGCCGGACC  314

Query  302  GTGGGAATTCGGAGAAATGCTGCTGAAGGG--GTCGAACAGACGGAAGCTCGCAGAGCTT  359
             |||    | ||| ||| | |||  ||| ||  |||  ||| ||||||| |     ||| |
Sbjct  315  CTGGAGTTACGGTGAATTACTGAAGAAAGGTAGTC--ACAAACGGAAGATTATGGAGTTA  372

Query  360  CTGGATGATCCCGATAACGAAATAAATGAACCTCAAAAATATAGACGAGCGATGGCTAAG  419
             |  | ||||| || |||||| |  | ||||| || ||||| ||| |||||||||||  |
Sbjct  373  ATTAAAGATCCGGAGAACGAATTGGAAGAACCCCAGAAATACAGAAGAGCGATGGCTTGG  432

Query  420  TCCGCCATGGATGAATCTCGGAAGCTTGCTGAAG-AGTATGATTTCCCTCACGAACTACG  478
            ||||||||||| |||||||||||||||||||||| || | | ||||||| |    |||
Sbjct  433  TCCGCCATGGACGAATCTCGGAAGCTTGCTGAAGAAGAAGGGTTTCCCT-ATTCACTTTA  491

Query  479  CTCGTGGCAAAAAACCCTAATCTCATTCCTCGAAGAGGAACCGGATGATCGTACTATTTA  538
            |    |||||| ||||   |   |      |   | || || || ||  |||| |||||||||
Sbjct  492  CAGCTGGCAAGAAACAGTGTTGGGCCTATTAGATGAAGAGCCCAATGACCGTACTATTAT  551

Query  539  CTGGGTCTATGGTCCTAATGGAGGAGAAGGTAAAACCCAGTTCGGTAAACACCTGGGATT  598
             |||||||| || || |||||     ||||| ||| | ||||| ||||||   | |||||||
Sbjct  552  TTGGGTCTACGGCCCAAATGGTAATGAAGGAAAATCACAGTTTGGTAAATTCTTGGGATT  611

Query  599  aaaaaaaGGATGGACC--TATTTACCCGGAGGGGAACTGAAAGACATGATGTATCTATAC  656
            ||||||| |||  |||  |||||||| |||||   ||     |||| ||||   ||| |  |
Sbjct  612  AAAAAAA-GAT-TACCTTTATTTACCTGGAGGTAAAACCCAAGATATGACATATATGTTA  669

Query  657  AGTAAGGAGTTAAAAAACCATGTGGTTATTGATTTCCCCAGATGTACGAAAGACTTTGTA  716
            |  ||  |    |||       |||| || || ||| | ||   | ||||     ||| | | ||
Sbjct  670  ATGAAAAATCCAAAGGCAAATGTTGTGATGGATATTCCTCGGTGTAATTCAGAATATTTA  729

Query  717  AGTTATAAATTCCTAGAGATGGTAAAAAATAGAACTGTATATAGTTATAAATACGAACCG  776
            | |||  ||||   | ||   |   | |||||||||||   ||| |||||||||||| |||||
Sbjct  730  AATTACCAATTTATGGAATTAATTAAAAATAGAACAATATTTAGTTATAAATATGAACCA  789

Query  777  ATAGGTTCTATAGTCAGTAACAAAGTTCATGTAGTGGTATTATGTAATTTCCTGCCGGAA  836
             | |||| |||   | | ||| ||| | |||||| | ||||||   |||| |   |||| ||
Sbjct  790  GTTGGTTGTATTATAAATAATAAAATACATGTAATTGTATTAGCTAATGTATTGCCTGAT  849
```

```
Query  837   GAAGAAAAAAT--CTCCGGAGACAGATTAATAATAATAAATTGCTAAACAC  885
              | ||||||||    || |  |||||| ||| ||||||  |||| |||| ||
Sbjct  850   TATGAAAAAAATTAGTCAG--GACAGAATAAAAATAATTTATTGTTAAATAC  898
```

Milk vetch chlorotic dwarf virus alphasatellite 1 isolate G53, complete sequence
Sequence ID: MN273340.1 Length: 999 Number of Matches: 1
Range 1: 62 to 885

#1 Score	Expect	Identities	Gaps	Strand
262 bits(290)	5e-65	557/827(67%)	6/827(0%)	Plus/Plus

```
Query  48    AATTGGGTGTTCACACTCAATTTCACAGGAGAGACTCCTATC-CTTTCCTTTGGAGAGGA  106
             |||||||| || || || ||||||||||| |    ||||  || || || || |  |||
Sbjct  62    AATTGGGTTTTTACGCTGAATTTCACAGGCGCT-CTCCCTTCTCTCTCGTTCGACGAGAG  120

Query  107   AACACAATATGCGTGTTGGCAACACGAGAAGGTGGATCACGACCACCTACAGGGAGTGAT  166
             |    ||||||||    ||||||||| ||||  ||   | || ||||| || |||||||| ||
Sbjct  121   AGTTCAATATGCAGCTTGGCAACATGAGAGAGTTAACCATGACCATCTCCAGGGAGTAAT  180

Query  167   CCAATTGAAGAAGAAGACCCGATTAAACGGAGCGAAGCGACTGATCGGGGGAAATCCACA  226
              ||||||||||| ||   | ||   | |||    |   |||     |||| ||||||||||| ||
Sbjct  181   TCAATTGAAGAATAAAGCTCGCCTTAACACTGTTAAGGCTATGATTGGGGGAAATCCCCA  240

Query  227   CCTCGAACCCATGCGAGGTTCAATTACAGAGGCGAAAGCCTACTGTACGAAAGAACAATC  286
              || ||     ||| ||||    ||||   ||| ||    ||   |||    | ||||||   | ||
Sbjct  241   TCTGGAGAAGATGAGAGGGAAAATTGAAGAAGCATCAGAATACGCCATGAAAGAGGAGTC  300

Query  287   ACGGATCGCAGGTCCGTGGGAATTCGGAGAAATGCTGCTGAAGGG--GTCGAACAGACGG  344
               |   | ||||| || |||    |   || || ||| ||   ||| ||   |||   ||| |||
Sbjct  301   GAGAGTTGCAGGACCTTGGATTTATGGCGAGATGTTGAAGAAAGGAAGTC--ACAAACGT  358

Query  345   AAGCTCGCAGAGCTTCTGGATGATCCCGATAACGAAATAAATGAACCTCAAAAATATAGA  404
             ||| |     ||| |   | || ||||| || ||||||  |   | ||||| |||||||   |||
Sbjct  359   AAGATTATGGAGTTAATTGAAGATCCTGAGAACGAATTGGAAGAACCCCAAAAATTCAGA  418

Query  405   CGAGCGATGGCTAAGTCCGCCATGGATGAATCTCGGAAGCTTGCTGAAGAGTATGATTTC  464
              |||||||||||  ||||||||||||  |||||||||||||||||    |||| |  |   ||
Sbjct  419   AGAGCGATGGCTTGGTCCGCCATGGAAGAATCTCGGAAGCTTGCTTCAGAGGAAGGGTTT  478

Query  465   CCTCACGAACTACGCTCGTGGCAAAAAACCCTAATCTCATTCCTCGAAGAGGAACCGGAT  524
             |||||    |||    |    |||||| ||| |      | |||  |   | |||  | || || |||
Sbjct  479   CCTCATACACTTTACAGCTGGCAAGAAAACGCGTTGTCACTGTTGGAAAACGACCCTGAT  538

Query  525   GATCGTACTATTTACTGGGTCTATGGTCCTAATGGAGGAGAAGGTAAAACCCAGTTCGGT  584
             |||||||| ||    |||||| ||||| ||   | ||     ||||| ||| | ||||| |||
Sbjct  539   GATCGTACAATCATCTGGGTGTATGGGCCCCACGGTAATGAAGGAAAATCACAGTTTGGT  598

Query  585   AAACACCTGGGATTaaaaaaaGGATGGACCTATTTACCCGGAGGGGAACTGAAAGACATG  644
             |||   |||||| ||||||||||   |     ||||||||| || ||   ||     |||| |||
Sbjct  599   AAATTCCTGGGGTTAAAAAAAGATTACCTCTATTTACCAGGGGGTAAAACACAAGATATG  658

Query  645   ATGTATCTATACAGTAAGGAGTTAAAAAACCATGTGGTTATTGATTTCCCCAGATGTACG  704
             | |||   |      |   ||| |    ||||     |||| || |||||| | ||   | ||||
Sbjct  659   ACGTACATGCTGATGAAGAACCCAAAAGCTAATGTTGTAATTGATATTCCACGCTGTAAT  718

Query  705   AAAGACTTTGTAAGTTATAAATTCCTAGAGATGGTAAAAAATAGAACTGTATATAGTTAT  764
               ||| | | | | |||| ||||   | ||   |   |||||||||||||    | | |||||||
Sbjct  719   TCAGAATATCTGAATTATCAATTTATGGAATTAATAAAAAATAGAACAATTTTTAGTTAT  778

Query  765   AAATACGAACCGATAGGTTCTATAGTCAGTAACAAAGTTCATGTAGTGGTATTATGTAAT  824
             ||||| || ||   | |||| ||| || | ||| ||| | |||||| | |||||     |||
Sbjct  779   AAATATGAGCCAGTTGGTTGTATTGTAAATAATAAAATACATGTAATTGTATTGGCCAAT  838

Query  825   TTCCTGCCGGAAGAAGAAAAAATCTCCGGAGACAGATTAATAATAAT  871
              |  |||| ||  | ||||||||       ||||||| ||| ||| ||
Sbjct  839   GTATTGCCAGATTATGAAAAAAATAAGTCAAGACAGAATAAAAATGAT  885
```

Milk vetch chlorotic dwarf virus alphasatellite 1 isolate G50, complete sequence
Sequence ID: MN273330.1 Length: 999 Number of Matches: 1
Range 1: 62 to 885

#1 Score	Expect	Identities	Gaps	Strand
262 bits(290)	5e-65	557/827(67%)	6/827(0%)	Plus/Plus

```
Query  48    AATTGGGTGTTCACACTCAATTTCACAGGAGAGACTCCTATC-CTTTCCTTTGGAGAGGA  106
             |||||||| || || || ||||||||||| |    ||||  || || || || |  |||
Sbjct  62    AATTGGGTTTTTACGCTGAATTTCACAGGCGCT-CTCCCTTCTCTCTCGTTCGACGAGAG  120

Query  107   AACACAATATGCGTGTTGGCAACACGAGAAGGTGGATCACGACCACCTACAGGGAGTGAT  166
             |    ||||||||    ||||||||| ||||  ||   | || ||||| || |||||||| ||
Sbjct  121   AGTTCAATATGCAGCTTGGCAACATGAGAGAGTTAACCATGACCATCTCCAGGGAGTAAT  180

Query  167   CCAATTGAAGAAGAAGACCCGATTAAACGGAGCGAAGCGACTGATCGGGGGAAATCCACA  226
              ||||||||||| ||   | ||   | |||    |   |||     |||| ||||||||||| ||
Sbjct  181   TCAATTGAAGAATAAAGCTCGCCTTAACACTGTTAAGGCTATGATTGGGGGAAATCCCCA  240

Query  227   CCTCGAACCCATGCGAGGTTCAATTACAGAGGCGAAAGCCTACTGTACGAAAGAACAATC  286
              || ||     ||| ||||    ||||   ||| ||    ||   |||    | ||||||   | ||
Sbjct  241   TCTGGAGAAGATGAGAGGGAAAATTGAAGAAGCATCAGAATACGCCATGAAAGAGGAGTC  300

Query  287   ACGGATCGCAGGTCCGTGGGAATTCGGAGAAATGCTGCTGAAGGG--GTCGAACAGACGG  344
               |   | ||||| || |||    |   || || ||| ||   ||| ||   |||   ||| |||
Sbjct  301   GAGAGTTGCAGGACCTTGGATTTATGGCGAGATGTTGAAGAAAGGAAGTC--ACAAACGT  358
```

```
Query  345  AAGCTCGCAGAGCTTCTGGATGATCCCGATAACGAAATAAATGAACCTCAAAAATATAGA  404
            ||| |    ||| |  | || ||||| || |||||| |  | ||||| |||||||  |||
Sbjct  359  AAGATTATGGAGTTAATTGAAGATCCTGAGAACGAATTGGAAGAACCCCAAAAATTCAGA  418

Query  405  CGAGCGATGGCTAAGTCCGCCATGGATGAATCTCGGAAGCTTGCTGAAGAGTATGATTTC  464
             |||||||||||  |||||||||||| ||||||||||||||||||  |||| | |  || 
Sbjct  419  AGAGCGATGGCTTGGTCCGCCATGGAAGAATCTCGGAAGCTTGCTTCAGAGGAAGGGTTT  478

Query  465  CCTCACGAACTACGCTCGTGGCAAAAAACCCTAATCTCATTCCTCGAAGAGGAACCGGAT  524
            |||||   |||   |   |||||| ||| |    | ||| |  | ||| | || || |||
Sbjct  479  CCTCATACACTTTACAGCTGGCAAGAAAACGCGTTGTCACTGTTGGAAAACGACCCTGAT  538

Query  525  GATCGTACTATTTACTGGGTCTATGGTCCTAATGGAGGAGAAGGTAAAACCCAGTTCGGT  584
            |||||||| ||   |||||| ||||| ||  | ||    ||||| ||| | ||||| |||
Sbjct  539  GATCGTACAATCATCTGGGTGTATGGGCCCCACGGTAATGAAGGAAAATCACAGTTTGGT  598

Query  585  AAACACCTGGGATTaaaaaaaGGATGGACCTATTTACCCGGAGGGGAACTGAAAGACATG  644
            |||  |||||| ||||||||||  |    |||||||||| ||  ||    |||| |||
Sbjct  599  AAATTCCTGGGGTTAAAAAAAGATTACCTCTATTTACCAGGGGGTAAAACACAAGATATG  658

Query  645  ATGTATCTATACAGTAAGGAGTTAAAAAACCATGTGGTTATTGATTTCCCCAGATGTACG  704
            | |||  |    |  ||| |   ||||    |||| || |||||| | ||  | ||||  
Sbjct  659  ACGTACATGCTGATGAAGAACCCAAAAGCTAATGTTGTAATTGATATTCCACGCTGTAAT  718

Query  705  AAAGACTTTGTAAGTTATAAATTCCTAGAGATGGTAAAAAATAGAACTGTATATAGTTAT  764
              ||| | | | | |||| ||||  | ||  |  |||||||||||||  | | |||||||
Sbjct  719  TCAGAATATCTGAATTATCAATTTATGGAATTAATAAAAAATAGAACAATTTTTAGTTAT  778

Query  765  AAATACGAACCGATAGGTTCTATAGTCAGTAACAAAGTTCATGTAGTGGTATTATGTAAT  824
            ||||| || ||  | |||| ||| || | ||| ||| | |||||| | |||||    |||
Sbjct  779  AAATATGAGCCAGTTGGTTGTATTGTAAATAATAAAATACATGTAATTGTATTGGCCAAT  838

Query  825  TTCCTGCCGGAAGAAGAAAAAATCTCCGGAGACAGATTAATAATAAT  871
             |  |||| ||  | ||||||||      ||||||| ||| ||| ||
Sbjct  839  GTATTGCCAGATTATGAAAAAATAAGTCAAGACAGAATAAAAATGAT  885
```

Faba bean necrotic yellows C9 alphasatellite C9-Eg gene, isolate Egyptian EV1-93
Sequence ID: AJ132187.1 Length: 1007 Number of Matches: 1
Range 1: 85 to 918

#1 Score	Expect	Identities	Gaps	Strand
256 bits(283)	7e-63	572/844(68%)	20/844(2%)	Plus/Plus

```
Query  48   AATTGGGTGTTCACACTCAATTTCACAGGAGAGACTCCTATCCTTTCCTTTGGAGAGGAA  107
            || ||||| |||||  | || ||| | || ||   |||| | || || || |  |||  |
Sbjct  85   AACTGGGTTTTCACGTTGAACTTCGCCGGCGAAGTTCCTGTTCTCTCGTTCGACGAGAGA  144

Query  108  ACACAATATGCGTGTTGGCAACACGAGAAGGTGGATCACGACCACCTACAGGGAGTGATC  167
               ||||| ||    ||||||||||| |  ||  ||||||||||  | ||||||||||| 
Sbjct  145  GTTCAATACGCCGTCTGGCAACACGAAAGAGTAAATCACGACCATATTCAGGGAGTGATT  204

Query  168  CAATTGAAGAAGAAGACCCGATTAAACGGAGCGAAGCGACTGATCGGGGGAAATCCACAC  227
            ||||| ||||||||| |     | |||  || ||||    | || || |||||||| || 
Sbjct  205  CAATTAAAGAAGAAGGCAAAGATGAACACAGTGAAGAACATCATTGGTGGAAATCCTCAT  264

Query  228  CTCGAACCCATGCGAGGTTCAATTACAGAGGCGAAAGCCTACTGTAC-GAAAGAACAATC  286
            || ||    |||   ||||| ||   ||| ||    || || ||  | ||||||| ||||
Sbjct  265  CTGGAGAAGATGAAGGGTTCGATAGAAGAAGCTTCTGCGTA-TGCCCAGAAAGAAGAATC  323

Query  287  ACGGATCGCAGGTCCGTGGGAATTCGGAGAAATGCTGCTGAAGGG--GTCGAACAGACGG  344
            | |  |||| || || |||   | ||| ||| |  ||  ||| ||  |||  | | ||||
Sbjct  324  AAGAGTCGCCGGACCCTGGAGTTACGGTGAATTATTGAAGAAAGGTAGTC--ATAAACGG  381

Query  345  AAGCTCGCAGAGCTTCTGGATGATCCCGATAACGAAATAAATGAACCTCAAAAATATAGA  404
            ||| |    ||| |  |  | ||||| || |||||| |  | ||||| || ||||| |||
Sbjct  382  AAGATTATGGAGTTAATTAAAGATCCGGAGAACGAATTGGAAGAACCCCAGAAATACAGA  441

Query  405  CGAGCGATGGCTAAGTCCGCCATGGATGAATCTCGGAAGCTTGCTGAAG-AGTATGATTT  463
             |||||||||||  |||||||||||| |||||||||||||||||||||| || | | |||
Sbjct  442  AGAGCGATGGCTTGGTCCGCCATGGACGAATCTCGGAAGCTTGCTGAAGAAGGAGGCTTT  501

Query  464  CCCT-CACGAACTAC-GCTCGTGGCAAAAAACCCTAATCTCATTCCTCGAAGAGGAACCG  521
            ||||  |||   ||| ||   |||||| ||||  |  |     |  | ||||| || || 
Sbjct  502  CCCTATACGCTTTACAGC---TGGCAAGAAACAGTGTTGGGCCTATTAGAAGAAGAGCCC  558

Query  522  GATGATCGTACTATTTACTGGGTCTATGGTCCTAATGGAGGAGAAGGTAAAACCCAGTTC  581
             |||| |||| ||||   |||||||| || || |||||    ||||| ||| | ||||| 
Sbjct  559  AATGACCGTATTATTATTTGGGTCTACGGCCCAAATGGTAACGAAGGAAAATCACAGTTT  618

Query  582  GGTAAACACCTGGGATTaaaaaaaGGATGGACC--TATTTACCCGGAGGGGAACTGAAAG  639
            ||||||  |||||||||||||||| |||  |||  |||||||| |||||  ||    |||
Sbjct  619  GGTAAATTCCTGGGATTAAAAAAA-GAT-TACCTTTATTTACCTGGAGGTAAAACCCAAG  676

Query  640  ACATGATGTATCTATACAGTAAGGAGTTAAAAAACCATGTGGTTATTGATTTCCCCAGAT  699
            | ||||  ||| | |  |  ||  |   |||     |||| || || ||| | ||  | |
Sbjct  677  ATATGACATATATGTTAATGAAAAATCCAAAGGCAAATGTTGTGATGGATATTCCTCGTT  736

Query  700  GTACGAAAGACTTTGTAAGTTATAAATTCCTAGAGATGGTAAAAAATAGAACTGTATATA  759
            |||     || | | ||| |||| ||||  | ||  |  | |||||||||||  ||| ||
Sbjct  737  GTAATTCTGAATATTTAAATTATCAATTTATGGAATTAATTAAAAATAGAACCATATTTA  796

Query  760  GTTATAAATACGAACCGATAGGTTCTATAGTCAGTAACAAAGTTCATGTAGTGGTATTAT  819
            |||||||||| |||||  | || | |||  | | ||| ||| | |||||| | |||||| 
Sbjct  797  GTTATAAATATGAACCAGTTGGATGTATTATAAATAATAAAATACATGTAATTGTATTAG  856
```

```
Query  820  GTAATTTCCTGCCGGAAGAAGAAAAAAT--CTCCGGAGACAGATTAATAATAATAAATTG  877
             |||| |  |||| ||  | ||||||||   || |  |||||| | | ||||||  ||||
Sbjct  857  CTAATGTATTGCCTGATTATGAAAAAATTAGTCAG--GACAGAATTAAAATAATTTATTG  914

Query  878  CTAA  881
             |||
Sbjct  915  TTAA  918
```

...
